# Cationic Pd(II)-catalyzed C–H activation/cross-coupling reactions at room temperature: synthetic and mechanistic studies

**DOI:** 10.3762/bjoc.12.99

**Published:** 2016-05-20

**Authors:** Takashi Nishikata, Alexander R Abela, Shenlin Huang, Bruce H Lipshutz

**Affiliations:** 1 Department of Chemistry & Biochemistry, University of California, Santa Barbara, CA 93106, USA

**Keywords:** arylation, cationic palladium, C–H functionalization, green chemistry, olefination

## Abstract

Cationic palladium(II) complexes have been found to be highly reactive towards aromatic C–H activation of arylureas at room temperature. A commercially available catalyst [Pd(MeCN)_4_](BF_4_)_2_ or a nitrile-free cationic palladium(II) complex generated in situ from the reaction of Pd(OAc)_2_ and HBF_4_, effectively catalyzes C–H activation/cross-coupling reactions between aryl iodides, arylboronic acids and acrylates under milder conditions than those previously reported. The nature of the directing group was found to be critical for achieving room temperature conditions, with the urea moiety the most effective in promoting facile coupling reactions at an *ortho* C–H position. This methodology has been utilized in a streamlined and efficient synthesis of boscalid, an agent produced on the kiloton scale annually and used to control a range of plant pathogens in broadacre and horticultural crops. Mechanistic investigations led to a proposed catalytic cycle involving three steps: (1) C–H activation to generate a cationic palladacycle; (2) reaction of the cationic palladacycle with an aryl iodide, arylboronic acid or acrylate, and (3) regeneration of the active cationic palladium catalyst. The reaction between a cationic palladium(II) complex and arylurea allowed the formation and isolation of the corresponding palladacycle intermediate, characterized by X-ray analysis. Roles of various additives in the stepwise process have also been studied.

## Introduction

Transition metal-catalyzed, direct functionalization of aryl C–H bonds has made enormous progress over the past decade, and continues to attract a great deal of attention due to the highly efficient routes now available for elaborating aromatic rings. While reactions of this type have been known for decades [1–71], serious challenges remain in achieving reactivity and selectivity due to the inertness and ubiquity of C–H bonds. High temperatures are frequently required to realize aromatic C–H functionalization (>120 °C), increasing the potential for side reactions and functional group compatibility issues. Indeed, C–H activation transformations until recently have rarely proceeded at ambient temperature due to the typically low reactivity of these positions [72–79]. In the case of palladium-catalyzed C–H activation, the crucial, namesake “C–H activation” step typically involves a C–H to C–Pd refunctionalization, generating a reactive aryl-palladium species that is poised for further transformations.

**Figure 1:**
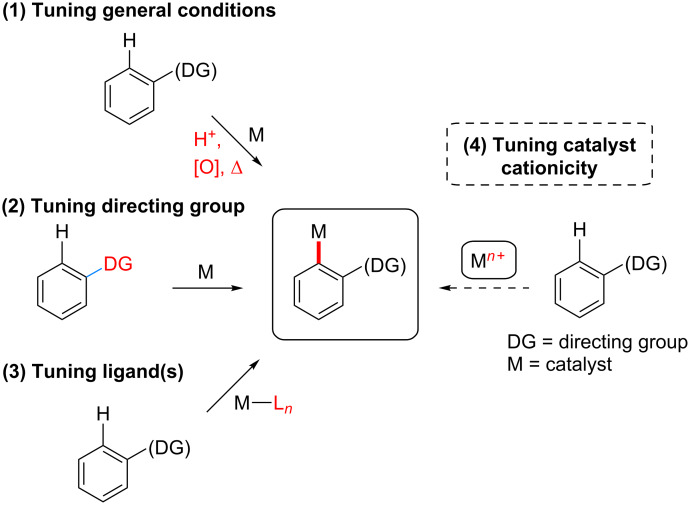
Road map to enhanced C–H activation reactivity.

Three approaches (Figure 1) have generally been employed to enhance the reactivity and promote the key metalation/C–H bond cleavage step: (1) tuning of the reaction conditions through inclusion of various additives such as metal salts [1–22], or strong acids such as TFA or HOAc, in addition to the application of heat, although it is not always clear which steps within the overall mechanism are most directly effected under these conditions; (2) in a major subset of C–H activation chemistry, internally chelating *ortho*-directing groups [71–85] have been found to effectively promote selective C–H activation, typically by aiding in the formation of a palladacycle intermediate. Careful tuning of the structure of the directing group, with functionalities including a variety of nitrogen-containing moieties, such as amides [86–87], *N*-heterocycles [88–89], imines [90–91], pyridine *N*-oxide [92], amines [93–94], as well as a variety of others [1–71], has been found to profoundly impact reactivity; (3) tuning of ligands around the transition metal catalyst center has emerged as an especially powerful means of enhancing and controlling reactivity in these processes [95–107].

**Scheme 1:**
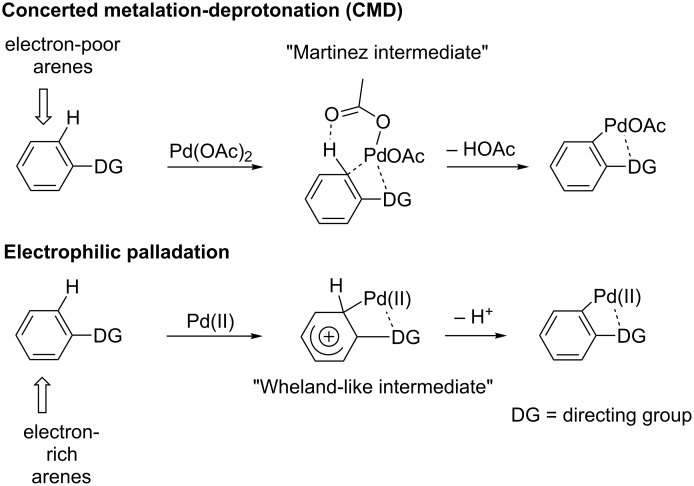
Concerted metalation–deprotonation and elelectrophilic palladation pathways for C–H activation.

A fourth approach with considerable potential, and which appears to have received considerably less attention, involves tuning the cationicity of the transition metal catalyst [72–79]. Literature studies have suggested that certain anionic ligands on palladium, such as acetate or carbonate, may assist C–H bond cleavage by acting as internal bases as part of a concerted metalation–deprotonation (CMD) pathway, particularly in the case of less electron-rich arenes (Scheme 1, top) [34,108–119]. In other arrays, particularly those with more electron-rich substituents, evidence suggests an electrophilic aromatic substitution mechanism may be operative. In these instances, electron-poor catalysts, such as those generated from the reaction of Pd(II) and TFA, have in some cases been shown to be especially effective. We reasoned that substitution with a more distant coordinating anion would result in a highly Lewis acidic, dicationic palladium species that might be still more reactive in the electrophilic palladation step, potentially gaining entry to C–H activation under even milder reaction conditions for selected couplings than have previously been observed (Scheme 1, bottom) [120–122].

Metal cations, in general, are well known to increase the reactivity of C–C and C–N double bonds due to their Lewis acidity. Cationic palladium complexes [123], in particular, possess a wide breadth of reactivity, having been used to catalyze Diels–Alder [124–125], aldol and Mannich reactions [126–128], Wacker oxidations [129], polymerizations of alkenes [130–131], and asymmetric 1,4-additions with arylboronic [132–134], arylbismuth [135], and arylsilicon [136] reagents. Although carbocations react with arenes through electrophilic aromatic hydrogen substitution in a Friedel–Crafts reaction, the potential for metal cations to participate in similar chemistry has been far less widely examined. A cationic palladium-catalyzed electrophilic aromatic C–H substitution without basic anions [137–142] would hold considerable promise as an alternative and potentially milder approach to achieving valued C–H activation/coupling reactions.

**Scheme 2:**
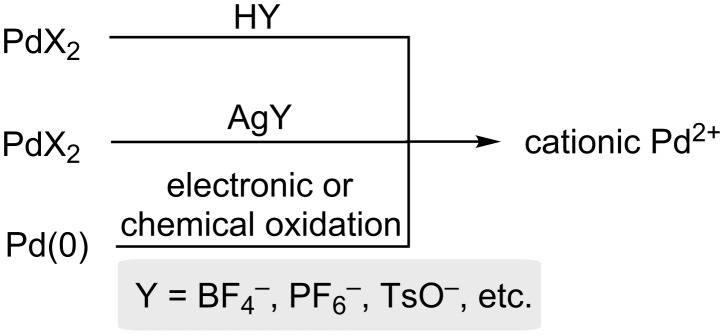
Routes for generation of cationic palladium(II) species.

While several cationic palladium complexes are commercially available, they may also be generated in situ via a variety of routes (Scheme 2), including: (a) reaction of a palladium complex with a non-coordinating anion source, usually an acid or metal salt; (b) reaction of Pd(II) halide complexes and silver salts [143–145]; (c) electronic oxidation of Pd(0) [146]; and (d) chemical oxidation of Pd(0) with HBF_4_, Cu(BF_4_)_2_ or AgBF_4_ [136,147–148].

We have previously reported that cationic palladium-catalyzed C–H arylations of arylureas [121–122] and Fujiwara–Moritani reactions of anilide derivatives [148] can be effected at room temperature. In this account we disclose, in addition to full details associated with this C–H activation chemistry, additional applications of room temperature Fujiwara–Moritani reactions including a synthesis of the herbicide boscalid, as well as spectroscopic and mechanistic studies.

## Results and Discussion

### C–H arylations of arylureas with aryl iodides and arylboronic acids

**Scheme 3:**
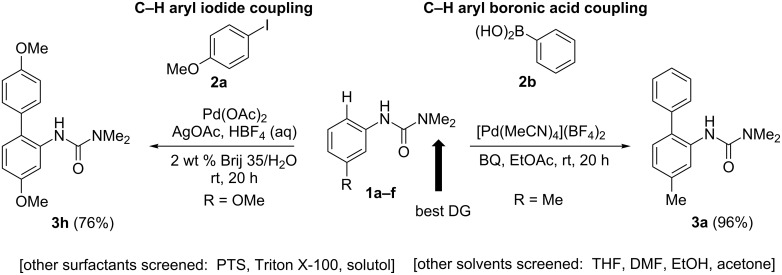
Optimized conditions for C–H arylations at room temperature.

Among the most conceptually attractive approaches to aromatic C–H activation is the efficient synthesis of biaryls through direct arylation reactions. The widespread availability of aryl iodides and arylboronic acids make them appealing arylating agents [149–171]. Previously reported C–H activation reaction conditions employing these reagents, however, have typically required high temperatures to obtain the desired coupling products in good yields. In order to successfully carry out these reactions at ambient temperature, several considerations must be addressed, as illustrated in Figure 1. Optimization studies initially focused on the choice of an *ortho*-directing group together with a cationic palladium(II) catalyst. Although the combination of acetanilide together with a palladium(II) catalyst lead to the corresponding palladacycle, as reported by Tremont [172], in the presence of **2a**, Pd(OAc)_2_, HBF_4_ and AgOAc at room temperature, acetyl or isopropyl anilides afford essentially no product. Only after heating to 50 °C did substrate **1a** react with iodide **2a**. The corresponding pivaloylanilide is also known to serve as an effective directing group at 130 °C, but at room temperature a poor yield was obtained. Only the dimethylurea analog gave satisfactory conversion to the desired biaryl, thus arriving at optimized conditions, as shown in Scheme 3 (left). By contrast, the Daugulis group and others [173–174] have demonstrated Pd-catalyzed *ortho-*arylations of anilides at temperatures typically greater than 100 °C, and the Sanford group has also studied similar transformations involving diaryliodonium salts [175]. Arylureas have recently been noticed to be more active coupling partners for C–H functionalizations as opposed to anilides, especially at lower temperatures [176]. A number of strong acids have been previously utilized in C–H activation reactions [1–22], e.g., HBF_4_ was found to be critical for generation of biaryl **3h** in good yield. The structure of the *ortho-*arylated product was confirmed by X-ray analysis. While similar reactions, in addition to requiring high temperatures, have typically employed strong acids such as TFA as the organic solvent, here 2 wt % solutions of selected surfactants in water were found to be excellent reaction media, providing an additional environmentally appealing feature to this protocol. While good yields could be obtained using the first generation surfactant PTS (polyoxyethanyl α-tocopheryl sebacate) [121,177–178], several other amphiphiles that are both less costly and are items of commerce gave comparable results. Using commercialy available Brij 35 (2 wt %) in water [179–183] afforded the best levels of conversion and thus, overall yields, while in its absence (i.e.,”on water”), there was a noticeable drop in the extent of conversion. In addition, lower loadings of HBF_4_, silver salt, or the palladium catalyst also gave inferior results.

**Scheme 4:**
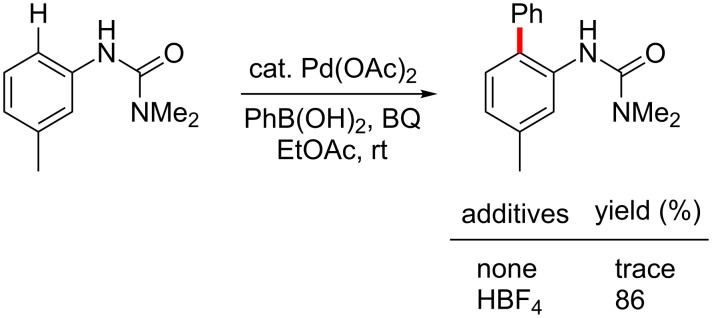
Biaryl formation catalyzed by Pd(OAc)_2_.

In the case of C–H activation/Suzuki–Miyaura coupling reactions, the commercially available, pre-formed cationic Pd(II) catalyst [Pd(MeCN)_4_](BF_4_)_2_ [184], was found to efficiently catalyze the reaction between arylureas and arylboronic acids. On the other hand, C–H arylations with aryl iodides catalyzed by [Pd(MeCN)_4_](BF_4_)_2_ did not give any of the desired products (see mechanistic discussion; vide infra). Various neutral palladium catalysts were examined, such as Pd(OAc)_2_, PdCl_2_L*_n_*, Pd_2_(dba)_3_, in the absence of added acid, but none led to cross-coupling at room temperature. 1,4-Benzoquinone (BQ) was found to be an effective additive in promoting the reaction, while addition of stoichiometric metal salts (e.g., silver or copper salts) was unnecessary. Moreover, in this case organic solvents were far more effective as the reaction medium than was water, possibly due to BQ solubility issues. EtOAc, rather than EtOH and THF was the most effective (Scheme 3, right), while other organic solvents (e.g., DMF) gave low-to-moderate yields of product **3a**. Although reduced amounts of both phenylboronic acid (**2b**) and BQ still gave excellent yields, lower catalyst loadings caused slower reactions. A neutral palladium(II) complex, Pd(OAc)_2_, showed no catalytic acitivity, whereas catalytic Pd(OAc)_2_ in the presence of stoichiometric HBF_4_ reacted with an arylurea and arylboronic acid to afford the biaryl in high yield (Scheme 4).

**Figure 2:**
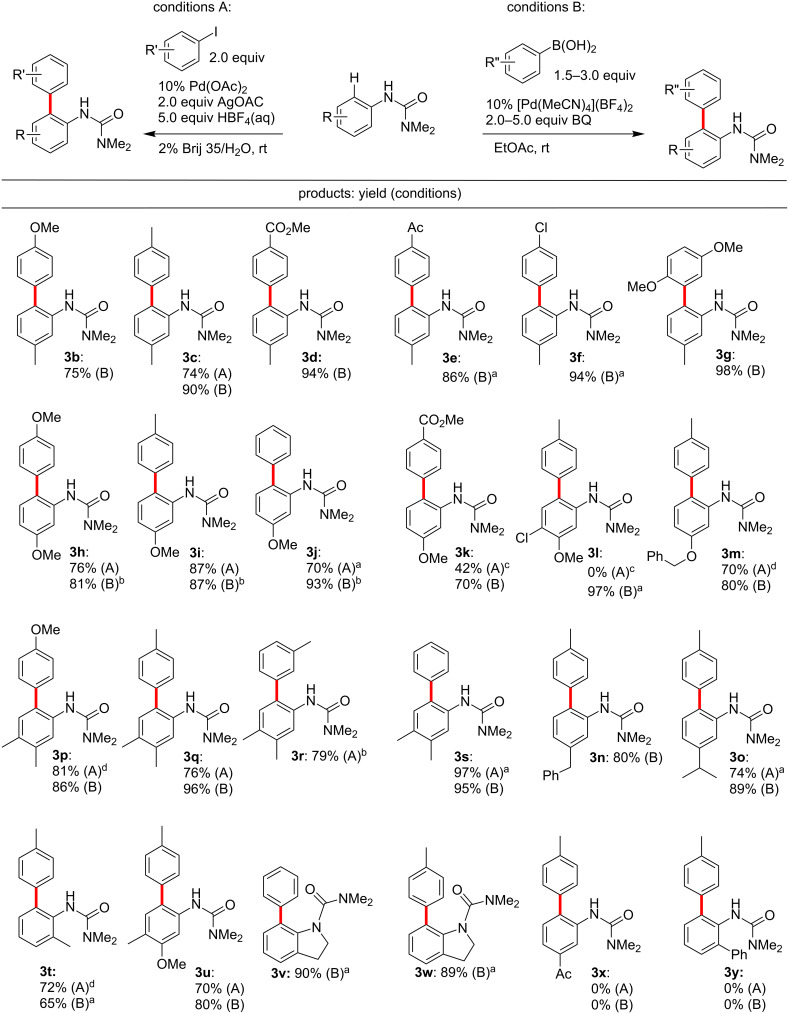
C–H arylation results. Conditions A: Conducted at rt for 20 h in 2 wt % Brij 35/water (1 mL) with 10 mol % Pd(OAc)_2_, AgOAc (2 equiv), HBF_4_ (5 equiv), arylurea (**1**, 0.25 mmol), and ArI (2.0 equiv). Conditions B: Conducted at rt for 20 h in EtOAc (1 mL) with 10 mol % [Pd(MeCN)_4_](BF_4_)_2_, BQ (2 or 5 equiv), arylurea (**1**, 0.25 mmol), and ArB(OH)_2_ (1.5 or 3 equiv). ^a^Run for 48 h. ^b^2 equiv of BQ. ^c^Run for 96 h. ^d^Run for 72 h.

Representative results for the reactions between aryl iodides and arylboronic acids are summarized in Figure 2. These arylations tolerate various combinations of substrates and reagents having electron-withdrawing or electron-donating groups, as well as sterically hindered aromatic rings, all taking place at room temperature. Suzuki–Miyaura-type C–H coupling reactions are typically more tolerant of electron-withdrawing groups (**3d**, **3f**, **3k**) and *ortho*-substitution (**3g**) on the aryl ring. On the other hand, the reaction with 4-methoxycarbonylphenyl iodide, for example, gave a low yield of product **3k**. Arylureas having only an electron-withdrawing group showed no reactivity towards coupling under either set of conditions (**3x**), consistent with an electrophilic aromatic substitution pathway in the initial C–H activation step by a cationic palladium(II) species (vide infra). Arylureas with various alkyl substituents in the *ortho*-position, including cyclic arrays, gave good isolated yields (**3t**, **3v**, **3w**), whereas a 2-phenyl substituted arylurea did not participate in the C–H activation/coupling reaction under these conditions (**3y**). Overall, C–H Suzuki–Miyaura coupling reactions were applicable to a broader substrate scope than the corresponding reaction with aryl iodides, although the latter protocol remains appealing for a variety of cross-coupling combinations due to both the convenience of aryl iodides as substrates, and the use of water as the gross reaction medium.

**Figure 3:**
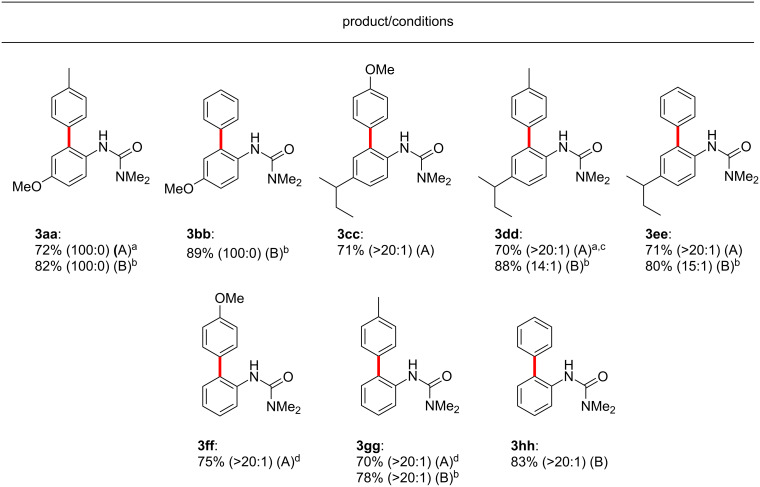
Monoarylations in water at rt. Conditions A: Conducted at rt for 20 h in 2 wt % Brij 35/water with 10 mol % Pd(OAc)_2_, AgOAc (2 equiv), HBF_4_ (5 equiv), arylurea (**1**, 0.25 mmol), and ArI (**2**, 1.5 equiv). Conditions B: Conducted at rt for 20 h in EtOAc with 10 mol % [Pd(MeCN)_4_](BF_4_)_2_, BQ (5 equiv), arylurea (**1**, 0.25 mmol), and ArB(OH)_2_ (**2**, 1.5 equiv). The ratios of single:double arylation determined by ^1^H NMR are shown in the parentheses. ^a^Run for 48 h. ^b^1.2 equiv of ArB(OH)_2_. ^c^1.2 equiv of ArI. ^d^Run for 72 h.

Especially noteworthy are the numerous examples of aniline derivatives lacking *ortho-* or *meta-*substitution, which have previously been shown to be prone to double arylation (Figure 3)**.** Since literature conditions generally employ elevated temperatures, directed C–H arylations have often suffered from uncontrollable double arylation in symmetrical or unsubstituted substrates [1–22,173–174]. At ambient temperatures, however, coupling reactions on these more challenging substrate types underwent selective *mono*-arylations in water (Figure 3). In fact, doubly arylated products were rather difficult to generate under these room temperature conditions, not unexpected given the previously described low reactivity of ureas already possessing an *ortho-*aryl substituent [121–122].

**Scheme 5:**
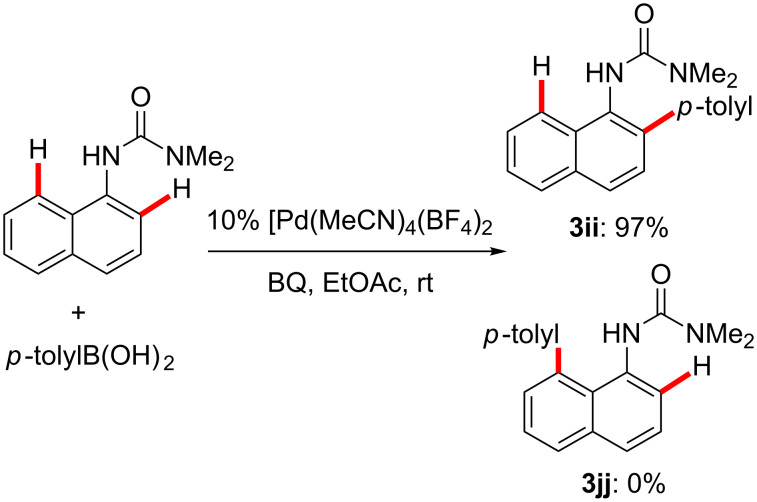
Selective arylation of a 1-naphthylurea derivative.

A 1-naphthylurea also gave excellent selectivity at room temperature (Scheme 5). When this substrate was subjected to optimized conditions for the boronic acid C–H coupling, the corresponding singly *ortho*-arylated product was obtained in 97% yield solely as the 2-aryl isomer, as confirmed by ^1^H NMR and X-ray crystallography. Generally, it is difficult to efficiently control the selectivity between the C2 and C8 positions in naphthalene rings towards a single isomeric C–H activation product [163,174,185–189].

### Fujiwara–Moritani reactions

Following these results on biaryl constructions via C–H activation at room temperature, we next sought to apply our cationic palladium(II) conditions to the venerable Fujiwara–Moritani reaction. As reported back in 1967, this direct aryl olefination reaction is among the first palladium-catalyzed C–H activation reactions to be described [190–192]. Subsequent studies have generally resorted to elevated temperatures (80–160 °C) and anhydrous conditions, and in many cases high pressures of CO or O_2_ are also required in order to carry out these Heck-like coupling reactions [1–22,193–203]. Additional progress of note includes coupling reactions with arenes containing an *ortho*-directing group [1–22,45–51,193–203], as well as a *meta*-selective Fujiwara–Moritani reaction [204–205]. A recent report employing arylureas as the C–H coupling partner achieved limited coupling at ambient temperature, with most examples requiring heating to 60 °C [74,206].

**Figure 4:**
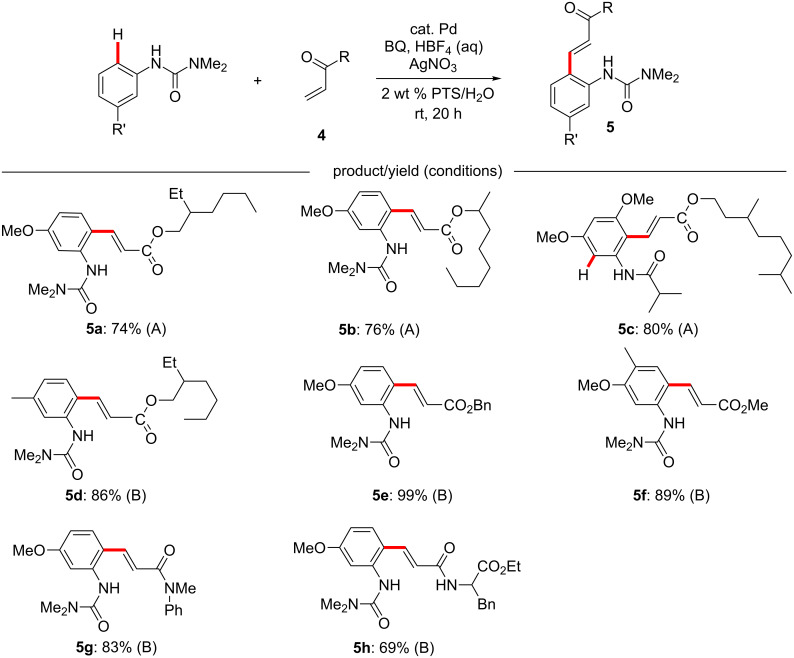
Fujiwara–Moritani coupling rreactions in water. Conditions A: 10 mol % [Pd(MeCN)_4_](BF_4_)_2_, 1 equiv BQ, 2 equiv AgNO_3_, 2 wt % PTS/water, rt, 20 h. Conditions B: 10 mol % Pd(OAc)_2_, 1 equiv BQ, 2 equiv AgOAc, 5 equiv HBF_4_ aq, 2 wt % Brij 35/water, rt, 20 h.

**Figure 5:**
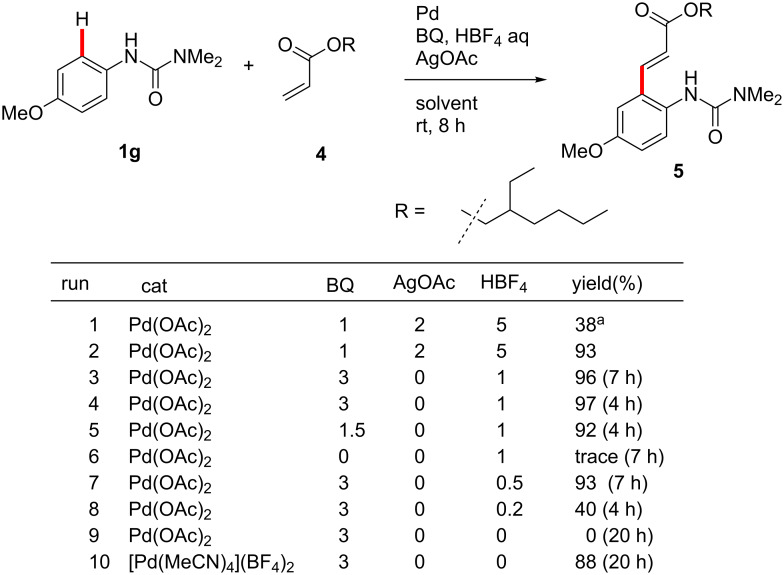
Optimization. Conducted at rt for 8 h or as otherwise noted in EtOAc with 10 mol % Pd catalyst, AgOAc, HBF_4_, arylurea (**1g**, 0.25 mmol), BQ and acrylate (**2c**, 2.0 equiv). ^a^2 wt % Brij 35 in water instead of EtOAc.

We have previously reported a methodology enabling Fujiwara–Moritani reactions to be run in water at room temperature using the cationic palladium catalyst [Pd(MeCN)_4_](BF_4_)_2_ (Figure 4, **5a**–**c**, conditions A). While this reaction proceeded with a number of alkyl anilide derivatives, as well as ureas as directing groups (**5c**), the substrate scope was otherwise somewhat limited; only anilides possessing a strongly donating alkoxy group *meta* to the directing group (*para* to the position where the C–H activation would occur) were reactive. However, we have since found that use of acetonitrile-free, in situ generated cationic palladium with arylureas as the directing group expanded the substrate scope to include reactions with 3-alkyl-substituted ureas, as well as a wider variety of acrylates and even some acrylamides (Figure 4, **5d**–**h**, conditions B). Many combinations of acrylates and more challenging arylureas, however, did not produce the desired product in satisfactory yields, and the reaction still required the use of stoichiometric silver salts in addition to benzoquinone.

To overcome these limitations, further optimization of the catalyst system was conducted (Figure 5). A combination of AgNO_3_ or AgOAc and BQ was critical to obtain good yields of the same products formed earlier in water (vide supra), but as seen previously in the corresponding Suzuki–Miyaura reactions, a switch to EtOAc obviated the need for a silver salt. In the presence of BQ and HBF_4_, the reaction of **1g** and acrylate **4** was efficiently catalyzed by Pd(OAc)_2_ (Figure 5, runs 3 and 4). Lower loadings of BQ and HBF_4_ also gave good results (Figure 5, runs 5 and 7). Much lower loading of HBF_4_, however, afforded a low yield of product **5** (Figure 5, run 8). In the absence of acid or BQ, the product was not obtained (Figure 5, runs 6 and 9). The pre-formed cationic palladium(II) complex, [Pd(MeCN)_4_](BF_4_)_2_, was also found to effectively catalyze the reaction between **1g** and ester **4** at room temperature, without additional acid, although somewhat longer reaction times were necessary (Figure 5, run 10). Despite the presence of two potentially reactive *ortho*-aromatic C–H bonds in **1g**, the mono-acrylated product was obtained exclusively.

**Figure 6:**
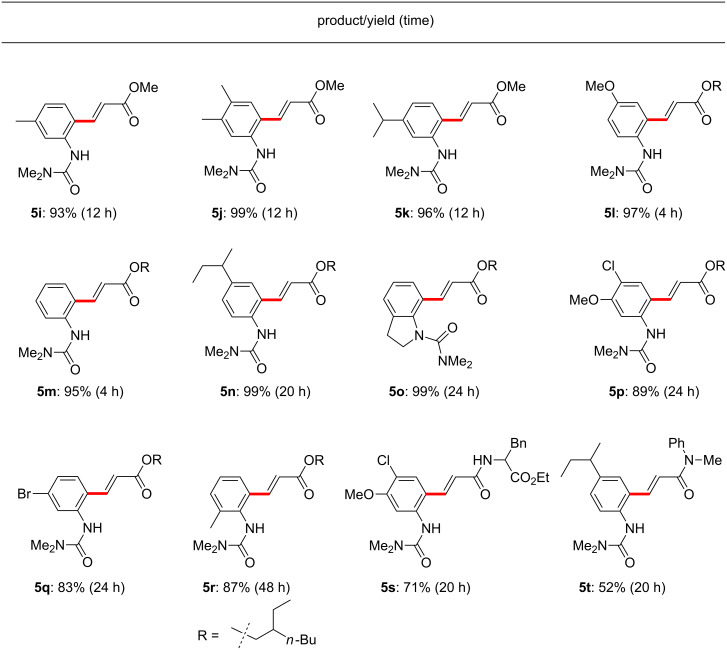
Representative results in EtOAc. Conducted at rt in EtOAc with 10 mol % Pd(OAc)_2_, HBF_4_ (1 equiv), arylurea (**1**, 0.25 mmol), BQ (3 equiv) and acrylate (**2**, 2.0 equiv).

Under optimized conditions, various acrylates and amides can be synthesized via C–H activation reactions (Figure 6). Methyl acrylate, which did not show good general reactivity with arylureas under previous conditions, could be coupled in excellent yields (**5i**, **5j**, **5k**). As previously mentioned, a drawback characteristic of several *ortho*-directed C–H activation cross-coupling approaches has been the undesired coupling at both sites *ortho-* to the directing group. These new conditions completely inhibited second-stage alkenylation, thereby generating singly derivatized arylureas in good yields (**5l**, **5m**, **5n**). Arylureas containing halogens, which are slightly electron-deficient but provide useful synthetic handles for subsequent functionalization, reacted cleanly to form the desired products (**5p**, **5q**). Arylureas bearing *ortho*-alkyl substituents also gave excellent yields (**5o**, **5r**), while acrylamides having simple amine or amino acid moieties also participated in cross-coupling reactions with the arylurea to produce the corresponding amide derivatives in moderate to good yields (**5s**, **5t**).

### Total synthesis of boscalid^®^ via C–H activation

**Scheme 6:**
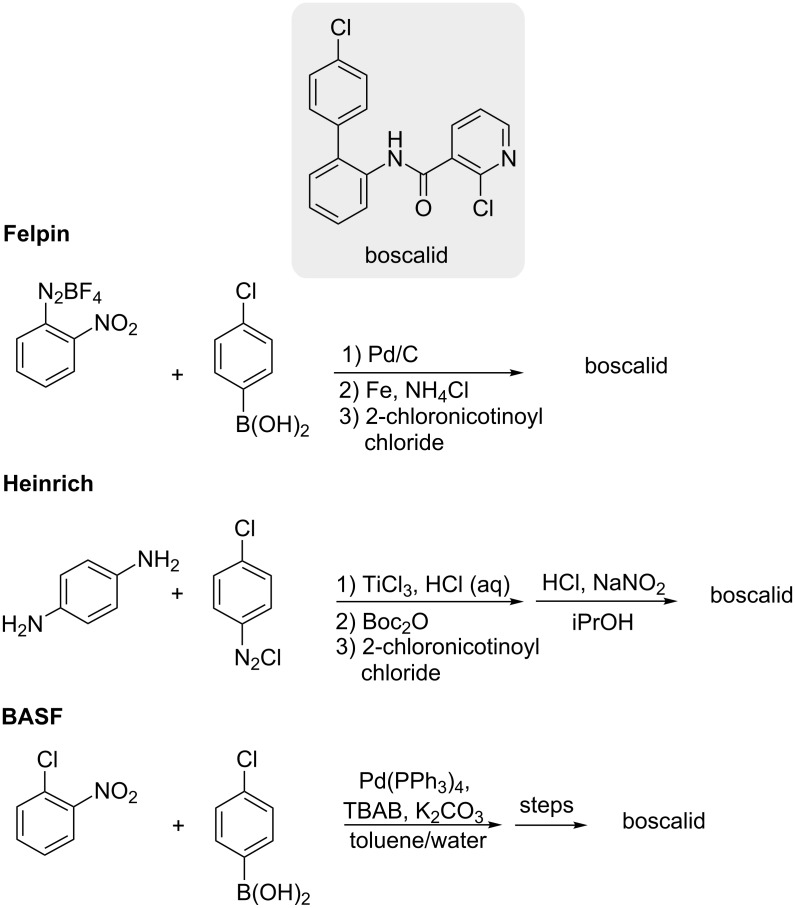
Previous syntheses of boscalid^®^.

The rationale behind the attention recently accorded C–H activation chemistry has been based, in part, on its potential to streamline routes towards valuable synthetic targets. As a demonstration of the utility of our C–H activation approach, we chose to synthesize boscalid^®^, a pesticide currently prepared on a yearly kiloton scale by means of a traditional Suzuki–Miyaura coupling. It is used to control a range of plant pathogens in broadacre and horticultural crops (Scheme 6) [207]. Felpin and co-workers have reported its synthesis starting from aryldiazonium salts [208], while the Heinrich group has employed a free-radical biaryl cross-coupling of diaminobenzene promoted by TiCl_3_ [209]. A number of additional syntheses can also be found in the literature [86,210]. Moreover, the BASF has patented routes using a traditional Suzuki–Miyaura cross-coupling in the presence of 0.5 mol % Pd catalyst to reach the same nitro-intermediate found in the Felpin route [211–213].

**Scheme 7:**
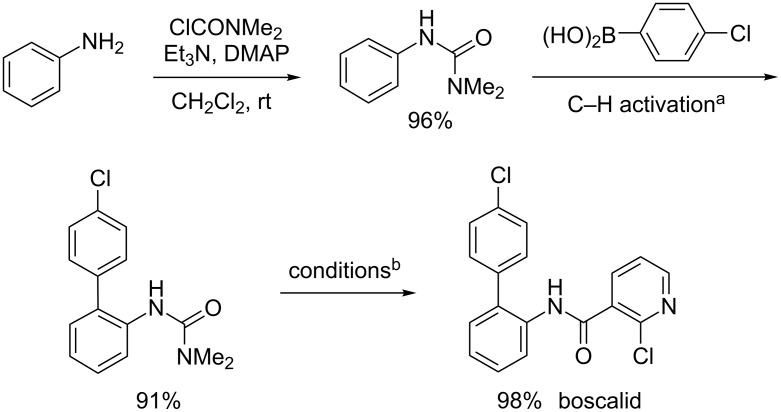
Synthesis of boscalid^®^. ^a^Conducted at rt for 20 h in EtOAc with 10 mol % [Pd(MeCN)_4_](BF_4_)_2_, BQ (5 equiv), arylurea (**1**, 0.25 mmol), and ArB(OH)_2_ (**2**, 1.5 equiv). ^b^NaOH, dioxane/water, reflux, then 2-chloronicotinoyl chloride, Et_3_N, THF, rt.

Many of these syntheses require large excesses of iron or other stoichiometric metals to obtain high yields (Scheme 6). As shown in Scheme 7, a synthesis that proceeds via a C–H activation strategy, however, might provide a highly efficient, alternative route originating from just aniline. The corresponding phenylurea can be prepared in high yield (96%), which is then subjected to C–H Suzuki–Miyaura coupling at room temperature (91%). Sequential deprotection and acylation with 2-chloronicotinoyl chloride result in boscalid in four steps in an overall yield of 86%, which compares favorably with all known routes to this pesticide shown in Scheme 6 [86,208–213].

### Mechanistic insight

**Scheme 8:**
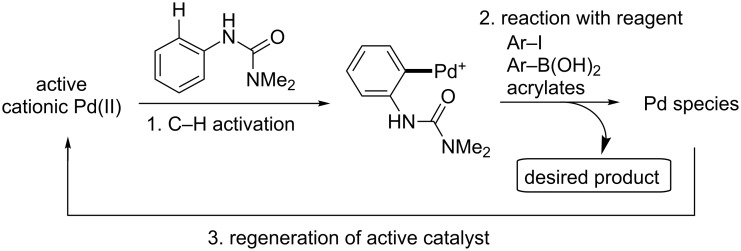
Hypothetical reaction sequence for cationic Pd(II)-catalyzed aromatic C–H activation reactions.

Although there have been a number of mechanistic studies on C–H activation reactions involving neutral palladium species [34,108–119], those catalyzed by cationic palladium have been much less thoroughly examined. We hypothesized that our catalytic cycles for the Fujiwara–Moritani, arylboronic acid, and aryl iodide coupling reactions catalyzed by cationic palladium complexes are composed of three key steps; (1) aromatic C–H activation by cationic palladium; (2) reaction of the resulting intermediate (a cationic palladacycle) with a corresponding reagent; and (3) re-generation of the active catalyst (Scheme 8). In order to test this hypothesis we explored the viability of each of these individual steps.

### The C–H activation step

Although aromatic C–H bond activation through palladacycle [214] generation is a critical step in the *ortho*-directed, activation/cross-coupling sequence, many of its specific mechanistic features are still controversial. Previous studies with arylureas [73,206,215] have formulated a palladacycle as the likely initial intermediate associated with palladation and subsequent C–H bond cleavage.

**Scheme 9:**
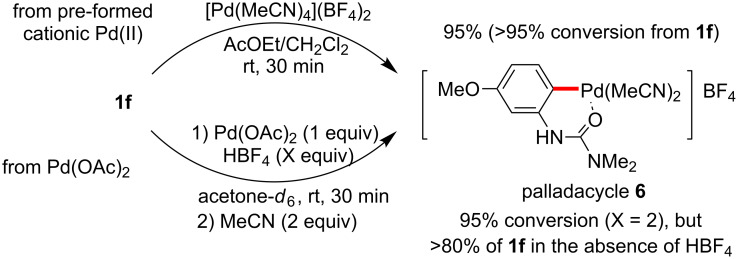
Palladacycle formation.

In order to confirm palladacycle formation in our reactions with arylureas, the dicationic palladium complex [Pd(MeCN)_4_](BF_4_)_2_ was exposed to one equivalent of 3-methoxyphenylurea **1f** at room temperature for 20 minutes (Scheme 9). This stoichiometric reaction led to the corresponding palladacycle **6** in 95% yield, without the aid of additives (i.e., no Ag salt or protic acid). In harmony, in situ-generated cationic palladium from the reaction of Pd(OAc)_2_ and HBF_4_ gave the same palladacycle upon addition of acetonitrile, as confirmed by NMR. The facile formation of this species supports the intermediacy of a palladacycle in the catalytic cycle. The structure of the isolated palladacycle was confirmed by X-ray analysis [216].

**Figure 7:**
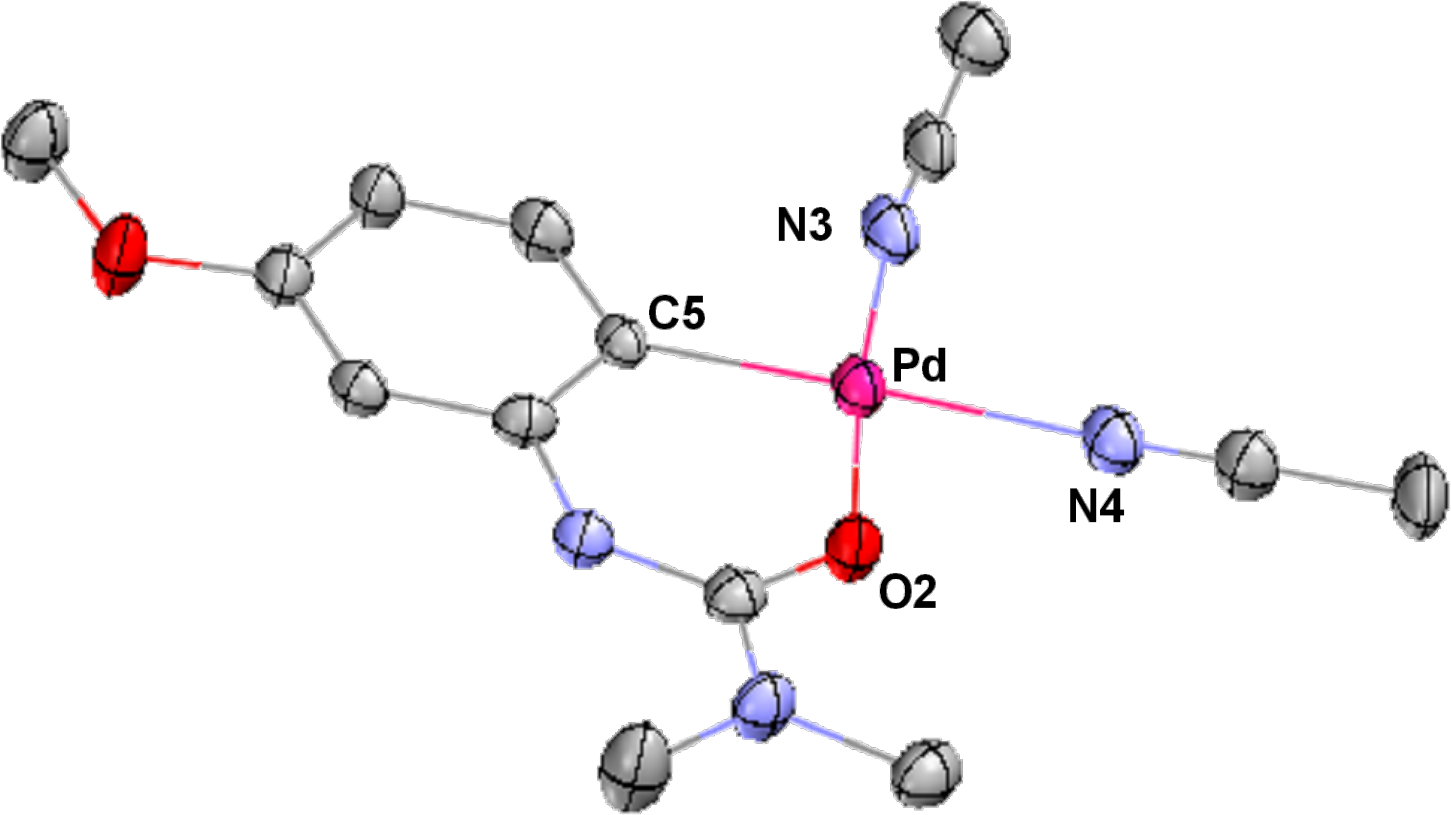
X-ray structure of palladacycle **6** with thermal ellipsoids at the 50% probability level. BF_4_ and hydrogen atoms were omitted for clarity. Selected bond length (Å): Pd–C(5) = 1.980, Pd–N(3) = 1.995, Pd–N(4) = 2.126, Pd–O(2) = 1.988. Selected angles (°): C(5)–Pd–N(3) = 94.15, N(3)–Pd–N(4) = 87.81, N(4)–Pd–O(2) = 86.07, O(2)–Pd–C(5) = 91.98.

An ORTEP plot for palladacycle **6** is shown in Figure 7. The molecular structure consists of a Pd atom with an arylurea and two molecules of acetonitrile assembled in a square-planar geometry around the metal. The sum of the angles around Pd is 360.01°. The C(5)–Pd–O(2) angle (91.98°) is slightly larger than that of N(3)–Pd–N(4) (87.81°), but it is similar to the angles of neutral PdCl_2_(Ph_2_PCH_2_CH_2_CH_2_PPh_2_)(dppp)) (angle of P–Pd–P: 90.58°) having a six-membered ring conformation [217], and palladacycles reported previously [73,206,215]. The length of the Pd–N4 bond, (2.126 Å), is slightly longer than those of Pd–N(3), Pd–C(5), Pd–O(2) bonds, likely due to a trans effect of the strong σ-donor aryl group as has been observed in a related urea palladacycle [73,215]. The bond length of Pd–N is 1.96 Å in [Pd(MeCN)_4_](BF_4_)_2_.

**Figure 8:**
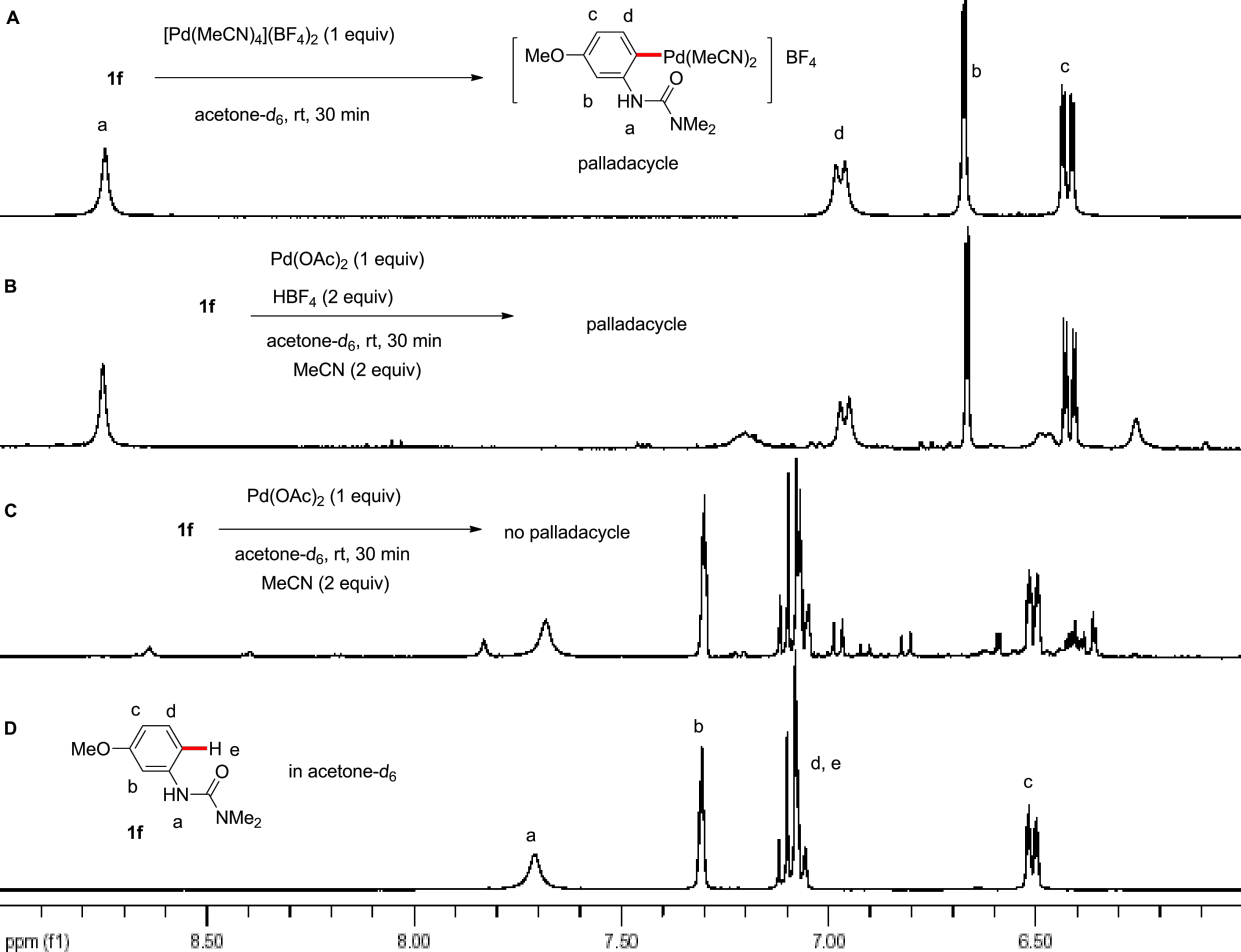
NMR studies. A: The reaction of [Pd(MeCN)_4_](BF_4_)_2_ and 3-MeOC_6_H_4_NHCONMe_2_ in acetone-*d*_6_. B: The reaction of Pd(OAc)_2_ and 3-MeOC_6_H_4_NHCONMe_2_ in the presence of HBF_4_ and MeCN in acetone-*d*_6_. C: The reaction of Pd(OAc)_2_ and 3-MeOC_6_H_4_NHCONMe_2_ in the presence of MeCN in acetone-*d*_6_. D: 3-MeOC_6_H_4_NHCONMe_2_ in acetone-*d*_6_.

**Scheme 10:**
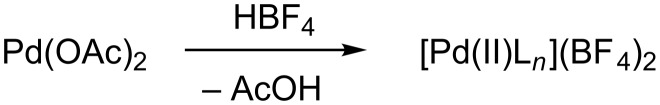
The generation of cationic Pd(II) from Pd(OAc)_2_.

NMR spectroscopic studies on the reaction between a cationic Pd(II) complex and an arylurea to generate a palladacycle are illustrated in Figure 8. The pure palladacycle from pre-formed cationic palladium [Pd(MeCN)_4_](BF_4_)_2_ is shown as spectrum in Figure 8A. Generally, monocationic arylpalladium(II) complexes without strongly coordinating ligands are unstable even at low temperatures [218–220]; nonetheless, this cationic palladacycle, aided by the presence of strongly coordinating MeCN, was found to be quite stable at room temperature. While the in situ generated cationic palladium species from the reaction of Pd(OAc)_2_ and HBF_4_ gave the same palladacycle upon treatment with the arylurea (spectrum Figure 8B), the reaction in the absence of HBF_4_ did not result in palladacycle formation (spectrum Figure 8C). Here, essentially no conversion of the starting material was detected by ^1^H NMR in acetone-*d*_6_ (spectrum Figure 8D). Indeed, for reactions starting from Pd(OAc)_2_, no cross-coupling product was observed without adding a BF_4_^−^ source for the Fujiwara–Moritani reaction (Figure 5, run 9), Suzuki–Miyaura coupling (Scheme 4), and arylation with aryl iodide [121–122,150]. HBF_4_ apparently acts as an acetate scavenger to generate the active cationic palladium(II) species (Scheme 10).

**Scheme 11:**
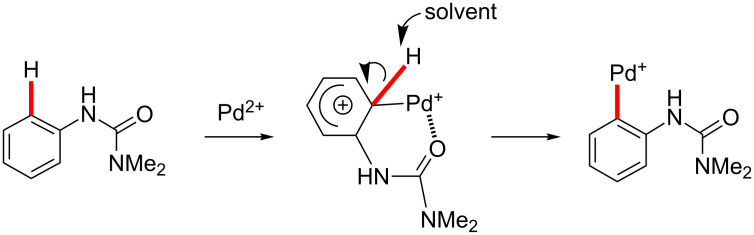
Electrophilic substitution of aromatic hydrogen by cationic palladium(II) species.

As discussed previously herein, there are several routes available for cyclopalladation and C–H bond cleavage, most notably the concerted metalation-deprotection (CMD) or electrophilic palladation pathways (Scheme 1) [221–224]. Although control experiments had previously indicated the importance of conditions involving cationic palladium for achieving overall reaction conversion, our studies of palladacycle formation suggest that a cationic palladium catalyst is specifically required for the initial C–H activation step itself. Since the crystal structure of **6** (Figure 7) is indicative of a monocationic palladacycle, the cationicity of the metal may still play a role as well in subsequent steps. However, the much higher reactivity of a cationic Pd species (even under acetate-free conditions), the lack of effectiveness of Pd(OAc)_2_ alone in palladacycle formation, and the observed reactivity trends that strongly favor more electron-rich arylureas, all appear to be most consistent with an electrophilic palladation pathway over a CMD mechanism (Scheme 11). However, it is appreciated that further study might provide additional insight on this point.

### Reactions of palladacycle **6** with Ph–I, PhB(OH)_2_, and an acrylate

**Scheme 12:**
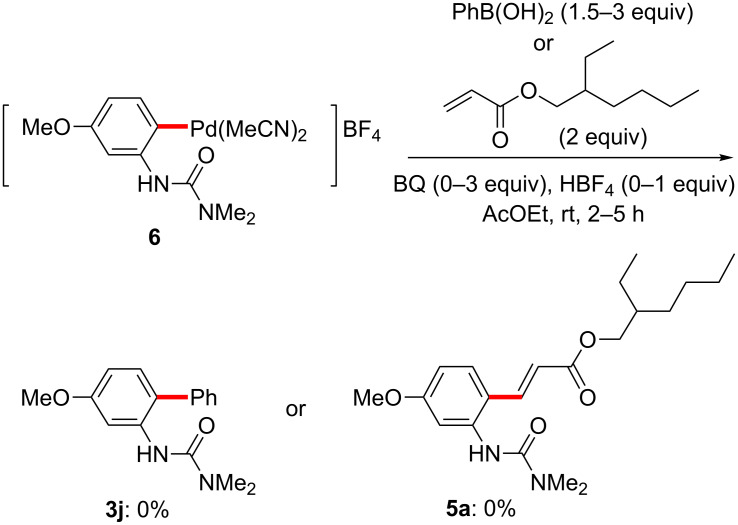
Attempted reactions of palladacycle **6**.

Having demonstrated the potential for facile palladacycle formation at room temperature, we next examined the reactivity of this intermediate with coupling partners for each of the three reaction types studied. Stoichiometric reactions between the isolated palladacycle **6** and an acrylate or arylboronic acid were first attempted at room temperature (Scheme 12). Initial experiments, however, resulted in no formation of the desired products. Although the palladacycles were subjected to various conditions in the presence of BQ and HBF_4_, the anticipated reaction did not proceed from isolated catalyst complexes containing the stabilizing ligand MeCN.

**Scheme 13:**
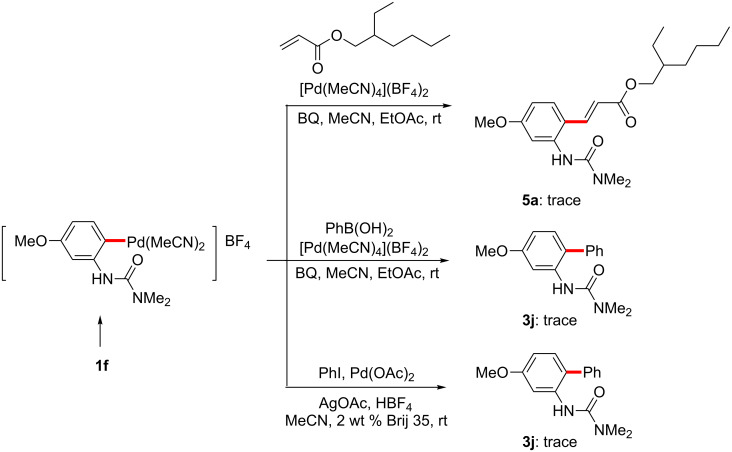
The impact of MeCN on C-H activation/coupling reactions.

Although the initial C–H activation step proceeded readily in the presence of MeCN in these stoichiometric reactions, subsequent reactions of the palladacycle with acrylates, arylboronic acids, and aryl iodides appeared to be significantly suppressed by the presence of stoichiometric MeCN. The inhibitory effect of this ligand had been previously observed in the coupling reactions of aryl iodides (in which even 40 mol % of MeCN was enough to almost completely shut down the reaction) [121]. In a cationic palladium(II) complex-catalyzed 1,4-addition of arylsilane, the nitrile-free cationic Pd(II) catalyst was much more effective than a PhCN-containing cationic palladium(II) complex towards transmetallations with arylsilicon-containing partners and insertion of mono-cationic arylpalladium(II) species into olefins [136]. The detrimental effect of MeCN under our C–H activation conditions was further established through a series of reactions as illustrated in Scheme 13. Under optimized conditions previously determined, where C–H functionalized products were obtained in good yields, in the presence of added MeCN (1 equiv relative to **1f**) all three reactions were completely inhibited, in all likelihood due to its strong coordinating ability as a ligand on cationic palladium.

**Scheme 14:**
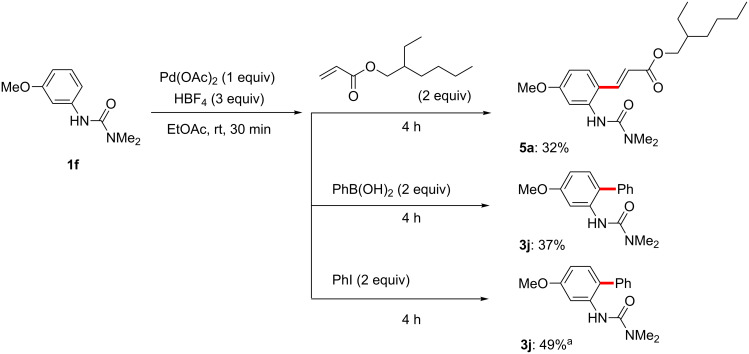
Stoichiometric MeCN-free reactions. ^a^2% Brij 35 was used instead of EtOAc.

On the other hand, when nitrile-free conditions were applied to urea **1f**, with in situ-generated palladacycle (from Pd(OAc)_2_ and HBF_4_; Figure 8), followed by addition of the usual reagents, each reaction proceeded to give the anticipated acrylated/arylated product (Scheme 14; unoptimized yields). Notably, all three stoichiometric reactions now proceeded in the absence of other additives, such as BQ or Ag(I) salts, which are required for the catalytic versions to proceed efficiently.

**Scheme 15:**
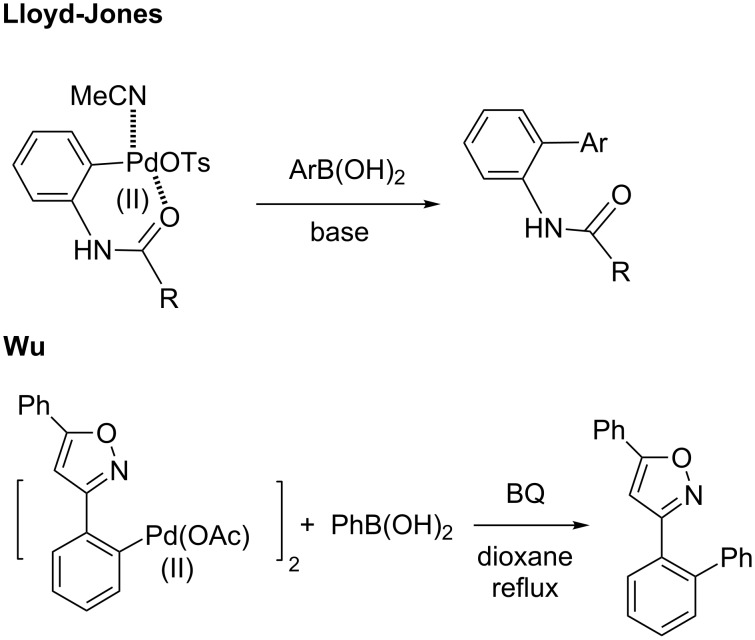
The reactions of divalent palladacycles.

Although Lloyd-Jones and Booker-Milburn also reported the reaction of a urea-derived palladacycle and arylboronic acid in the presence of base in THF under reflux conditions to produce the corresponding coupling product, our cationic palladacycle underwent coupling without added base (Scheme 15) [178]. In fact, it has been previously shown that cationic palladium species can undergo transmetalation with an arylboronic acid in the absence of base even at 0 °C [219–220]. Wu and co-workers have also reported the interesting reactivities of neutral palladacycles with arylboronic acids (Scheme 15). Under their conditions, BQ and high temperature were critical to obtain the product from their 5-membered isoxazoline-containing palladacycle [160,168,225]. Although BQ is sometimes used as a ligand for palladium to accelerate reductive elimination [103,226–230], its presence was not necessary in our stoichiometric reaction of a cationic 6-membered ring palladacycle.

### Regeneration of active catalyst; the roles of additives BQ, AgOAc, and HBF_4_

**Scheme 16:**
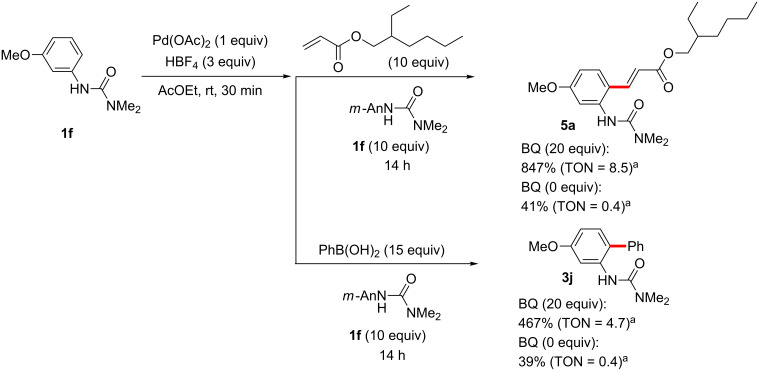
Role of BQ in stoichiometric Fujiwara–Moritani and Suzuki–Miyaura coupling reactions. ^a^Yields based on Pd.

As shown in previous sections herein, both the formation of palladacycles from arylureas and their subsequent coupling reactions with acrylates, arylboronic acids, and aryl iodides proceed under stoichiometric palladium conditions in the absence of additives, such as BQ and AgOAc, which had been necessary in the corresponding optimized catalytic reactions. To establish the roles of these additives, the reaction of the palladacycle in the presence of excesses of both coupling partners was carried out (Scheme 16). In Fujiwara–Moritani and Suzuki–Miyaura coupling reactions, 41 and 39% of the products (isolated yields based on the palladacycle) were obtained, respectively, in the absence of BQ, indicating that no catalyst turnover was occurring without this additive. When 20 equivalents of BQ were added, along with the coupling partners, however, 847% (TON = 8.5) and 467% (TON = 4.7) yields of the products were obtained, respectively, supporting a key role for BQ in regeneration of the active dicationic species (PdL_4_(BF_4_)_2_).

**Scheme 17:**
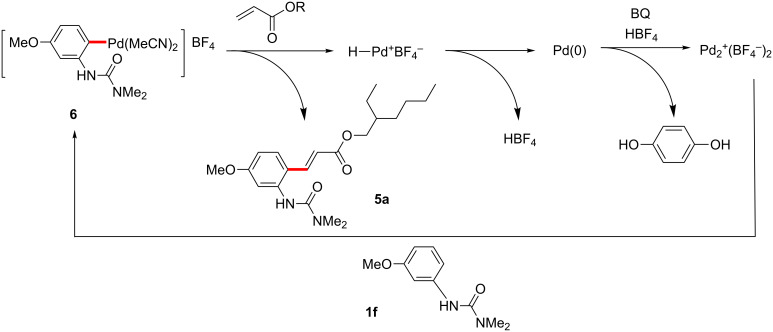
Proposed role of BQ in Fujiwara–Moritani reactions.

**Scheme 18:**
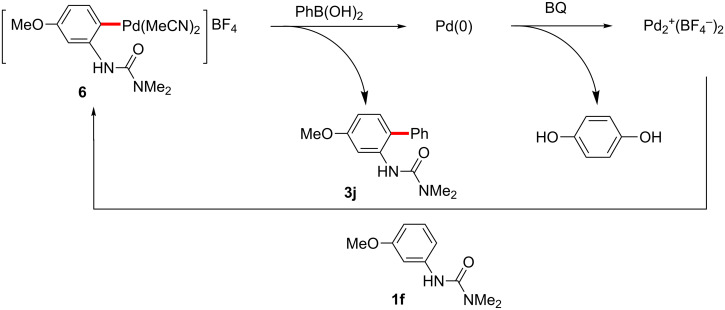
Proposed role of BQ in Suzuki–Miyaura coupling reactions.

Benzoquinone (BQ) has been well studied as an oxidant for Pd(0) to Pd(II) processes, generating hydroquinone as a byproduct. For the Fujiwara–Moritani coupling, addition of the palladacycle **6** to an acrylate followed by β-hydride elimination and reductive elimination of HPd^+^BF_4_^−^ would result in a Pd(0) species unable to participate in palladacycle formation until it is oxidized by BQ to Pd^2+^(BF_4_)_2_, whereupon it reacts with another equivalent of arylurea (Scheme 17) [231]. Similarly, BQ’s role in C–H coupling of boronic acids would likely be to oxidize Pd(0) to Pd(II) after the product forming step (Scheme 18). Transmetallation between the palladacycle and arylboronic acid followed by reductive elimination would give the expected product and Pd(0), where the metal can be subsequently oxidized with BQ.

**Scheme 19:**
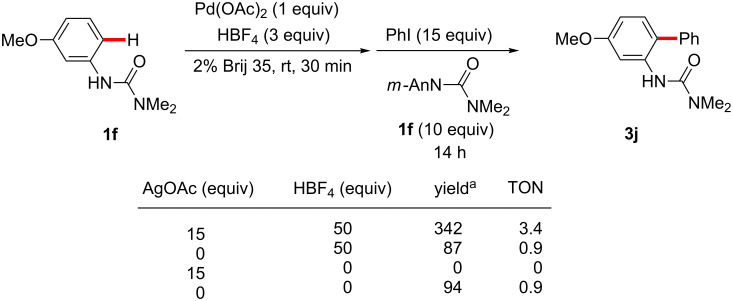
Stoichiometric C–H arylation of iodobenzene. ^a^Yields based on Pd.

**Scheme 20:**
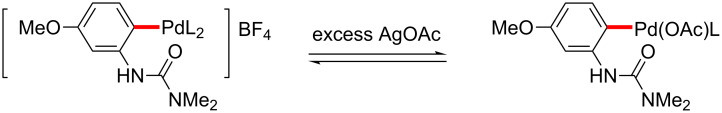
Impact of acetate on the cationicity of Pd.

In the reaction with aryl iodides, when an excess of coupling partner was employed, a yield of ca. 90% (TON = 0.9) of the product was obtained with or without additional HBF_4_ (Scheme 19), in this case indicating that no catalyst turnover was occurring in the absence of a Ag(I) salt. Surprisingly, when 15 equivalents of AgOAc were added along with an excess of both coupling partners, but without the addition of HBF_4_ beyond the three equivalents required for initial palladacycle formation, no coupling product was observed. Under these conditions, a large excess of acetate anion relative to BF_4_^−^ would exist, which may decrease the cationicity of the cationic palladium(II) species formed in the initial cyclopalladation (Scheme 20), or otherwise disrupt the reaction sequence subsequent to palladacycle formation. On the other hand, the reaction in the presence of both silver salt and excess of HBF_4_ gave the corresponding product in 342% yield relative to palladium (TON = 3.4) (Scheme 19).

**Scheme 21:**
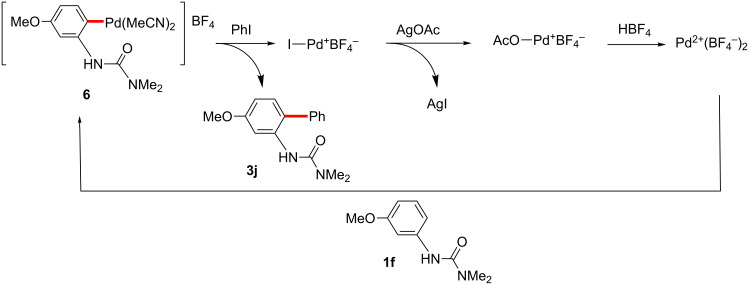
Roles of additives in C–H arylation.

Based on these results, the proposed roles of silver and acid are shown in Scheme 21. After the coupling of the palladacycle and aryl iodide, I–Pd^+^BF_4_^−^ is generated, which is catalytically inactive. Then, I–Pd^+^BF_4_^−^ reacts with AgOAc and HBF_4_ to regenerate active cationic Pd^2+^(BF_4_^−^)_2_. Under this proposed sequence, AgOAc would primarily act, therefore, as an iodide scavenger.

**Scheme 22:**
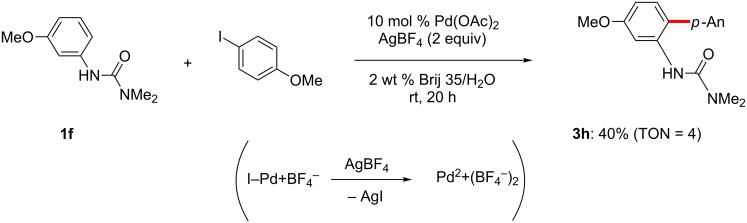
Cross-coupling in the presence of AgBF_4_.

In our previous studies it was found that when AgBF_4_ was used in place of AgOAc under optimized conditions for C–H arylation with aryl iodides, HBF_4_ (or any other added acid) was unnecessary for the catalytic reaction to occur. Here, since AgBF_4_ apparently reacts with I–Pd^+^BF_4_^−^ to produce catalytically active Pd^2+^(BF_4_)_2_^−^ (Scheme 22), and there are no stoichiometric quantities of competing acetate anions, additional acid is not needed to produce and maintain active catalyst.

### Proposed mechanisms

Our results have demonstrated that a dicationic palladium complex effectively catalyzes C–H activation of arylureas at room temperature. Based on these studies of the major steps for each reaction, proposed catalytic cycles are illustrated below.

**Scheme 23:**
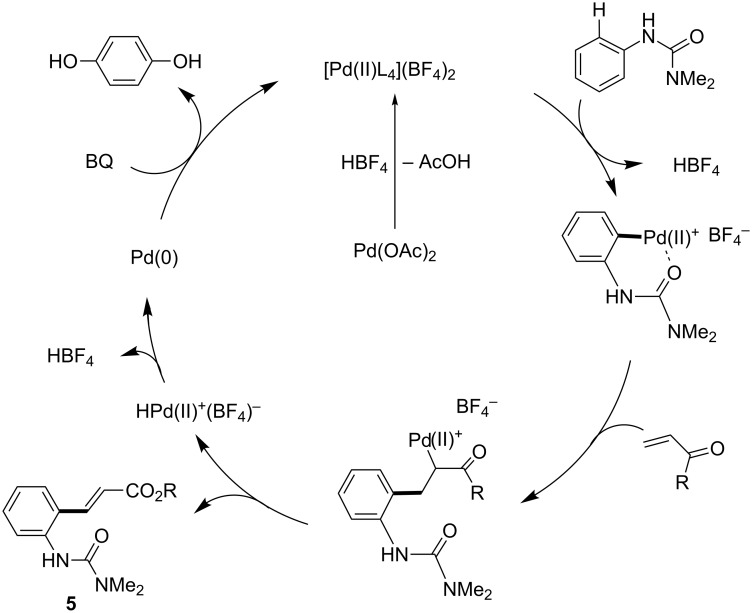
A proposed catalytic cycle for Fujiwara–Moritani reactions.

**Fujiwara–Moritani reactions**. At the first stage of the catalytic cycle, an active divalent cationic palladium species is generated from the reaction of a neutral complex, Pd(OAc)_2_, and HBF_4_ [232]. This reaction results in the formation of a monocationic arylpalladium(II) palladacycle, likely via a Wheland-like intermediate (Scheme 23) [233–237]. The product-forming portion of the cycle may proceed in a manner resembling a traditional Heck cross-coupling. In this case, addition of the palladacycle to an acrylate followed by β-hydride elimination yields the corresponding product **5**. As previously demonstrated in palladium-catalyzed Mizoroki–Heck reactions [238–240], insertion of a alkenyl double bond into C–Pd^+^ present within the cationic palladacyle is facile, owing to the high Lewis acidity of the metal center. This is a noteworthy advantage associated with the use of cationic palladium(II) catalysts. Finally, BQ oxidizes the Pd(0) that is reductively eliminated from the HPd(II)^+^BF_4_^−^ formed to regenerate the active cationic palladium species Pd^2+^(BF_4_)_2_^−^.

**Scheme 24:**
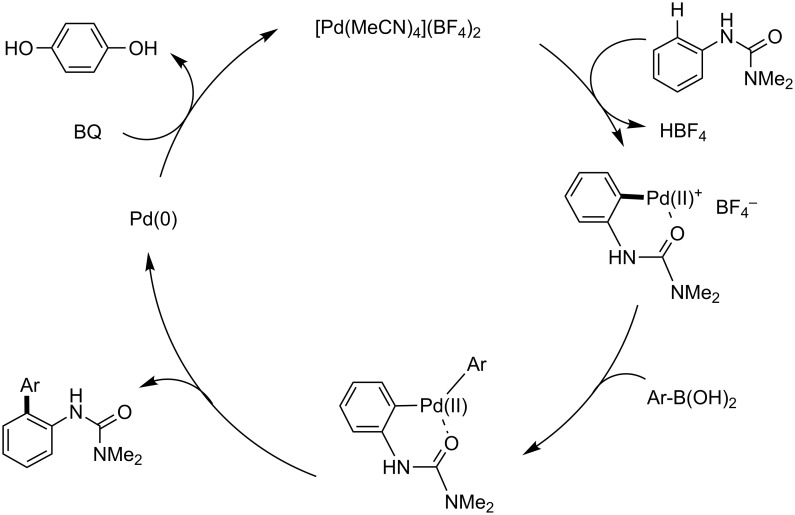
Proposed catalytic cycle of C–H activation/Suzuki–Miyaura coupling reactions.

**C–H boronic acid coupling reactions**. Unlike traditional Suzuki–Miyaura coupling reactions [241–243], C–H coupling reactions catalyzed by a cationic palladium(II) complex require an oxidant instead of a strong base, but otherwise likely share a number of features with this widely used C–C bond-forming process. The reaction also presumably starts from the generation of a cationic palladacycle, which may undergo a facile transmetalation with an arylboronic acid without prior activation by base (Scheme 24) [241–248]. This step is followed by reductive elimination of a diarylpalladium(II) species, affording the coupling product and Pd(0). The resulting Pd(0) is then oxidized with BQ to regenerate the dicationic palladium species, which can re-enter the catalytic cycle.

**Scheme 25:**
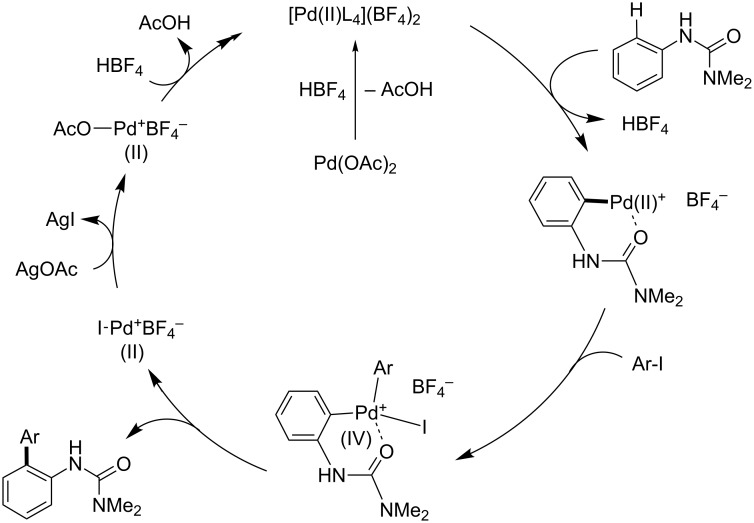
A proposed catalytic cycle for C–H arylation involving a Pd(IV) intermediate.

**Arylation with an aryl iodide**. Coupling reactions of aryl iodides have fewer similarities in terms of traditional cross-coupling reactions compared with features characteristic of other room temperature C–H activations we have studied, and are more difficult to rationalize with a Pd(II)/Pd(0) catalytic cycle. Hence, a possible Pd(II)/Pd(IV) catalytic cycle, similar to that previously proposed by Daugulis [8], is proposed below (Scheme 25). A mono-cationic palladium intermediate reacts with the aryl iodide, albeit in a poorly understood step of the sequence. Stoichiometric studies reveal that this step occurs in the absence of AgOAc, and in fact its presence in excess relative to HBF_4_ inhibits the reaction (Scheme 19). In one possible pathway, an oxidative addition to the aryl iodide would provide a Pd(IV) intermediate, which could then rapidly reductively eliminate in the C–C bond forming step. The resulting I–Pd(II)–X species could then be converted back to the active cationic palladium species through reactions with the silver salt and HBF_4_ (or in situ generated AgBF_4_).

**Scheme 26:**
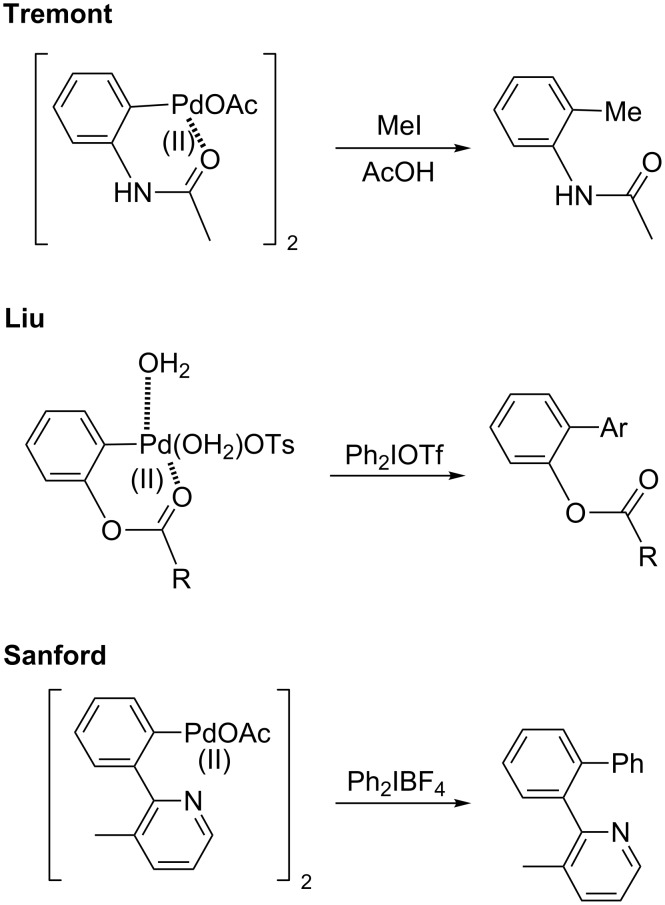
Selected reactions of divalent palladacycles.

The specific nature of the reaction between the palladacycle and aryl iodide and resulting intermediate is lacking in details. It is known that divalent palladacycles react with alkyl iodides or diaryliodonium salts, and this process likely involves a Pd(IV) intermediate. Tremont and co-workers previously proposed a Pd(IV) intermediate in C–H alkylation of acetoanilides and alkyl iodides [172]. In this case the reaction of a divalent palladacycle and MeI readily occurred at room temperature and was shown not to proceed through a radical pathway. Notably, in 2011 Vicente and co-workers obtained the crystal structure of a Pd(IV) complex obtained by the room temperature oxidative addition of an internally-chelated Pd(II) species into an aryl iodide, and demonstrated this species’ competence as a pre-catalyst for C–H olefinations. Liu [73] and Sanford reported the stoichiometric reaction of palladacycles and aryliodonium salts at high temperature to give the corresponding products through Pd(II)/Pd(IV) or Pd(III)/Pd(III) dimeric species bridged by acetates (Scheme 26) [68,175,249–250]. Sequences involving Pd(III) intermediates have been suggested as alternatives to Pd(II)/Pd(IV) cycles in some cases, but most well-studied examples involve Pd(III)/Pd(III) dimers formed with the aid of bridging anionic ligands such as acetate or nitrate [251–252] that do not match as well with the cationic palladium conditions employed here. Silver-mediated one electron oxidations to form monomeric Pd(III) complexes have also been studied [251], but the successful implementation of silver-free conditions with stoichiometric palladium herein would appear to eliminate this as a key step. To the best of our knowledge, the existence of Pd(IV) complexes has yet to be conclusively demonstrated from the oxidative addition of anilide-derived, divalent palladacycles into aryl halides, although formation of octahedral Pd(IV) complexes from *N*-substituted biphenyl palladacycles that possess similar highly planar structures as found in the urea-derived palladacycle (Figure 7) have been well studied [253–255].

## Conclusion

In conclusion, we have demonstrated that a dicationic palladium complex enables facile C–H activation reactions of arylureas with aryl iodides, arylboronic acids, and acrylates at room temperature. In many cases nanomicelles in water can be used in place of organic solvents, allowing for some of the mildest and environmentally responsible conditions yet achieved in C–H activation chemistry. The practical value of this approach has been further demonstrated with an efficient, streamlined application to the synthesis of the herbicide boscalid. Mechanistic investigations revealed that a dicationic Pd(II) complex reacts readily with an arylurea to rapidly produce a mono-cationic palladacycle at room temperature, and this likely, catalytically competent species has been characterized by X-ray crystallography. Experiments revealed that a highly cationic palladium complex is required for the formation of this palladacycle at room temperature. While some key steps, including the precise nature of the reaction between this cationic palladacycle and aryl iodides, require further clarification, studies using stoichiometric palladium have provided insight into the roles of additives HBF_4_, BQ, and AgOAc, as well as the crucial steps of each reaction’s catalytic cycle. Lastly, this study highlights the advantages of dicationic palladium complexes in synthesis, and their tolerance to aqueous media. Such cationic reagent/medium combinations, potentially applicable to other group 10 metals, and Ni in particular, may well offer related synthetic opportunities.

## Supporting Information

File 1 Experimental procedures and characterization of all new compounds.

File 2 Crystal structure of **6**.

File 3 Crystal structure of **6** No 2.
