# Supplementary material for: Cationic Pd(II)-catalyzed C–H activation/cross-coupling reactions at room temperature: synthetic and mechanistic studies
Source: Beilstein J Org Chem. 2016 May 20;12:1040–64. doi: 10.3762/bjoc.12.99 (PMC4902085; doi:10.3762/bjoc.12.99)

Supporting Information

for

Cationic Pd(II)-catalyzed C–H activation/cross-coupling reactions at room temperature: synthetic and mechanistic studies

Takashi Nishikata, Alexander R. Abela, Shenlin Huang and Bruce H. Lipshutz*

Address: Department of Chemistry & Biochemistry, University of California, Santa Barbara, CA 93106, USA

Email: lipshutz@chem.ucsb.edu

* Corresponding author

Experimental procedures and characterization of all new compounds

I.	Synthesis of arylurea	S2–S7
II.	C–H arylations with aryl iodides	S7–S8
III.	C–H Suzuki–Miyaura	S8–S15
IV.	Fujiwara–Moritani	S15–S22
V.	Synthesis of boscalid	S23
VI.	Mechanistic studies	S24–S26
VII.	References	S26
VIII.	Spectral data	S27–S105

General information

For TLC analyses precoated Kieselgel 60 F_{254} plates (Merck, 0.25 mm thick) were used; for column chromatography Silica Flash® P60 (SiliCycle, 40–63 µm) was used. Reactions were monitored using an Hewlett-Packard HP6890 gas chromatograph. ^{1}H and ^{13}C NMR spectra were obtained using a Varian UNITY INOVA 400 MHz NMR spectrometer. High resolution mass spectral analyses were obtained using a VG70 double-focusing magnetic sector instrument (VG Analytical) for EI and a PE Sciex QStar Pulsar quadrupole/TOF instrument (API) for ESI. *Compounds **1a**,**1b**, **1d**, **1f**, **1j**, **1k**, **1l**, **1n**, **3a**–**3hh**, **5a**–**5c** were previously reported[1-5].

I. The synthesis of arylureas

Table 1. Starting materials.

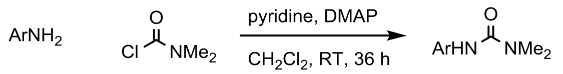

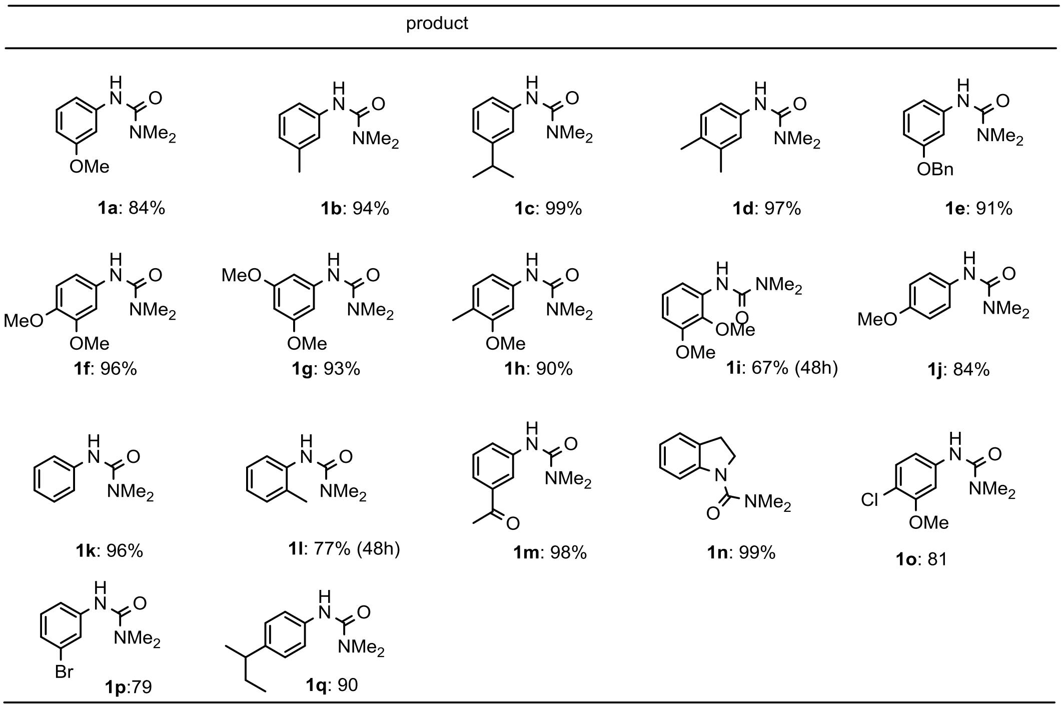

General procedure A [similar as described in ref. [3]].

Anilines (1 mmol), *N*,*N*-dimethylcarbamoyl chloride (2 mmol), DMAP (1 mmol), and pyridine (4 mmol) were sequentially added under air to a reaction tube equipped with a stir bar and a septum. CH_2Cl_2 (2 mL) was added by syringe and the resulting mixture vigorously stirred for 36–48 h at ambient temperature. After this time, the contents of the flask were extracted with

EtOAc. The solution obtained was filtered through the plug of silica gel and anhydrous $MgSO_4$, and then concentrated by rotary evaporation. The residue was purified by flash chromatography, eluting with hexane/EtOAc to afford the product.

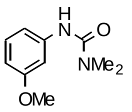

Following the general procedure **A**, using aniline (0.11 mL, 1 mmol), DMAP (122 mg, 1 mmol), pyridine (0.32 mL, 4 mmol), *N*,*N*-dimethylcarbamoyl chloride (0.18 mL, 2 mmol), and CH_2Cl_2 (2 mL), yielded the product **1a** (164 mg, 84%); ^{1}H NMR ($CDCl_3$) δ: 3.02 (s, 6H), 3.79 (s, 3H), 6.36 (brs, 1H), 6.57 (d, *J* = 8.0 Hz, 1H), 6.83 (d, *J* = 7.5 Hz, 1H), 7.14-7.19 (m, 2H); ^{13}C NMR ($CDCl_3$), δ: 36.66, 55.46, 105.39, 109.22, 111.94, 129.63, 140.72, 155.80, 160.40; HRESIMS calcd. for $C_{10}H_{14}N_2O_2Na$ ($M+Na^+$): 217.0953; found 217.0948.

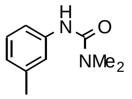

Following the general procedure **A**, using *m*-toluidine (1.07 mL, 10 mmol), DMAP (1.22 g, 10 mmol), pyridine (3.24 mL, 40 mmol), *N*,*N*-dimethylcarbamoyl chloride (1.84 mL, 20 mmol), and CH_2Cl_2 (20 mL), yielded the product **1b** (1.68 g, 94%); ^{1}H NMR ($CDCl_3$) δ: 2.32 (s, 3H), 3.02 (s, 6H) , 6.26 (brs, 1H), 6.83-6.85 (m, 1H), 7.14-7.26 (m, 3H); ^{13}C NMR ($CDCl_3$), δ: 21.57, 36.50, 117.12, 120.80, 123.76, 128.65, 138.67, 139.33, 156.01; HRESIMS calcd. for $C_{10}H_{14}N_2ONa$ ($M+Na^+$): 201.1004; found 201.0999.

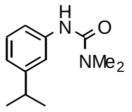

Following the general procedure **A**, using aniline (0.14 mL, 1 mmol), DMAP (122 mg, 1 mmol), pyridine (0.32 mL, 4 mmol), *N*,*N*-dimethylcarbamoyl chloride (0.18 mL, 2 mmol), and CH_2Cl_2 (2 mL), yielded the product **1c** (204 mg, 99%); ^{1}H NMR ($CDCl_3$) δ: 1.24 (d, *J* = 7.2 Hz, 6H), 2.87 (sept, *J* = 7.2 Hz, 1H), 3.04 (s, 6H) , 6.26 (brs, 1H), 6.89-6.92 (m, 1H), 7.16-7.26 (m, 2H), 7.29 (s, 1H); ^{13}C NMR ($CDCl_3$), δ: 24.12, 34.34, 36.64, 117.53, 118.20, 121.28, 128.89, 139.32, 149.93, 155.99; HRESIMS calcd. for $C_{12}H_{18}N_2ONa$ ($M+Na^+$): 229.1317; found 229.1314.

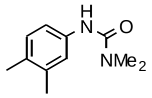

Following the general procedure **A**, using aniline (121 mg, 1 mmol), DMAP (122 mg, 1 mmol), pyridine (0.32 mL, 4 mmol), *N,N*-dimethylcarbamoyl chloride (0.18 mL, 2 mmol), and CH_2Cl_2 (2 mL), yielded the product **1d** (187 mg, 97%); ^{1}H NMR ($CDCl_3$) δ: 2.20 (s, 3H), 2.23 (s, 3H), 3.02 (s, 6H), 6.18 (brs, 1H), 7.03 (d, J = 8.4 Hz, 1H), 7.07 (dd, J = 2.0 and 8.4 Hz, 1H), 7.20 (d, J = 1.6 Hz, 1H); ^{13}C NMR ($CDCl_3$), δ: 19.24, 20.08, 36.64, 117.65, 121.70, 130.02, 131.40,137.04, 137.19, 156.12; HRESIMS calcd. for $C_{11}H_{16}N_2ONa$ (M+Na$^+$): 215.1160; found 215.1157.

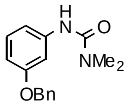

Following the general procedure **A**, using aniline (199 mg, 1 mmol), DMAP (122 mg, 1 mmol), pyridine (0.32 mL, 4 mmol), *N,N*-dimethylcarbamoyl chloride (0.18 mL, 2 mmol), and CH_2Cl_2 (2 mL), yielded the product **1e** (244 mg, 91%); ^{1}H NMR ($CDCl_3$) δ: 3.04 (s, 6H), 5.06 (s, 2H), 6.29 (brs, 1H), 6.66 (dd, J = 2.4 and 8.4 Hz, 1H), 6.84 (d, J = 7.2 Hz, 1H), 7.17 (t, J = 8.0 Hz, 1H), 7.28-7.44 (m, 6H); ^{13}C NMR ($CDCl_3$), δ: 36.63, 70.10, 106.38, 109.97, 112.27, 127.67, 128.02, 128.68, 129.64, 137.28, 140.73, 155.79, 159.58; HRESIMS calcd. for $C_{16}H_{18}N_2O_2Na$ (M+Na$^+$): 293.1266; found 293.1269.

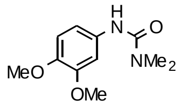

Following the general procedure **A**, using aniline (153 mg, 1 mmol), DMAP (122 mg, 1 mmol), pyridine (0.32 mL, 4 mmol), *N,N*-dimethylcarbamoyl chloride (0.18 mL, 2 mmol), and CH_2Cl_2 (2 mL), yielded the product **1f** (216 mg, 96%); ^{1}H NMR ($CDCl_3$) δ: 3.03 (s, 6H), 3.85 (s, 3H), 3.88 (s, 3H), 6.20 (brs, 1H), 6.69 (dd, J = 2.8 and 8.8 Hz, 1H), 6.78 (d, J = 8.8 Hz, 1H), 7.26 (d, J = 2.8 Hz, 1H); ^{13}C NMR ($CDCl_3$), δ: 36.54, 55.93, 56.29, 105.67, 111.54, 112.09, 133.11, 145.16, 149.13, 156.28; HRESIMS calcd. for $C_{11}H_{16}N_2O_3Na$ (M+Na$^+$): 247.1059; found 247.1057.

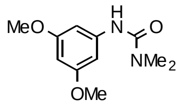

Following the general procedure **A**, using aniline (153 mg, 1 mmol), DMAP (122 mg, 1 mmol), pyridine (0.32 mL, 4 mmol), *N,N*-dimethylcarbamoyl chloride (0.18 mL, 2 mmol), and CH_2Cl_2 (2 mL), yielded the product **1g** (209 mg, 93%); ^{1}H NMR ($CDCl_3$) δ: 3.02 (s, 6H), 3.76 (s, 6H), 6.16 (s, 1H), 6.31 (brs, 1H), 6.65 (s, 2H); ^{13}C NMR ($CDCl_3$), δ: 36.48, 55.33, 95.51, 97.95, 141.39, 155.81, 160.97; HRESIMS calcd. for $C_{11}H_{16}N_2O_3Na$ (M+Na$^+$): 247.1059; found 247.1058.

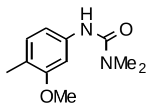

Following the general procedure **A**, using aniline (250 mg, 1.8 mmol), DMAP (1.8 mmol, 223 mg), pyridine (7.3 mmol, 0.6 mL), *N,N*-dimethylcarbamoyl chloride (3.6 mmol, 0.34 mL), and CH_2Cl_2 (3.6 mL), yielded the product **1h** (344 mg, 90%); ^{1}H NMR ($CDCl_3$) δ: 2.15 (s, 3H), 3.03 (s, 6H), 3.83 (s, 3H), 6.26 (brs, 1H), 6.60 (dd, *J* = 2 and 8 Hz, 1H), 6.99 (d, *J* = 8 Hz, 1H), 7.28 (s, 1H); ^{13}C NMR ($CDCl_3$), δ: 15.68, 36.43, 55.25, 103.17, 111.44, 120.84, 130.15, 138.46, 156.10, 157.84; HRESIMS calcd. for $C_{11}H_{16}N_2O_2Na$ ($M+Na^+$): 231.1109; found 231.1107.

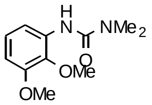

Following the general procedure **A**, using aniline (0.14 mL, 1 mmol), DMAP (122 mg, 1 mmol), pyridine (0.32 mL, 4 mmol), *N,N*-dimethylcarbamoyl chloride (0.18 mL, 2 mmol), and CH_2Cl_2 (2 mL), yielded the product **1i** (150 mg, 67%); ^{1}H NMR ($CDCl_3$) δ: 3.05 (s, 6H), 3.855 (s, 3H), 3.859 (s, 3H), 6.57 (dd, *J* = 1.2 and 8.4 Hz, 1H), 7.00 (t, *J* = 8.4 Hz, 1H), 7.19 (brs, 1H), 7.81 (dd, *J* = 1.2 and 8.4 Hz, 1H); ^{13}C NMR ($CDCl_3$), δ: 36.21, 55.67, 60.29, 105.74, 111.59, 124.02, 133.52, 136.89, 151.77, 155.25; HRESIMS calcd. for $C_{11}H_{16}N_2O_3Na$ ($M+Na^+$): 247.1059; found 247.1057.

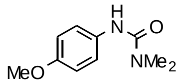

Following the general procedure **A**, using aniline (123 mg, 1 mmol), DMAP (122 mg, 1 mmol), pyridine (0.32 mL, 4 mmol), *N,N*-dimethylcarbamoyl chloride (0.18 mL, 2 mmol), and CH_2Cl_2 (2 mL), yielded the product **1j** (163 mg, 84%); ^{1}H NMR ($CDCl_3$) δ: 3.01 (s, 6H), 3.78 (s, 3H), 6.19 (brs, 1H), 6.83 (d, *J* = 9.2 Hz, 2H), 7.26 (d, *J* = 9.2 Hz, 2H); ^{13}C NMR ($CDCl_3$), δ: 36.58, 55.67, 114.20, 122.50, 132.44, 155.91, 156.44; HRESIMS calcd. for $C_{10}H_{14}N_2O_2Na$ ($M+Na^+$): 217.0953; found 217.0947.

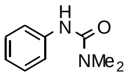

Following the general procedure **A**, using *m*-toluidine (0.91 mL, 10 mmol), DMAP (1.22 g, 10 mmol), pyridine (3.24 mL, 40 mmol), *N,N*-dimethylcarbamoyl chloride (1.84 mL, 20 mmol), and CH_2Cl_2 (20 mL), yielded the product **1k** (1.46 g, 89%); ^{1}H NMR ($CDCl_3$) δ: 3.03 (s, 6H) , 6.32 (brs, 1H), 7.02 (t, *J* = 7.2 Hz, 1H), 7.26-7.31 (m, 2H), 7.35-7.38 (m, 2H); ^{13}C NMR ($CDCl_3$), δ: 36.56, 120.10, 122.99, 128.89, 139.44, 155.97; HRESIMS calcd. for $C_9H_{12}N_2ONa$ ($M+Na^+$): 187.0847; found 187.0839.

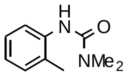

Following the general procedure **A**, using aniline (0.11 mL, 1 mmol), DMAP (122 mg, 1 mmol), pyridine (0.32 mL, 4 mmol), *N,N*-dimethylcarbamoyl chloride (0.18 mL, 2 mmol), and CH_2Cl_2 (2 mL), yielded the product **1l** (138 mg, 77%); ^{1}H NMR ($CDCl_3$) δ: 2.25 (s, 3H), 3.04 (s, 6H), 6.12 (brs, 1H), 6.98-7.02 (m, 1H), 7.14-7.21 (m, 2H), 7.71 (d, *J* = 8 Hz, 1H); ^{13}C NMR ($CDCl_3$), δ: 17.94, 36.64, 122.70, 123.93, 126.91, 128.50, 130.41, 137.39, 156.10; HRESIMS calcd. for $C_{10}H_{14}N_2ONa$ (M+Na$^+$): 201.1004; found 201.1000.

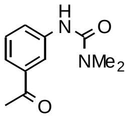

Following the general procedure **A**, using aniline (135 mg, 1 mmol), DMAP (122 mg, 1 mmol), pyridine (0.32 mL, 4 mmol), *N,N*-dimethylcarbamoyl chloride (0.18 mL, 2 mmol), and CH_2Cl_2 (2 mL), yielded the product **1m** (201 mg, 98%); ^{1}H NMR ($CDCl_3$) δ: 2.60 (s, 3H), 3.06 (s, 6H), 6.46 (brs, 1H), 7.39 (t, *J* = 8 Hz, 1H), 7.61 (d, *J* = 8 Hz, 1H), 7.76 (d, *J* = 8 Hz, 1H), 7.89 (s, 1H); ^{13}C NMR ($CDCl_3$), δ: 26.83, 36.62, 119.43, 122.91, 124.77, 129.18, 137.73, 140.10, 155.89, 198.47; HRESIMS calcd. for $C_{11}H_{14}N_2O_2Na$ (M+Na$^+$): 229.0953; found 229.0948.

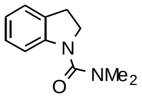

Following the general procedure **A**, using aniline (0.11 mL, 1 mmol), DMAP (122 mg, 1 mmol), pyridine (0.32 mL, 4 mmol), *N,N*-dimethylcarbamoyl chloride (0.18 mL, 2 mmol), and CH_2Cl_2 (2 mL), yielded the product **1n** (189 mg, 99%); ^{1}H NMR ($CDCl_3$) δ: 2.93 (s, 6H), 3.02 (t, *J* = 8 Hz, 2H), 3.90 (t, *J* = 8 Hz, 2H), 6.87-6.94 (m, 2H), 7.11-7.18 (m, 2H); ^{13}C NMR ($CDCl_3$), δ: 28.15, 38.17, 50.37, 113.36, 121.34, 124.84, 127.01, 131.40, 144.37, 160.30; HRESIMS calcd. for $C_{11}H_{14}N_2ONa$ (M+Na$^+$): 213.1004; found 213.0997.

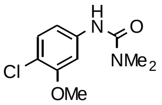

Following the general procedure **A**, using aniline (1 mmol), DMAP (122 mg, 1 mmol), pyridine (0.32 mL, 4 mmol), *N,N*-dimethylcarbamoyl chloride (0.18 mL, 2 mmol), and CH_2Cl_2 (2 mL), yielded the product **1q** (81%);

^{1}H NMR (acetone-*d*$_6$) δ: 2.99 (s, 6H), 3.81 (s, 3H), 3.84 (s, 3H), 7.09 (dd, *J* = 2.4 and 8.7 Hz, 1H), 7.18 (d, *J* = 8.7 Hz, 1H), 7.51 (d, *J* = 2.4 Hz, 1H), 7.89 (brs, 1H). ^{13}C NMR (acetone-*d*$_6$) δ: 35.57, 56.25, 105.02, 112.78, 115.07, 130.15, 142.08, 155.78, 156.44; HRESIMS calcd. for $C_{10}H_{13}N_2O_2Na$ (M+Na$^+$): 251.0563; found 251.0569.

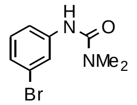

Following the general procedure **A**, using aniline (1 mmol), DMAP (122 mg, 1 mmol), pyridine (0.32 mL, 4 mmol), *N,N*-dimethylcarbamoyl chloride (0.18 mL, 2 mmol), and CH_2Cl_2 (2 mL), yielded the product **1q** (79%);

^{1}H NMR (acetone-d_6) δ: 2.99 (s, 6H), 7.08-7.18 (m, 2H), 7.48 (d, J = 8.0 Hz, 1H), 7.90-7.91 (m, 2H). ^{13}C NMR (acetone-d_6) δ: 36.52, 118.77, 122.47, 122.84, 125.16, 130.88, 143.53, 156.22; HRESIMS calcd. for $C_9H_{11}N_2ONa$ (M+Na$^+$): 264.9952; found 264.9953.

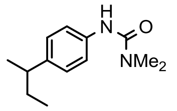

Following the general procedure **A**, using aniline (1 mmol), DMAP (122 mg, 1 mmol), pyridine (0.32 mL, 4 mmol), *N,N*-dimethylcarbamoyl chloride (0.18 mL, 2 mmol), and CH_2Cl_2 (2 mL), yielded the product **1q** (90%);

^{1}H NMR ($CDCl_3$) δ: 0.80 (t, J = 7.6 Hz, 3H), 1.29 (d, J = 6.8 Hz, 3H), 1.56 (m, 2H), 2.53 (m, 1H), 3.03 (s, 6H), 6.22 (brs, 1H), 7.09 (d, J = 8.4 Hz, 1H), 7.28 (d, J = 8.4 Hz, 1H); ^{13}C NMR ($CDCl_3$), δ: 12.13, 21.86, 31.11, 36.25, 40.93, 120.52, 126.98, 137.13, 141.96, 156.28; HRESIMS calcd. for $C_{13}H_{20}N_2ONa$ (M+Na$^+$): 243.1473; found 243.1471.

II. C–H arylations with aryl iodide

General procedure B [analogous as described in ref [3]].

Aryl urea **1** (0.25 mmol), aryl iodide **2** (0.5 mmol), AgOAc (0.5 mmol, 83 mg), and $Pd(OAc)_2$ (0.025 mmol, 5.6 mg) were sequentially added under air to a reaction tube equipped with a stir bar and a septum. An aqueous solution containing the surfactant (1.0 mL, 2 wt %), and 48 wt % HBF_4 (1.25 mmol, 0.16 mL) was added by syringe and the resulting mixture vigorously stirred for 20 h at ambient temperature. After this time, the contents of the flask were quenched with aqueous $NaHCO_3$ and extracted with EtOAc. The solution obtained was filtered through the plug of silica gel and anhydrous $MgSO_4$, and then concentrated by rotary evaporation. The residue was purified by flash chromatography, eluting with hexane/EtOAc to afford the product. All products are reported previously (See also section III).[1,2]

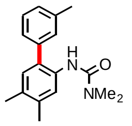

Following the general procedure above, using **1d** (48 mg, 0.25 mmol), *m*-tolyl-I (109 mg, 0.50 mmol), AgOAc (0.5 mmol, 83 mg), and $Pd(OAc)_2$ (0.025 mmol, 5.6 mg), 2 wt % Brij 35 solution (1.0 mL) 48 wt % aqueous HBF_4 (1.25 mmol, 0.16 mL), the product **3r** was obtained (56 mg, 79%), ^{1}H NMR ($CDCl_3$) δ: 2.23 (s, 3H), 2.29 (s, 3H), 2.39 (s, 3H), 2.81 (s, 6H), 6.44 (brs, 1H), 6.96 (s, 1H), 7.15-7.17 (m, 3H), 7.33 (dd, J = 7.2 and 8.2 Hz, 1H), 7.94 (s, 1H).

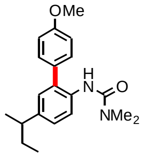

Following the general procedure above, using **1q** (55 mg, 0.25 mmol), *p*-An-I (117 mg, 0.50 mmol), AgOAc (0.5 mmol, 83 mg), and $Pd(OAc)_2$ (0.025 mmol, 5.6 mg), 2 wt % Brij 35 solution (1.0 mL) 48 wt % aqueous HBF_4 (1.25 mmol, 0.16 mL), the product **3cc** was obtained (58 mg, 71%); ^1H NMR ($CDCl_3$) δ: 0.83 (t, *J* = 7.4 Hz, 3H), 1.22 (t, *J* = 6.9 Hz, 3H), 1.54-1.61 (m, 2H), 2.55 (sext, *J* = 6.9 Hz, 1H), 2.81 (s, 6H), 3.85 (s, 3H), 6.43 (brs, 1H), 6.98 (brs, 1H), 7.00 (d, *J* = 8.4 Hz, 2H), 7.13 (dd, *J* = 2.1 and 8.4 Hz, 1H), 7.32 (d, *J* = 8.4 Hz, 2H), 8.01 (d, *J* = 8.4 Hz, 1H).

III. C–H Suzuki–Miyaura

General procedure C [analogous as described in ref [4]].

Arylurea **1** (0.25 mmol), arylboronic acid **2** (1.5–3 equiv), 1,4-benzoquinone (2–5 equiv), and $[Pd(MeCN)_4](BF_4)_2$ (0.025 mmol, 11 mg) were sequentially added in air to a reaction tube equipped with a stir bar and a septum. EtOAc was added by syringe and the resulting mixture vigorously stirred for 20 h at ambient temperature. After this time, the contents of the flask were quenched with aqueous NaOH (to remove 1,4-hydroxybenzene) and extracted with EtOAc. The solution obtained was filtered through the plug of silica gel and anhydrous $MgSO_4$, and then concentrated by rotary evaporation. The residue was purified by flash chromatography, eluting with hexane/EtOAc to afford the product. All products are reported previously except **3ii**.[1,2]

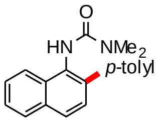

3ii: 97%

Following the general procedure above, using arylurea (53 mg, 0.25 mmol), *p*-tolylB(OH)$_2$ (102 mg, 0.75 mmol), BQ (1.25 mmol, 135 mg), and $[Pd(MeCN)_4](BF_4)_2$ (0.025 mmol, 11 mg), EtOAc (1.0 mL), yielded the product **3ii** (73 mg, 97%); ^1H NMR ($CDCl_3$) δ: 2.41 (s, 3H), 2.91 (s, 6H), 6.02 (brs, 1H), 7.25 (d, *J* = 8.1 Hz, 2H), 7.33 (d, *J* = 8.8 Hz, 2H), 7.43 (d, *J* = 8.1 Hz, 1H), 7.46-7.56 (m, 2H), 7.78 (d, *J* = 8.4 Hz, 1H), 7.83 (d, *J* = 8.8 Hz, 1H), 7.99 (d, *J* = 8.8 Hz, 1H). ^{13}C NMR ($CDCl_3$) δ: 20.85, 36.18, 123.96, 125.45, 126.05, 126.38, 127.29, 127.50, 128.69, 128.81, 130.80, 133.25, 134.71, 136.38, 136.73, 157.26; HRESIMS calcd. for $C_{20}H_{20}N_2ONa$ ($M+Na^+$): 327.1473; found 327.1479.

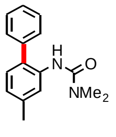

Following the general procedure above, using **1b** (44 mg, 0.25 mmol), PhB(OH)$_2$ (96 mg, 0.75 mmol), BQ (1.25 mmol, 135 mg), and $[Pd(MeCN)_4](BF_4)_2$ (0.025 mmol, 11 mg), EtOAc (1.0 mL), yielded the product **3a** (61 mg, 96%); ^1H NMR ($CDCl_3$) δ: 2.38 (s, 3H), 2.79 (s, 6H), 6.48 (brs,

1H), 6.90 (dd, *J* = 1.0 and 7.7 Hz, 1H), 7.08 (d, *J* = 7.7 Hz, 1H), 7.35-7.38 (m, 3H), 7.43-7.47 (m, 2H), 8.02 (brs, 1H).

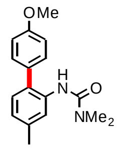

Following the general procedure above, using **1b** (44 mg, 0.25 mmol), *p*-AnB(OH)$_2$ (114 mg, 0.75 mmol), BQ (1.25 mmol, 135 mg), and [Pd(MeCN)$_4$](BF$_4$)$_2$ (0.025 mmol, 11 mg), EtOAc (1.0 mL), yielded the product **3b** (53 mg, 75%); ^{1}H NMR (CDCl$_3$) δ: 2.37 (s, 3H), 2.81 (s, 6H), 3.84 (s, 3H), 6.50 (brs, 1H), 6.87 (dd, *J* = 1.0 and 7.7 Hz, 1H), 6.98 (d, *J* = 8.7 Hz, 2H), 7.05 (d, *J* = 7.7 Hz, 1H), 7.28 (d, *J* = 8.7 Hz, 2H), 8.01 (brs, 1H).

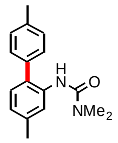

Following the general procedure above, using **1b** (44 mg, 0.25 mmol), *p*-tolylB(OH)$_2$ (102 mg, 0.75 mmol), BQ (1.25 mmol, 135 mg), and [Pd(MeCN)$_4$](BF$_4$)$_2$ (0.025 mmol, 11 mg), EtOAc (1.0 mL), yielded the product **3c** (60 mg, 90%); ^{1}H NMR (CDCl$_3$) δ: 2.38 (s, 3H), 2.40 (s, 3H), 2.81 (s, 6H), 6.52 (brs, 1H), 6.88 (dd, *J* = 1.0 and 7.7 Hz, 1H), 7.07 (d, *J* = 7.7 Hz, 1H), 7.26 (brs, 4H), 8.02 (s, 1H).

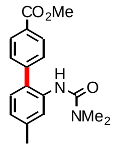

Following the general procedure above, using **1b** (44 mg, 0.25 mmol), *p*-MeCO$_2$C$_6$H$_4$B(OH)$_2$ (135 mg, 0.75 mmol), BQ (1.25 mmol, 135 mg), and [Pd(MeCN)$_4$](BF$_4$)$_2$ (0.025 mmol, 11 mg), EtOAc (1.0 mL), yielded the product **3d** (73 mg, 94%); ^{1}H NMR (CDCl$_3$) δ: 2.38 (s, 3H), 2.82 (s, 6H), 3.94 (s, 3H), 6.37 (brs, 1H), 6.92 (dd, *J* = 1.2 and 8.4 Hz, 1H), 7.09 (d, *J* = 7.8 Hz, 1H), 7.46 (d, *J* = 7.8 Hz, 2H), 7.95 (brs, 1H), 8.12 (d, *J* = 8.4 Hz, 2H).

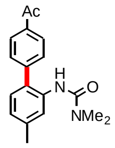

Following the general procedure above, using **1b** (44 mg, 0.25 mmol), *p*-AcC$_6$H$_4$B(OH)$_2$ (123 mg, 0.75 mmol), BQ (1.25 mmol, 135 mg), and [Pd(MeCN)$_4$](BF$_4$)$_2$ (0.025 mmol, 11 mg), EtOAc (1.0 mL), yielded the product **3e** (63 mg, 86%); ^{1}H NMR (CDCl$_3$) δ: 2.38 (s, 3H), 2.64 (s, 3H), 2.83 (s,

6H), 6.38 (brs, 1H), 6.93 (d, J = 7.7 Hz, 1H), 7.09 (d, J = 7.7 Hz, 1H), 7.49 (d, J = 8.3 Hz, 2H), 7.93 (brs, 1H), 8.04 (d, J = 8.3 Hz, 2H).

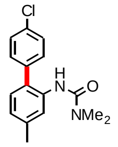

Following the general procedure above, using **1b** (44 mg, 0.25 mmol), *p*-$ClC_6H_4B(OH)_2$ (117 mg, 0.75 mmol), BQ (1.25 mmol, 135 mg), and $[Pd(MeCN)_4](BF_4)_2$ (0.025 mmol, 11 mg), EtOAc (1.0 mL), yielded the product **3f** (67 mg, 94%); ^1H NMR ($CDCl_3$) δ: 2.37 (s, 3H), 2.83 (s, 6H), 6.33 (brs, 1H), 6.91 (d, J = 7.7 Hz, 1H), 7.05 (d, J = 7.7 Hz, 1H), 7.30 (d, J = 8.4 Hz, 2H), 7.42 (d, J = 8.4 Hz, 2H), 7.93 (brs, 1H).

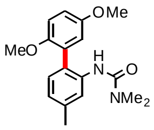

Following the general procedure above, using **1b** (44 mg, 0.25 mmol), 2,5-$(MeO)_2C_6H_3B(OH)_2$ (136 mg, 0.75 mmol), BQ (1.25 mmol, 135 mg), and $[Pd(MeCN)_4](BF_4)_2$ (0.025 mmol, 11 mg), EtOAc (1.0 mL), yielded the product **3g** (77 mg, 98%); ^1H NMR ($CDCl_3$) δ: 2.38 (s, 3H), 2.83 (s, 6H), 3.74 (s, 3H), 3.77 (s, 3H), 6.79 (d, J = 3.0 Hz, 1H), 6.89 (dd, J = 3.0 and 8.9 Hz, 1H), 6.89-6.90 (m, 2H), 7.03 (brs, 1H), 7.10 (d, J = 7.8 Hz, 1H), 7.78 (brs, 1H).

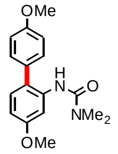

Following the general procedure above, using **1a** (48 mg, 0.25 mmol), *p*-$AnB(OH)_2$ (114 mg, 0.75 mmol), BQ (0.5 mmol, 54 mg), and $[Pd(MeCN)_4](BF_4)_2$ (0.025 mmol, 11 mg), EtOAc (1.0 mL), yielded the product **3h** (60 mg, 81%); ^1H NMR ($CDCl_3$) δ: 2.81 (s, 6H), 3.84 (s, 6H), 6.60 (brs, 1H), 6.61 (dd, J = 2.6 and 8.3 Hz, 1H), 6.98 (d, J = 8.6 Hz, 2H), 7.05 (d, J = 8.3 Hz, 1H), 7.27 (d, J = 8.6 Hz, 2H), 7.92 (d, J = 2.6 Hz, 1H).

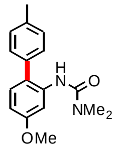

Following the general procedure above, using **1a** (48 mg, 0.25 mmol), *p*-tolyl$B(OH)_2$ (102 mg, 0.75 mmol), BQ (0.5 mmol, 54 mg), and $[Pd(MeCN)_4](BF_4)_2$ (0.025 mmol, 11 mg), EtOAc (1.0 mL), yielded the product **3i** (61 mg, 87%); ^1H NMR ($CDCl_3$) δ: 2.40 (s, 3H), 2.81 (s, 6H), 3.85 (s, 3H), 6.63 (dd, J = 2.7 and 8.5 Hz, 1H), 6.64 (brs, 1H), 7.02 (d, J = 8.5 Hz, 1H), 7.25 (brs, 4H), 7.94 (d, J = 2.7 Hz, 1H).

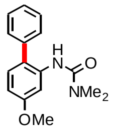

Following the general procedure above, using **1a** (48 mg, 0.25 mmol), PhB(OH)$_2$ (96 mg, 0.75 mmol), BQ (0.5 mmol, 54 mg), and [Pd(MeCN)$_4$](BF$_4$)$_2$ (0.025 mmol, 11 mg), EtOAc (1.0 mL), yielded the product **3j** (62 mg, 70%); ^{1}H NMR (CDCl$_3$) δ: 2.96 (s, 6H), 3.85 (s, 3H), 6.60 (brs, 1H), 6.63 (dd, *J* = 2.6 and 8.4 Hz, 1H), 7.08 (d, *J* = 8.6 Hz, 1H), 7.34-7.38 (m, 3H), 7.43-7.47 (m, 2H), 7.94 (d, *J* = 2.6 Hz, 1H).

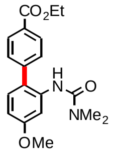

Following the general procedure above, using **1a** (48 mg, 0.25 mmol), **2d** (135 mg, 0.75 mmol), BQ (0.5 mmol, 54 mg), and [Pd(MeCN)$_4$](BF$_4$)$_2$ (0.025 mmol, 11 mg), EtOAc (1.0 mL), yielded the product **3k** (60 mg, 70%), ^{1}H NMR (CDCl$_3$) δ: 1.41 (t, *J* = 7.1 Hz, 3H), 2.82 (s, 6H), 3.84 (s, 3H), 4.39 (q, *J* = 7.1 Hz, 2H), 6.49 (brs, 1H), 6.65 (dd, *J* = 2.6 and 8.4 Hz, 1H), 7.08 (d, *J* = 8.4 Hz, 1H), 7.44 (d, *J* = 8.4 Hz, 2H), 7.88 (d, *J* = 2.6 Hz, 1H), 8.11 (d, *J* = 8.4 Hz, 2H).

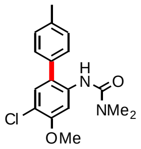

Following the general procedure above, using **1o** (57 mg, 0.25 mmol), *p*-tolylB(OH)$_2$ (102 mg, 0.75 mmol), BQ (1.25 mmol, 135 mg), and [Pd(MeCN)$_4$](BF$_4$)$_2$ (0.025 mmol, 11 mg), EtOAc (1.0 mL), yielded the product **3l** (77 mg, 97%); ^{1}H NMR (CDCl$_3$) δ: 2.40 (s, 3H), 2.81 (s, 6H), 3.95 (s, 3H), 6.65 (brs, 1H), 7.22 (d, *J* = 8.0 Hz, 2H), 7.27 (d, *J* = 8.0 Hz, 2H), 8.09 (brs, 1H).

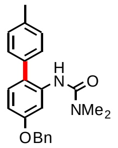

Following the general procedure above, using **1e** (67 mg, 0.25 mmol), *p*-tolylB(OH)$_2$ (102 mg, 0.75 mmol), BQ (1.25 mmol, 135 mg), and [Pd(MeCN)$_4$](BF$_4$)$_2$ (0.025 mmol, 11 mg), EtOAc (1.0 mL), yielded the product **3m** (72 mg, 80%);^{1}H NMR (CDCl$_3$) δ: 2.40 (s, 3H), 2.82 (s, 6H), 5.11 (s, 2H), 6.65 (brs, 1H), 6.70 (dd, *J* = 2.6 and 8.4 Hz, 1H), 7.08 (d, *J* = 8.4 Hz, 1H), 7.26 (brs, 4H), 7.32 (d, *J* = 7.2 Hz, 1H), 7.39 (t, *J* = 7.2 Hz, 2H), 7.47 (d, *J* = 7.2 Hz, 2H), 8.05 (d, *J* = 2.6 Hz, 1H).

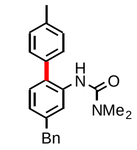

Following the general procedure above, using arylurea (63 mg, 0.25 mmol), *p*-tolylB(OH)$_2$ (102 mg, 0.75 mmol), BQ (1.25 mmol, 135 mg), and [Pd(MeCN)$_4$](BF$_4$)$_2$ (0.025 mmol, 11 mg), EtOAc (1.0 mL), yielded the product **3n** (69 mg, 80%);^{1}H NMR (CDCl$_3$) δ: 2.38 (s, 3H), 2.79 (s, 6H), 3.98 (s, 2H), 6.54 (brs, 1H), 6.85 (dd, *J* = 1.7 and 7.8 Hz, 1H), 7.07 (d, *J* = 7.8 Hz, 1H), 7.14-7.19 (m, 1H), 7.24-7.27 (m, 8H), 8.11 (d, *J* = 1.7 Hz, 1H).

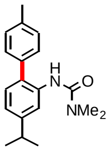

Following the general procedure above, using **1c** (51 mg, 0.25 mmol), *p*-tolylB(OH)$_2$ (102 mg, 0.75 mmol), BQ (1.25 mmol, 135 mg), and [Pd(MeCN)$_4$](BF$_4$)$_2$ (0.025 mmol, 11 mg), EtOAc (1.0 mL), yielded the product **3o** (66 mg, 89%); ^{1}H NMR (CDCl$_3$) δ: 1.29 (t, *J* = 7.0 Hz, 6H), 2.40 (s, 3H), 2.82 (s, 6H), 2.95 (sept, *J* = 7.0 Hz, 1H), 6.57 (brs, 1H), 6.93 (dd, *J* = 1.7 and 8.7 Hz, 1H), 7.11 (d, *J* = 8.7 Hz, 1H), 7.26 (brs, 4H), 8.10 (d, *J* = 1.7 Hz, 1H).

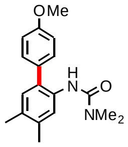

Following the general procedure above, using **1d** (48 mg, 0.25 mmol), *p*-AnB(OH)$_2$ (114 mg, 0.75 mmol), BQ (1.25 mmol, 135 mg), and [Pd(MeCN)$_4$](BF$_4$)$_2$ (0.025 mmol, 11 mg), EtOAc (1.0 mL), yielded the product **3p** (64 mg, 86%), ^{1}H NMR (CDCl$_3$) δ: 2.22 (s, 3H), 2.28 (s, 3H), 2.82 (s, 6H), 3.85 (s, 3H), 6.38 (brs, 1H), 6.95 (brs, 1H), 6.98 (dd, *J* = 8.8 Hz, 2H), 7.28 (d, *J* = 8.8 Hz, 2H), 7.91 (s, 1H).

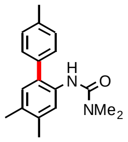

Following the general procedure above, using **1d** (48 mg, 0.25 mmol), *p*-tolylB(OH)$_2$ (102 mg, 0.75 mmol), BQ (1.25 mmol, 135 mg), and [Pd(MeCN)$_4$](BF$_4$)$_2$ (0.025 mmol, 11 mg), EtOAc (1.0 mL), yielded the product **3q** (67 mg, 96%), ^{1}H NMR (CDCl$_3$) δ: 2.22 (s, 3H), 2.29 (s, 3H), 2.39 (s, 3H), 2.81 (s, 6H), 6.42 (brs, 1H), 6.96 (s, 1H), 7.25 (brs, 4H), 7.92 (s, 1H).

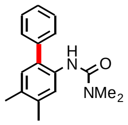

Following the general procedure above, using **1d** (48 mg, 0.25 mmol), PhB(OH)$_2$ (96 mg, 0.75

mmol), BQ (1.25 mmol, 135 mg), and $[Pd(MeCN)_4](BF_4)_2$ (0.025 mmol, 11 mg), EtOAc (1.0 mL), yielded the product **3s** (64 mg, 95%), ^{1}H NMR ($CDCl_3$) δ: 2.23 (s, 3H), 2.30 (s, 3H), 2.80 (s, 6H), 6.37 (brs, 1H), 6.98 (s, 1H), 7.34-7.38(m, 3H), 7.43-7.47 (m, 2H), 7.93 (s, 1H).

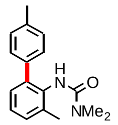

Following the general procedure above, using **1l** (44 mg, 0.25 mmol), *p*-tolylB(OH)$_2$ (102 mg, 0.75 mmol), BQ (1.25 mmol, 135 mg), and $[Pd(MeCN)_4](BF_4)_2$ (0.025 mmol, 11 mg), EtOAc (1.0 mL), yielded the product **3t** (43 mg, 65%); ^{1}H NMR ($CDCl_3$) δ: 2.32 (s, 3H), 2.39 (s, 3H), 2.85 (s, 6H), 5.71 (brs, 1H), 7.12 (dd, *J* = 1.8 and 7.5 Hz, 1H), 7.17 (t, *J* = 7.5 Hz, 1H), 7.20-7.26 (m, 5H).

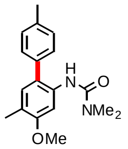

Following the general procedure above, using **1h** (52 mg, 0.25 mmol), *p*-tolylB(OH)$_2$ (102 mg, 0.75 mmol), BQ (1.25 mmol, 135 mg), and $[Pd(MeCN)_4](BF_4)_2$ (0.025 mmol, 11 mg), EtOAc (1.0 mL), yielded the product **3u** (60 mg, 80%); ^{1}H NMR ($CDCl_3$) δ: 2.18 (s, 3H), 2.39 (s, 3H), 2.82 (s, 6H), 3.89 (s, 3H), 6.59 (brs, 1H), 6.94 (s, 1H), 7.24 (brs, 4H), 7.87 (s, 1H).

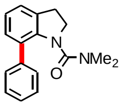

Following the general procedure above, using **1n** (47 mg, 0.25 mmol), PhB(OH)$_2$ (96 mg, 0.75 mmol), BQ (1.25 mmol, 135 mg), and $[Pd(MeCN)_4](BF_4)_2$ (0.025 mmol, 11 mg), EtOAc (1.0 mL), yielded the product **3v** (60 mg, 90%); ^{1}H NMR ($CDCl_3$) δ: 2.51 (s, 6H), 3.07 (t, *J* = 8.0 Hz, 2H), 3.94 (t, *J* = 8.0 Hz, 2H), 6.99 (t, *J* = 7.8 Hz, 1H), 7.12 (t, *J* = 8.1 Hz, 2H), 7.20-7.24 (m, 2H), 7.32 (d, *J* = 7.8 Hz, 2H), 7.39 (d, *J* = 8.1 Hz, 2H).

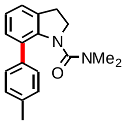

Following the general procedure above, using **1n** (47 mg, 0.25 mmol), *p*-tolylB(OH)$_2$ (102 mg, 0.75 mmol), BQ (1.25 mmol, 135 mg), and $[Pd(MeCN)_4](BF_4)_2$ (0.025 mmol, 11 mg), EtOAc (1.0 mL), yielded the product **3w** (62 mg, 89%); ^{1}H NMR ($CDCl_3$) δ: 2.34 (s, 3H), 2.57 (s, 6H), 3.09 (t, *J* = 7.9 Hz, 2H), 3.96 (t, *J* = 7.9 Hz, 2H), 6.99 (t, *J* = 7.8 Hz, 1H), 6.98-7.02 (m, 4H), 7.26 (brs, 1H), 7.32 (d, *J* = 8.0 Hz, 2H).

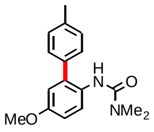

Following the general procedure above, using **1j** (48 mg, 0.25 mmol), *p*-tolylB(OH)$_2$ (51 mg, 0.375 mmol), BQ (1.25 mmol, 135 mg), and [Pd(MeCN)$_4$](BF$_4$)$_2$ (0.025 mmol, 11 mg), EtOAc (1.0 mL), yielded the product **3aa** (58 mg, 82%), ^{1}H NMR (CDCl$_3$) δ: 2.39 (s, 3H), 2.80 (s, 6H), 3.78 (s, 3H), 6.29 (brs, 1H), 6.75 (d, *J* = 3.0 Hz, 1H), 6.87 (dd, *J* = 3.0 and 9.0 Hz, 1H), 7.28 (brs, 4H), 7.93 (d, *J* = 9.0 Hz, 1H).

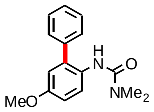

Following the general procedure above, using **1j** (48 mg, 0.25 mmol), PhB(OH)$_2$ (46 mg, 0.375 mmol), BQ (1.25 mmol, 135 mg), and [Pd(MeCN)$_4$](BF$_4$)$_2$ (0.025 mmol, 11 mg), EtOAc (1.0 mL), yielded the product **3bb** (60 mg, 89%), ^{1}H NMR (CDCl$_3$) δ: 2.79 (s, 6H), 3.79 (s, 3H), 6.24 (brs, 1H), 6.77 (d, *J* = 3.0 Hz, 1H), 6.89 (dd, *J* = 3.0 and 9.0 Hz, 1H), 7.26-7.40 (m, 3H), 7.44-7.48 (m, 2H), 7.93 (d, *J* = 9.0 Hz, 1H).

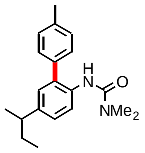

Following the general procedure above, using **1q** (55 mg, 0.25 mmol), *p*-tolylB(OH)$_2$ (51 mg, 0.375 mmol), BQ (1.25 mmol, 135 mg), and [Pd(MeCN)$_4$](BF$_4$)$_2$ (0.025 mmol, 11 mg), EtOAc (1.0 mL), yielded the product **3dd** (68 mg, 88%); ^{1}H NMR (CDCl$_3$) δ: 0.83 (t, *J* = 7.3 Hz, 3H), 1.22 (t, *J* = 7.0 Hz, 3H), 1.52-1.61 (m, 2H), 2.40 (s, 3H), 2.56 (sext, *J* = 7.0 Hz, 1H), 2.81 (s, 6H), 6.47 (brs, 1H), 6.99 (d, *J* = 2.2 Hz, 1H), 7.13 (dd, *J* = 2.2 and 8.4 Hz, 1H), 7.25-7.30 (m, 4H), 8.01 (d, *J* = 8.4 Hz, 1H).

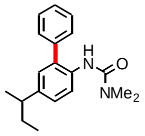

Following the general procedure above, using **1q** (55 mg, 0.25 mmol), PhB(OH)$_2$ (46 mg, 0.375 mmol), BQ (1.25 mmol, 135 mg), and [Pd(MeCN)$_4$](BF$_4$)$_2$ (0.025 mmol, 11 mg), EtOAc (1.0 mL), yielded the product **3ee** (59 mg, 80%); ^{1}H NMR (CDCl$_3$) δ: 0.83 (t, *J* = 7.5 Hz, 3H), 1.22 (t, *J* = 6.9 Hz, 3H), 1.56 (sept, *J* = 7.5 Hz, 2H), 2.54-2.62 (m, 1H), 2.79 (s, 6H), 6.42 (brs, 1H), 7.00 (d, *J* = 1.9 Hz, 1H), 7.15 (dd, *J* = 1.9 and 8.5 Hz, 1H), 7.35-7.40 (m, 3H), 7.44-7.48 (m, 2H), 8.02 (t, *J* = 8.5 Hz, 1H).

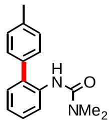

Following the general procedure above, using **1k** (41 mg, 0.25 mmol), *p*-tolylB(OH)$_2$ (51 mg, 0.375 mmol), BQ (1.25 mmol, 135 mg), and [Pd(MeCN)$_4$](BF$_4$)$_2$ (0.025 mmol, 11 mg), EtOAc (1.0 mL), yielded the product **3gg** (50 mg, 78%); ^{1}H NMR (CDCl$_3$) δ: 2.40 (s, 3H), 2.80 (s, 6H), 6.55 (brs, 1H), 7.05 (t, *J* = 7.4 Hz, 1H), 7.17 (d, *J* = 7.4 Hz, 1H), 7.27 (brs, 4H), 7.31 (t, *J* = 8.3 Hz, 1H), 8.17 (d, *J* = 8.3 Hz, 1H).

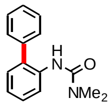

Following the general procedure above, using **1k** (41 mg, 0.25 mmol), PhB(OH)$_2$ (46 mg, 0.375 mmol), BQ (1.25 mmol, 135 mg), and [Pd(MeCN)$_4$](BF$_4$)$_2$ (0.025 mmol, 11 mg), EtOAc (1.0 mL), yielded the product **3hh** (50 mg, 83%); ^{1}H NMR (CDCl$_3$) δ: 2.78 (s, 6H), 6.51 (brs, 1H), 7.06 (dt, *J* = 1.2 and 7.5 Hz, 1H), 7.19 (dd, *J* = 1.6 and 7.5 Hz, 1H), 7.31-7.40 (m, 4H), 7.45-7.49 (m, 2H), 8.16 (dd, *J* = 1.2 and 7.4 Hz, 1H).

IV. Fujiwara–Moritani reactions

General procedure D in water [analogous as described in ref [5]].

Arylurea **1** (0.25 mmol), acrylate ester (0.5 mmol), 1,4-benzoquinone (0.25 mmol, 27 mg), AgOAc (0.5 mmol, 85 mg), and Pd(OAc)$_2$ (0.025 mmol, 5.6 mg) were sequentially added under air to a reaction tube equipped with a stir bar and a septum. An aqueous solution containing the surfactant (1.0 mL, 2 wt %), and 48 wt % HBF$_4$ (1.25 mmol, 0.16 mL) was added by syringe and the resulting mixture vigorously stirred for 20 h at ambient temperature. After this time, the contents of the flask were quenched with aqueous NaHCO$_3$ and extracted with EtOAc. The solution obtained was filtered through the plug of silica gel and anhydrous MgSO$_4$, and then concentrated by rotary evaporation. The residue was purified by flash chromatography, eluting with hexane/EtOAc to afford the product. All products are reported except **5d**–**5h**.[3]

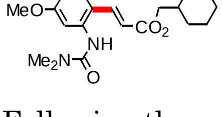

Following the general procedure above, using **1a** (48 mg, 0.25 mmol), acrylate ester (92 mg, 0.50 mmol), 1,4-benzoquinone (0.25 mmol, 27 mg), AgNO$_3$ (0.5 mmol, 85 mg), and [Pd(MeCN)$_4$](BF$_4$)$_2$ (0.025 mmol, 11 mg), 2 wt % PTS solution (1.0 mL), yielded the product **5a** (69 mg, 74%), ^{1}H NMR (CDCl$_3$) δ: 0.90 (t, *J* = 6.8 Hz, 3H), 0.91 (t, *J* = 7.5 Hz, 3H), 1.25-1.45 (m, 8H), 1.60-1.67 (m,

1H), 3.06 (s, 6H), 3.82 (s, 3H), 4.08 (dd, *J* = 6.0 and 11 Hz, 1H), 4.11 (dd, *J* = 5.7 and 11 Hz, 1H), 6.28 (d, *J* = 15.7 Hz, 1H), 6.45 (brs, 1H), 6.67 (dd, *J* = 2.6 and 8.7 Hz, 1H), 7.40 (d, *J* = 2.5 Hz, 1H), 7.46 (d, *J* = 8.7 Hz, 1H), 7.75 (d, *J* = 15.7 Hz, 1H).

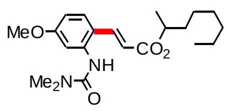

Following the general procedure above, using **1a** (48 mg, 0.25 mmol), acrylate ester (120 mg, 0.50 mmol), 1,4-benzoquinone (0.25 mmol, 27 mg), $AgNO_3$ (0.5 mmol, 85 mg), and $[Pd(MeCN)_4](BF_4)_2$ (0.025 mmol, 11 mg), 2 wt % PTS solution (1.0 mL), yielded the product **5b** (71 mg, 76%), ^{1}H NMR ($CDCl_3$) δ: 0.88 (t, *J* = 7.2 Hz, 3H), 1.26-1.49 (m, 12H), 1.55-1.61 (m, 1H), 3.07 (s, 6H), 3.82 (s, 3H), 5.00 (sext, *J* = 6.2 Hz, 1H), 6.28 (d, *J* = 15.7 Hz, 1H), 6.43 (brs, 1H), 6.67 (dd, *J* = 2.6 and 8.7 Hz, 1H), 7.41 (d, *J* = 2.6 Hz, 1H), 7.45 (d, *J* = 8.7 Hz, 1H), 7.75 (d, *J* = 15.7 Hz, 1H).

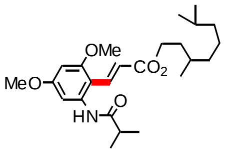

Following the general procedure above, using **1g** (56 mg, 0.25 mmol), acrylate ester (106 mg, 0.50 mmol), 1,4-benzoquinone (0.25 mmol, 27 mg), $AgNO_3$ (0.5 mmol, 85 mg), and $[Pd(MeCN)_4](BF_4)_2$ (0.025 mmol, 11 mg), 2 wt % PTS solution (1.0 mL), yielded the product **5c** (86 mg, 80%), ^{1}H NMR ($CDCl_3$) δ: 0.86 (d, *J* = 6.5 Hz, 6H), 0.92 (d, *J* = 6.5 Hz, 3H), 1.11-1.17 (m, 3H), 1.31 (d, *J* = 6.8 Hz, 6H), 1.23-1.33 (m, 3H), 1.45-1.54 (m, 3H), 1.69-1.77 (m, 1H), 2.57 (sept, *J* = 6.8 Hz, 1H), 3.83 (s, 3H), 3.84 (s, 3H), 4.19-4.25 (m, 2H), 6.25 (s, 1H), 6.48 (d, *J* = 16.2 Hz, 1H), 7.35 (s, 1H), 7.38 (brs, 1H), 7.75 (d, *J* = 16.2 Hz, 1H).

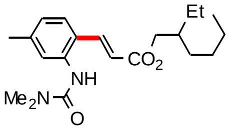

Following the general procedure above, using using **1b** (44 mg, 0.25 mmol), acrylate ester (92 mg, 0.50 mmol), 1,4-benzoquinone (0.25 mmol, 27 mg), AgOAc (0.5 mmol, 83 mg), and $[Pd(MeCN)_4](BF_4)_2$ (0.025 mmol, 11 mg), 2 wt % Brij 35 solution (1.0 mL), yielded the product **5d** (77 mg, 86%), ^{1}H NMR ($CDCl_3$) δ: 0.89-0.92 (m, 6H), 1.25-1.43 (m, 8H), 1.54-1.64 (m, 1H), 2.33 (s, 3H), 3.03 (s, 6H), 4.03 (dd, *J* = 6.0 and 11.0 Hz, 1H), 4.07 (dd, *J* = 5.7 and 11.0 Hz, 1H), 6.34 (d, *J* = 15.8 Hz, 1H), 6.46 (brs, 1H), 6.94 (d, *J* = 8.1 Hz, 1H), 7.42-7.44 (m, 2H), 7.75 (d, *J* = 15.8 Hz, 1H). ^{13}C NMR ($CDCl_3$) δ: 11.16, 14.19, 21.63, 23.11, 29.07, 30.58, 36.64, 38.95, 67.04, 118.89, 124.94, 125.75, 125.87, 126.94, 137.60, 139.83, 141.42, 156.12, 167.41; HRESIMS calcd. for $C_{21}H_{32}N_2O_3Na$ ($M+Na^+$): 383.2310; found 383.2311.

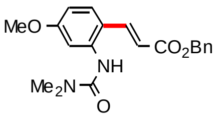

Following the general procedure above, using using **1a** (48 mg, 0.25 mmol), acrylate ester (81 mg, 0.50 mmol), 1,4-benzoquinone (0.25 mmol, 27 mg), AgOAc (0.5 mmol, 83 mg), and [Pd(MeCN)$_4$](BF$_4$)$_2$ (0.025 mmol, 11 mg), 2 wt % Brij 35 solution (1.0 mL), yielded the product **5e** (88 mg, 99%), ^{1}H NMR (CDCl$_3$) δ: 2.99 (s, 6H), 3.78 (s, 3H), 5.18 (s, 2H), 6.29 (d, *J* = 15.7 Hz, 1H), 6.60 (brs, 1H), 6.65 (dd, *J* = 2.6 and 8.8 Hz, 1H), 7.26 (d, *J* = 2.6 Hz, 1H), 7.31-7.37 (m, 5H), 7.42 (d, *J* = 8.8 Hz, 1H), 7.77 (d, *J* = 15.9 Hz, 1H). ^{13}C NMR (CDCl$_3$) δ: 36.63, 55.55, 66.37, 109.11, 111.96, 116.48, 119.71, 128.25, 128.31, 128.53, 128.69, 136.24, 139.63, 140.19, 155.96, 161.91, 167.19; HRESIMS calcd. for $C_{20}H_{22}N_2O_4Na$ (M+Na$^+$): 377.1477; found 377.1478.

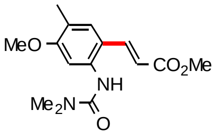

Following the general procedure above, using using **1h** (52 mg, 0.25 mmol), acrylate ester (43 mg, 0.50 mmol), 1,4-benzoquinone (0.25 mmol, 27 mg), AgOAc (0.5 mmol, 83 mg), and [Pd(MeCN)$_4$](BF$_4$)$_2$ (0.025 mmol, 11 mg), 2 wt % Brij 35 solution (1.0 mL), yielded the product **5f** (65 mg, 89%), ^{1}H NMR (CDCl$_3$) δ: 2.15 (s, 3H), 3.04 (s, 6H), 3.74 (s, 3H), 3.81 (s, 3H), 6.24 (d, *J* = 15.8 Hz, 1H), 6.58 (brs, 1H), 7.18 (s, 1H), 7.26 (s, 1H), 7.74 (d, *J* = 15.8 Hz, 1H). ^{13}C NMR (CDCl$_3$) δ: 15.94, 36.66, 51.69, 55.59, 106.68, 116.05, 119.04, 123.50, 128.53, 137.43, 139.89, 156.15, 159.96, 167.96; HRESIMS calcd. for $C_{15}H_{20}N_2O_4Na$ (M+Na$^+$): 315.1321; found 315.1322.

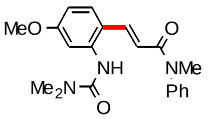

Following the general procedure above, using **1a** (48 mg, 0.25 mmol), acrylamide (80 mg, 0.50 mmol), 1,4-benzoquinone (0.25 mmol, 27 mg), AgOAc (0.5 mmol, 83 mg), and [Pd(MeCN)$_4$](BF$_4$)$_2$ (0.025 mmol, 11 mg), 2 wt % Brij 35 solution (1.0 mL), yielded the product **5g** (73 mg, 83%), ^{1}H NMR (CDCl$_3$) δ: 3.05 (s, 6H), 3.28 (s, 3H), 3.76 (s, 3H), 6.17 (d, *J* = 15.3 Hz, 1H), 6.52 (dd, *J* = 2.6 and 8.8 Hz, 1H), 6.87 (brs, 1H), 7.07 (d, *J* = 8.8 Hz, 1H), 7.15 (d, *J* = 7.2 Hz, 1H), 7.33-7.43 (m, 4H), 7.77 (d, *J* = 15.3 Hz, 1H). ^{13}C NMR (CDCl$_3$) δ: 36.69, 37.56, 55.50, 108.19, 111.59, 117.75, 119.58, 127.40, 127.77, 127.95, 129.76, 136.56, 139.40, 143.57, 155.87, 161.30, 166.42; HRESIMS calcd. for $C_{20}H_{23}N_3O_3Na$ (M+Na$^+$): 376.1637; found 376.1639.

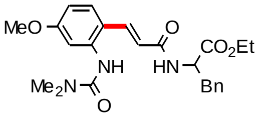

Following the general procedure above, using **1a** (48 mg, 0.25 mmol), acrylamide (123 mg, 0.50 mmol), 1,4-benzoquinone (0.25 mmol, 27 mg), AgOAc (0.5 mmol, 83 mg), and $[Pd(MeCN)_4](BF_4)_2$ (0.025 mmol, 11 mg), 2 wt % Brij 35 solution (1.0 mL), yielded the product **5h** (75 mg, 69%), ^{1}H NMR ($CDCl_3$) δ: 1.21 (t, *J* = 7.2 Hz, 3H), 3.02 (s, 6H), 3.11 (d, *J* = 6.0 Hz, 2H), 3.78 (s, 3H), 4.15 (q, *J* = 7.1 Hz, 2H), 4.84-4.89 (m, 1H), 6.20 (d, *J* = 16.2 Hz, 1H), 6.30-6.34 (m, 1H), 6.60 (dd, *J* = 2.5 and 8.7 Hz, 1H), 6.77 (brs, 1H), 7.10 (dd, *J* = 1.6 and 7.9 Hz, 1H), 7.20-7.28 (m, 2H), 7.33 (s, 1H), 7.36 (d, *J* = 8.8 Hz, 1H), 7.72 (d, *J* = 16.2 Hz, 1H). ^{13}C NMR ($CDCl_3$) δ: 14.23, 36.69, 38.07, 53.56, 55.54, 61.67, 108.56, 111.79, 119.17, 119.39, 127.18, 127.87, 128.64, 129.47, 136.11, 136.43, 139.43, 155.95, 161.49, 165.65, 171.83; HRESIMS calcd. for $C_{24}H_{29}N_3O_5Na$ (M+Na$^+$): 462.2005; found 462.2005.

General procedure E in EtOAc [similar as described in ref. [5]].

Arylurea **1** (0.25 mmol), acrylate ester (0.5 mmol), 1,4-benzoquinone (0.75 mmol, 81 mg), and $Pd(OAc)_2$ (0.025 mmol, 5.6 mg) were sequentially added under air to a reaction tube equipped with a stir bar and a septum. EtOAc (1.0 mL) and 48 wt % HBF_4 (0.25 mmol, 32 uL) were added by syringe and the resulting mixture vigorously stirred for 20 h at ambient temperature. After this time, the contents of the flask were quenched with aqueous $NaHCO_3$ and extracted with EtOAc. The solution obtained was filtered through the plug of silica gel and anhydrous $MgSO_4$, and then concentrated by rotary evaporation. The residue was purified by flash chromatography, eluting with hexane/EtOAc to afford the product.

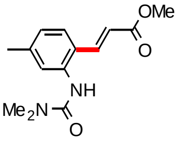

Following the general procedure above, using aryl urea **1b** (44.6 mg, 0.25 mmol), acrylate ester (43 mg, 0.5 mmol), 1,4-benzoquinone (0.75 mmol, 81 mg), $Pd(OAc)_2$ (0.025 mmol, 5.6 mg), 48 wt % HBF_4 (0.25 mmol, 32 uL), and EtOAc (1 mL), yielded the product **5i** (61.2 mg, 93%); ^{1}H NMR ($CDCl_3$) δ: 2.34 (s, 3H), 3.06 (s, 6H), 3.79 (s, 3H), 6.26 (brs, 1H), 6.36 (d, *J* = 15.5 Hz, 1H), 6.94 (d, *J* = 8 Hz, 1H), 7.41 (d, *J* = 8 Hz, 1H),7.48 (s, 1H), 7.80 (d, *J* = 15.5 Hz, 1H); ^{13}C NMR ($CDCl_3$), δ: 21.53, 36.57, 51.68, 118.02, 125.21, 125.90, 126.06, 126.84, 137.76, 140.38, 141.33, 156.26, 167.72; HRESIMS calcd. for $C_{14}H_{18}N_2O_3Na$ (M+Na$^+$): 285.1215; found 285.1206.

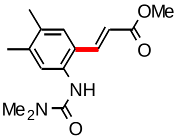

Following the general procedure above, using aryl urea **1d** (48.1 mg, 0.25 mmol), acrylate ester (43 mg, 0.5 mmol), 1,4-benzoquinone (0.75 mmol, 81 mg), Pd(OAc)$_2$ (0.025 mmol, 5.6 mg), 48 wt % HBF$_4$ (0.25 mmol, 32 uL), and EtOAc (1 mL), yielded the product **5j** (68.3 mg, 99%); ^{1}H NMR (CDCl$_3$) δ: 2.23 (s, 3H), 2.24 (s, 3H), 3.05 (s, 6H), 3.78 (s, 3H), 6.19 (brs, 1H), 6.36 (d, *J* = 15.5 Hz, 1H), 7.30 (s, 1H), 7.35 (s, 1H), 7.80 (d, *J* = 15.5 Hz, 1H); ^{13}C NMR (CDCl$_3$), δ: 19.33, 19.96, 36.62, 51.69, 117.71, 125.85, 127.09, 127.71, 133.57, 135.60, 140.10, 140.49, 156.53, 167.72; HRESIMS calcd. for $C_{15}H_{20}N_2O_3Na$ (M+Na$^+$): 299.1372; found 299.1364.

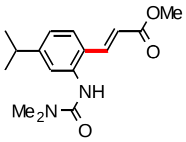

Following the general procedure above, using aryl urea **1c** (51.6 mg, 0.25 mmol), acrylate ester (43 mg, 0.5 mmol), 1,4-benzoquinone (0.75 mmol, 81 mg), Pd(OAc)$_2$ (0.025 mmol, 5.6 mg), 48 wt % HBF$_4$ (0.25 mmol, 32 uL), and EtOAc (1 mL), yielded the product **5k** (69.4 mg, 96%); ^{1}H NMR (CDCl$_3$) δ: 1.24 (d, *J* = 7 Hz, 6H), 2.89 (sept, *J* = 7 Hz, 1H), 3.06 (s, 6H), 3.79 (s, 3H), 6.28 (brs, 1H), 6.37 (d, *J* = 16 Hz, 1H), 7.01 (d, *J* = 8 Hz, 1H), 7.46 (d, *J* = 8 Hz, 1H), 7.52 (s, 1H), 7.81 (d, *J* = 16 Hz, 1H); ^{13}C NMR (CDCl$_3$), δ: 23.75, 34.19, 36.67, 51.74, 118.33, 123.17, 123.35, 125.39, 127.13, 137.85, 140.40, 152.29, 156.20, 167.64; HRESIMS calcd. for $C_{16}H_{22}N_2O_3Na$ (M+Na$^+$): 313.1528; found 313.1517.

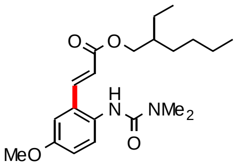

Following the general procedure above, using **1a** (48 mg, 0.25 mmol), acrylate ester (92 mg, 0.50 mmol), 1,4-benzoquinone (0.75 mmol, 81 mg), Pd(OAc)$_2$ (0.025 mmol, 5.6 mg), 48 wt % HBF$_4$ (0.25 mmol, 32 uL), and EtOAc (1 mL), yielded the product **5l** (91mg, 97%), ^{1}H NMR (CDCl$_3$) δ: 0.90 (t, *J* = 6.8 Hz, 3H), 0.91 (t, *J* = 7.5 Hz, 3H), 1.25-1.41 (m, 8H), 1.58-1.63 (m, 1H), 2.99 (s, 6H), 3.80 (s, 3H), 4.02 (dd, *J* = 6.0 and 11 Hz, 1H), 4.07 (dd, *J* = 5.6 and 11 Hz, 1H), 6.33 (d, *J* = 15.7 Hz, 1H), 6.47 (brs, 1H), 6.88 (dd, *J* = 2.6 and 8.7 Hz, 1H), 7.03 (d, *J* = 2.6 Hz, 1H), 7.29 (d, *J* = 8.7 Hz, 1H), 7.77 (d, *J* = 15.7 Hz, 1H). ^{13}C NMR (CDCl$_3$) δ: 10.95, 14.02, 22.91, 23.77, 28.89, 30.38, 36.42, 38.76, 55.45, 66.88, 110.72, 116.82, 119.42, 128.18, 130.35, 130.91, 140.08, 156.63, 157.01, 167.04; HRESIMS calcd. for $C_{21}H_{32}N_2O_4Na$ (M+Na$^+$): 399.2260; found 399.2260.

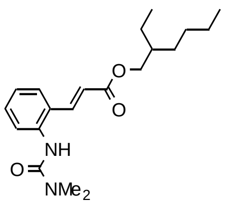

Following the general procedure above, using aryl urea **1k** (41.1 mg, 0.25 mmol), acrylate ester (43 mg, 0.5 mmol), 1,4-benzoquinone (0.75 mmol, 81 mg), $Pd(OAc)_2$ (0.025 mmol, 5.6 mg), 48 wt % HBF_4 (0.25 mmol, 32 uL), and EtOAc (1 mL), yielded the product **5m** (82 mg, 95%); ^1H NMR ($CDCl_3$) δ: 0.91 (t, J = 7.2 Hz, 3H), 0.92 (t, J = 7.6 Hz, 3H), 1.25-1.45 (m, 8H), 1.61-1.66 (m, 1H), 3.07 (s, 6H), 4.08-4.16 (m, 2H), 6.27 (br s, 1H), 6.42 (d, J = 15.6 Hz, 1H), 7.12-7.16 (m, 1H), 7.34-7.39 (m, 1H), 7.53 (d, J = 7.6 Hz, 1H), 7.67 (d, J = 8.4 Hz, 1H), 7.83 (d, J = 15.6 Hz, 1H); ^{13}C NMR ($CDCl_3$), δ: 11.56, 14.18, 23.10, 23.97, 29.07, 30.58, 36.62, 38.96, 67.10, 119.90, 124.86, 125.41, 127.05, 127.98, 130.73, 137.84, 140.03, 156.14, 167.20; HRESIMS calcd. for $C_{20}H_{30}N_2O_3Na$ ($M+Na^+$): 369.2154; found 369.2162.

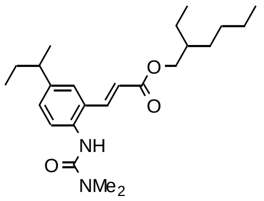

Following the general procedure above, using aryl urea **1q** (0.25 mmol), acrylate ester (0.5 mmol), 1,4-benzoquinone (0.75 mmol, 81 mg), and $Pd(OAc)_2$ (0.025 mmol, 5.6 mg), EtOAc (1.0 mL) and 48 wt % HBF_4 (0.25 mmol, 32 uL) yielded the product **5n** (99 mg, 99%); ^1H NMR ($CDCl_3$) δ: 0.81 (t, J = 7.3 Hz, 3H), 0.90 (t, J = 7.0 Hz, 6H), 1.31 (d, J = 7.0 Hz, 3H), 1.41-1.43 (m, 8H), 1.54-1.62 (m, 3H), 2.57 (sext, J = 7.0 Hz, 1H), 3.01 (s, 3H), 4.04 (dd, J = 6.0 and 11.0 Hz, 1H), 4.07 (dd, J = 5.6 and 11.0 Hz, 1H), 6.38 (d, J = 15.9 Hz, 1H), 6.51 (brs, 1H), 7.16 (dd, J = 2.0 and 8.4 Hz, 1H), 7.33 (d, J = 1.9 Hz, 1H), 7.44 (d, J = 8.4 Hz, 1H), 7.83 (d, J = 15.9 Hz, 1H). ^{13}C NMR ($CDCl_3$) δ: 10.99, 12.17, 14.03, 21.74, 22.95, 23.77, 28.91, 30.39, 31.01, 36.45, 38.79, 41.18, 66.85, 119.20, 125.24, 125.60, 127.82, 129.46, 135.45, 140.29, 144.20, 156.17, 167.16; HRESIMS calcd. for $C_{24}H_{38}N_2O_3Na$ ($M+Na^+$): 425.2780; found 425.2774.

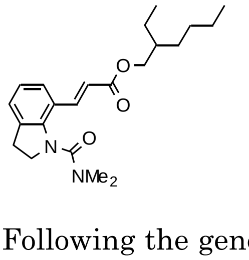

Following the general procedure above, using aryl urea **1n** (47.6 mg, 0.25 mmol), acrylate ester (43 mg, 0.5 mmol), 1,4-benzoquinone (0.75 mmol, 81 mg), $Pd(OAc)_2$ (0.025 mmol, 5.6 mg), 48

wt % HBF_4 (0.25 mmol, 32 uL), and EtOAc (1 mL), yielded the product **5o** (92.5 mg, 99%); ^{1}H NMR ($CDCl_3$) δ: 0.90-0.93 (m, 6H), 1.30-1.43 (m, 8H), 1.57-1.61 (m, 1H), 3.01 (s, 6H), 3.09 (t, *J* = 8 Hz, 2H), 3.94 (t, *J* = 8 Hz, 2H), 4.02-4.12 (m, 2H), 6.33 (d, *J* = 16 Hz, 1H), 7.00 (t, *J* = 7.6 Hz, 1H), 7.19 (d, *J* = 7.2 Hz, 1H), 7.38 (d, *J* = 7.6 Hz, 1H), 7.51 (d, *J* = 16 Hz, 1H); ^{13}C NMR ($CDCl_3$), δ: 11.15, 14.13, 23.04, 23.96, 29.05, 30.01, 30.55, 37.56, 38.93, 52.74, 66.59, 117.31, 123.62, 123.72, 125.56, 126.00, 134.30, 141.39, 144.85, 161.68, 167.39; HRESIMS calcd. for $C_{22}H_{32}N_2O_3Na$ (M+Na$^+$): 395.2311; found 395.2305.

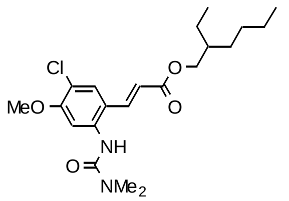

Following the general procedure above, using aryl urea **1o** (57.2 mg, 0.25 mmol), acrylate ester (43 mg, 0.5 mmol), 1,4-benzoquinone (0.75 mmol, 81 mg), $Pd(OAc)_2$ (0.025 mmol, 5.6 mg), 48 wt % HBF_4 (0.25 mmol, 32 uL), and EtOAc (1 mL), yielded the product **5p** (91.2 mg, 89%); ^{1}H NMR (acetone-d_6) δ: 0.88-0.94 (m, 6H), 1.31-1.42 (m, 8H), 1.61-1.65 (m, 1H), 3.08 (s, 6H), 3.93 (s, 3H), 4.08-4.14 (m, 2H), 6.32 (d, *J* = 16 Hz, 1H), 6.43 (brs, 1H), 7.52 (s, 1H), 7.59 (s, 1H), 7.68 (d, *J* = 16 Hz, 1H); ^{13}C NMR (acetone-d_6), δ: 11.14, 14.19, 23.11, 23.95, 29.07, 30.57, 36.66, 38.97, 56.41, 67.16, 107.91, 118.45, 118.51, 119.74, 128.01, 137.97, 155.64, 156.65, 167.24; HRESIMS calcd. for $C_{21}H_{31}N_2O_4NaCl$ (M+Na$^+$): 433.1870; found 433.1870.

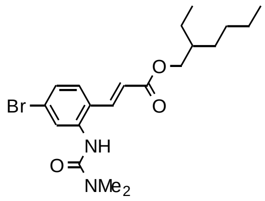

Following the general procedure above, using aryl urea **1p** (61 mg, 0.25 mmol), acrylate ester (43 mg, 0.5 mmol), 1,4-benzoquinone (0.75 mmol, 81 mg), $Pd(OAc)_2$ (0.025 mmol, 5.6 mg), 48 wt % HBF_4 (0.25 mmol, 32 uL), and EtOAc (1 mL), yielded the product **5q** (88.7 mg, 83%); ^{1}H NMR (acetone-d_6) δ: 0.88-0.94 (m, 6H), 1.30-1.42 (m, 8H), 1.61-1.65 (m, 1H), 3.06 (s, 6H), 4.08-4.14 (m, 2H), 6.33 (brs, 1H), 6.39 (d, *J* = 16 Hz, 1H), 7.24 (dd, *J* = 1.6 and 8.4 Hz, 1H), 7.36 (d, *J* = 8.4 Hz, 1H), 7.71 (d, *J* = 16 Hz, 1H), 7.95 (d, *J* = 1.6 Hz, 1H); ^{13}C NMR (acetone-d_6), δ: 11.17, 14.21, 23.12, 23.97, 29.09, 30.58, 36.65, 38.96, 67.26, 120.38, 124.51, 126.41, 127.80, 127.85, 128.17, 138.83, 138.92, 155.65, 166.99; HRESIMS calcd. for $C_{20}H_{29}N_2O_3NaBr$ (M+Na$^+$): 447.1259; found 427.1255.

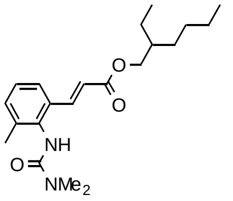

Following the general procedure above, using aryl urea **1l** (44.6 mg, 0.25 mmol), acrylate ester (43 mg, 0.5 mmol), 1,4-benzoquinone (0.75 mmol, 81 mg), $Pd(OAc)_2$ (0.025 mmol, 5.6 mg), 48 wt % HBF_4 (0.25 mmol, 32 uL), and EtOAc (1 mL), yielded the product **5r** (78.3 mg, 87%); ^1H NMR ($CDCl_3$) δ: 0.89-0.94 (m, 6H), 1.31-1.43 (m, 8H), 1.61-1.64 (m, 1H), 3.07 (s, 6H), 4.06-4.14 (m, 2H), 5.85 (brs, 1H), 6.40 (d, *J*= 16.5 Hz, 1H), 7.18 (t, *J*= 7.5 Hz, 1H), 7.26 (d, *J*= 7.5 Hz, 1H), 7.50 (d, *J*= 7.5 Hz, 1H), 7.88 (d, *J*= 16.5 Hz, 1H); ^{13}C NMR ($CDCl_3$), δ: 11.15, 14.18, 18.37, 23.09, 23.96, 29.06, 30.55, 36.64, 38.94, 66.89, 119.16, 124.45, 126.76, 132.38, 132.58, 136.41, 136.77, 141.27, 156.60, 167.45; HRESIMS calcd. for $C_{21}H_{32}N_2O_3Na$ ($M+Na^+$): 383.2311; found 383.2309.

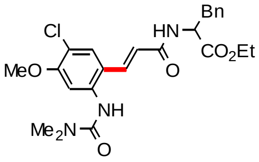

Following the general procedure above, using aryl urea **1o** (57.2 mg, 0.25 mmol), acrylamide (123 mg, 0.5 mmol), 1,4-benzoquinone (0.75 mmol, 81 mg), $Pd(OAc)_2$ (0.025 mmol, 5.6 mg), 48 wt % HBF_4 (0.25 mmol, 32 uL), and EtOAc (1 mL), yielded the product **5s** (84 mg, 71%); ^1H NMR ($CDCl_3$) δ: 1.22 (t, *J*= 7.1 Hz, 3H), 3.02 (s, 6H), 3.05-3.08 (m, 2H), 3.86 (s, 3H),4.14 (q, *J*= 7.1 Hz, 2H), 4.70-4.76 (m, 1H), 6.11 (dd, *J*= 8.6 and 15.3 Hz, 1H), 6.51 (dd, *J*= 7.8 and 11.8 Hz, 1H), 7.09-7.14 (m, 3H), 7.20-7.28 (m, 3H), 7.35 (d, *J*= 6.5 Hz, 1H), 7.59 (d, *J*= 15.3 Hz, 1H); ^{13}C NMR ($CDCl_3$), δ: 14.31, 36.77, 38.18, 53.73, 56.48, 61.84, 108.30, 108.31, 108.32, 118.22, 120.02, 127.35, 127.59, 128.78, 129.52, 135.35, 136.12, 138.14, 155.95, 156.33, 165.43, 171.78, 171.80; HRESIMS calcd. for $C_{24}H_{28}N_3O_5ClNa$ ($M+Na^+$): 496.1615; found 496.1620.

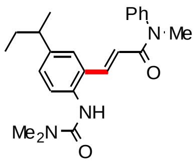

Following the general procedure above, using aryl urea **1q** (0.25 mmol), acrylamide (81 mg, 0.5 mmol), 1,4-benzoquinone (0.75 mmol, 81 mg), and $Pd(OAc)_2$ (0.025 mmol, 5.6 mg), EtOAc (1.0 mL) and 48 wt % HBF_4 (0.25 mmol, 32 uL) yielded the product **5t** (49 mg, 52%); ^1H NMR ($CDCl_3$) δ: 0.73 (t, *J*= 7.4 Hz, 3H), 1.10 (d, *J*= 7.4 Hz, 3H), 1.43-1.50 (m, 2H), 2.40-2.49 (m, 1H), 3.03 (s, 6H), 3.31 (s ,3H), 6.26 (d, *J*= 15.3 Hz, 1H), 6.59 (brs, 1H), 6.91 (brs, 1H), 7.10 (dd, *J*= 2.0 and 8.4 Hz, 1H), 7.17 (dd, *J*= 1.5 and 6.9 Hz, 2H), 7.34-7.44 (m, 3H), 7.54 (d, *J*= 8.4 Hz, 1H), 7.83 (d, *J*=

15.3 Hz, 1H). ^{13}C NMR ($CDCl_3$) δ: 12.33, 21.88, 31.15, 36.76, 37.63, 41.20, 120.31, 125.36, 125.61, 127.47, 127.82, 127.92, 128.93, 129.78, 135.62, 137.66, 143.63, 143.73, 156.32, 166.28; HRESIMS calcd. for $C_{23}H_{29}N_3O_2Na$ ($M+Na^+$): 402.2157; found 402.2159.

V. The synthesis of boscalid

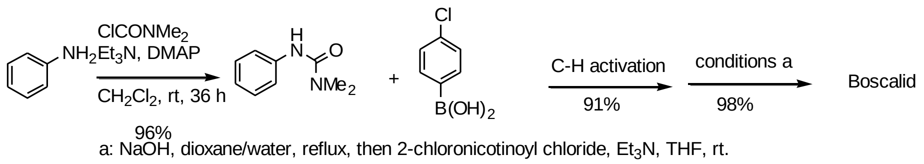

a: NaOH, dioxane/water, reflux, then 2-chloronicotinoyl chloride, Et_3N, THF, rt.

C–H Suzuki–Miyaura (1st step):

Following the general procedure **C**, using arylurea (82 mg, 0.5 mmol), *p*-ClPhB$(OH)_2$ (156 mg, 1.0 mmol), BQ (1.5 mmol, 162 mg), and $[Pd(MeCN)_4](BF_4)_2$ (0.05 mmol, 22 mg), EtOAc (2.0 mL), yielded the product (125 mg, 91%); ^1H NMR ($CDCl_3$) δ: 2.83 (s, 6H), 6.35 (brs, 1H), 7.09 (dt, *J* = 1.2 and 7.5 Hz, 1H), 7.16 (dd, *J* = 1.5 and 7.6 Hz, 1H), 7.32-7.37 (m, 3H), 7.45 (d, *J* = 8.4 Hz, 2H), 7.45 (dd, *J* = 1.2 and 8.4 Hz, 1H). ^{13}C NMR ($CDCl_3$) δ: 36.22, 121.21, 122.95, 128.71, 129.19, 129.61, 130.52, 130.62, 133.83, 136.09, 137.13, 155.42; HRESIMS calcd. for $C_{15}H_{15}N_2ONa$ ($M+Na^+$): 297.0771; found 297.0773.

Deprotection and 2-chloronicotinoylation (2nd step):

The resulting product (119 mg, 0.43 mmol) from 1st step shown in above was mixed with KOH (364 mg, 6.5 mmol) in 1,4-dioxane/water (1.6 mL/0.8 mL). After stiring under reflux conditions for 20 h, the solution obtained was filtered through the plug of silica gel and anhydrous $MgSO_4$. After evaporation to remove solvents, the crude oil, 2-chloronicotinoyl chloride and (122 mg, 0.69 mmol) were sequentially added under air to a reaction tube equipped with a stir bar and a septum. THF (5 mL), and Et_3N (1.39 mmol, 0.19 mL) was added by syringe and the resulting mixture vigorously stirred for 2 h at ambient temperature. After this time, the contents of the flask were quenched with aqueous K_2CO_3 and extracted with EtOAc. The solution obtained was filtered through the plug of silica gel and anhydrous $MgSO_4$, and then concentrated by rotary evaporation. The residue was purified by flash chromatography, eluting with hexane/EtOAc to afford Boscalid (145 mg, 98%); ^1H NMR ($CDCl_3$) δ: 7.27 (brs, 1H), 7.33-7.37 (m, 4H), 7.42-7.48 (m, 3H), 8.14 (dd, *J* = 1.9 and 7.8 Hz, 2H), 8.41 (d, *J* = 8.2 Hz, 1H), 8.45 (dd, *J* = 1.9 and 4.7 Hz, 1H). ^{13}C NMR ($CDCl_3$) δ: 122.55, 122.91, 125.56, 128.91, 129.27, 130.33, 130.86, 131.25, 132.61, 134.28, 134.35, 136.39, 139.89, 146.73, 151.21, 162.75; HRESIMS calcd. for $C_{18}H_{12}N_2ONa$ ($M+Na^+$): 365.0224; found 365.0216.

VI. Mechanistic studies

Palladacycle 6

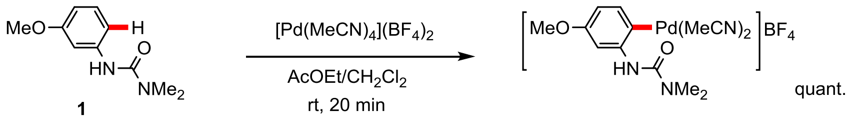

Arylurea **1** (0.1 or 0.11 mmol), and $[Pd(MeCN)_4](BF_4)_2$ (0.1 mmol) were sequentially added under Ar to a reaction tube equipped with a stir bar and a septum. EtOAc (6 mL, or EtOAc/CH_2Cl_2) was added by syringe and the resulting mixture stirred for 0.5 h at ambient temperature. To complete the reaction, we heat the reaction mixture at 40 °C for 0.5 h. The resulting crystals were filtered off and washed with EtOAc and Et_2O followed by dried under vacuum to give yellow crystals. Single crystals were obtained by the recrystalization from MeCN/toluene.; ^1H NMR (acetone-d_6) δ: 2.61 (s, 3H), 3.12 (brs, 9H), 3.72 (s, 3H), 6.42 (dd, J = 2.8 and 8.7 Hz, 1H), 6.67 (d, J = 2.8 Hz, 1H), 6.97 (d, J = 8.7 Hz, 1H), 8.74 (brs, 1H). ^{13}C NMR (acetone-d_6) δ: 2.46, 36.94, 54.87, 102.37, 104.30, 109.16, 122.59, 135.50, 135.59, 136.63, 154.72, 158.97. ^{19}F NMR (acetone-d_6) δ: −88.768. ^{11}B NMR (acetone-d_6) δ: 4.362; ESI/TOF $C_{14}H_{19}BF_4N_4O_2Pd$ (M^+−BF_4): 381.06.

palladacycle from $Pd(OAc)_2$

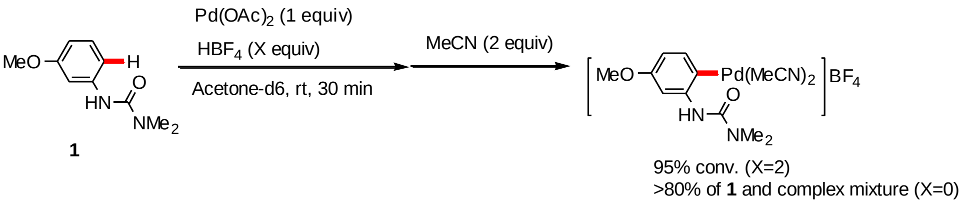

Arylurea **1** (0.05 mmol), and $Pd(OAc)_2$ (0.05 mmol) were sequentially added under Ar to a reaction tube equipped with a stir bar and a septum. Acetone-d_6 (1.0 mL), and 48 wt % HBF_4 (0.1 mmol) was added by syringe and the resulting mixture vigorously stirred for 0.5 h at ambient temperature. After this time, MeCN (0.1 mmol) was added and ^1H NMR was carried out to check the structure. The spectrum of the product was matched with the palladacycle shown in above. On the other hand, the reaction without HBF_4 gave no product.

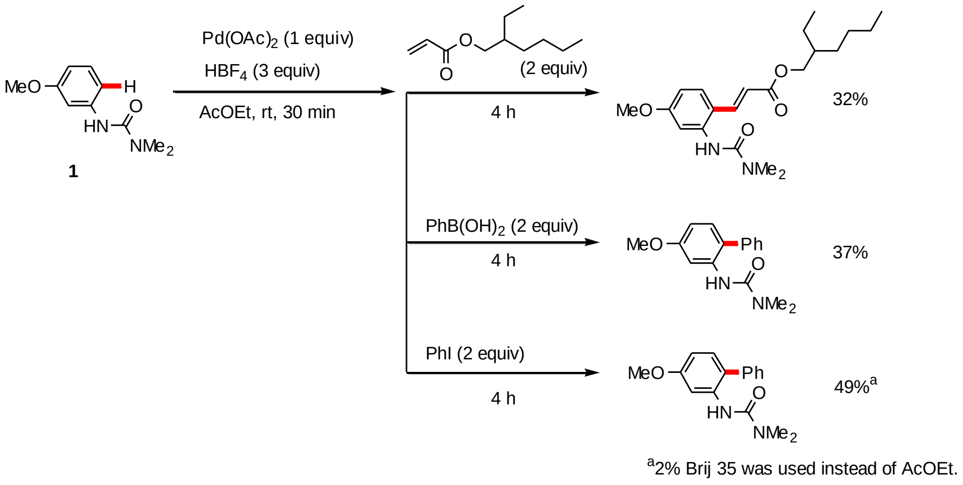

Arylurea **1** (0.05 mmol), and $Pd(OAc)_2$ (0.05 mmol) were sequentially added under Ar to a reaction tube equipped with a stir bar and a septum. EtOAc (1.0 mL), and 48 wt % HBF_4 (0.15 mmol) was added by syringe and the resulting mixture vigorously stirred for 0.5 h at ambient temperature. After this time, acrylate ester, phenylboronic acid, or iodobenzene (0.1 mmol) was added. After stirring 4 h, the contents of the flask were quenched with aqueous K_2CO_3 and extracted with EtOAc. The solution obtained was filtered through the plug of silica gel and anhydrous $MgSO_4$, and then concentrated by rotary evaporation and dried under vacuum. ^1H NMR was carried out to check the yields.

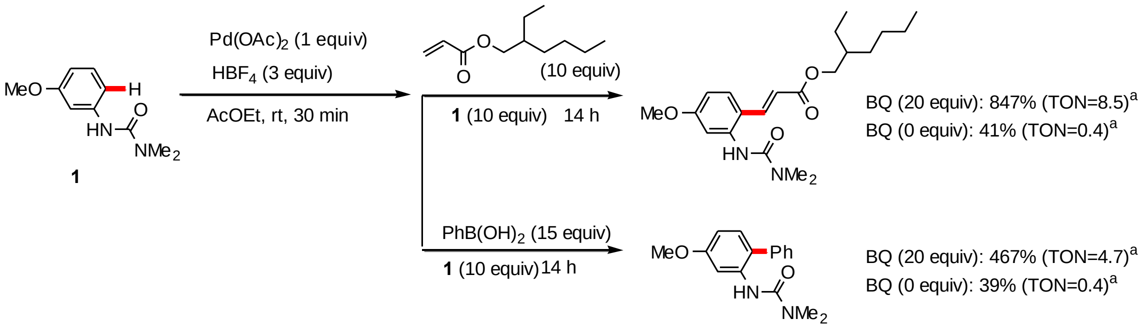

[a]Yields were based on Pd.

Arylurea **1** (0.05 mmol), and $Pd(OAc)_2$ (0.05 mmol) were sequentially added under Ar to a reaction tube equipped with a stir bar and a septum. EtOAc (1.0 mL), and 48 wt % HBF_4 (0.15 mmol) was added by syringe and the resulting mixture vigorously stirred for 0.5 h at ambient temperature. After this time, acrylate ester, or phenylboronic acid (0.5 mmol) and BQ (0 or 1 mmol) and **1** (0.5 mmol) were added. After stirring 14 h, the contents of the flask were quenched with aqueous K_2CO_3 and extracted with EtOAc. The solution obtained was filtered through the plug of silica gel and anhydrous $MgSO_4$, and then concentrated by rotary evaporation and dried under vacuum. ^1H NMR was carried out to check the yields.

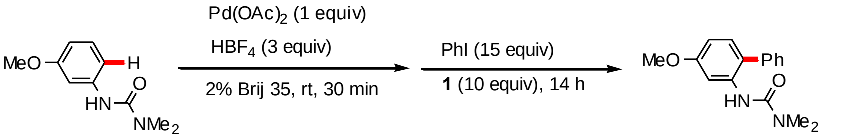

AgOAc (equiv)	HBF_4 (equiv)	yield[a]	TON
15	50	342	3.4
0	50	87	0.9
15	0	0	0
0	0	94	0.9

[a]Yields were based on Pd.

Arylurea **1** (0.05 mmol), and $Pd(OAc)_2$ (0.05 mmol) were sequentially added under Ar to a reaction tube equipped with a stir bar and a septum. EtOAc (1.0 mL), and 48 wt % HBF_4 (0.15 mmol) was added by syringe and the resulting mixture vigorously stirred for 0.5 h at ambient temperature. After this time, iodobenzene (0.75 mmol), **1** (0.5 mmol), HBF_4 (0 or 2.5 mmol) and

AgOAc (0 or 0.75 mmol) were added. After stirring 14 h, the contents of the flask were quenched with aqueous K_2CO_3 and extracted with EtOAc. The solution obtained was filtered through the plug of silica gel and anhydrous $MgSO_4$, and then concentrated by rotary evaporation and dried under vacuum. ^{1}H NMR was carried out to check the yields.

Reference

1. Houlden, C. E.; Bailey, C. D.; Ford, J. G.; Gagné, M. R.; Lloyd-Jones, G. C.; Booker-Milburn, K. I. *J. Am. Chem. Soc.* **2008**, *130*, 10066-10067.
2. Kathiravan, S.; Nicholls, I. A. *Chem. Commun.*, **2014**, *50*, 14964-14967.
3. Nishikata, T.; Abela, A. R.; Lipshutz, B. H. *Angew. Chem., Int. Ed.* **2010**, *49*, 781-784.
4. Nishikata, T.; Abela, A. R.; Huanf, S.; Lipshutz, B. H. *J. Am. Chem. Soc.* **2010**, *132*, 4978-4979.
5. Nishikata, T.; Lipshutz, B. H. *Org. Lett.* **2010**, *12*, 1972-1975

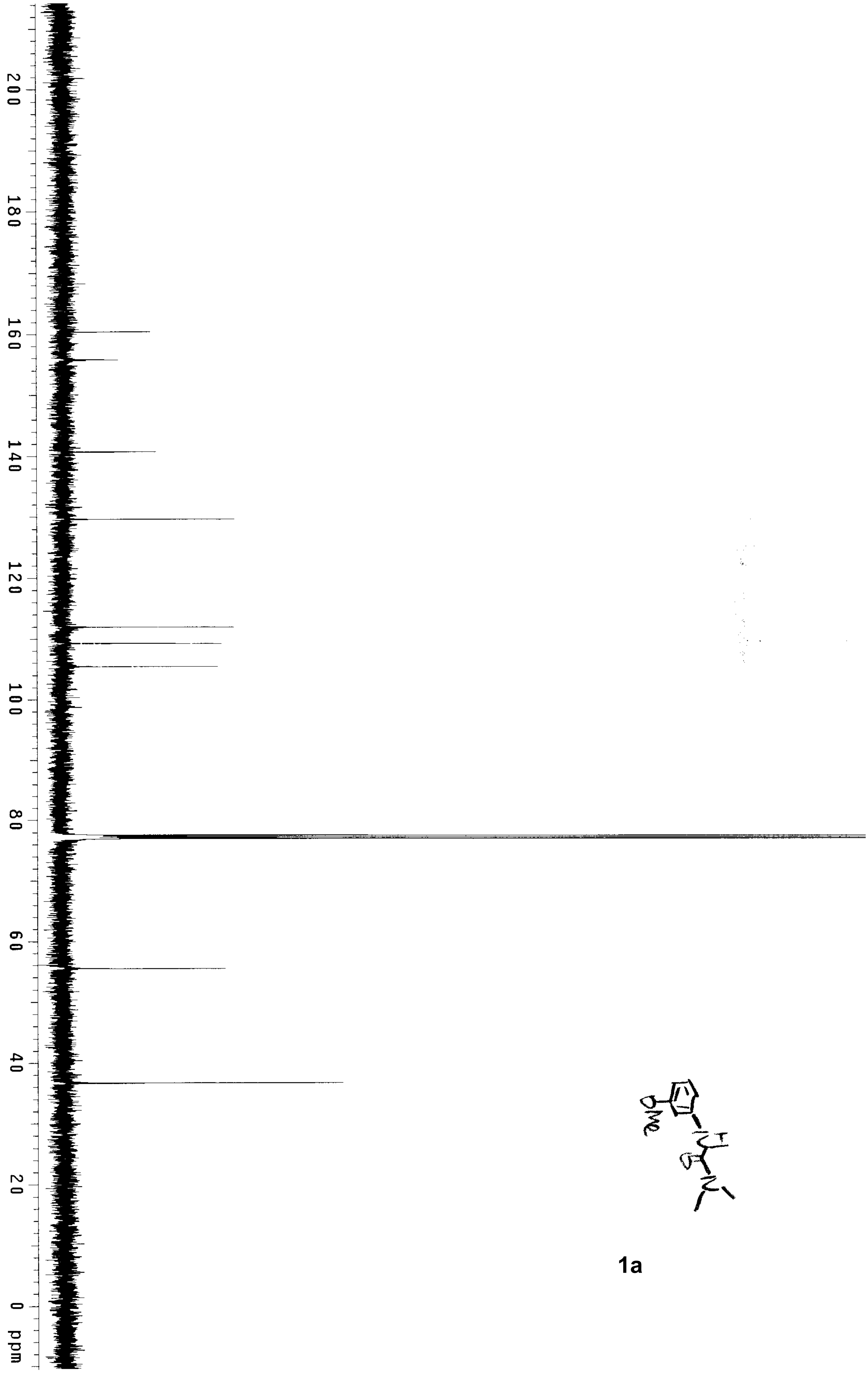

1a

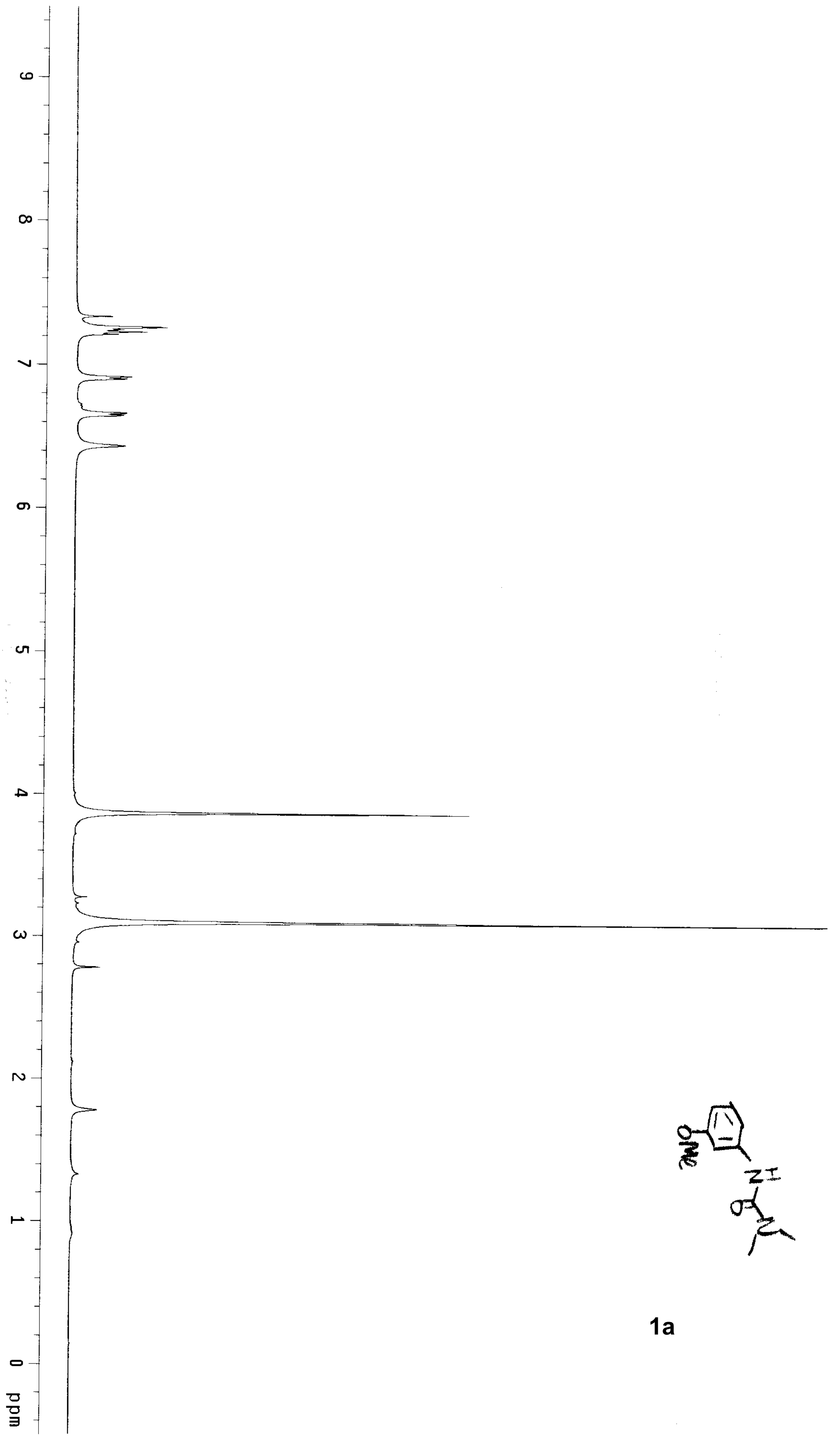

1a

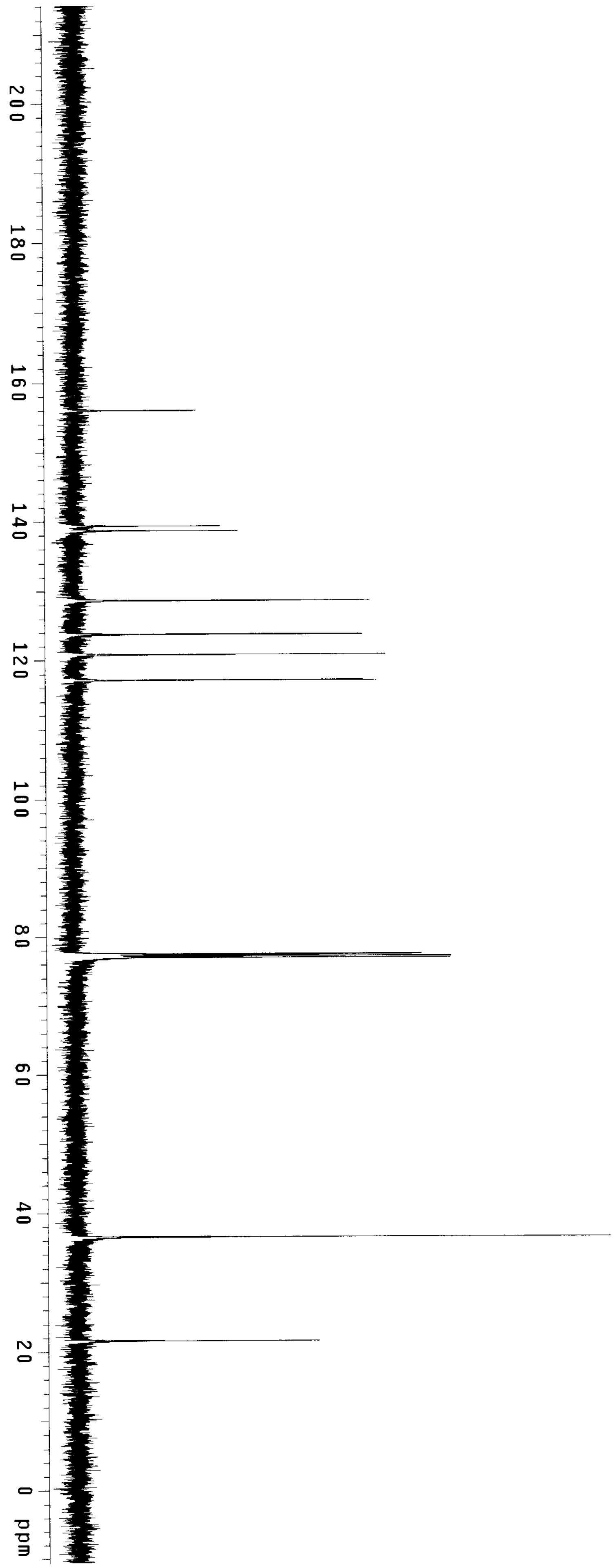

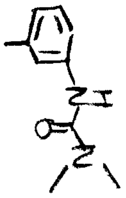

1b

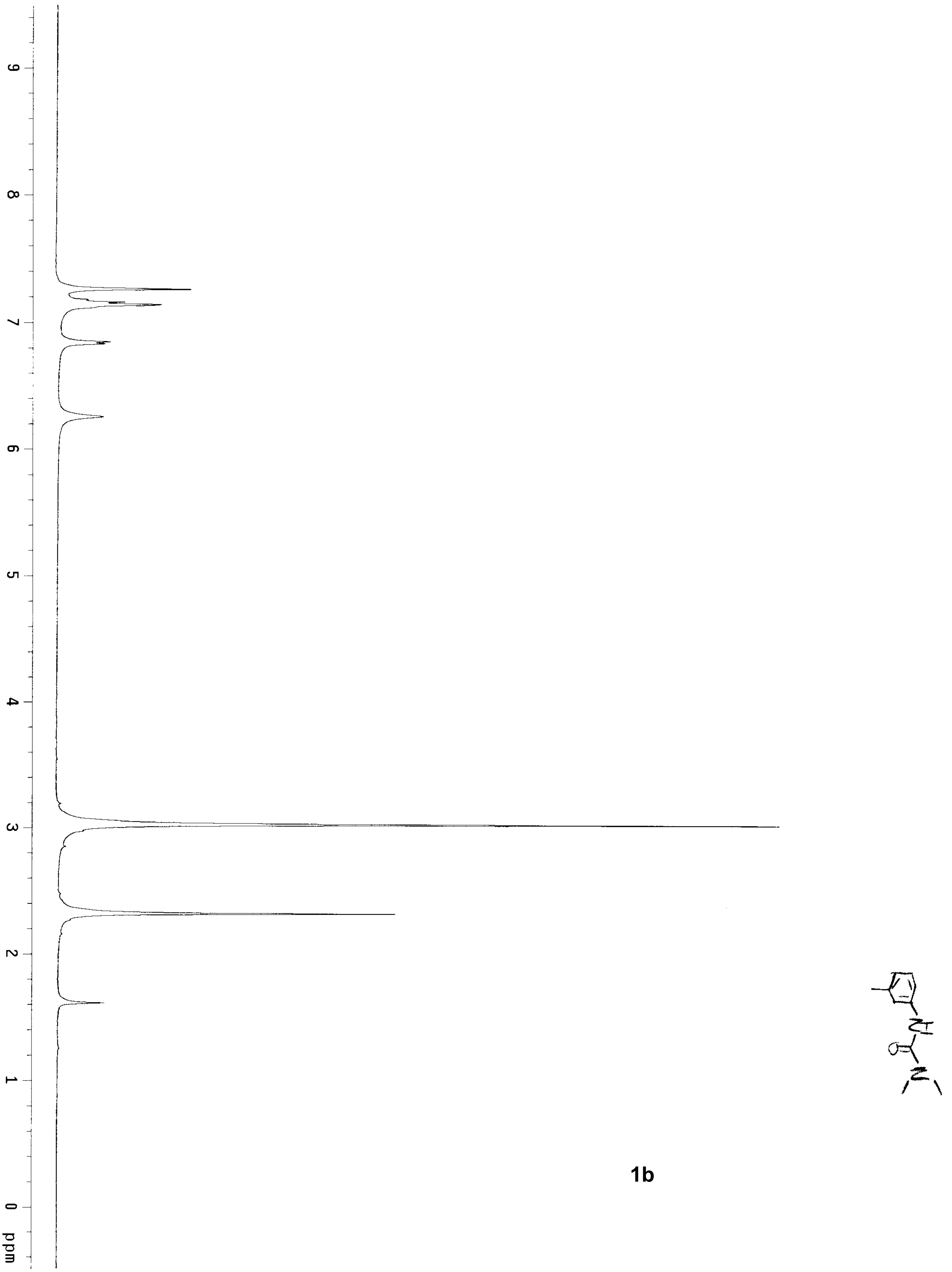

1b

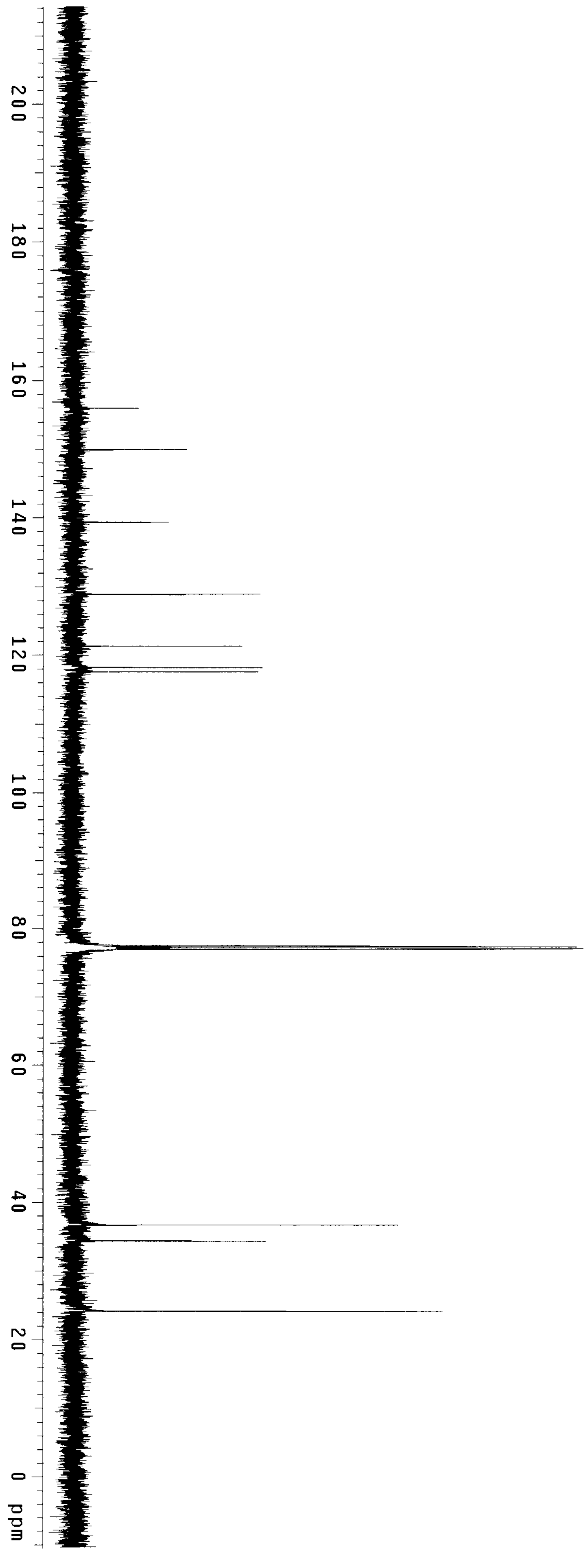

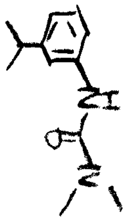

1c

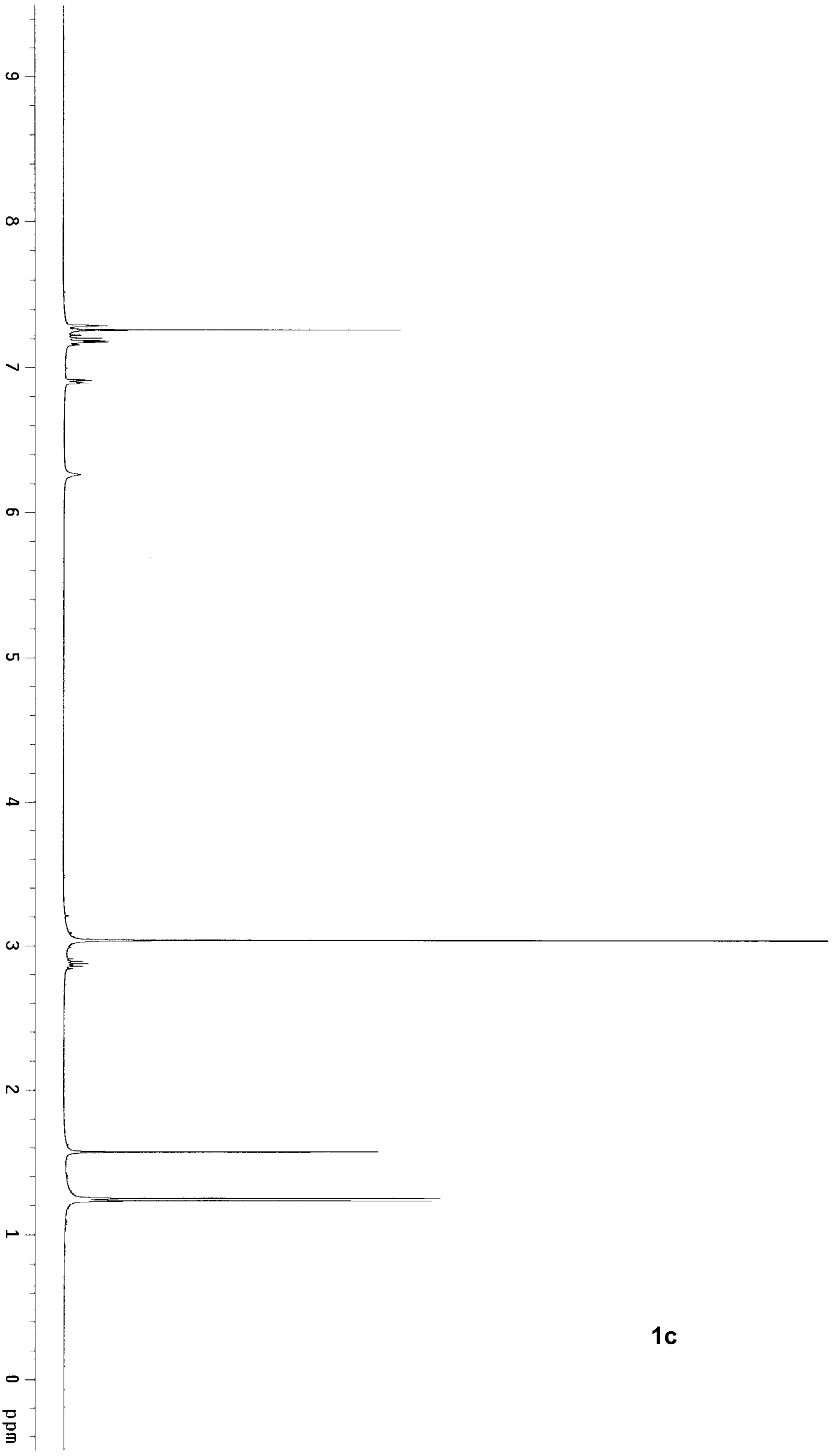

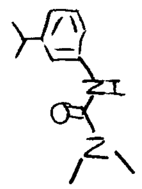

1c

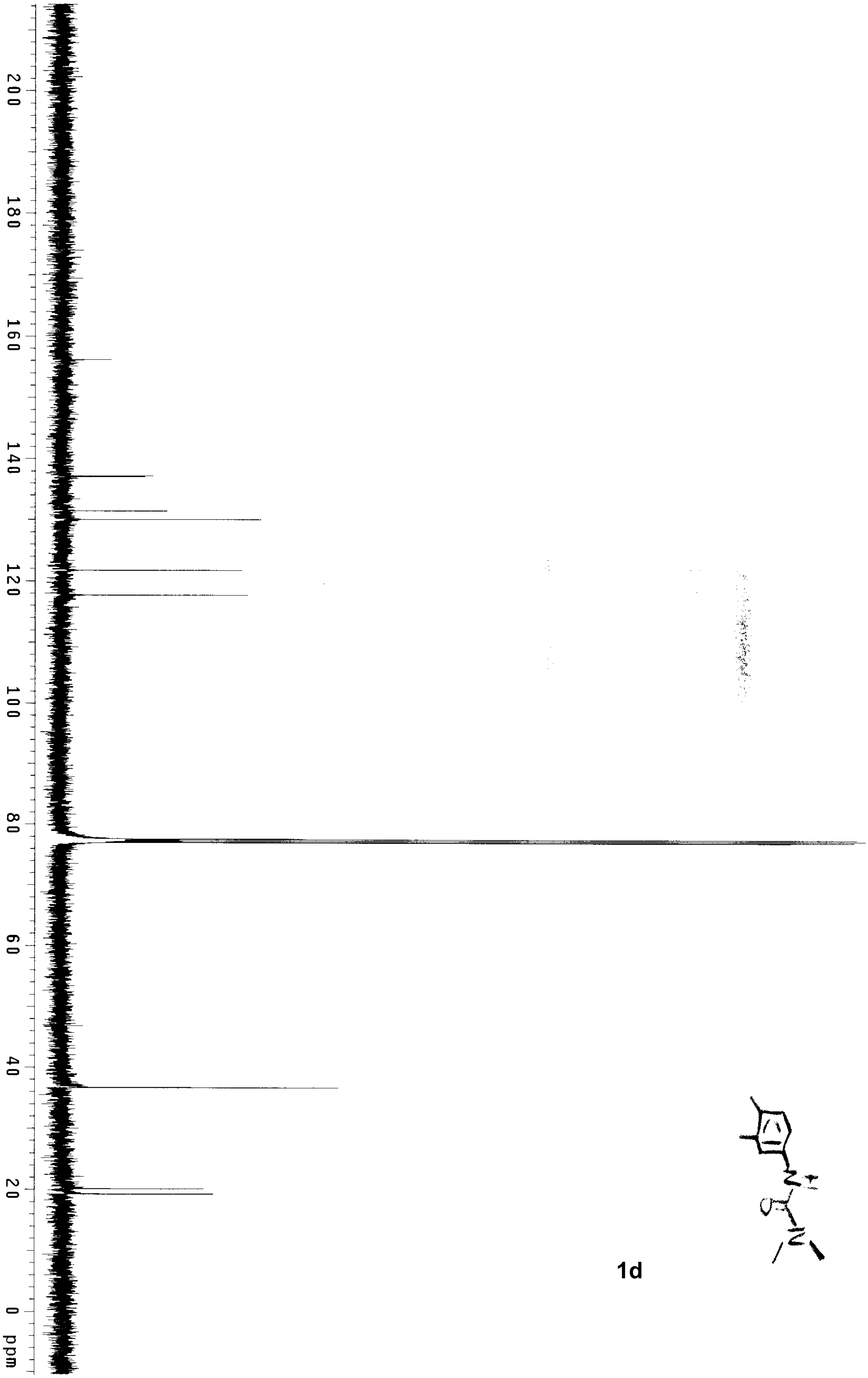

1d

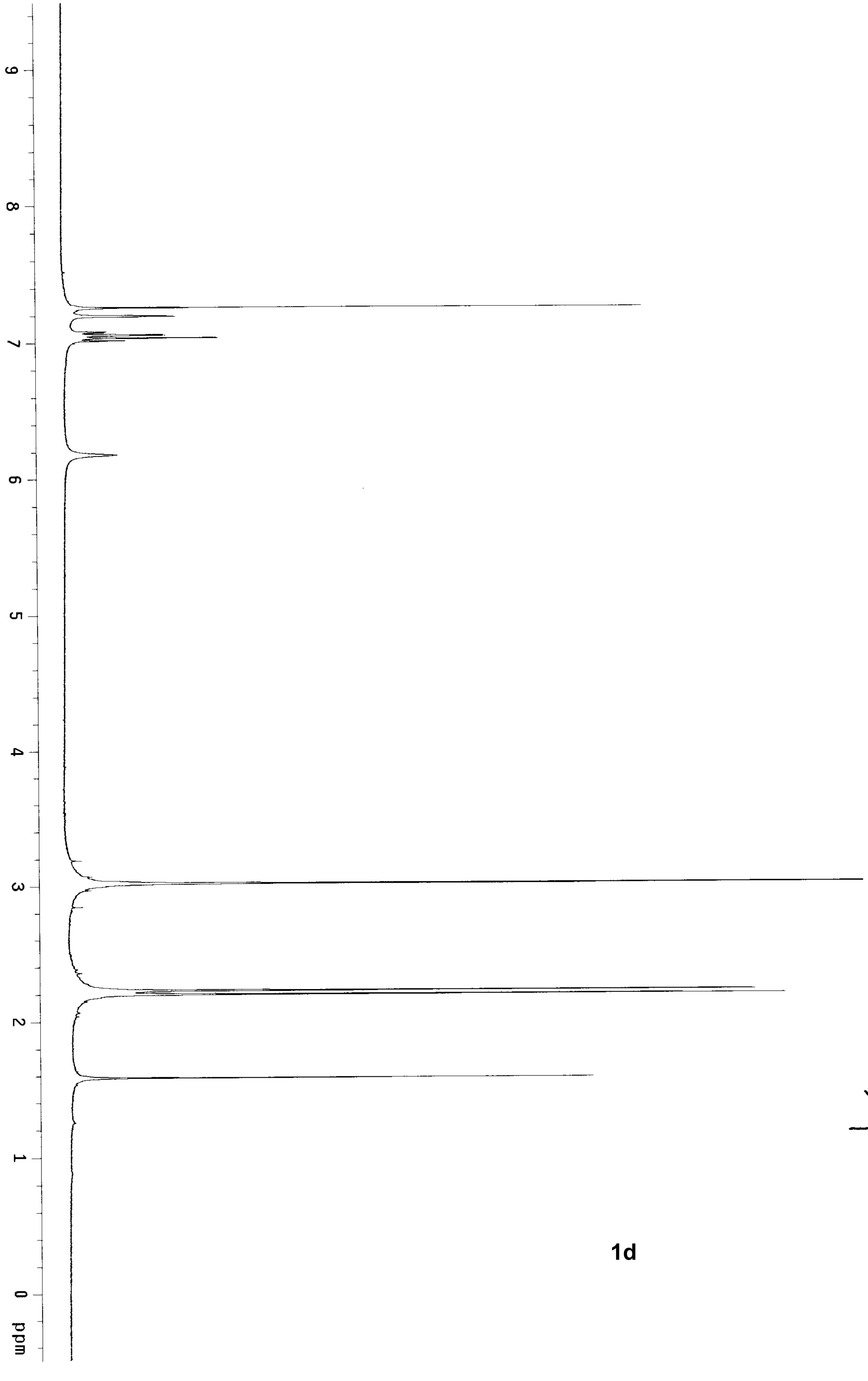

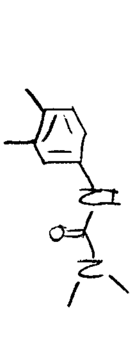

1d

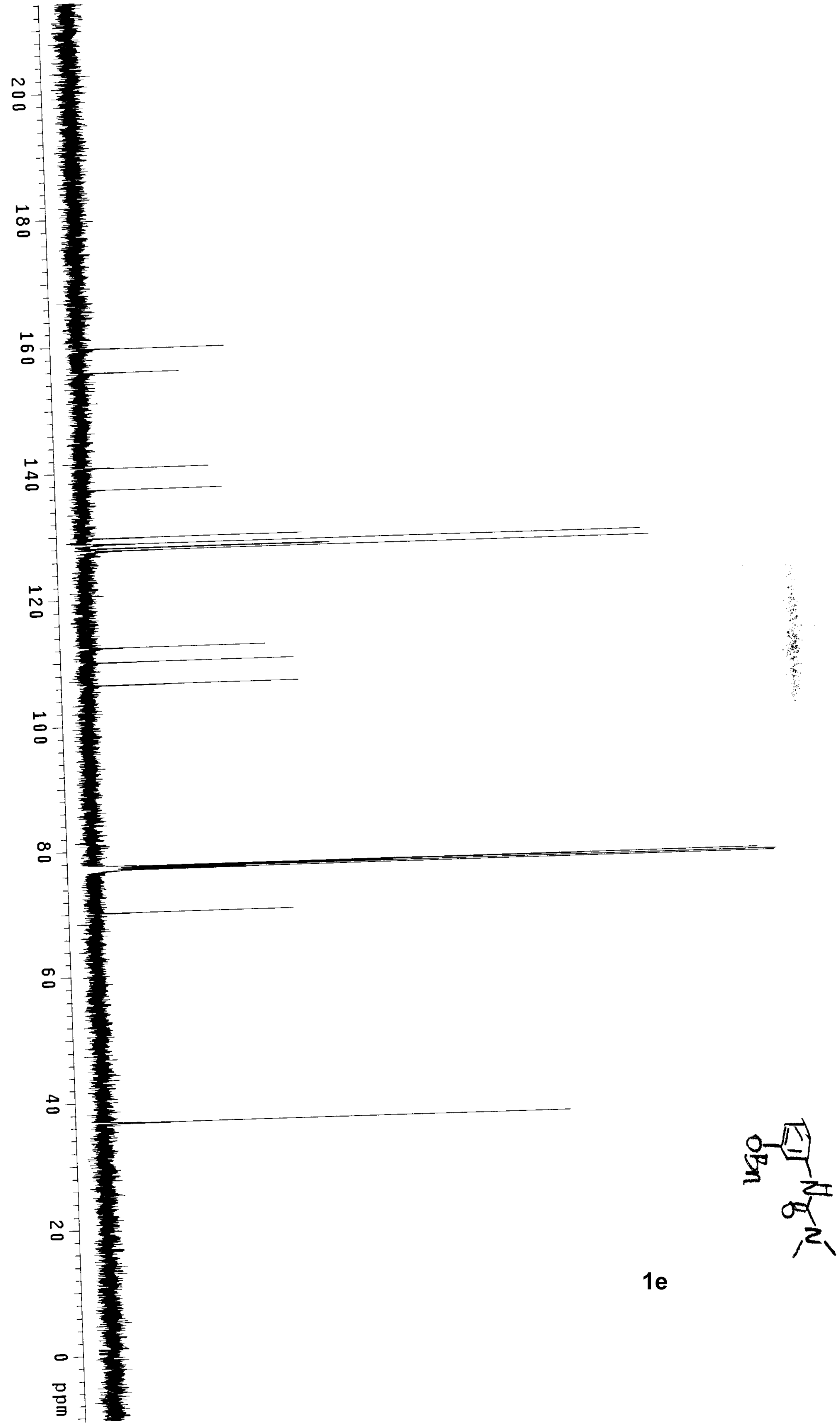

1e

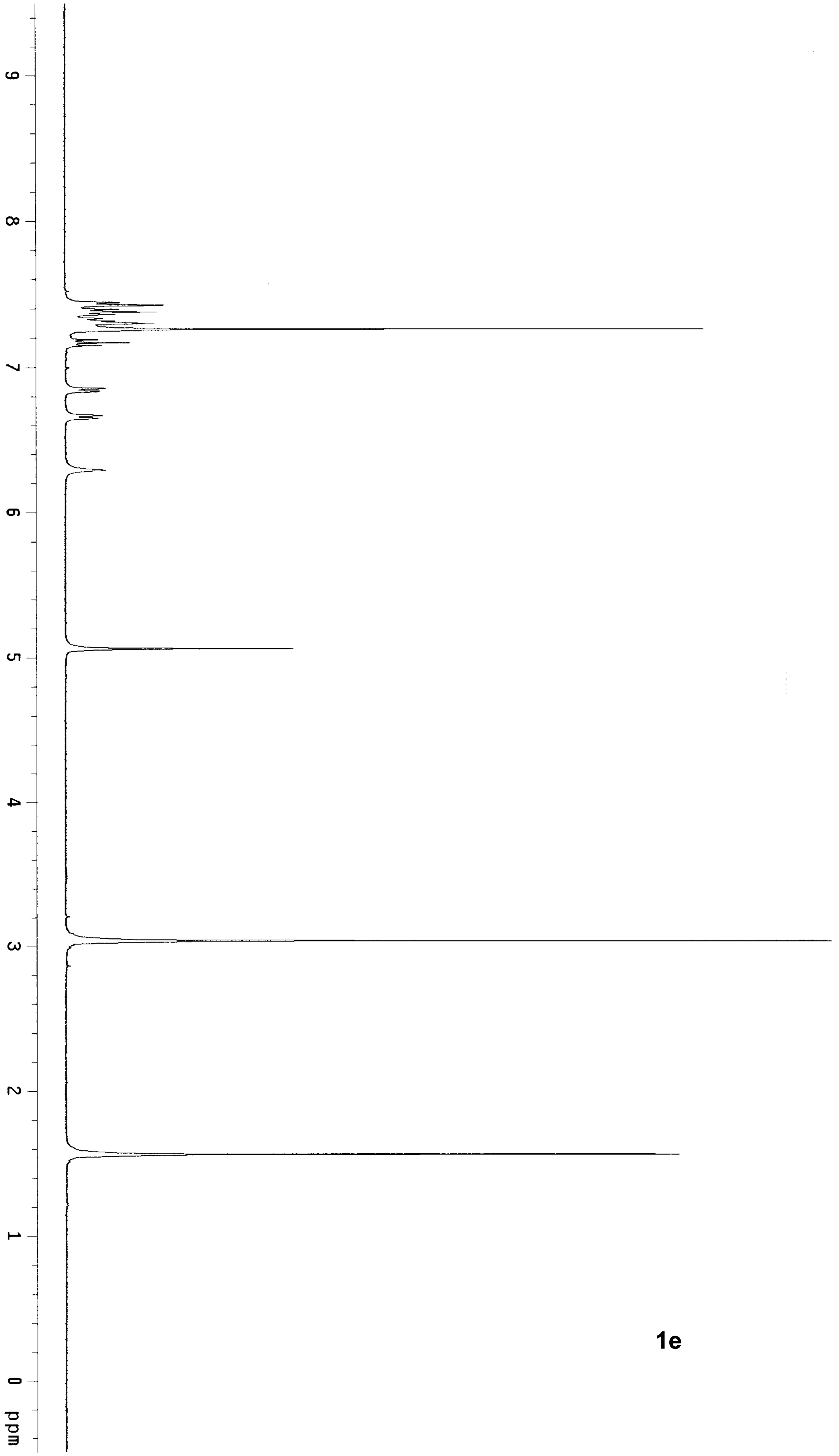

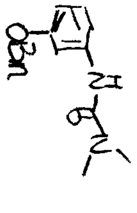

1e

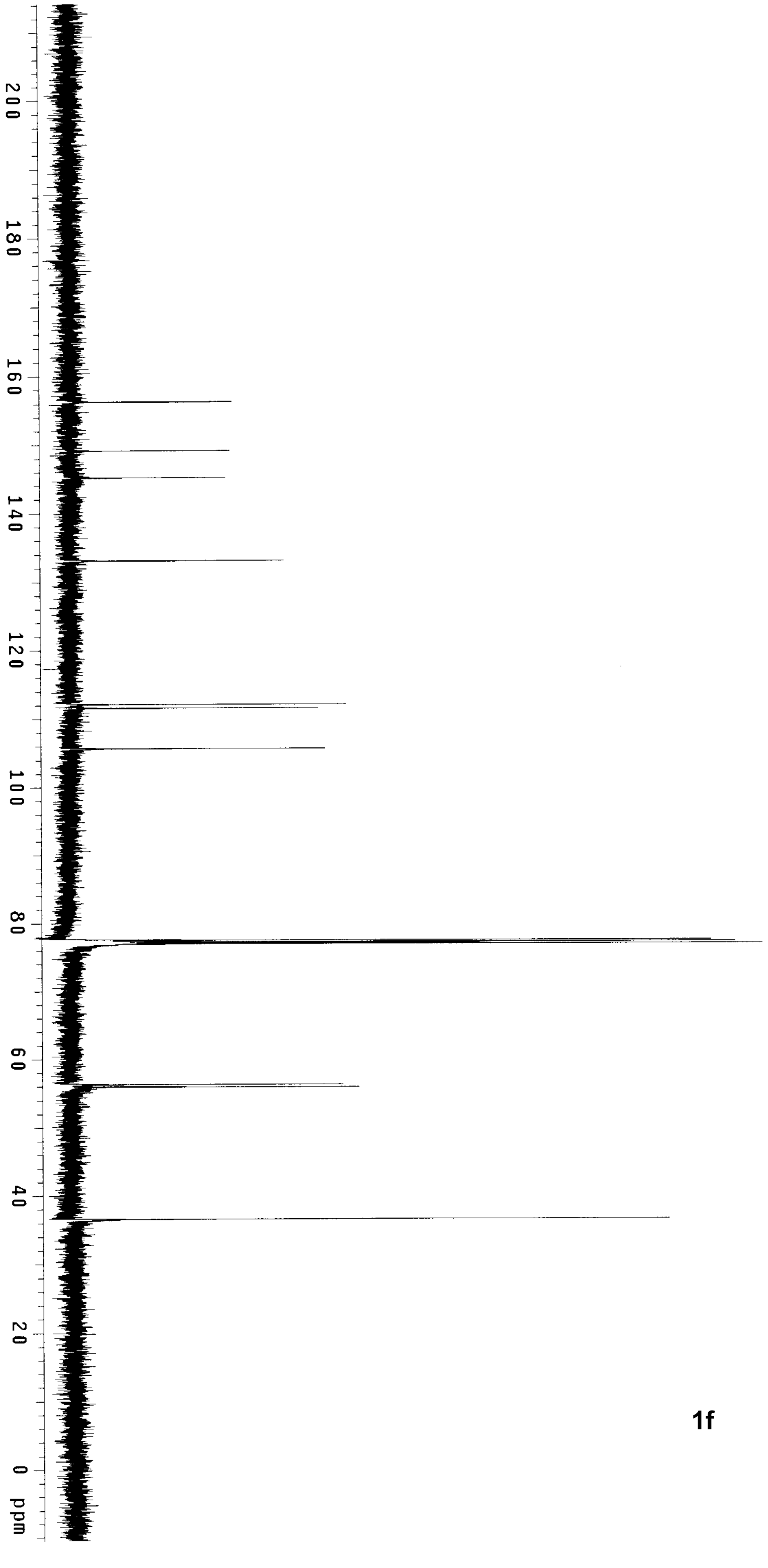

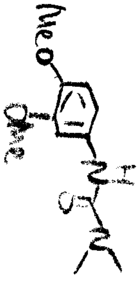

1f

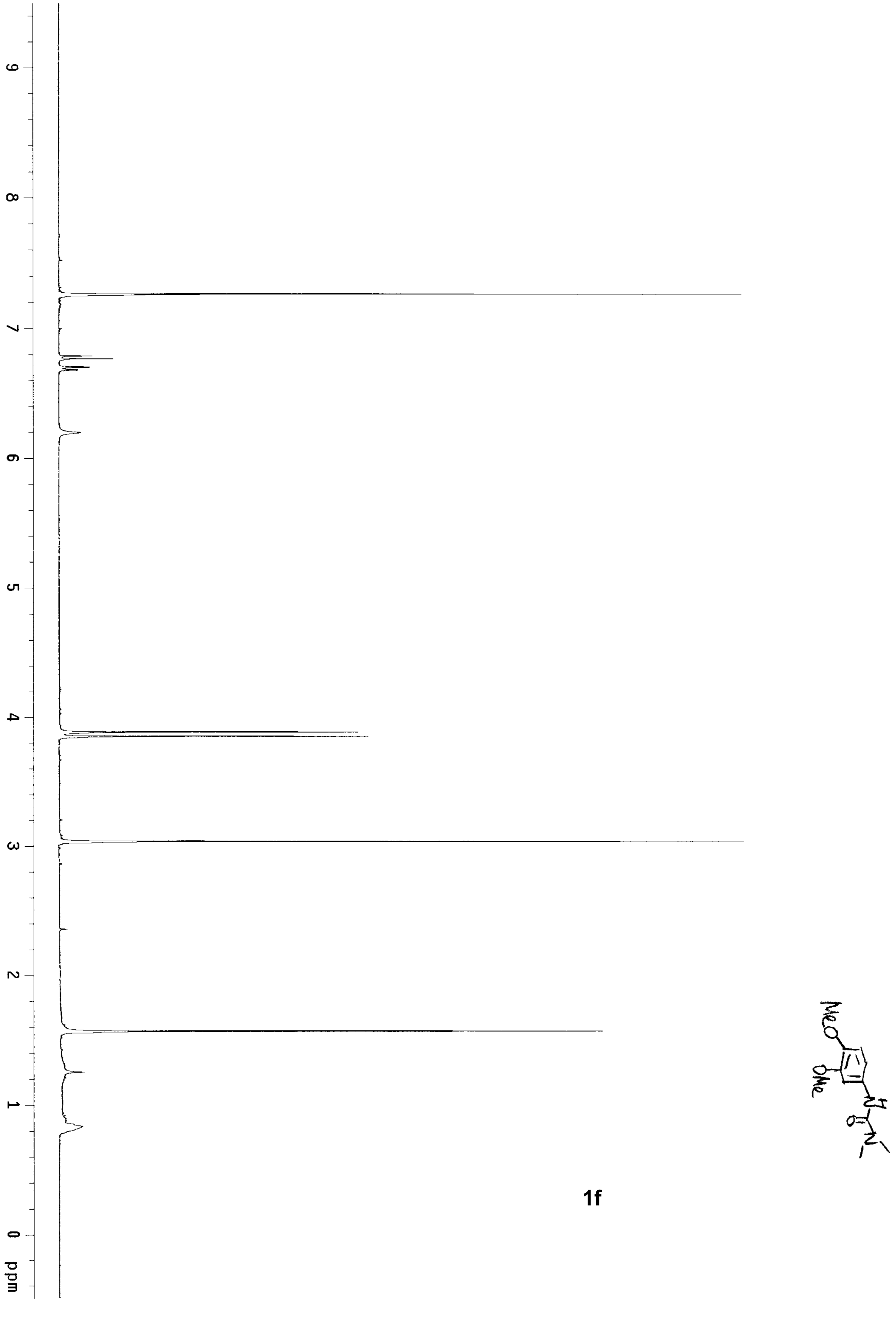

1f

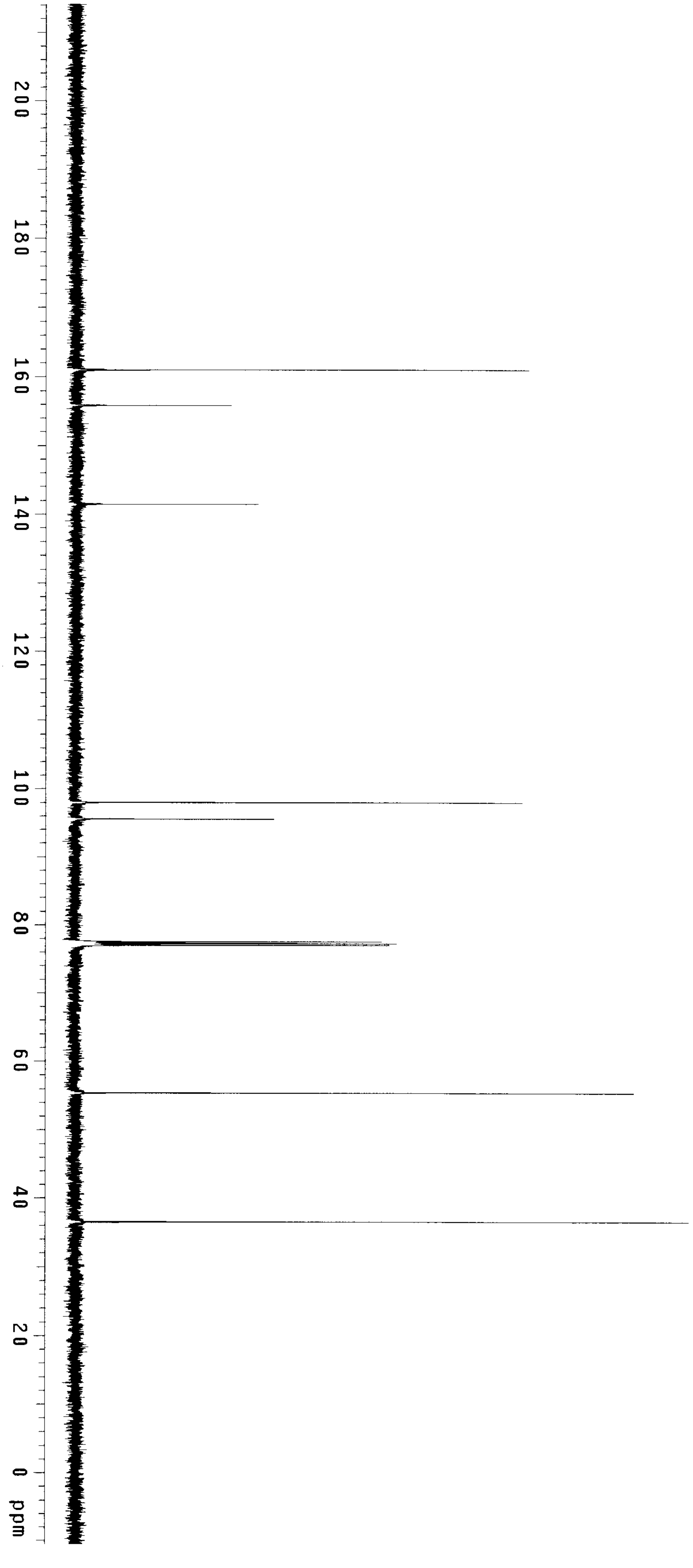

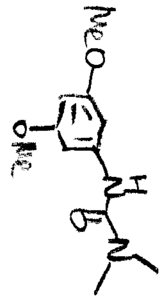

1g

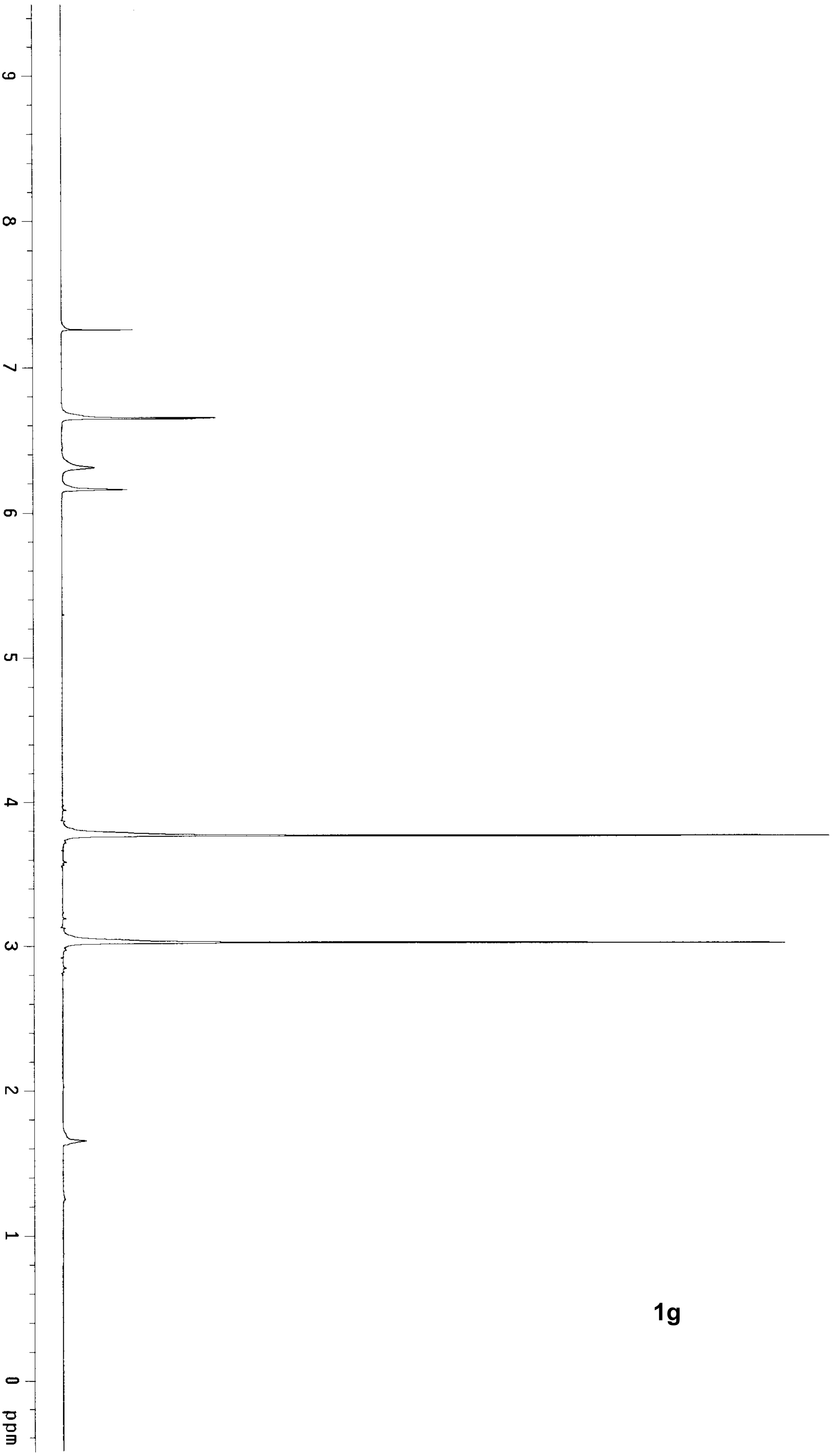

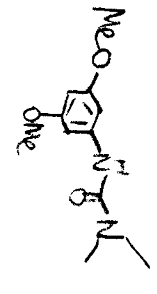

1g

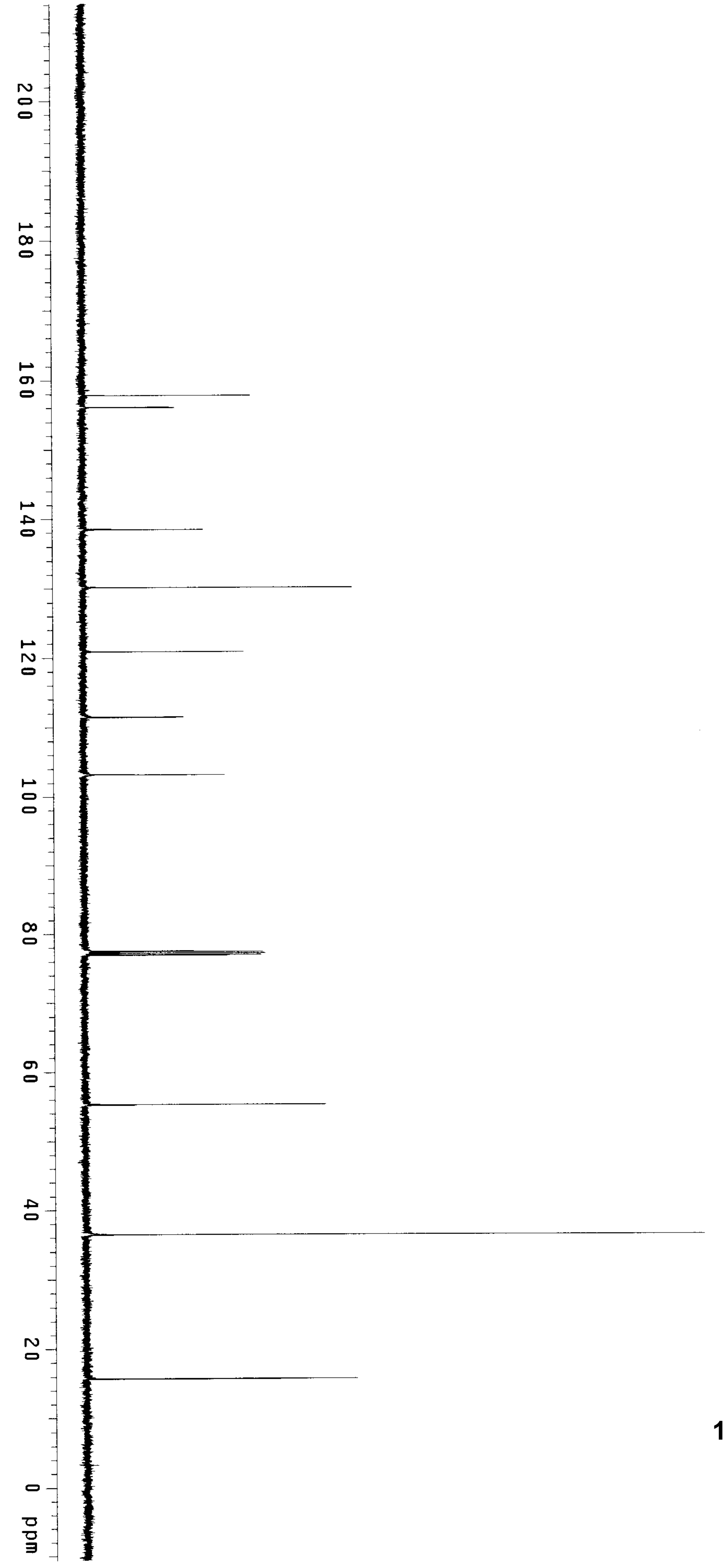

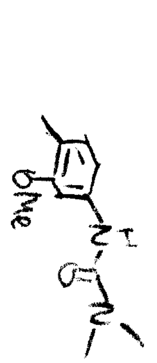

1h

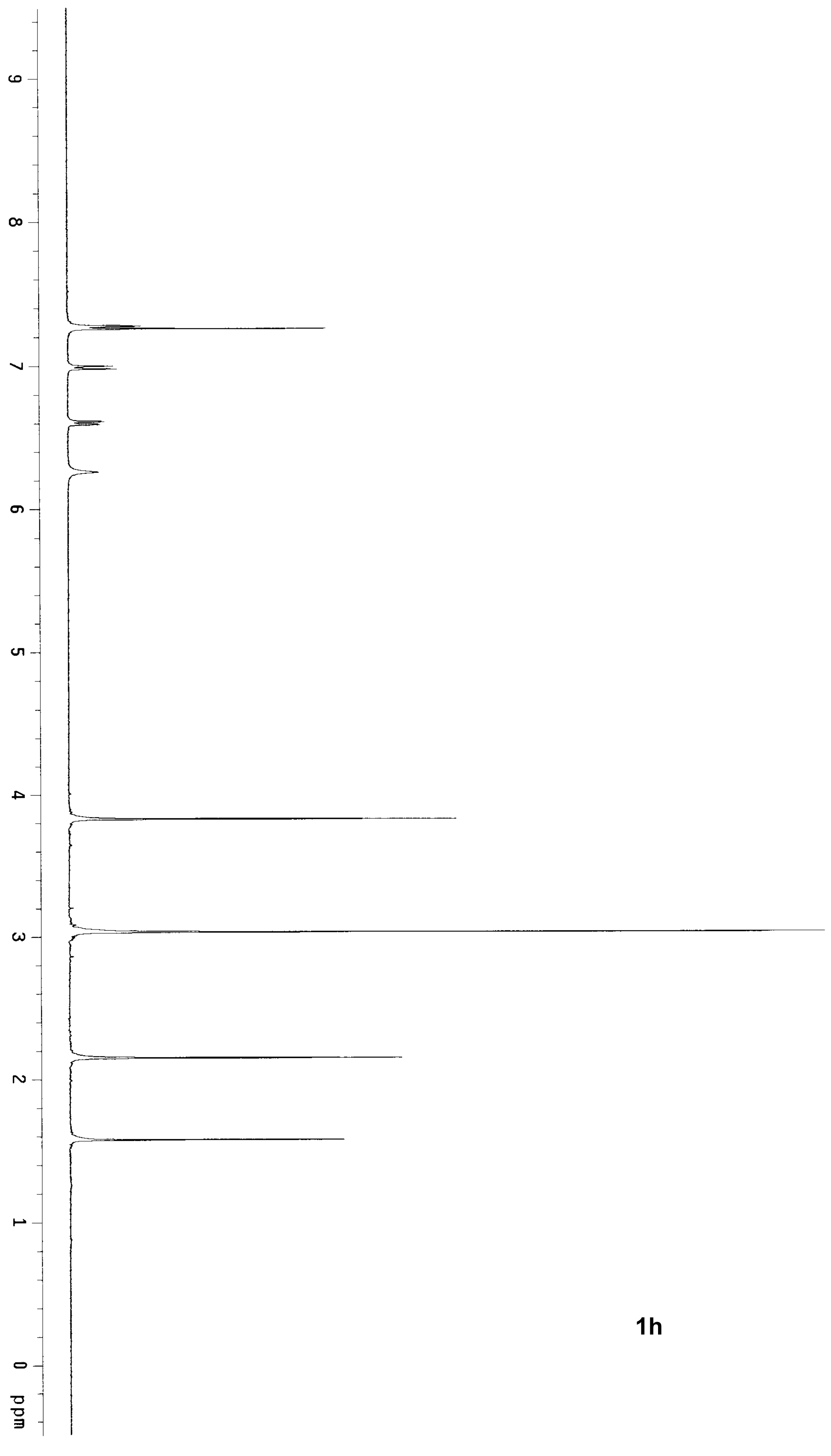

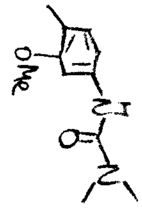

1h

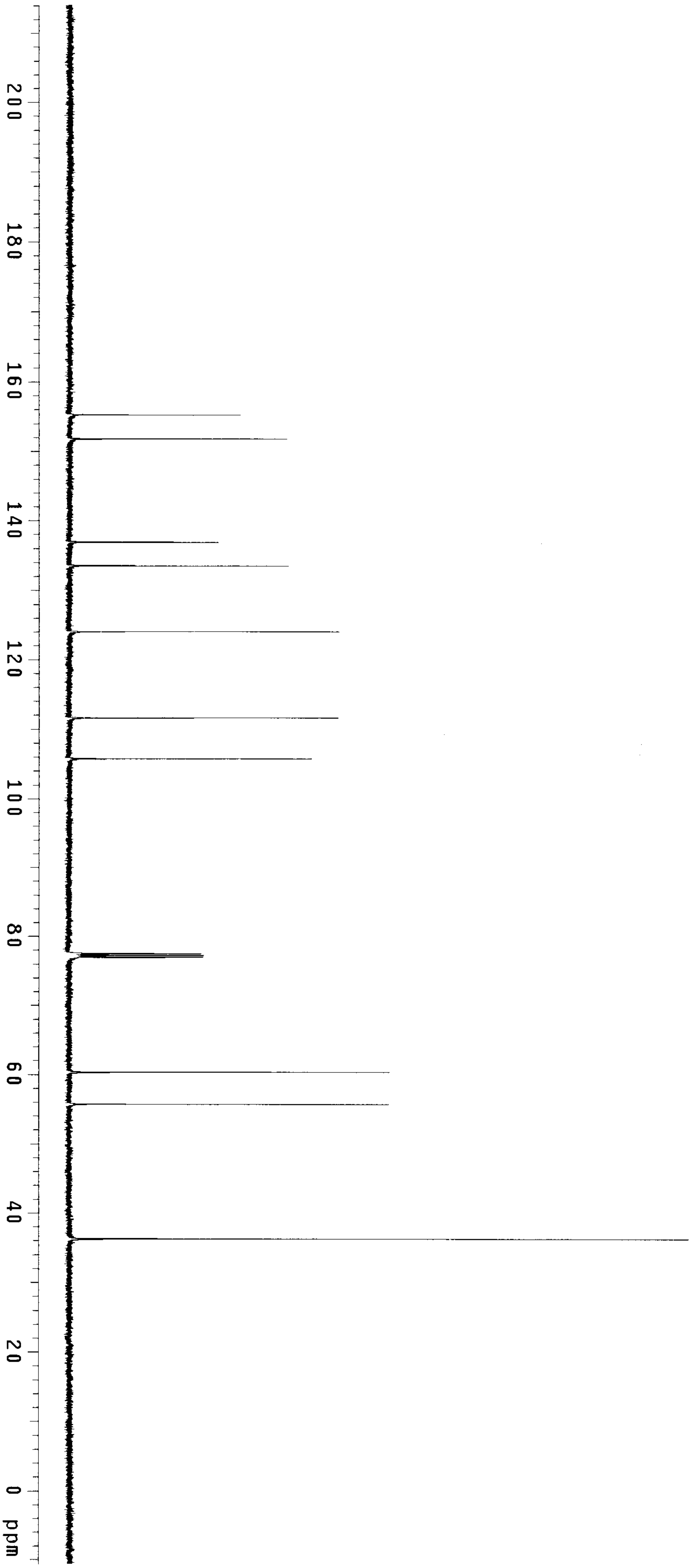

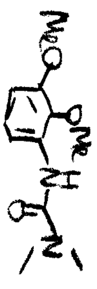

1i

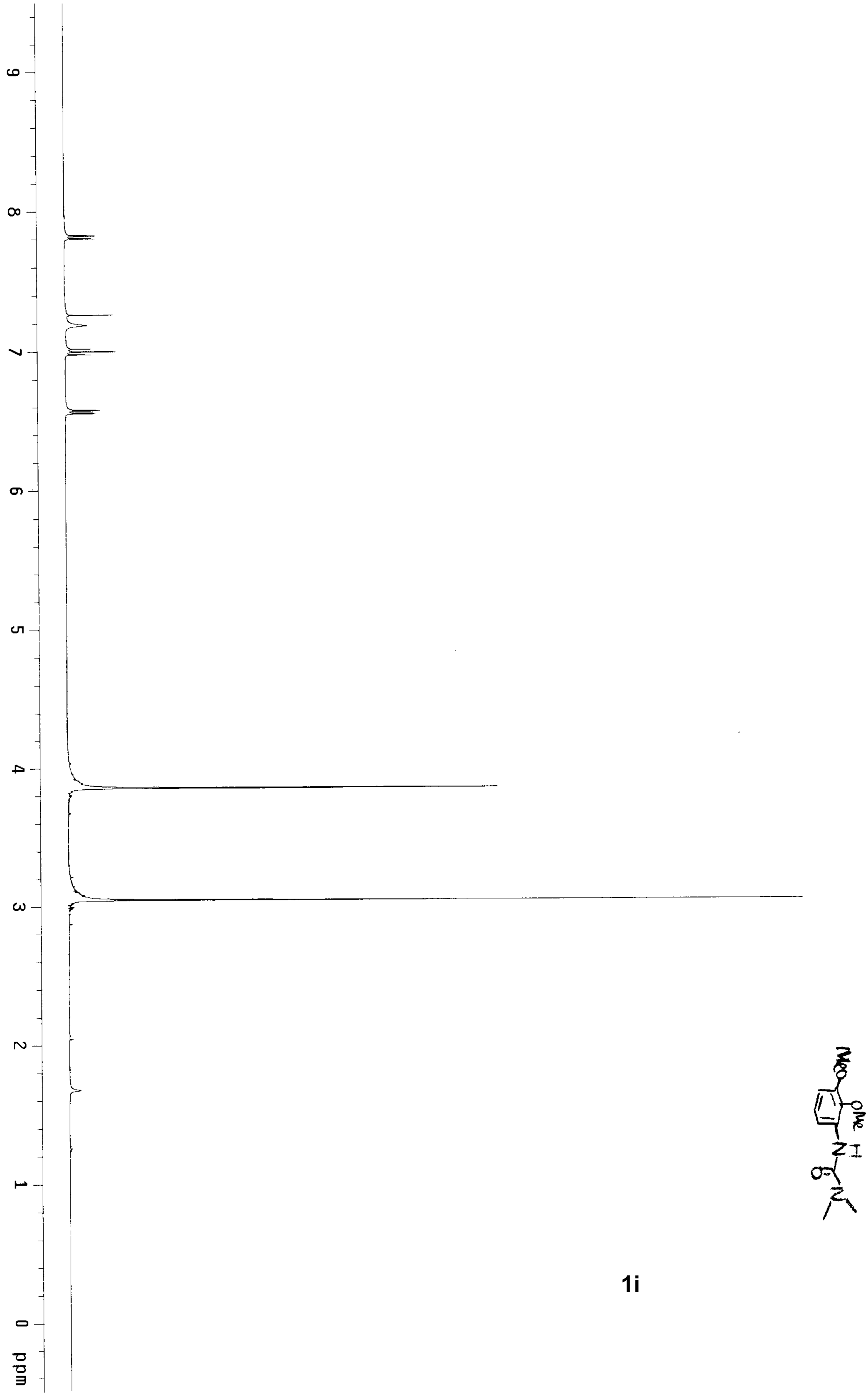

1i

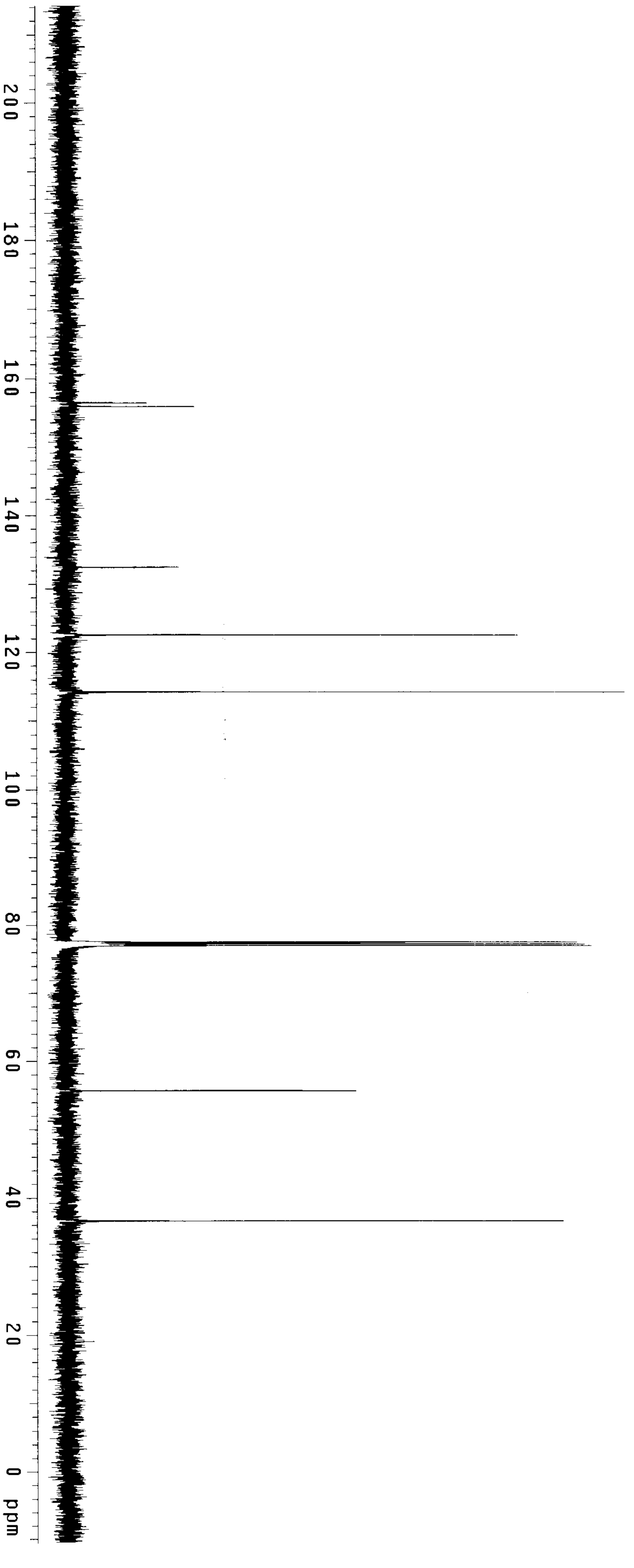

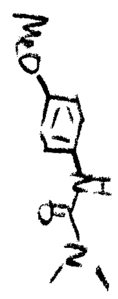

1j

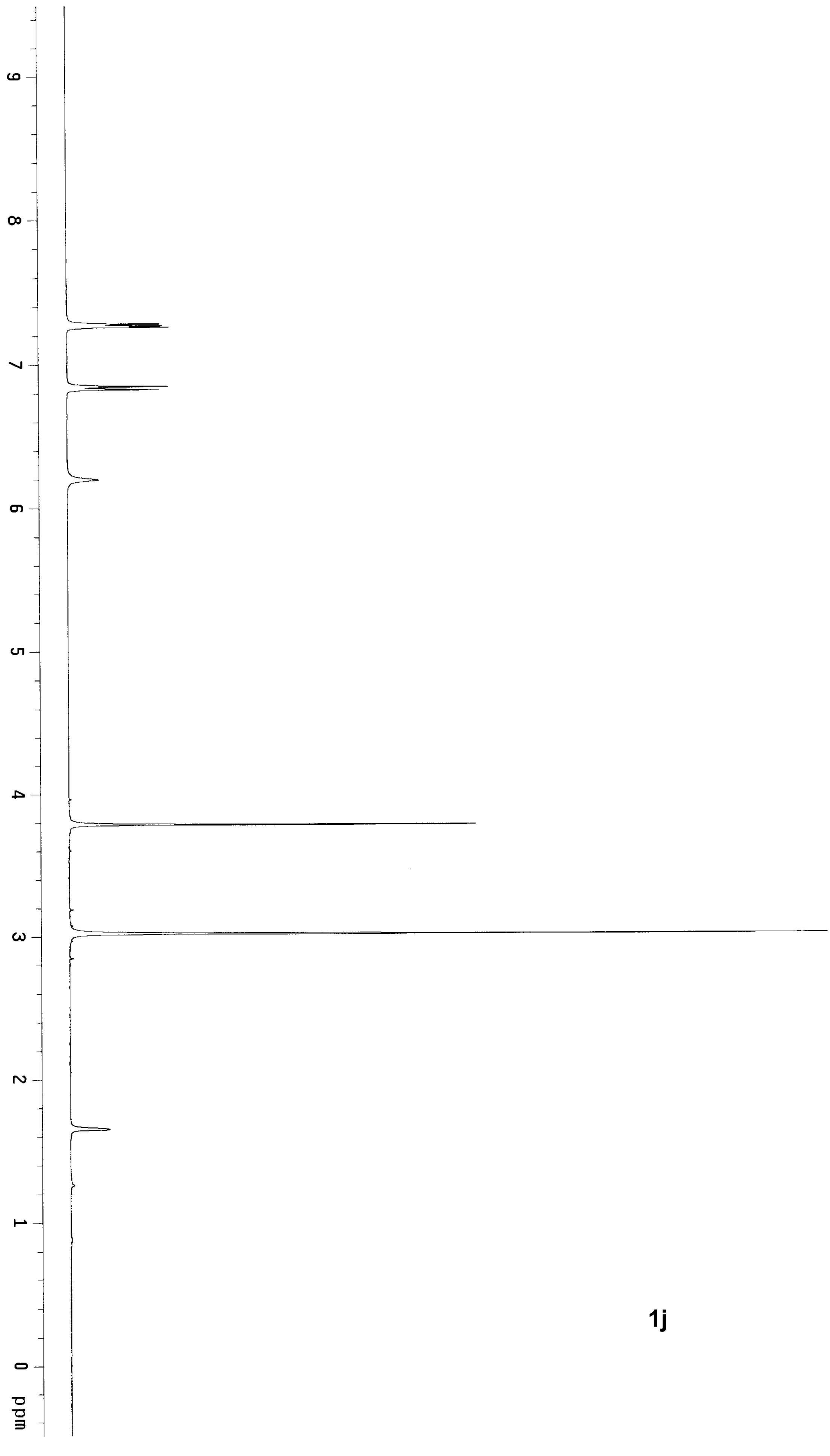

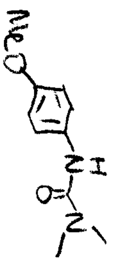

1j

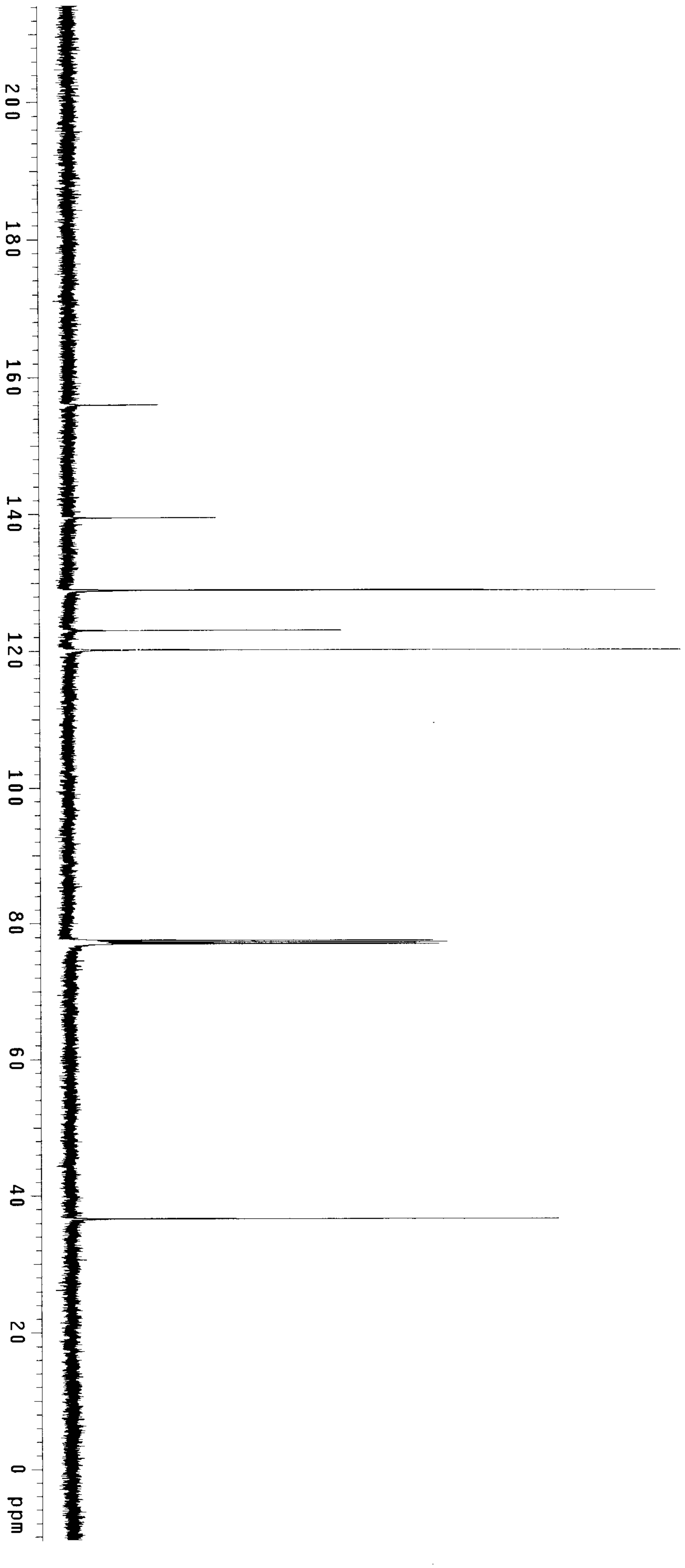

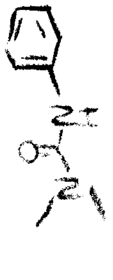

1k

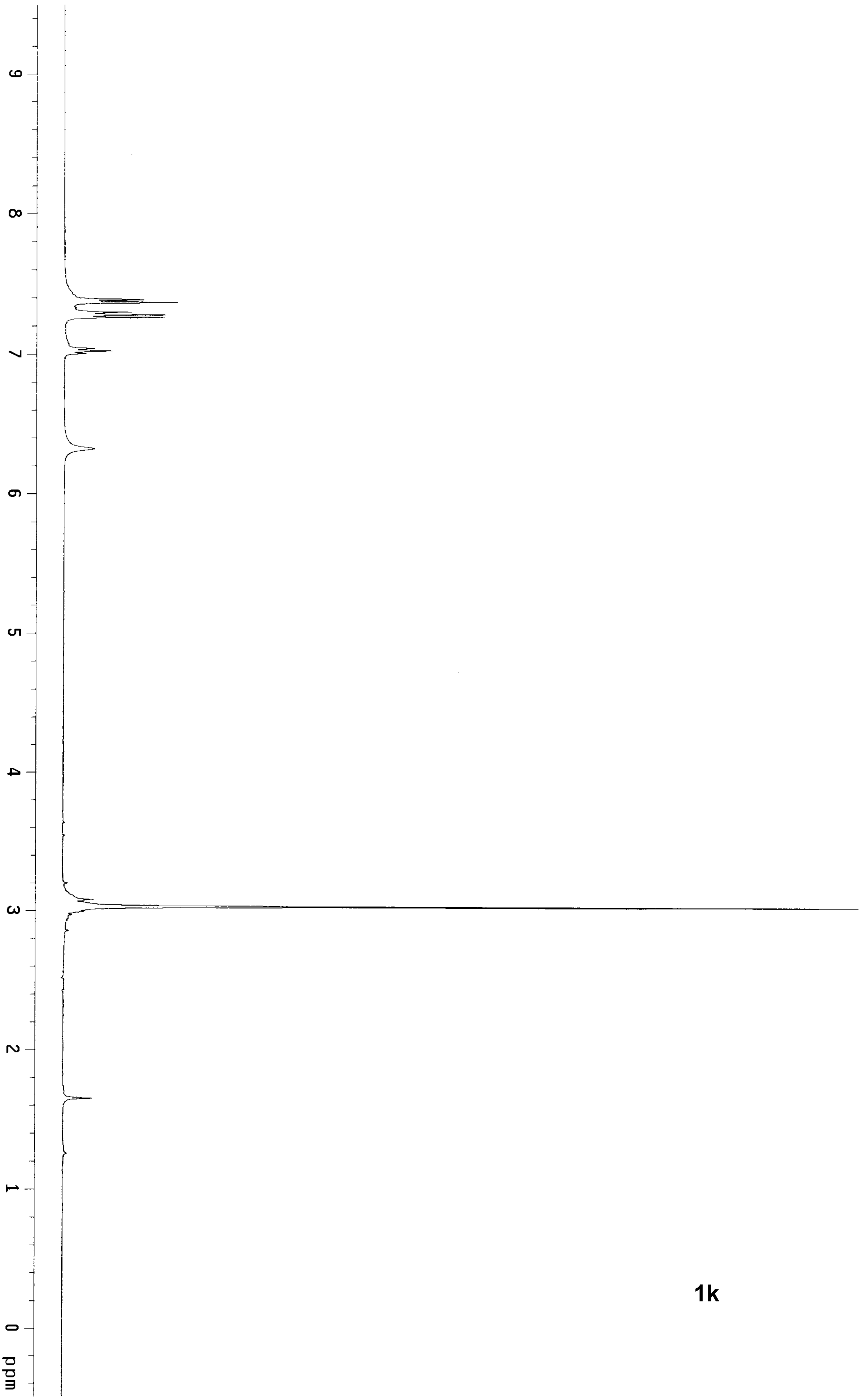

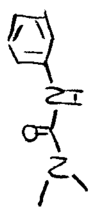

1k

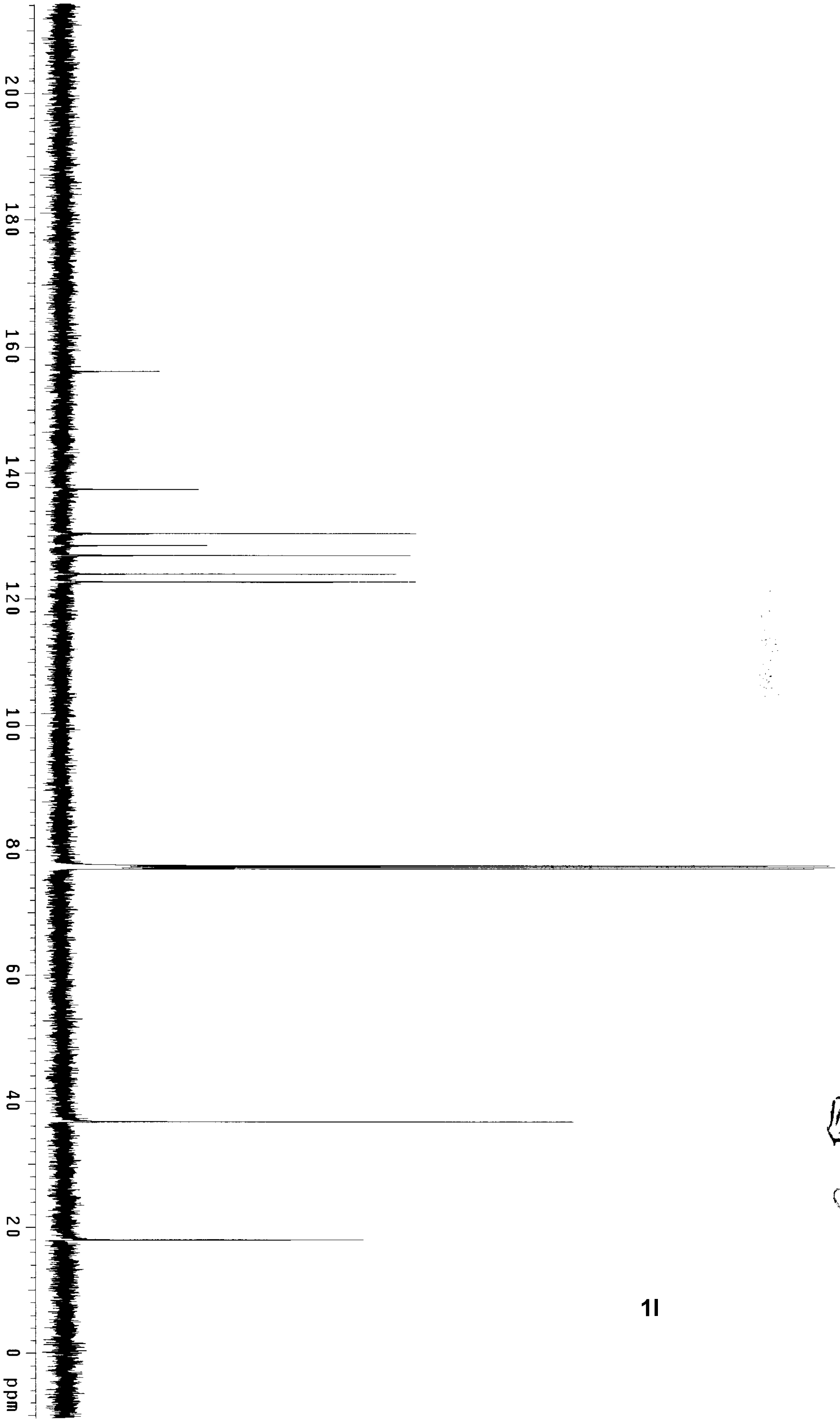

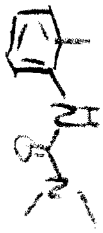

1l

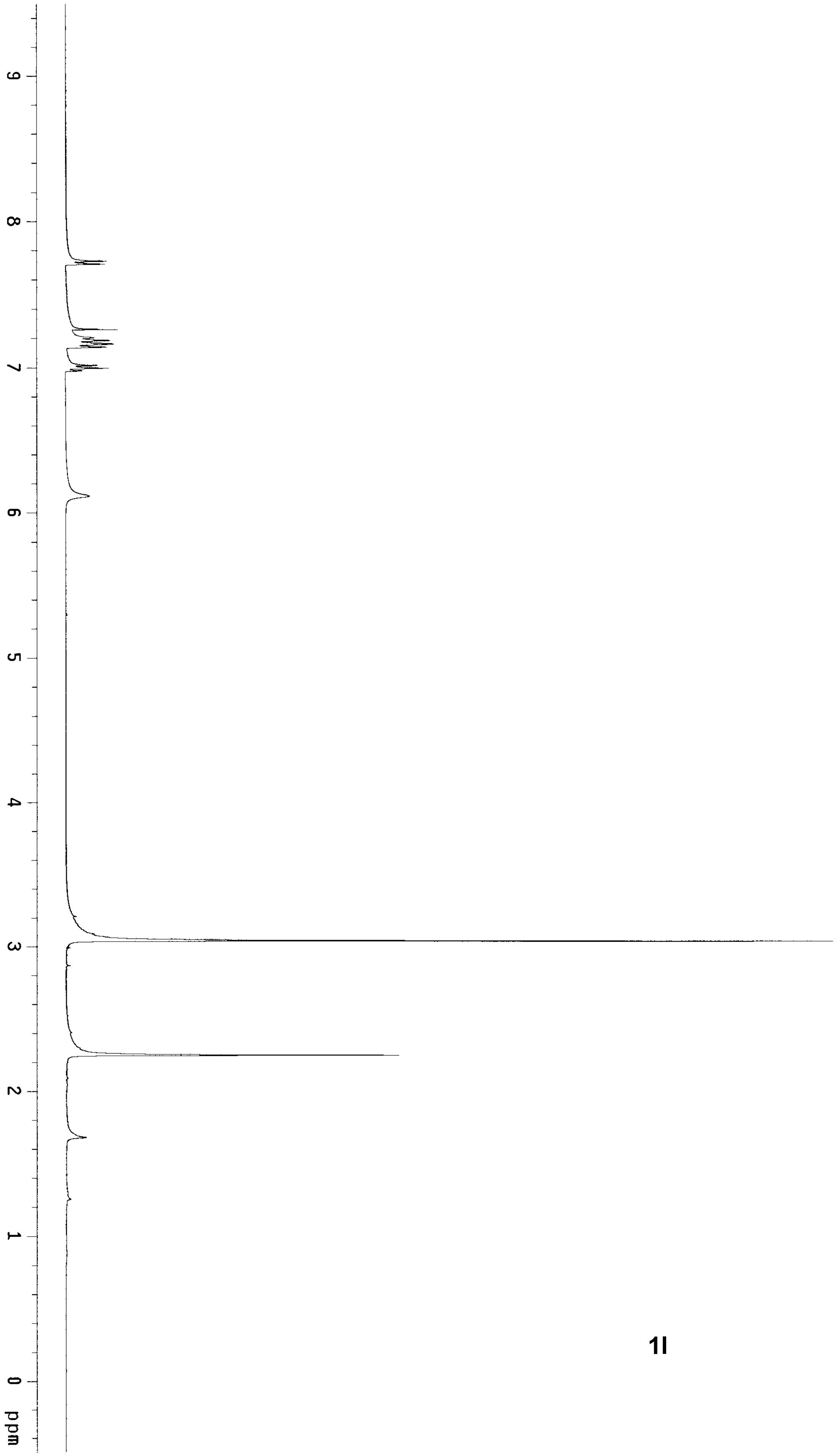

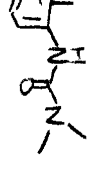

1l

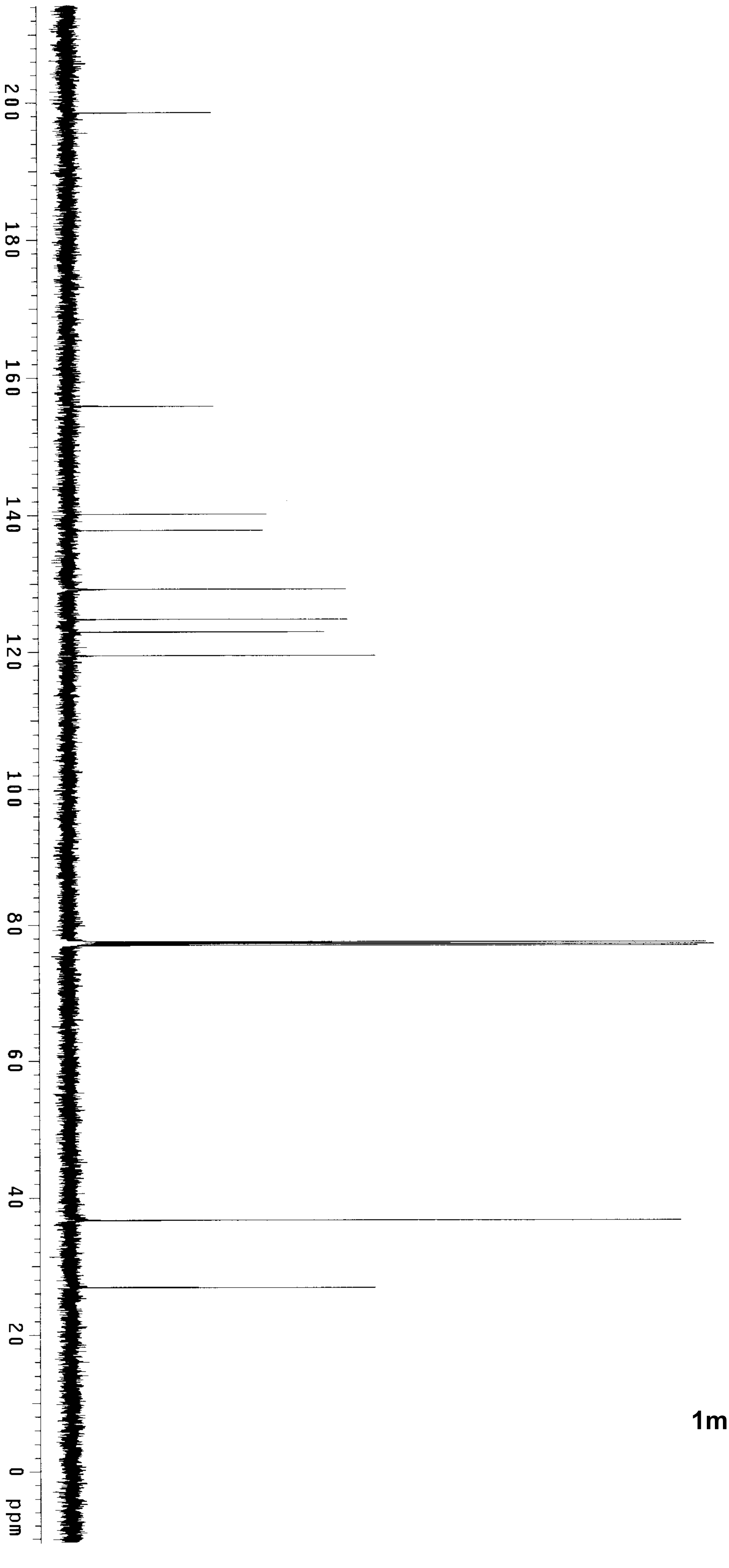

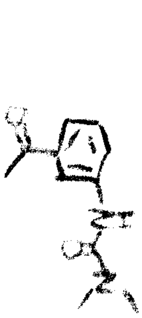

1m

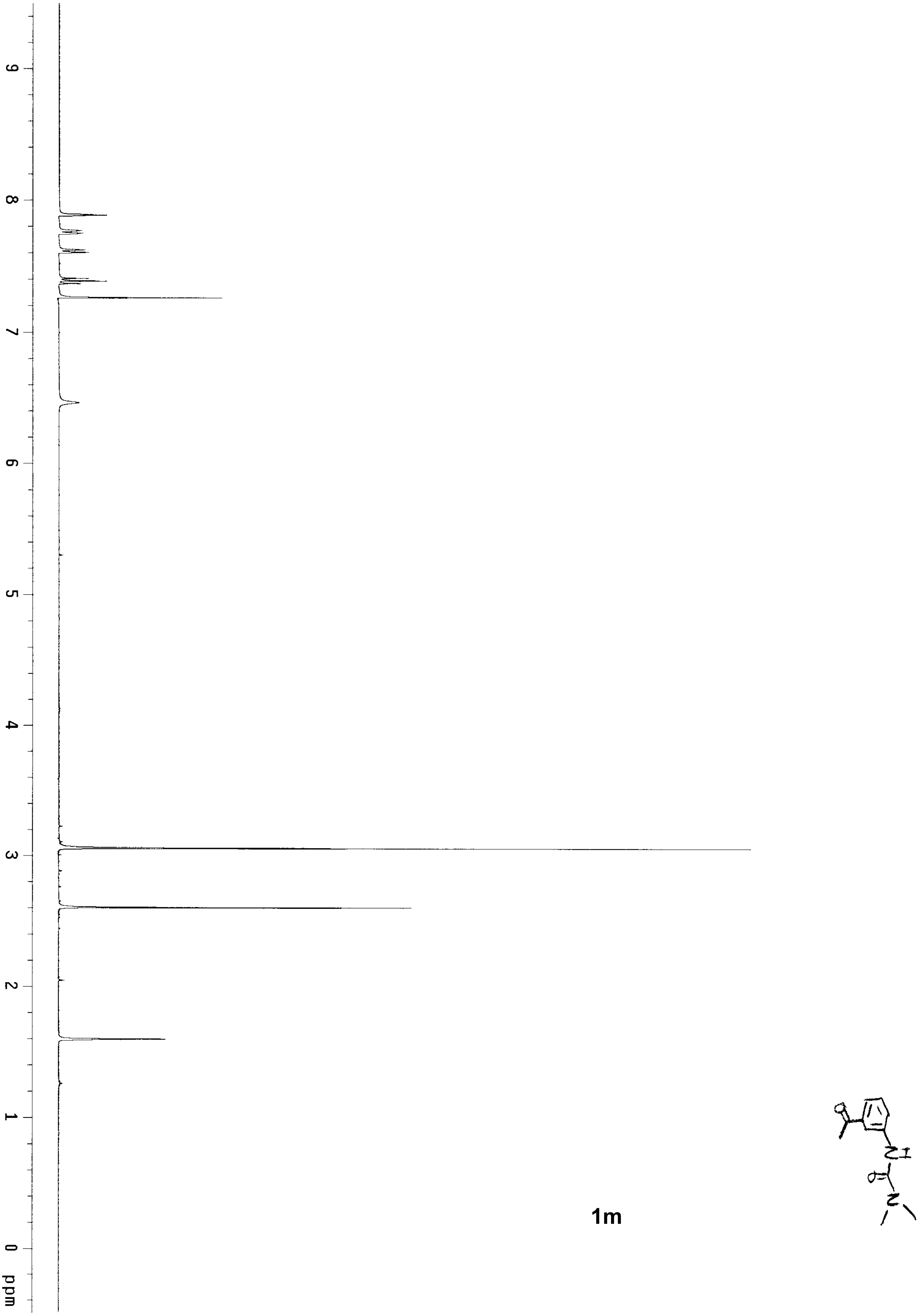

1m

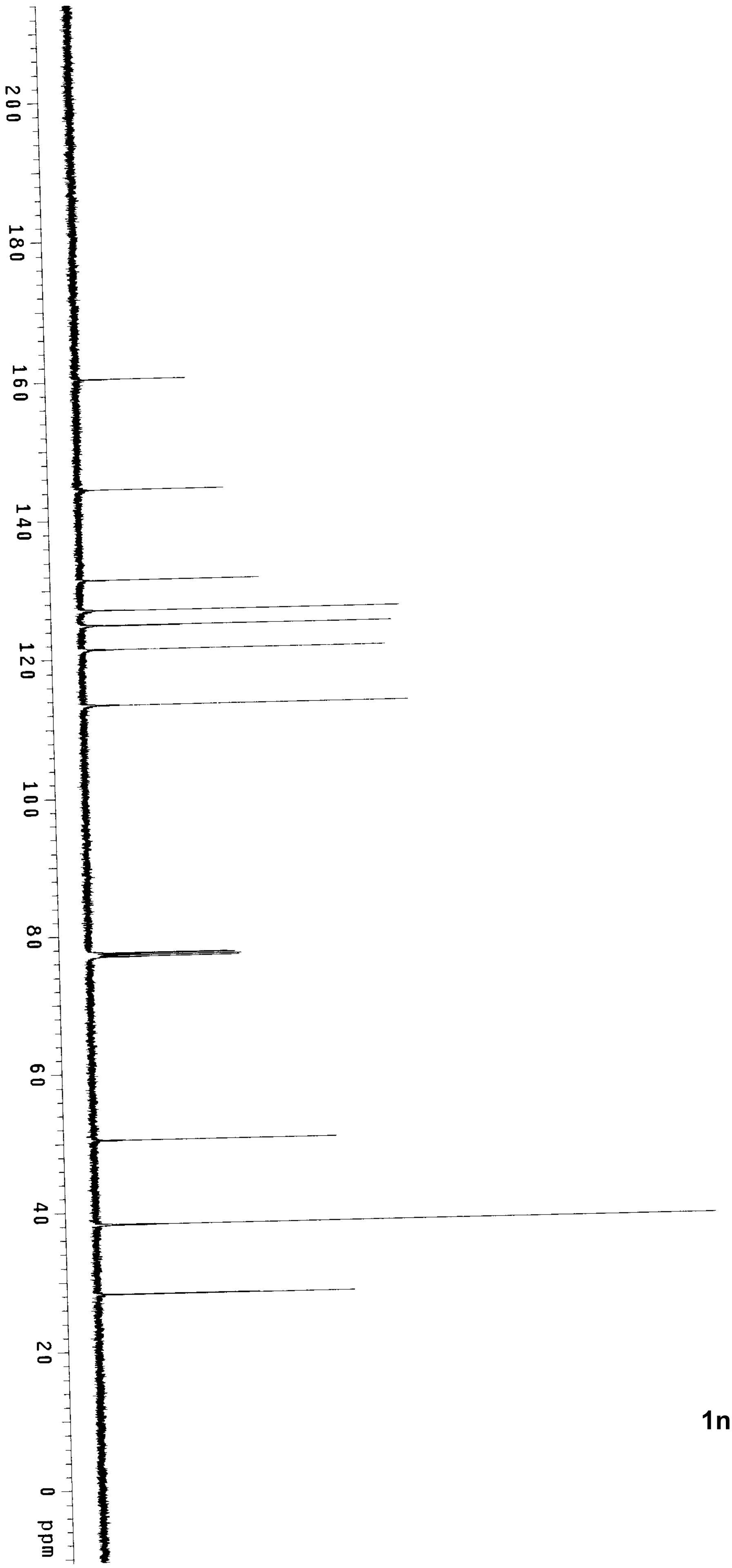

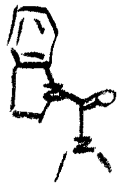

1n

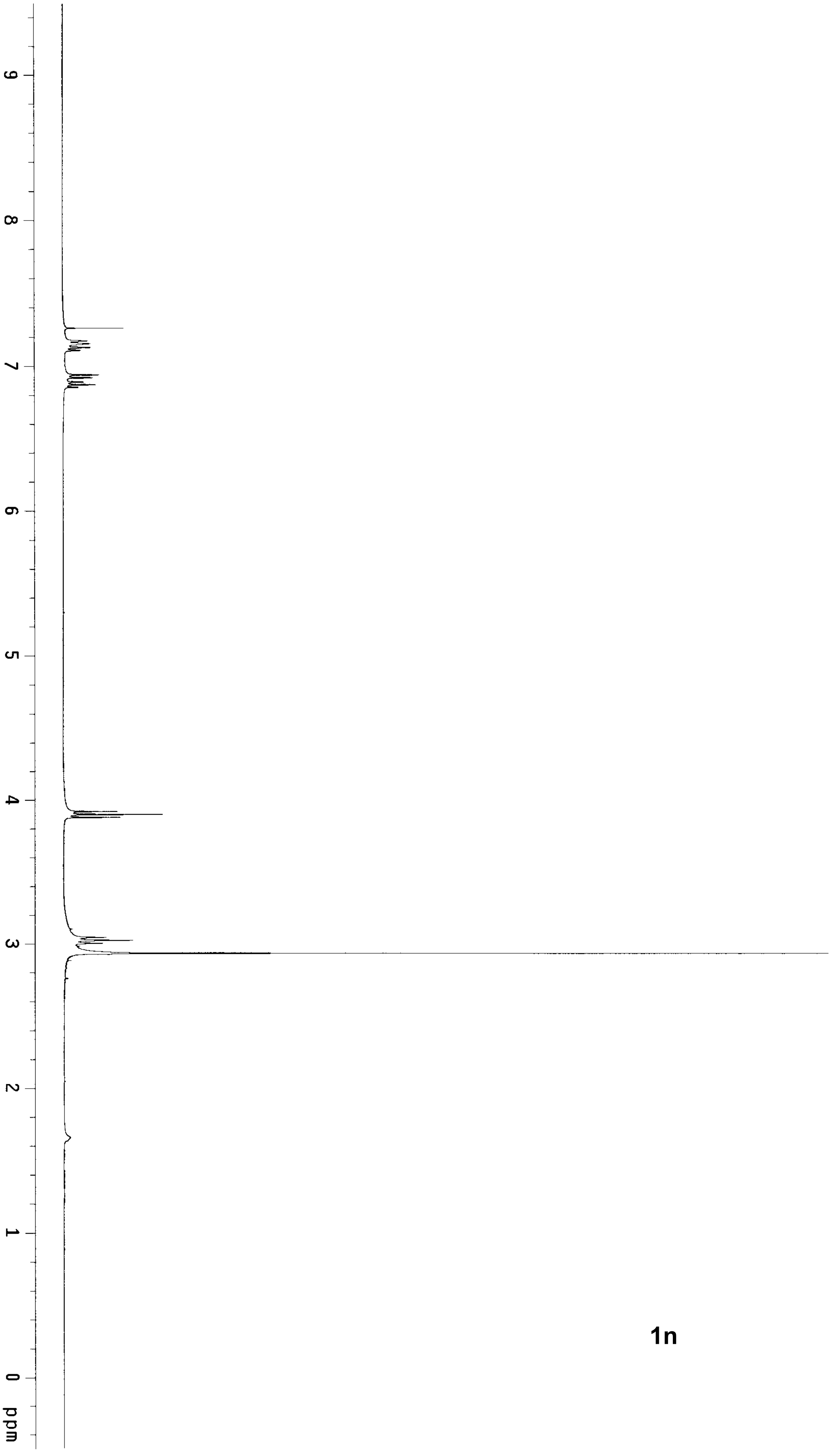

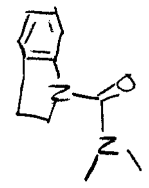

1n

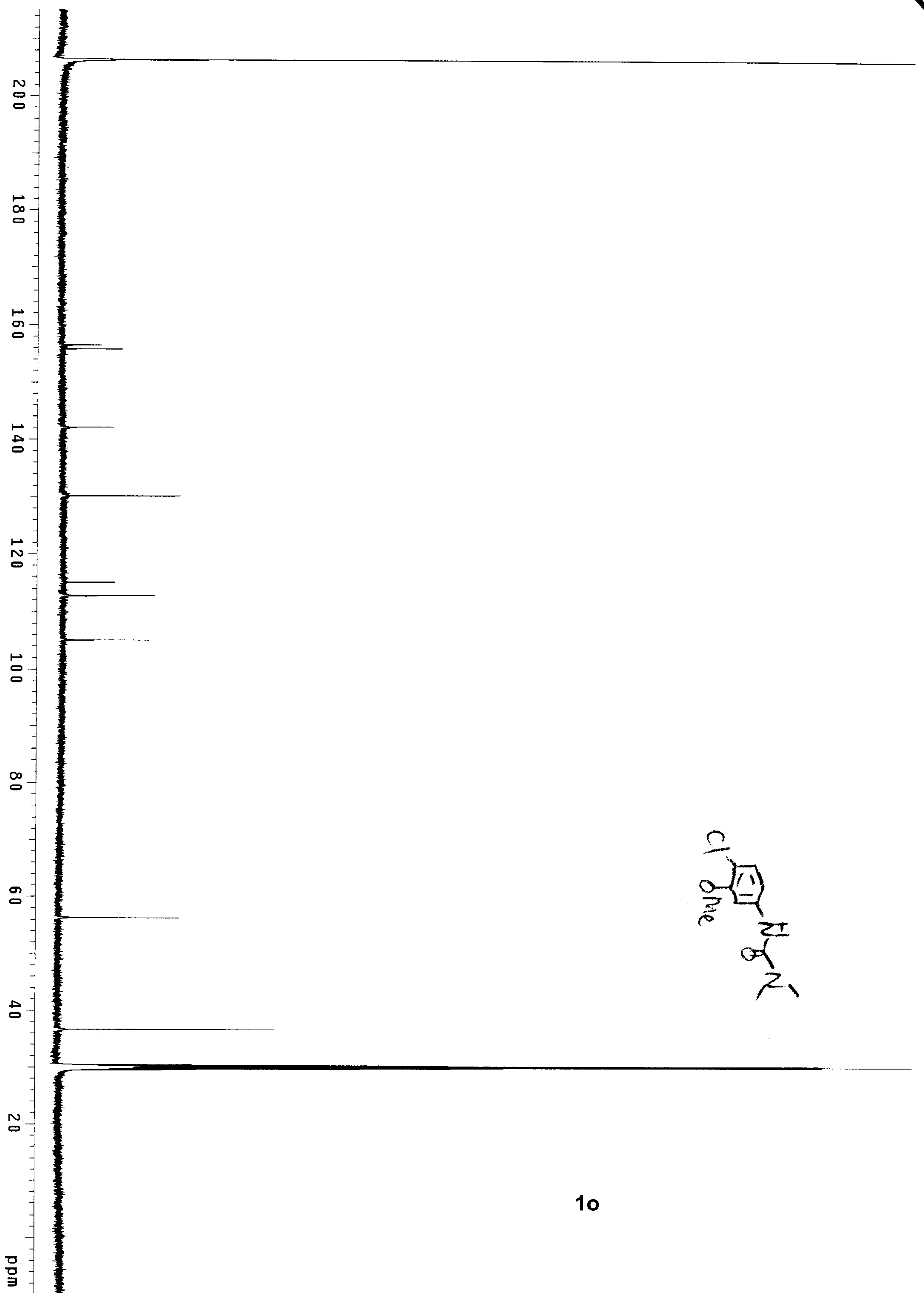

1o

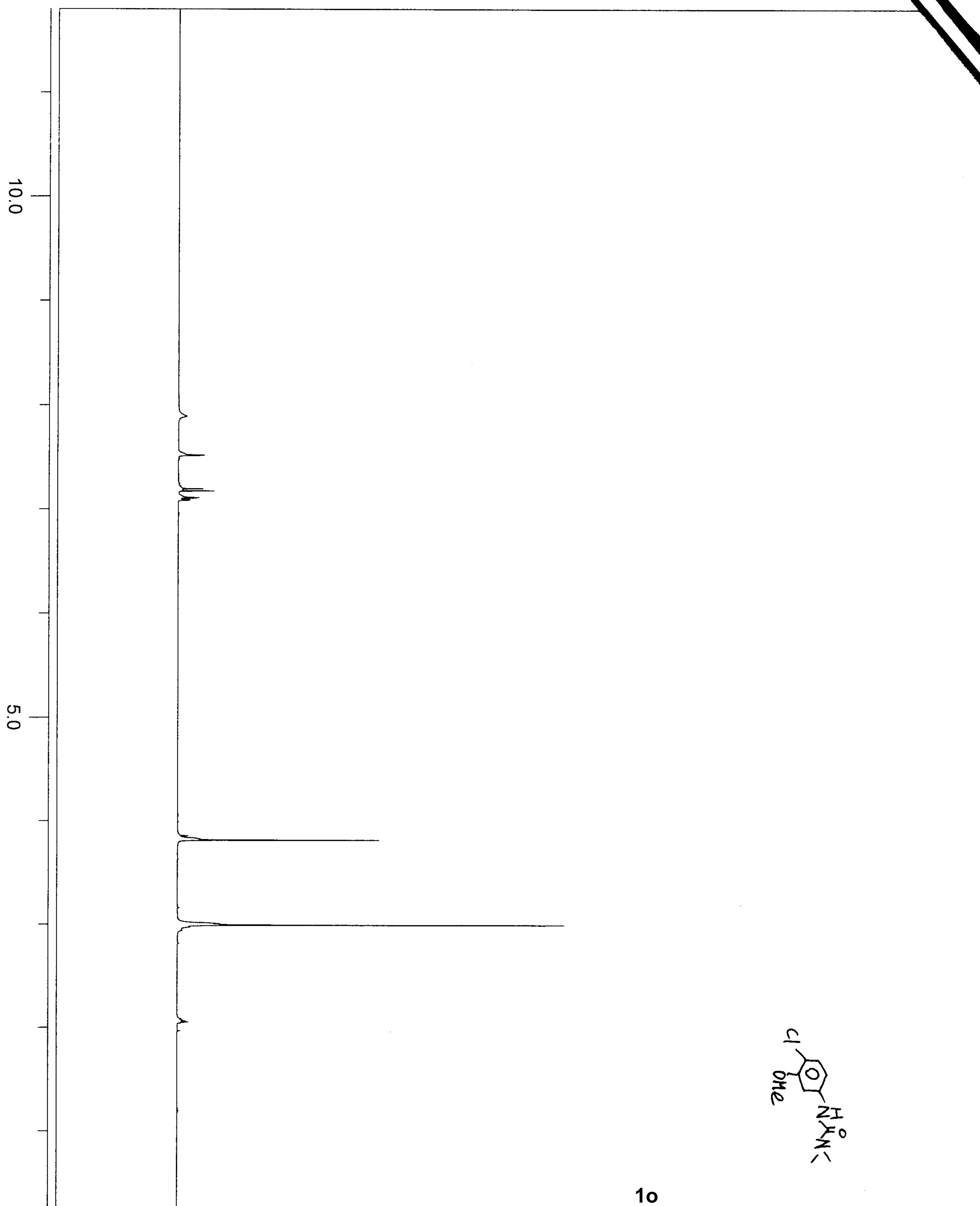

1o

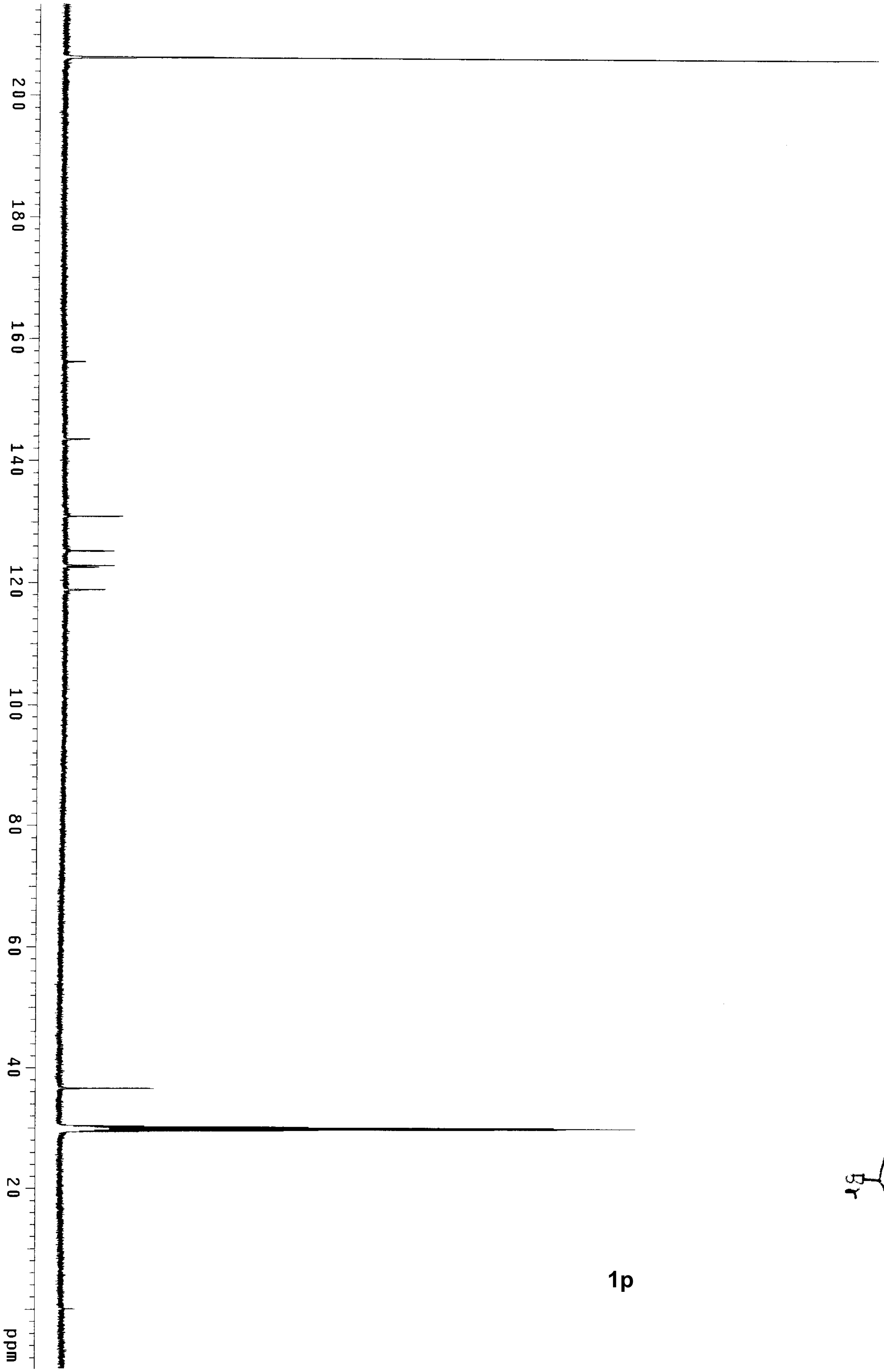

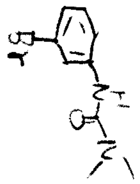

1p

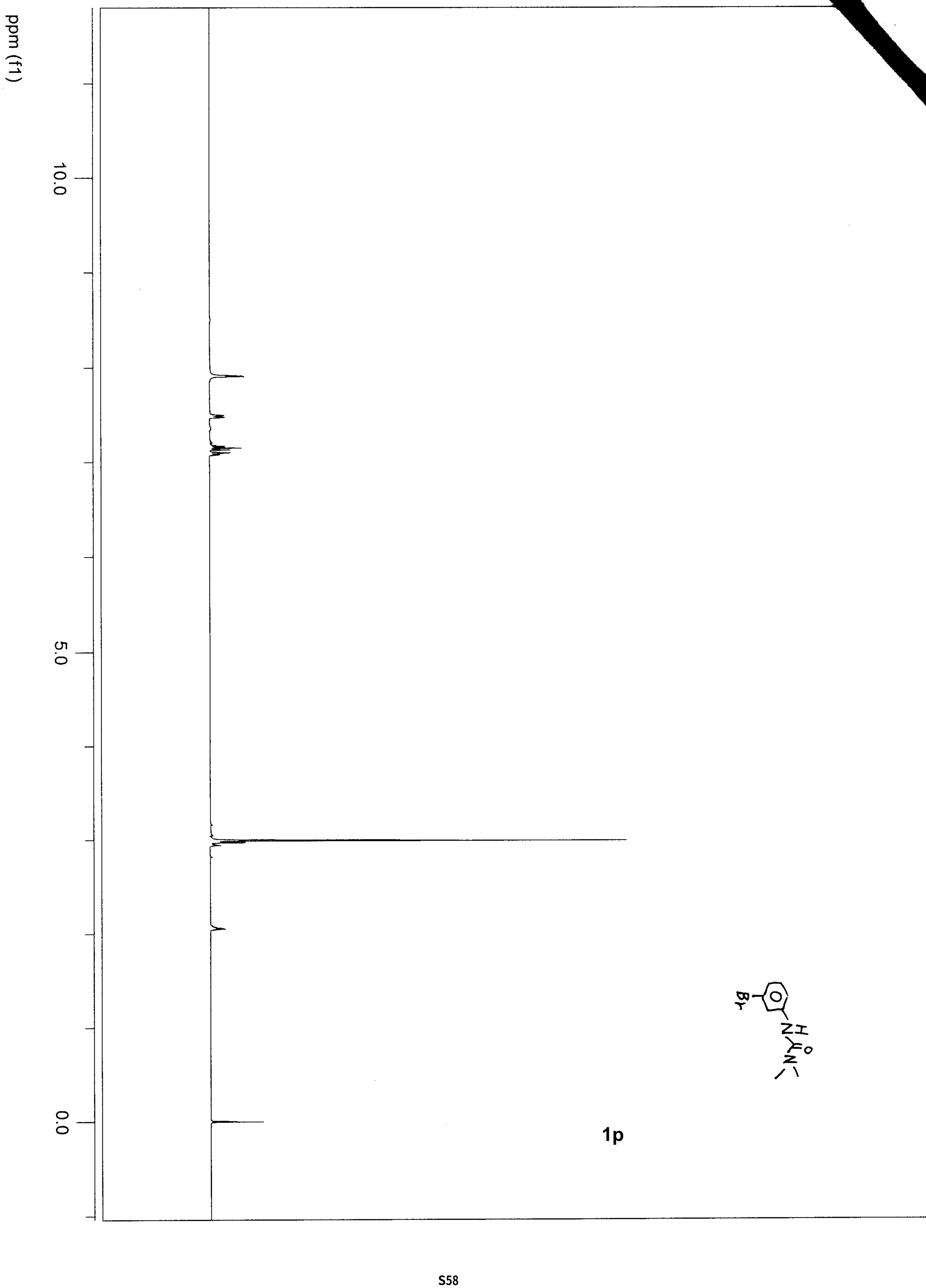
ppm (f1)
10.0
5.0
0.0
Br
1p

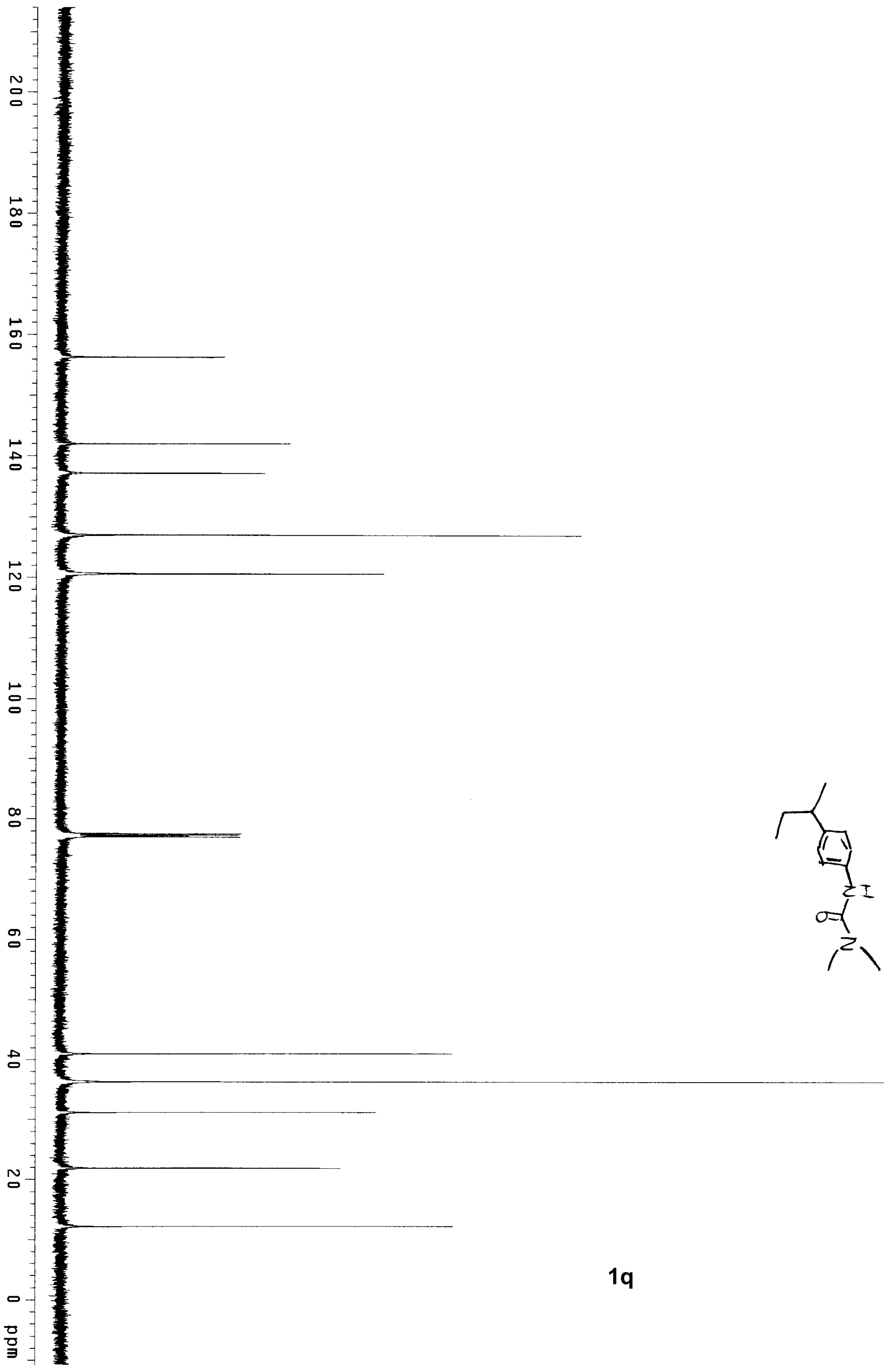

1q

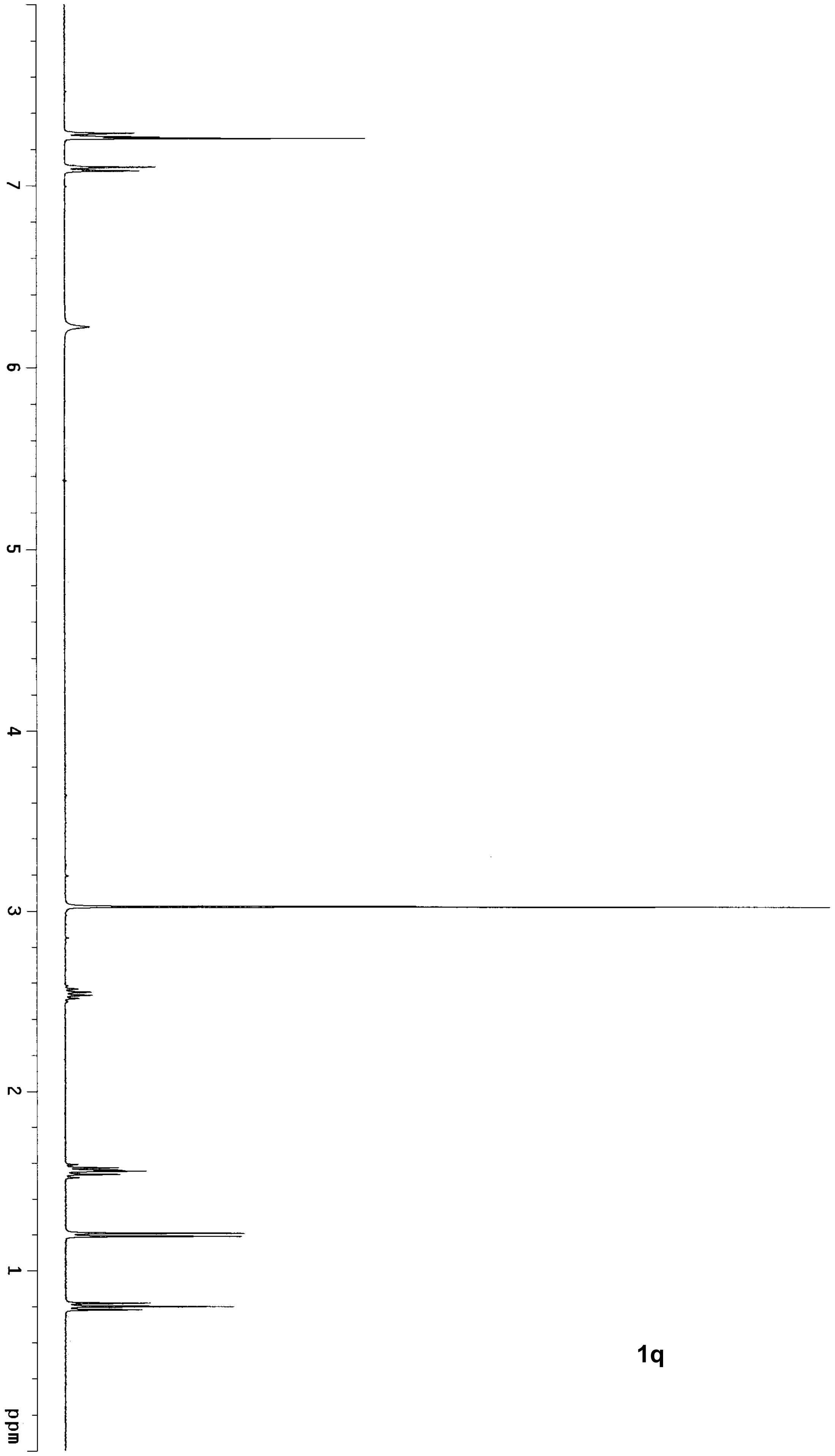

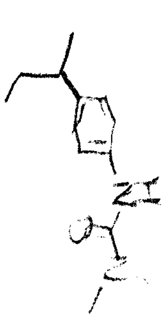

1q

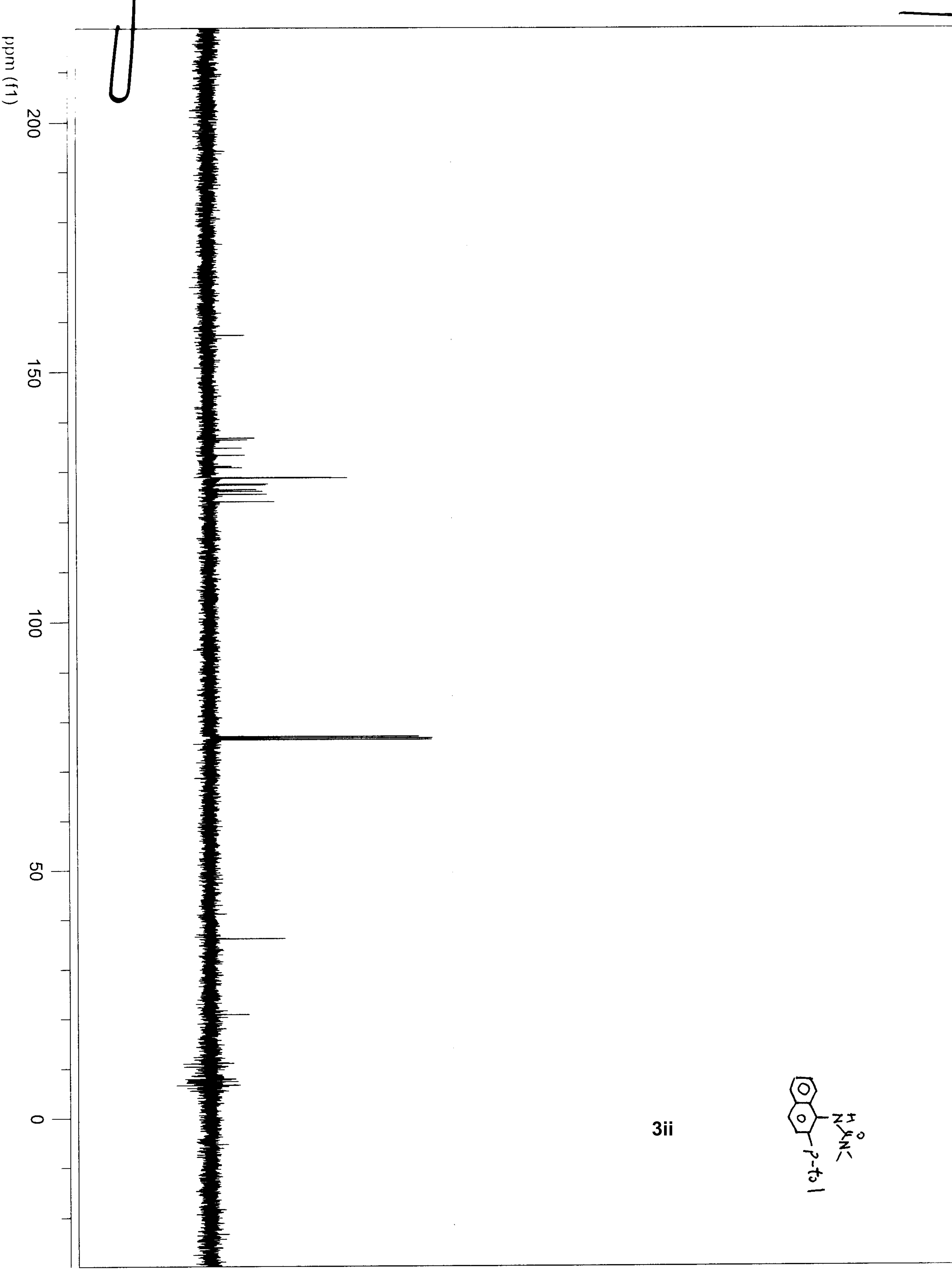
ppm (f1)
200
150
100
50
0
3ii

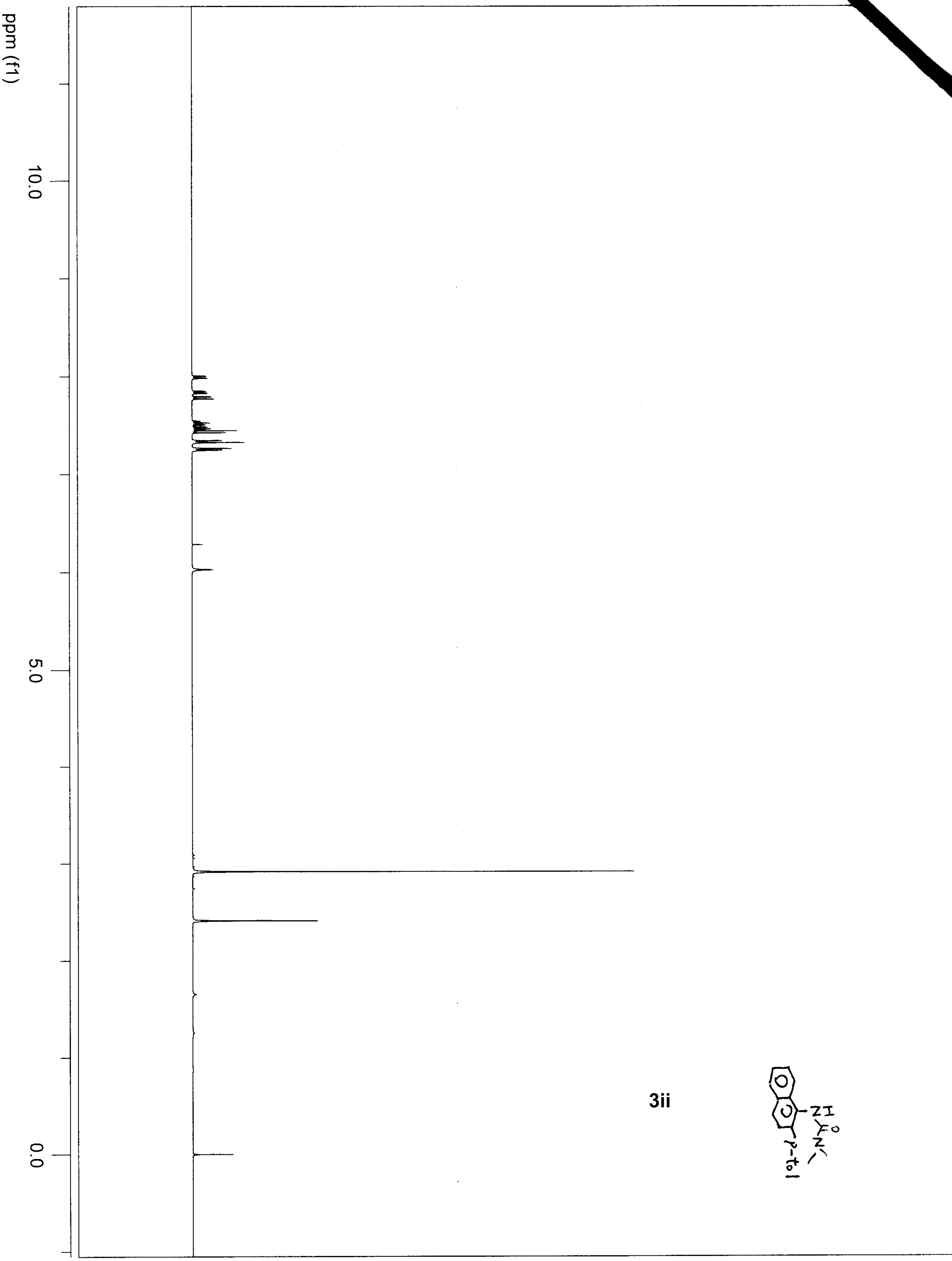
ppm (f1)
10.0
5.0
0.0
3ii

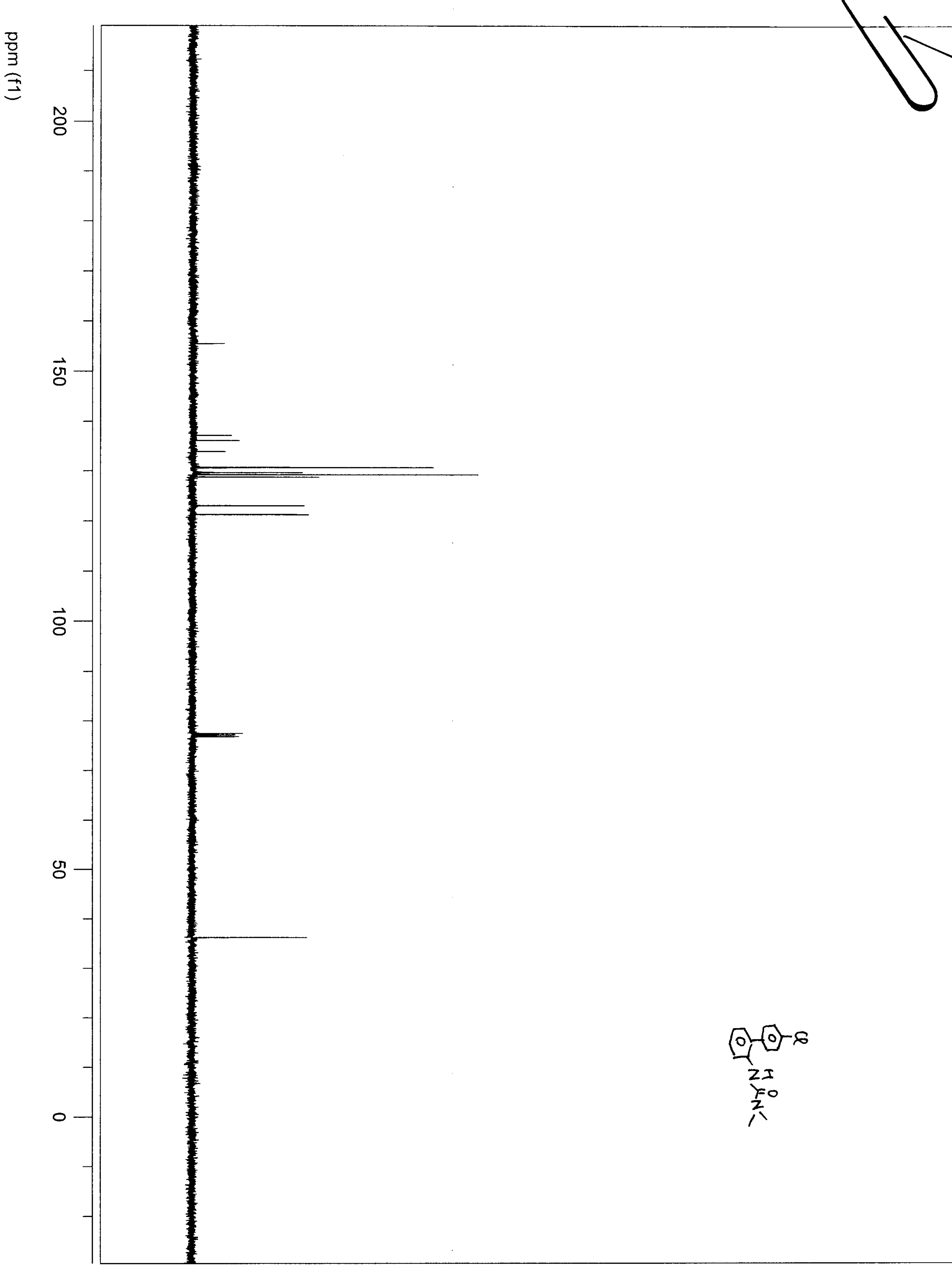
ppm (f1)
200
150
100
50
0

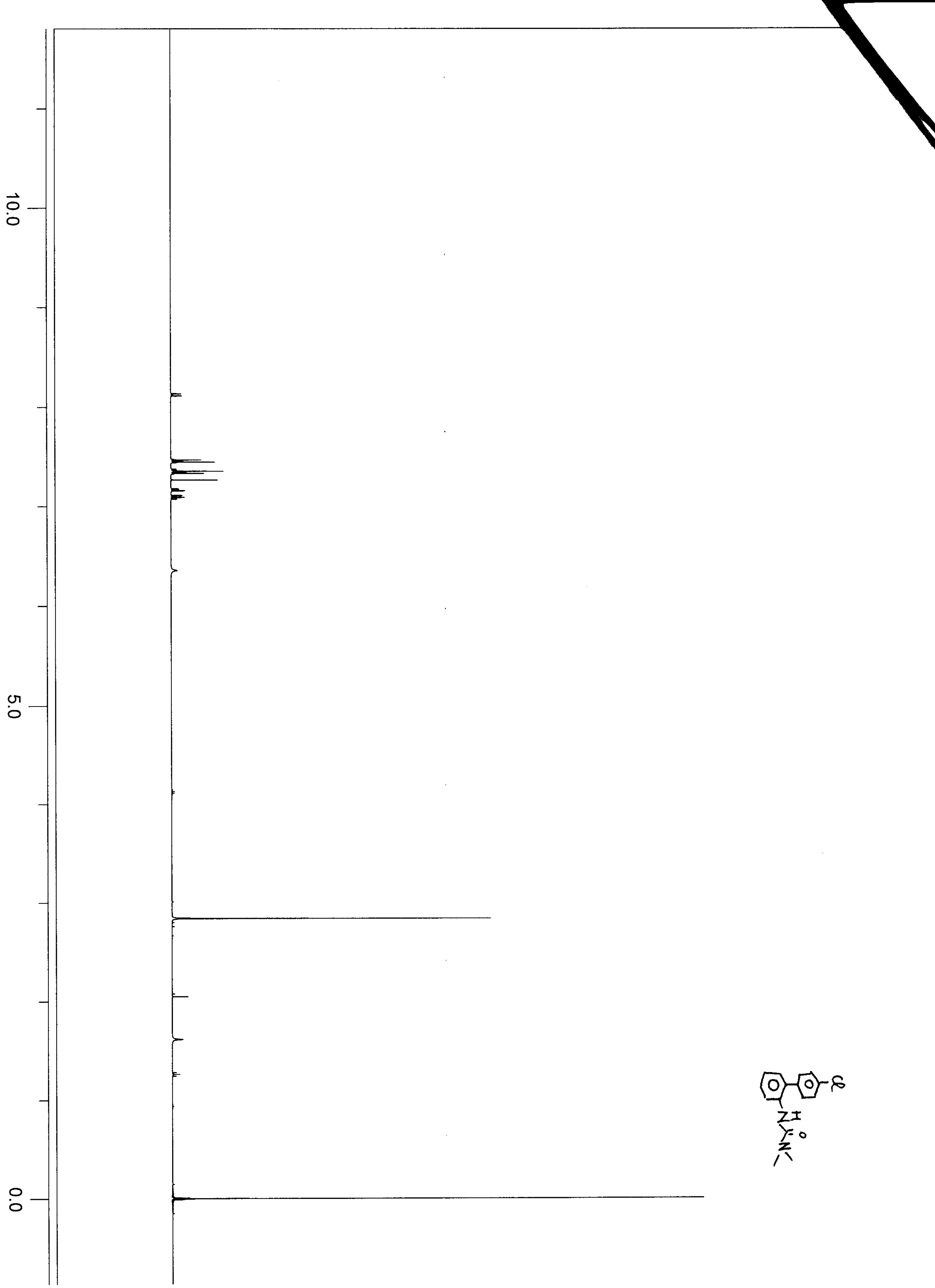
10.0
5.0
0.0

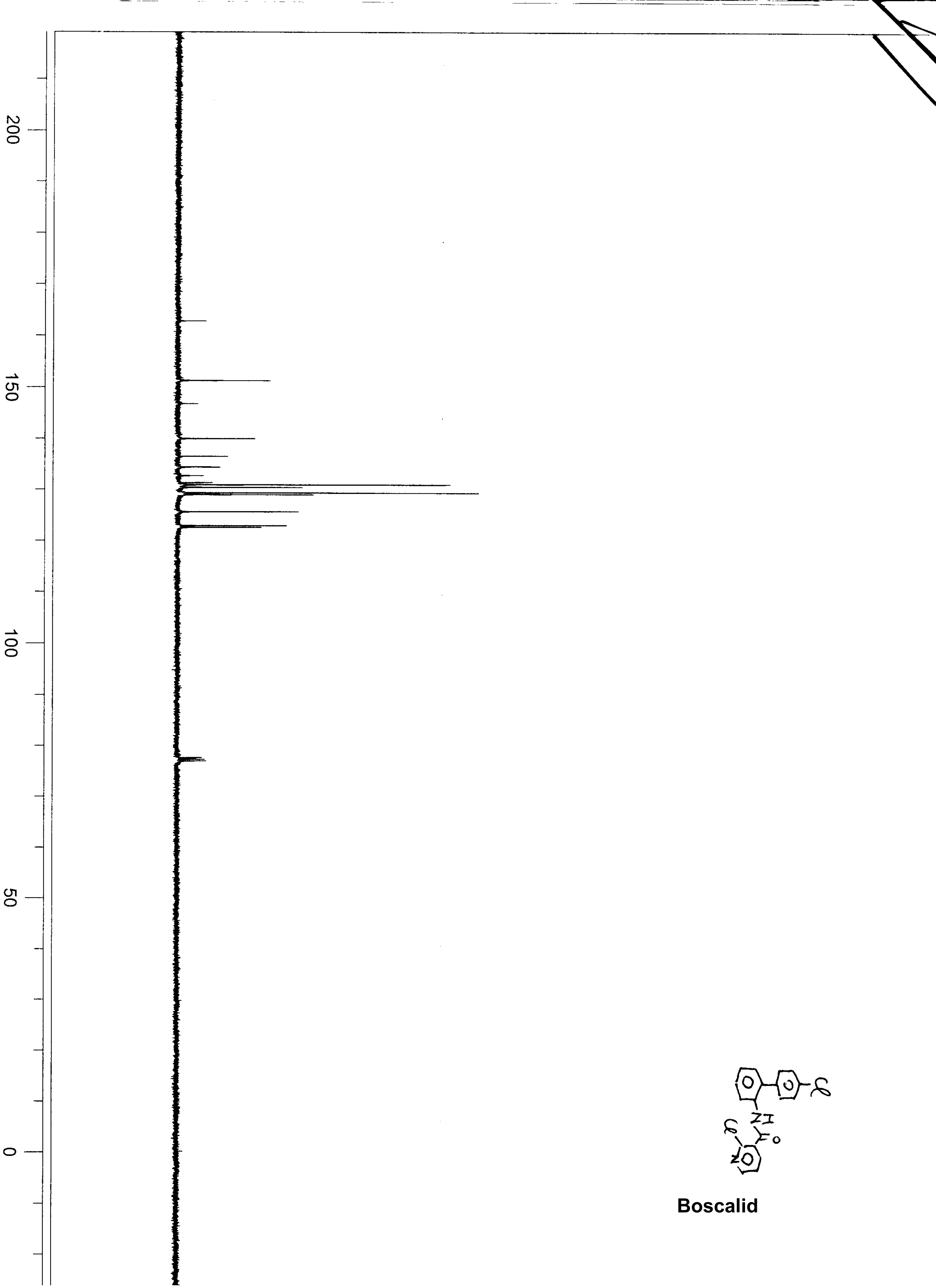

Boscalid

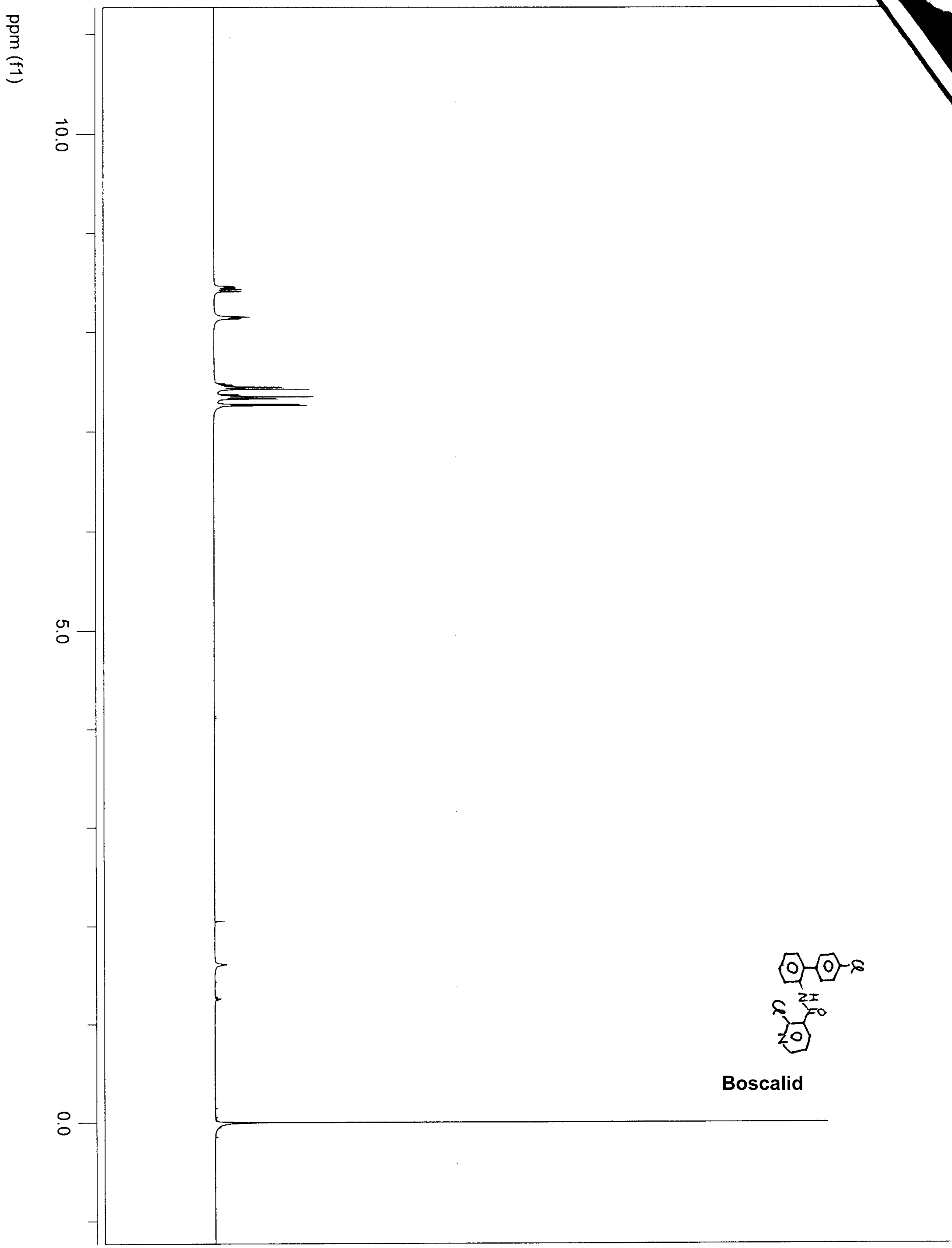
ppm (f1)
10.0
5.0
0.0
Boscalid

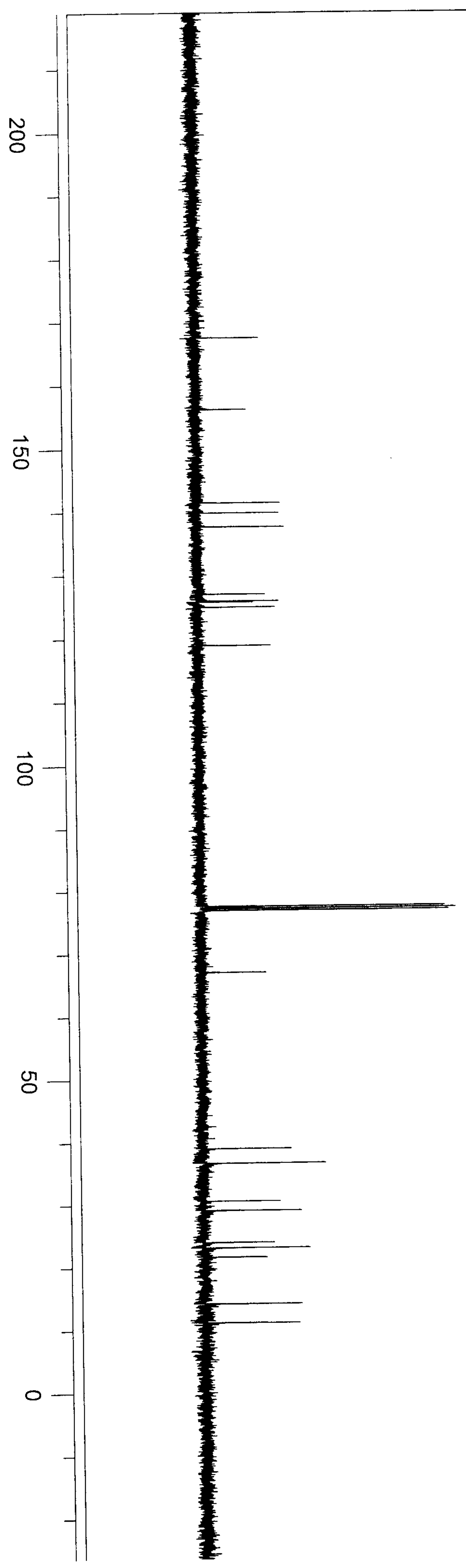

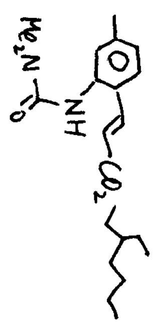

5d

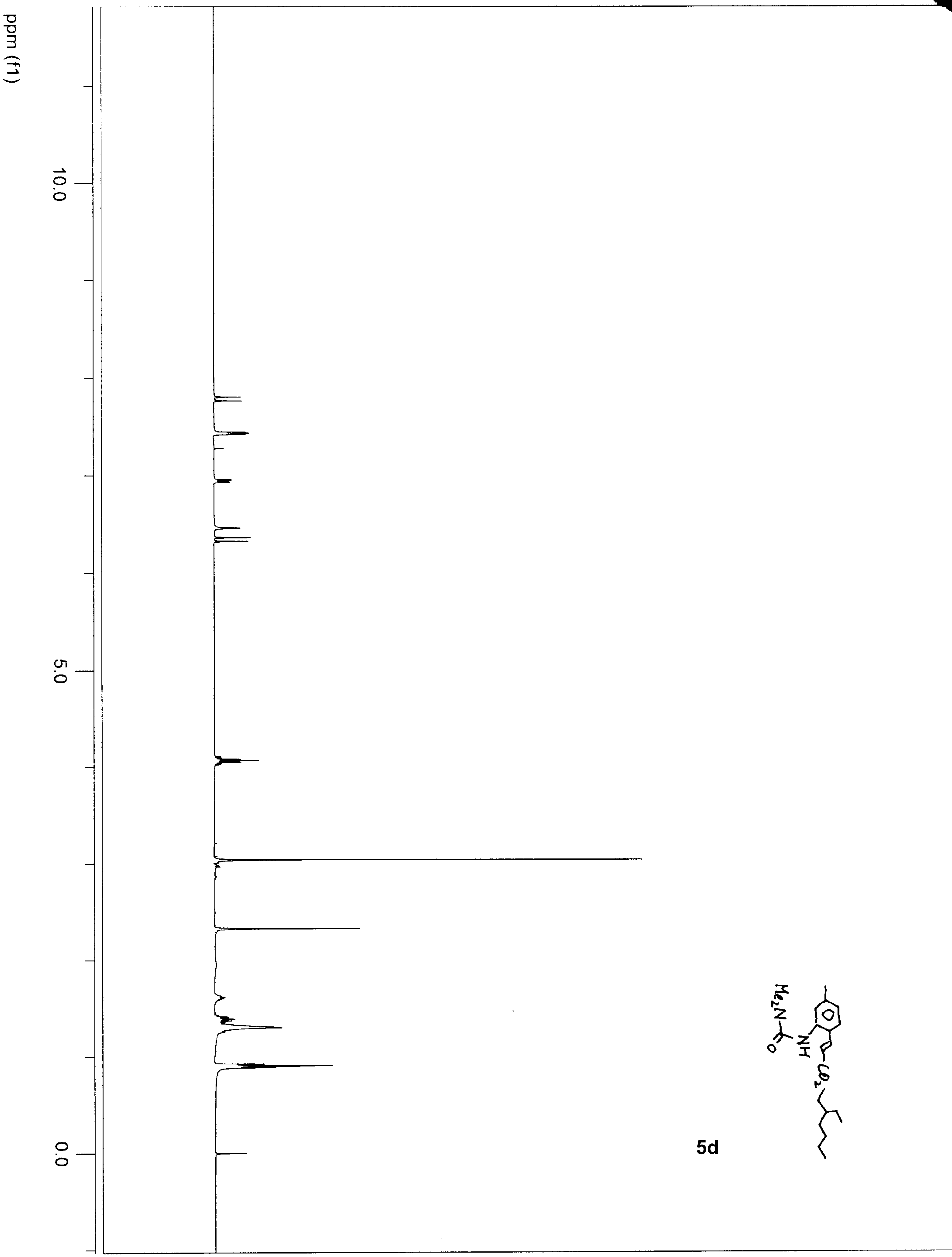
ppm (f1)
10.0
5.0
0.0
5d

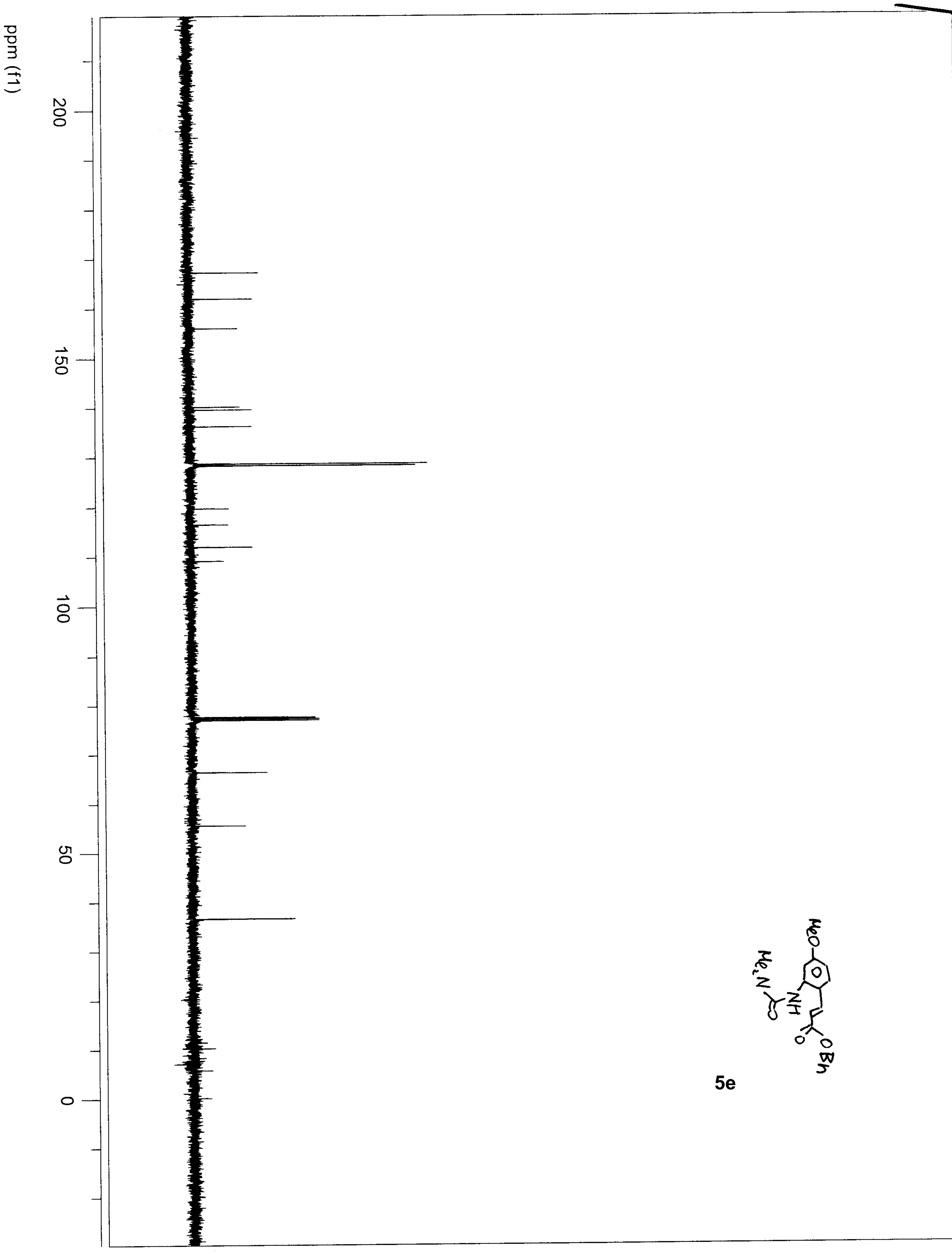
ppm (f1)
200
150
100
50
0
5e

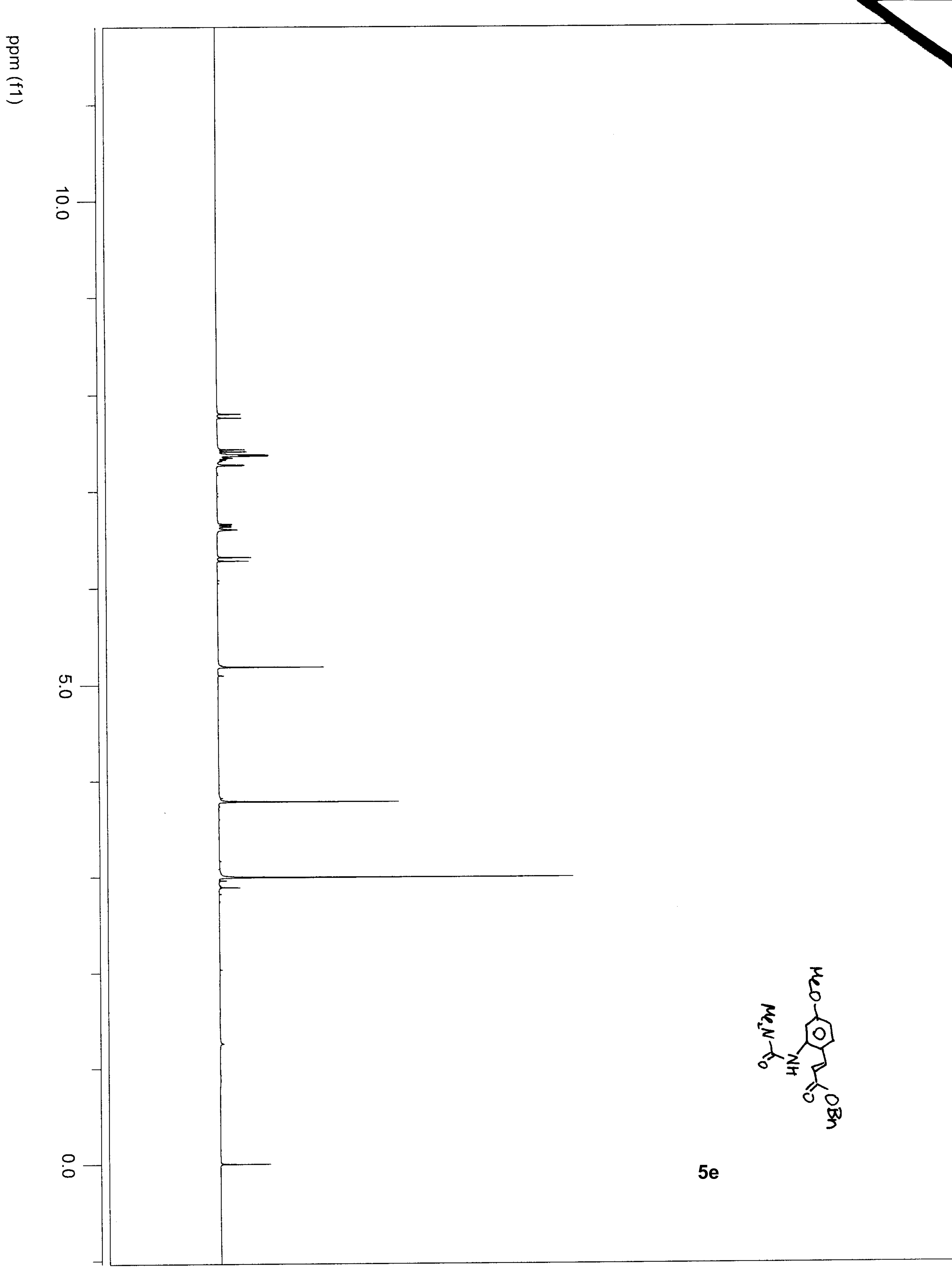
ppm (f1)
10.0
5.0
0.0
5e

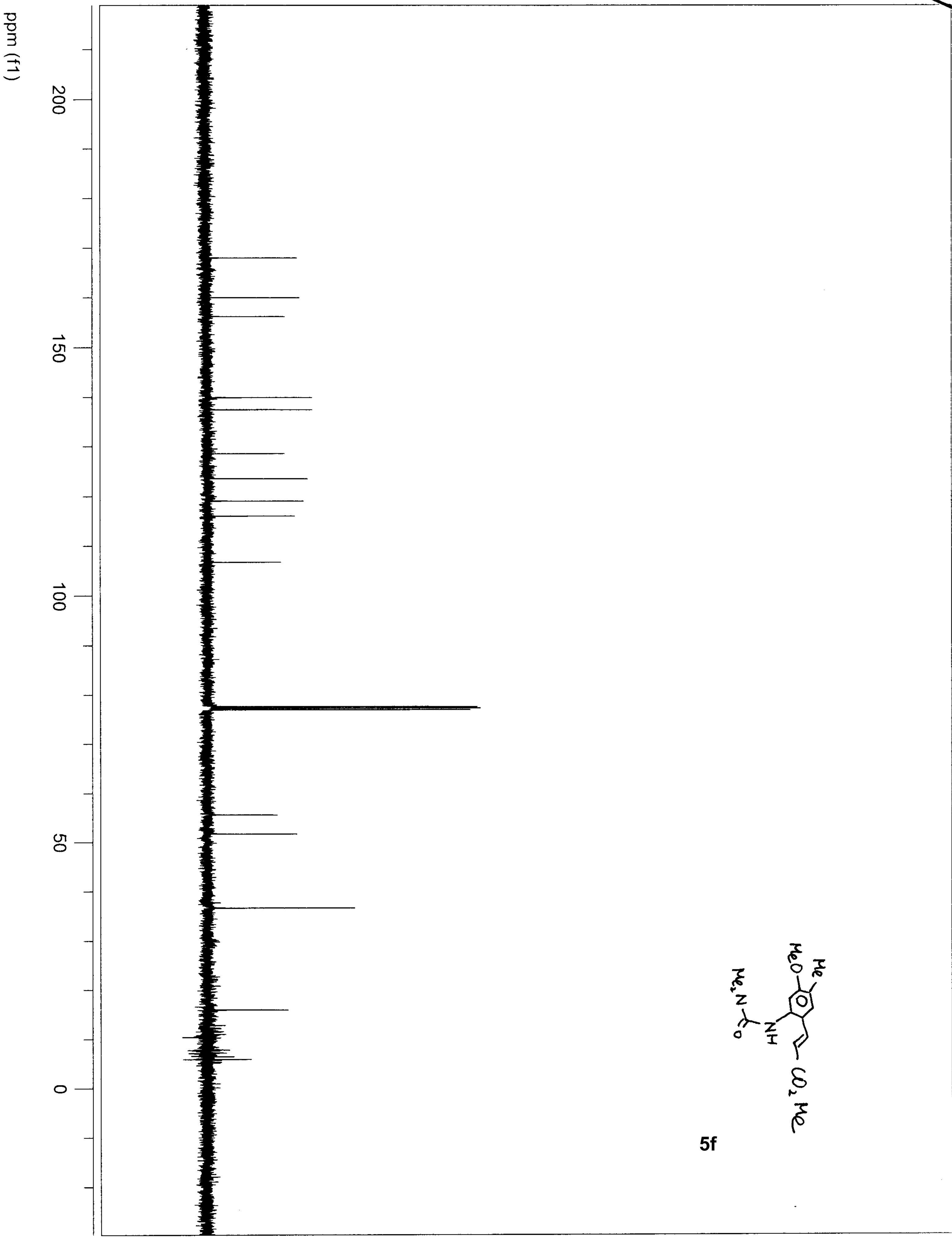

5f

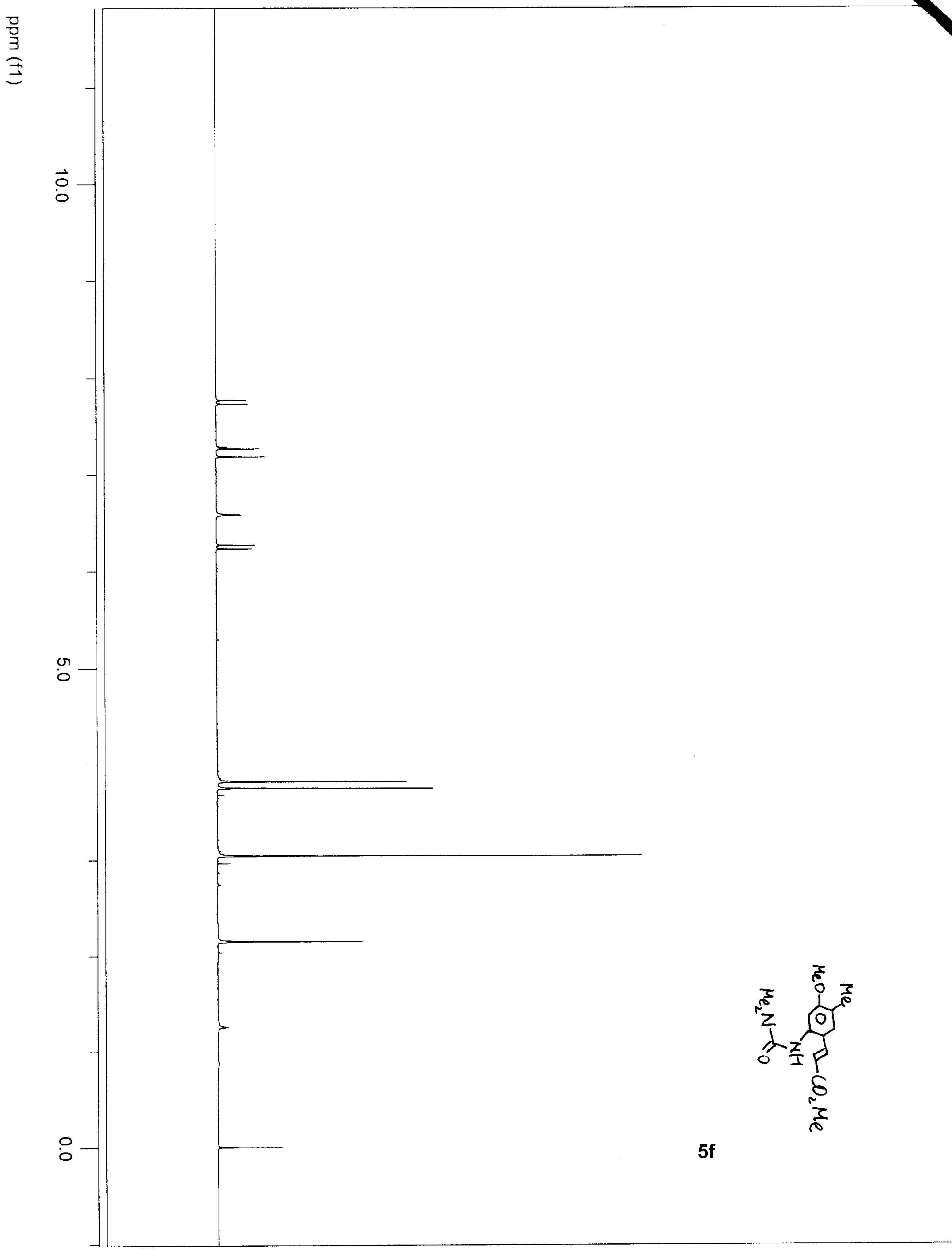

5f

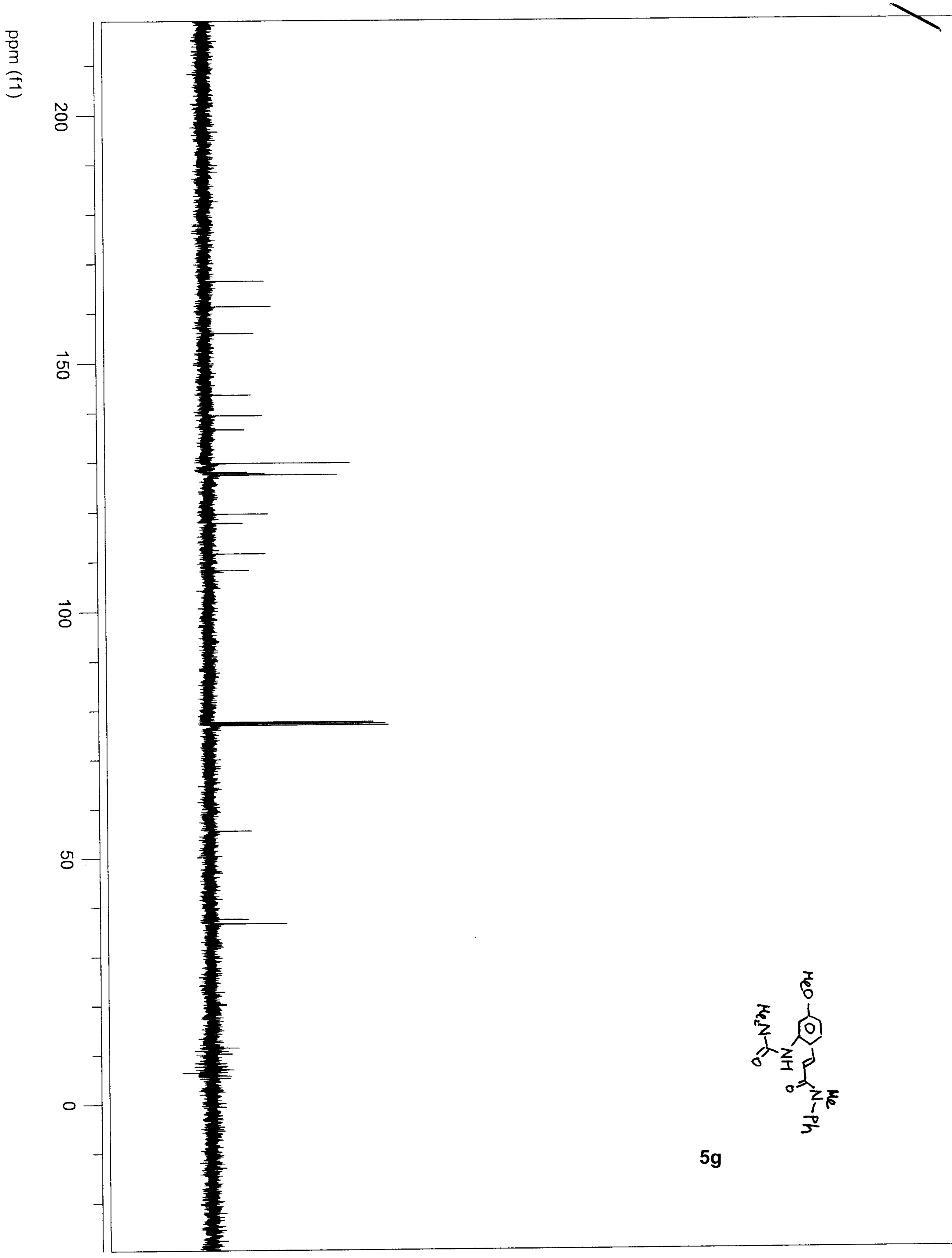
ppm (f1)
200
150
100
50
0
5g

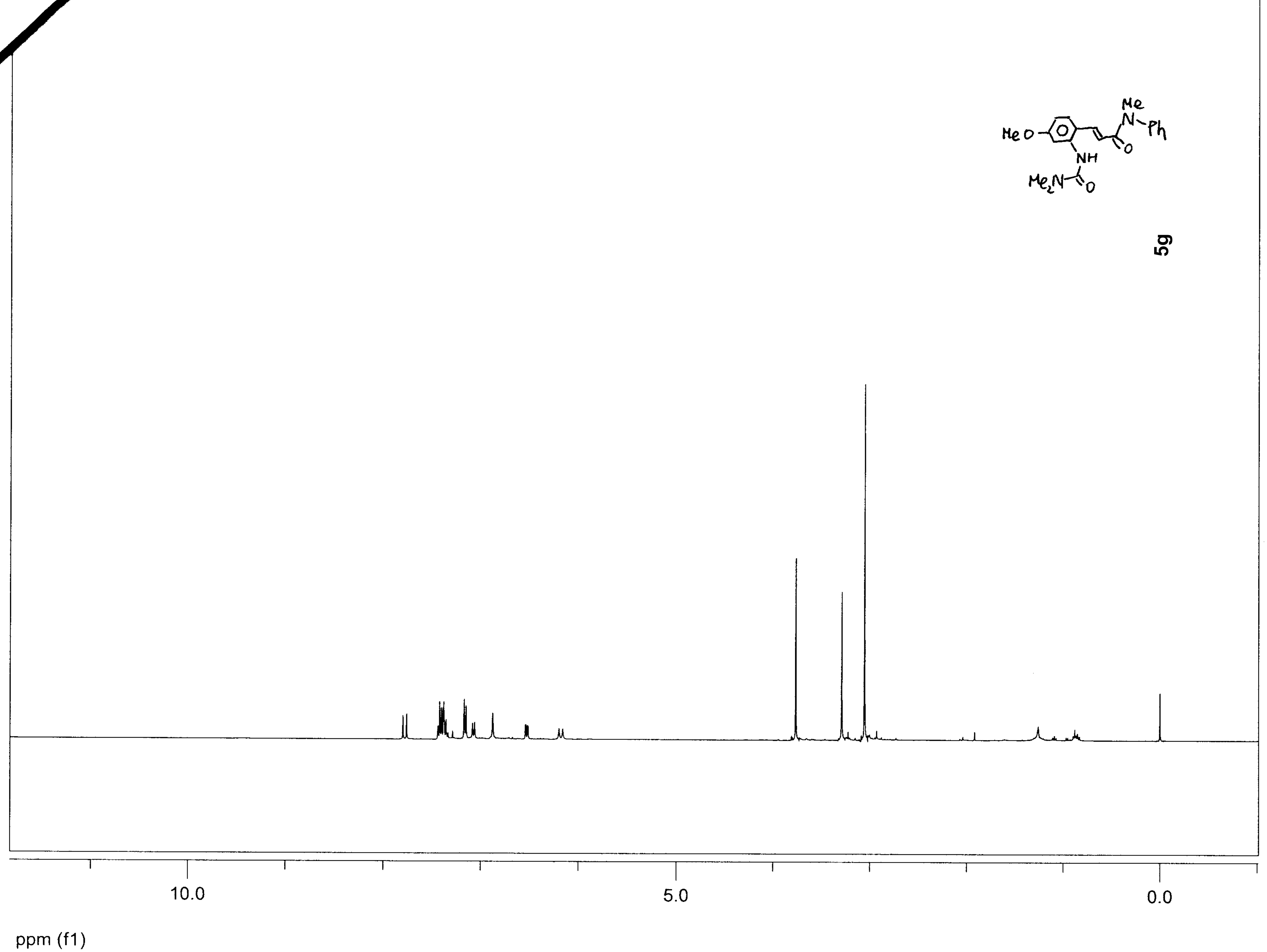
MeO
NH
Me2N
O
Me
N
Ph
O
5g
10.0
5.0
0.0
ppm (f1)

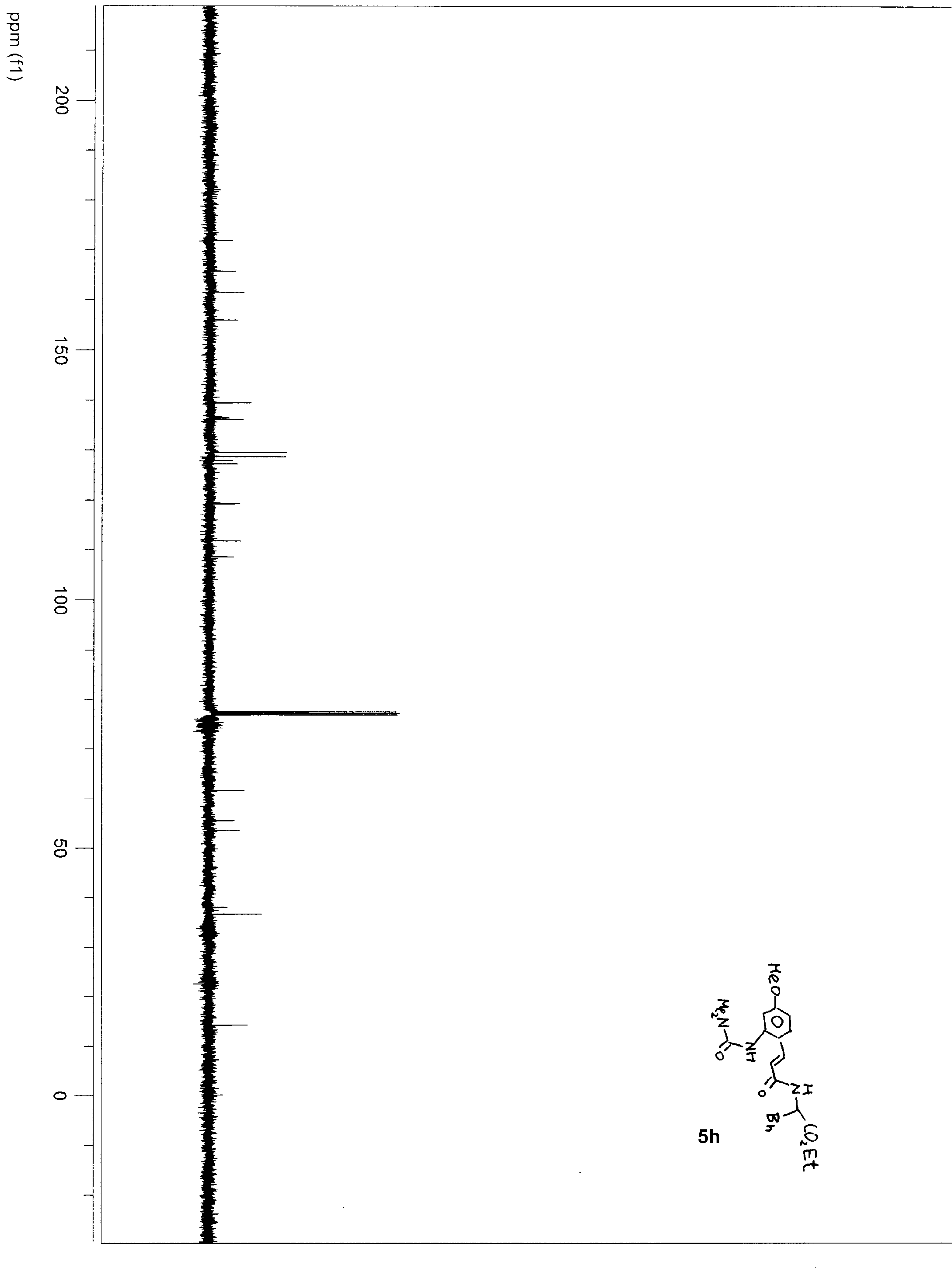
ppm (f1)
200
150
100
50
0
5h

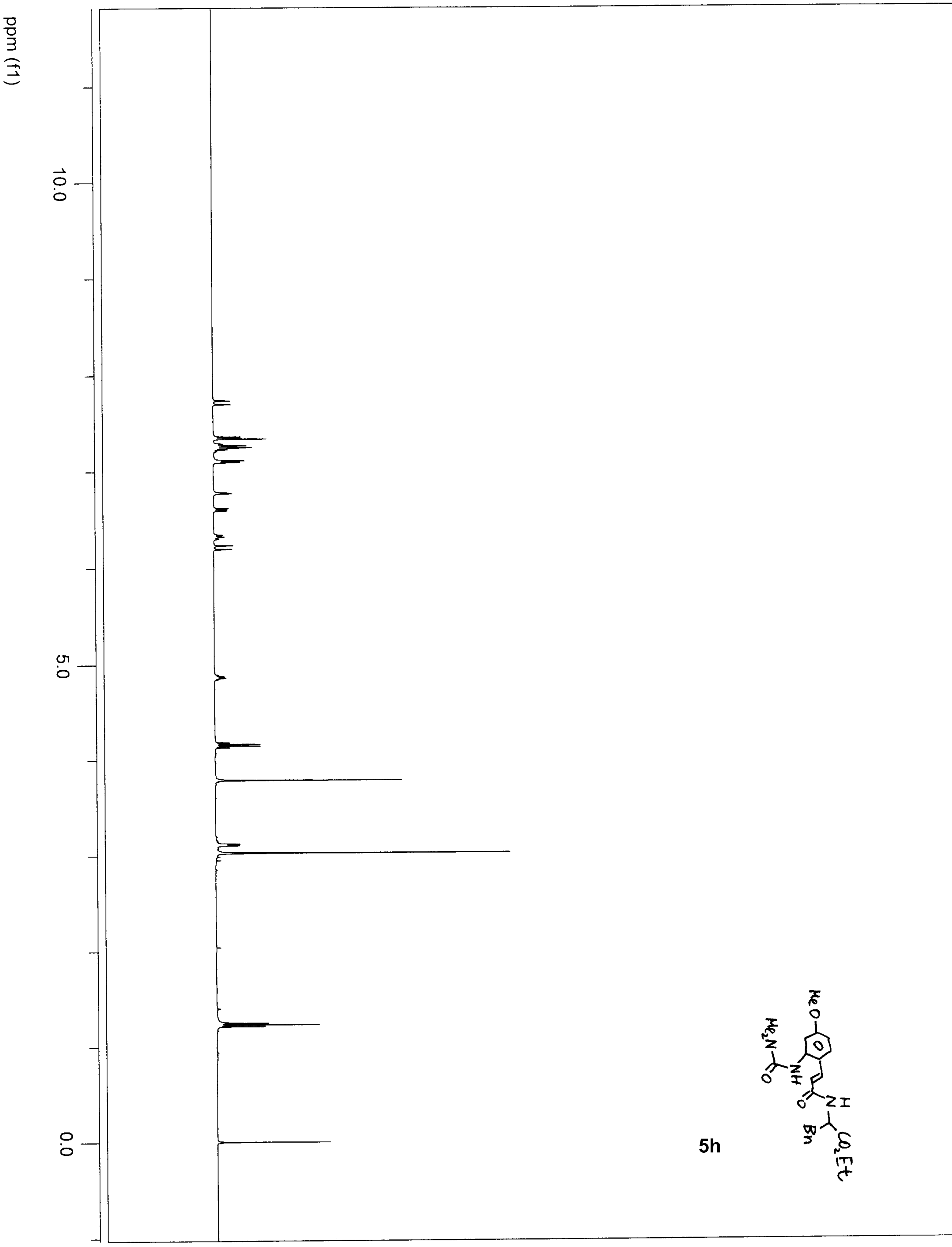
ppm (f1)
10.0
5.0
0.0
5h

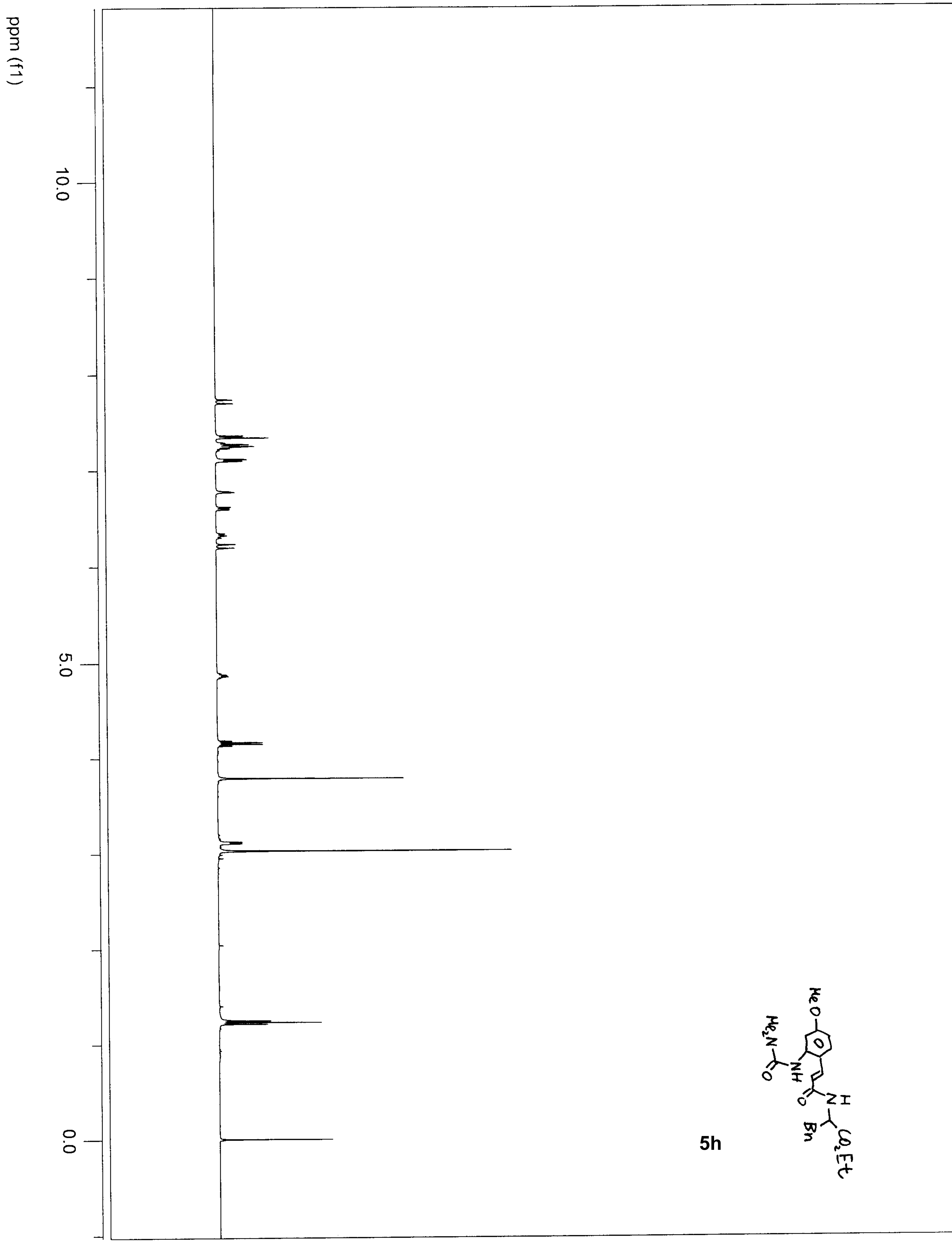
ppm (f1)
10.0
5.0
0.0
5h

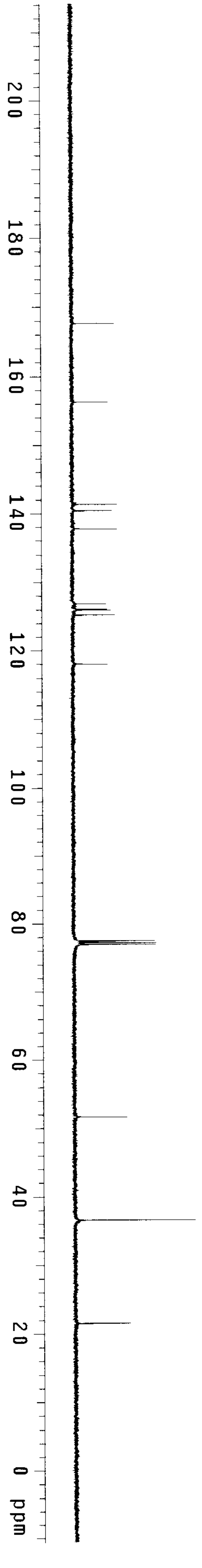

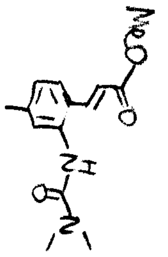

5i

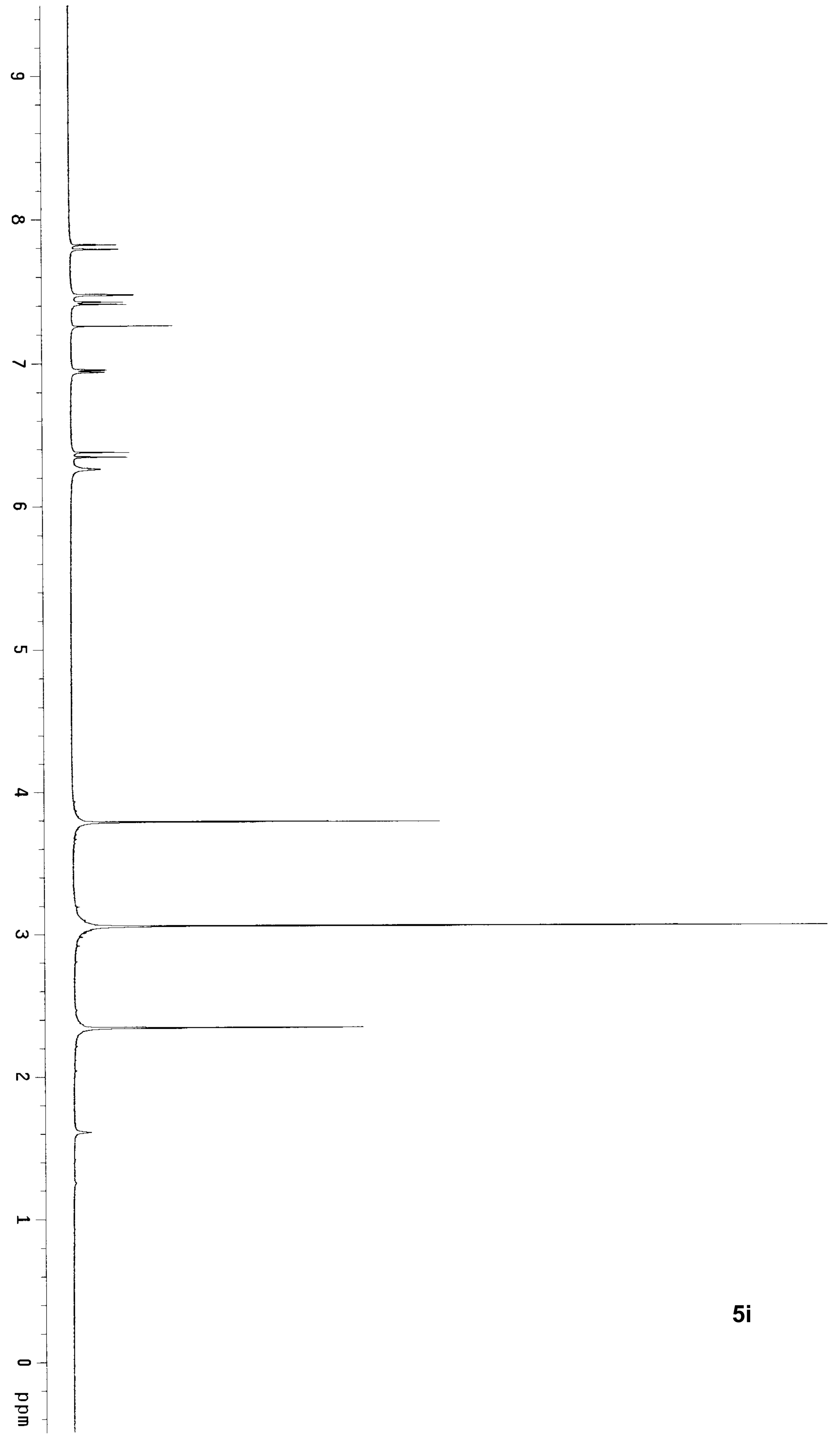

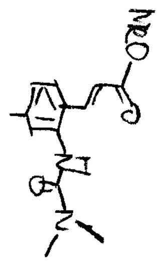

5i

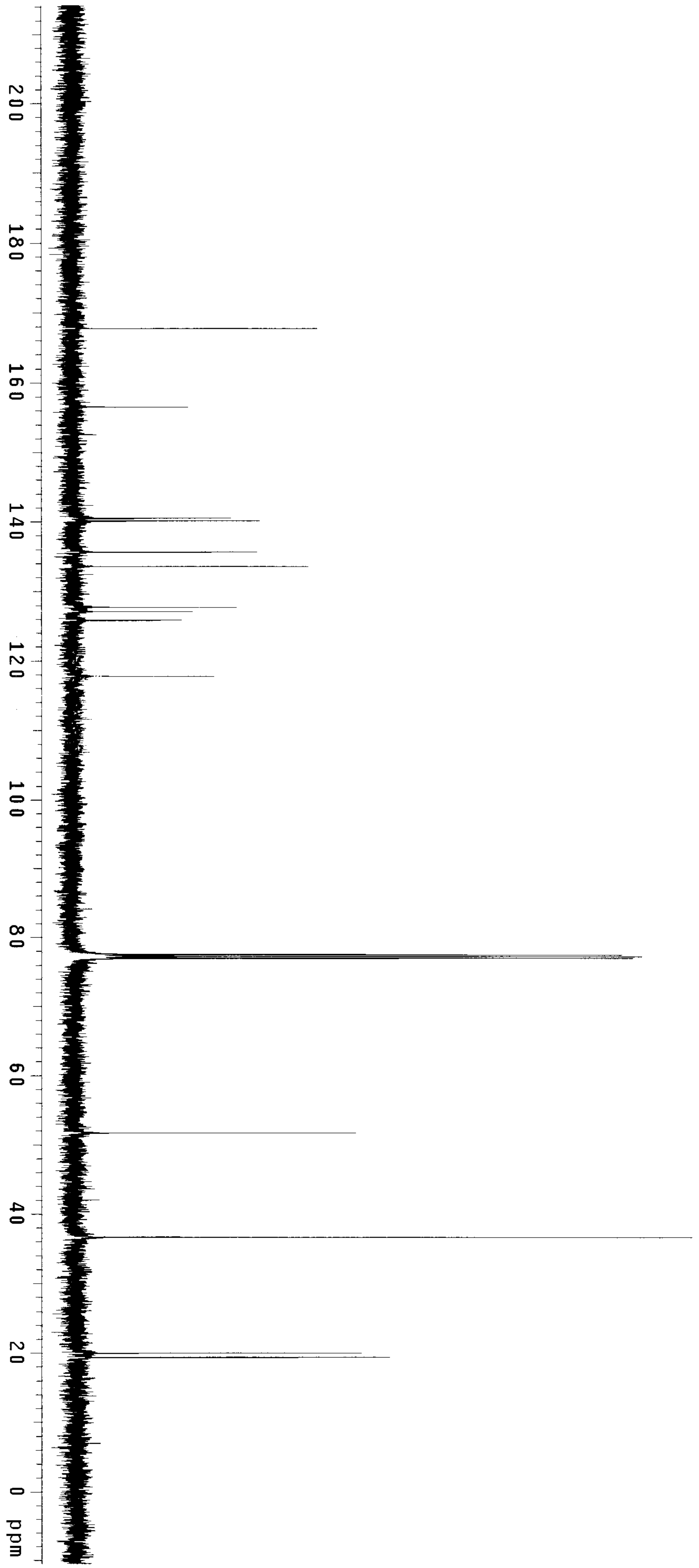

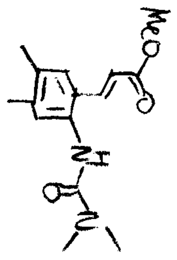

5j

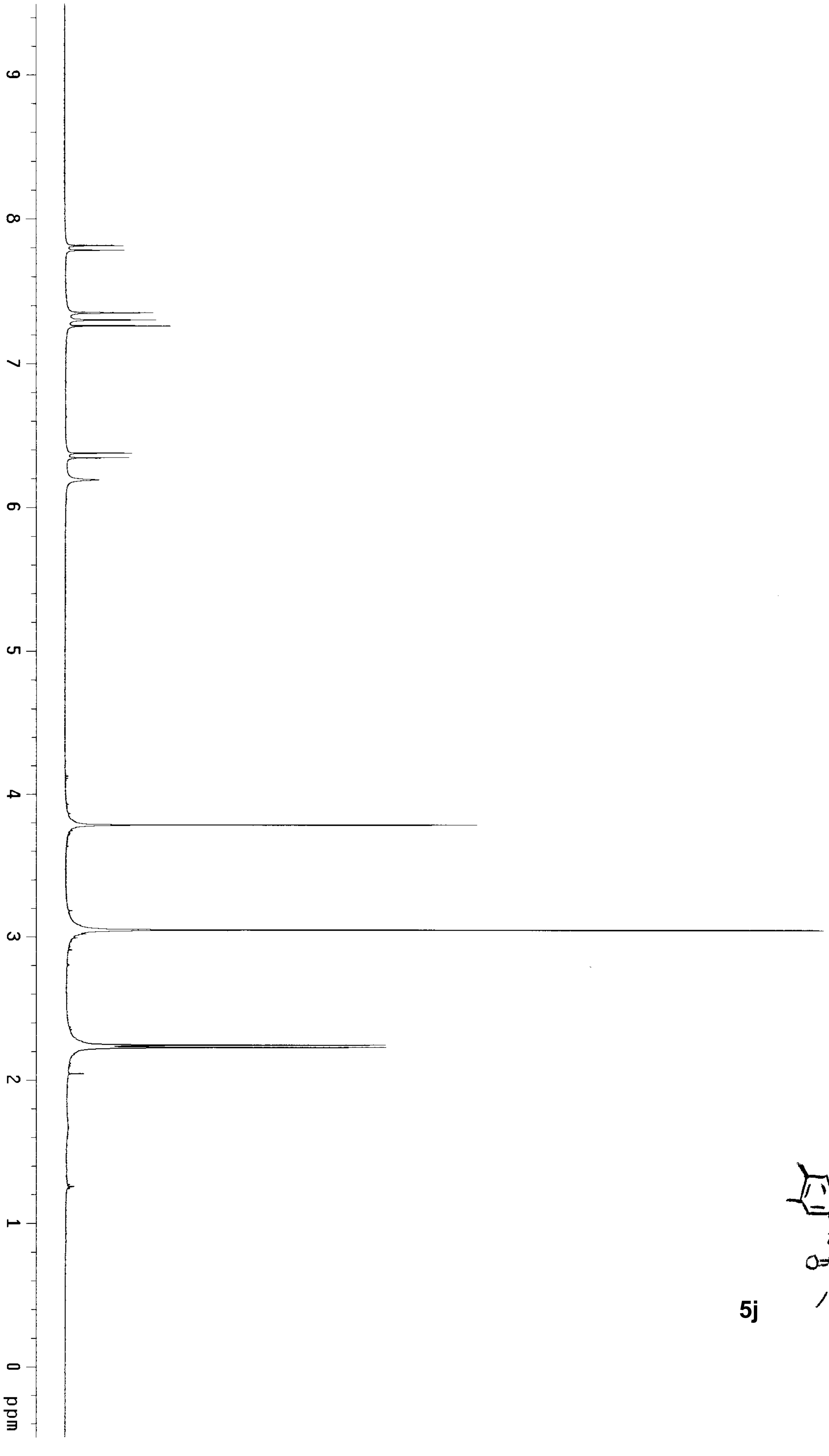

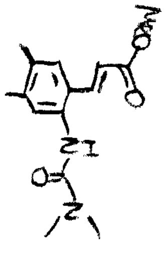

5j

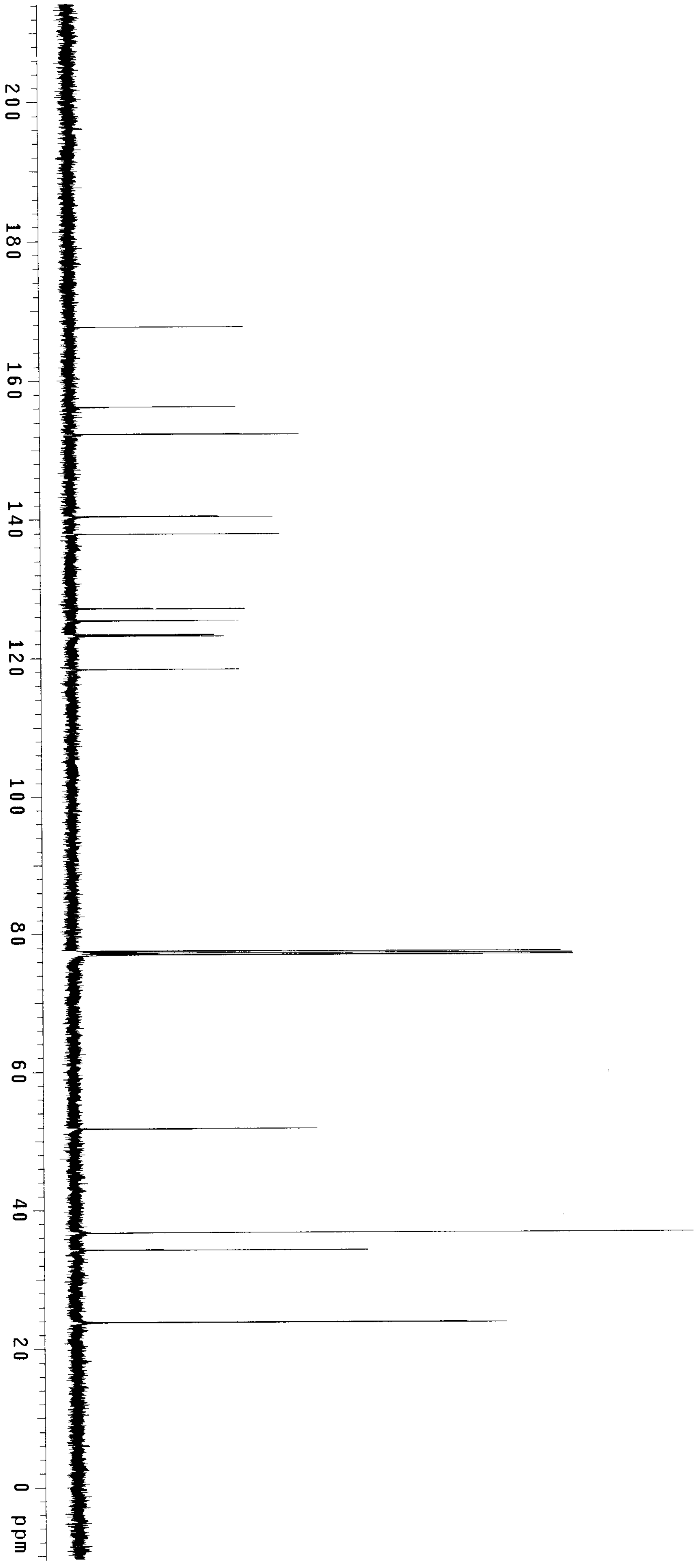

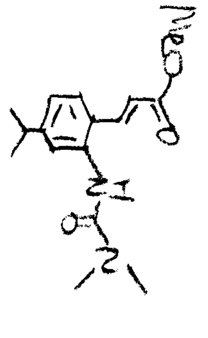

5k

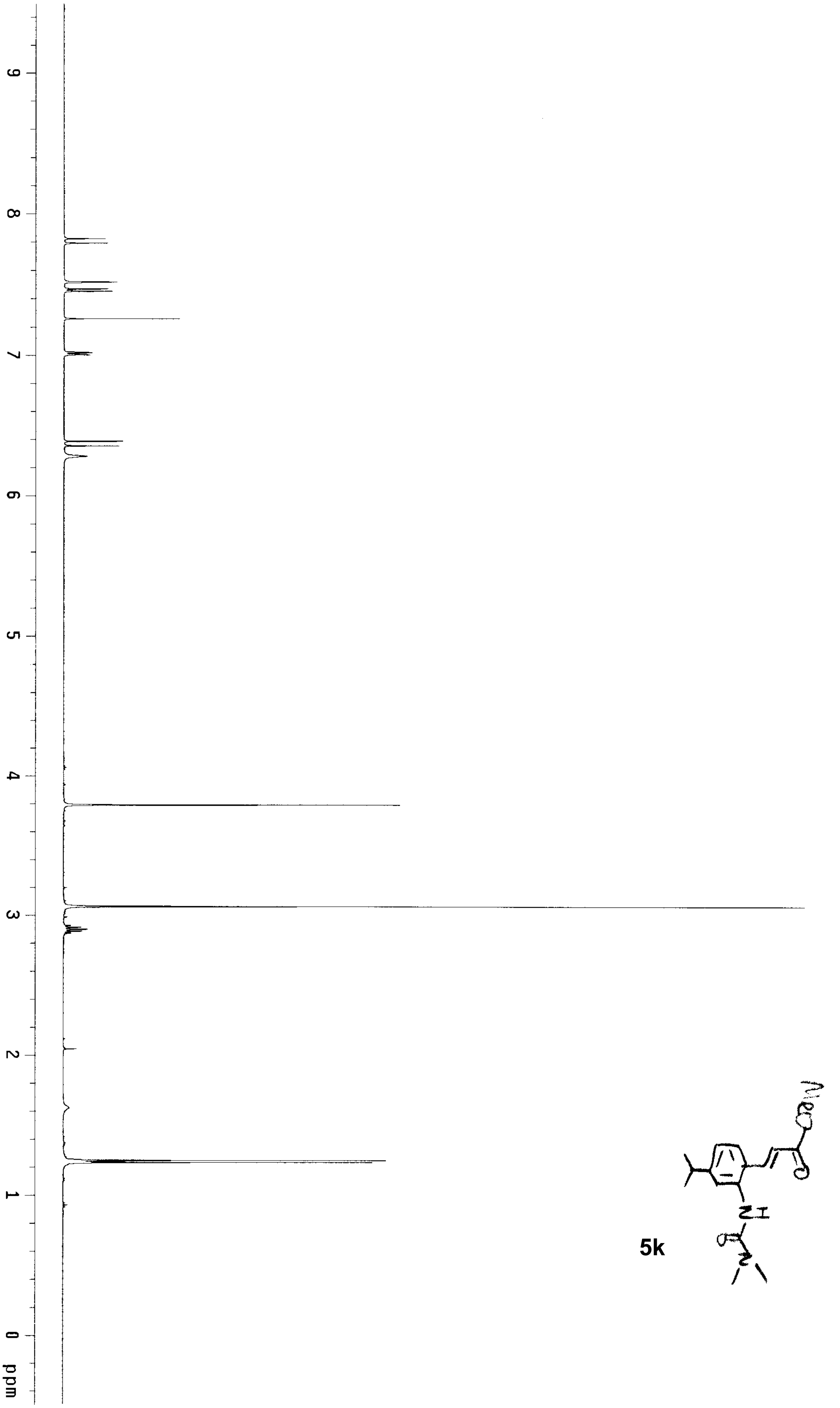

5k

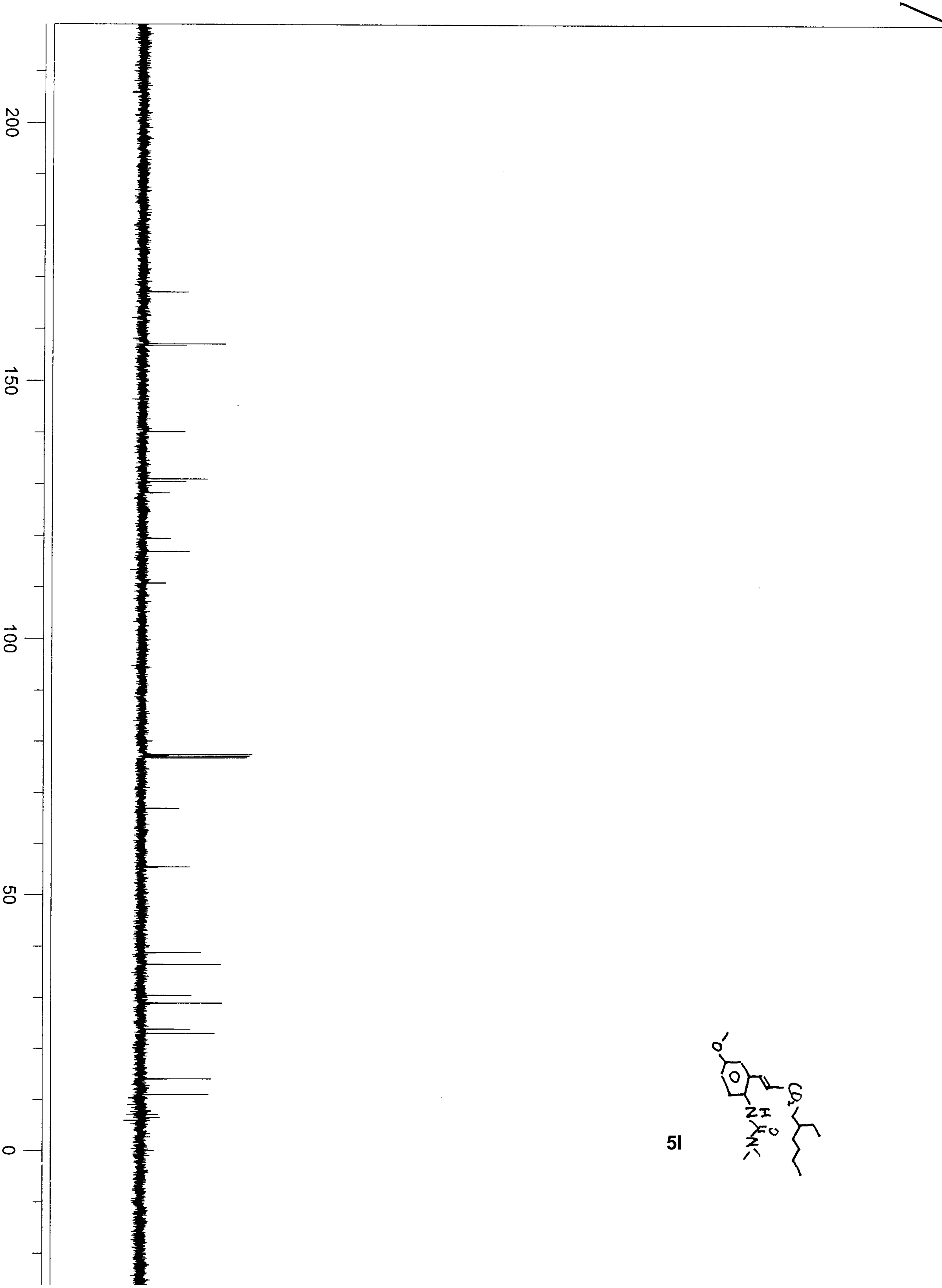
200
150
100
50
0
5l

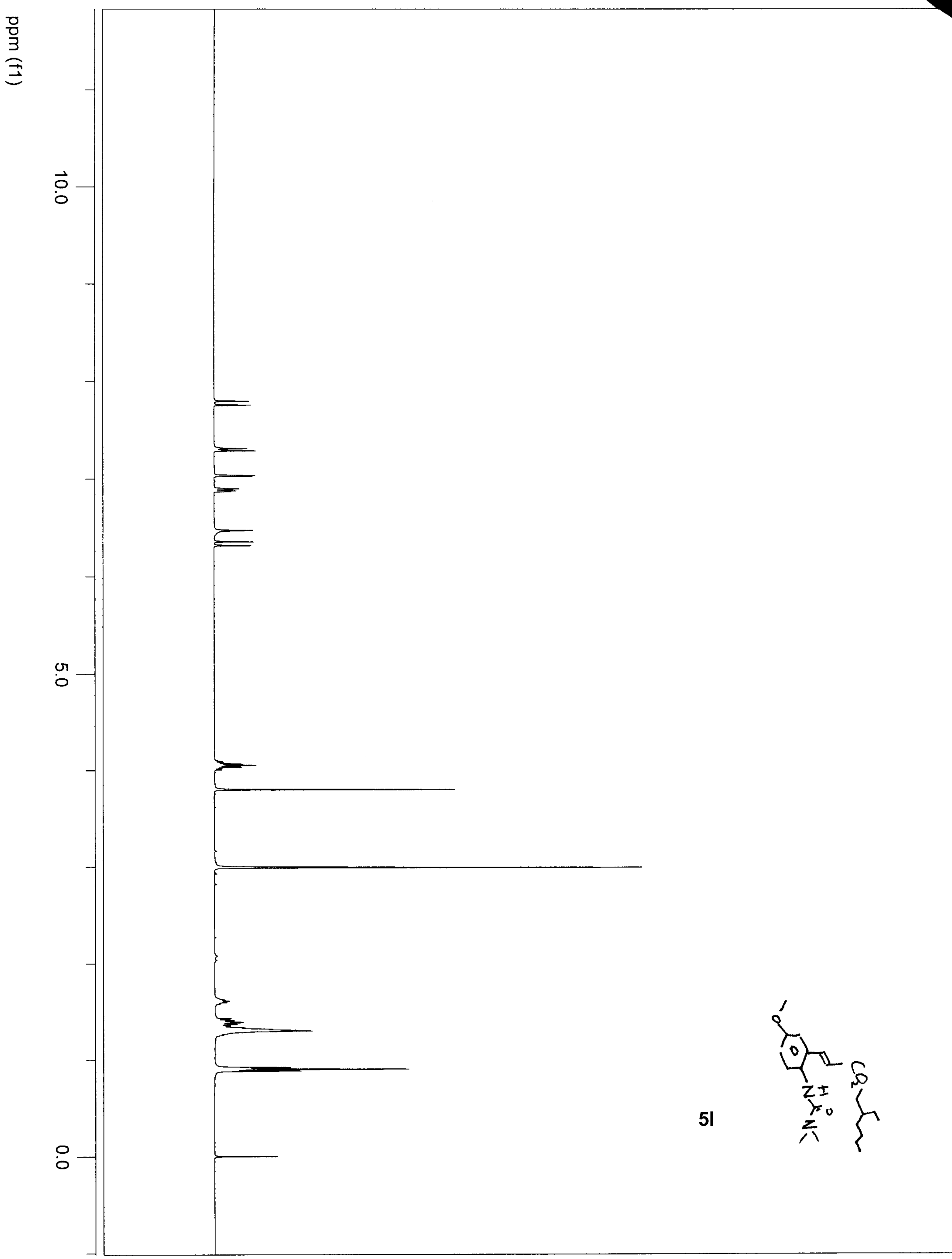
ppm (f1)
10.0
5.0
0.0
5l

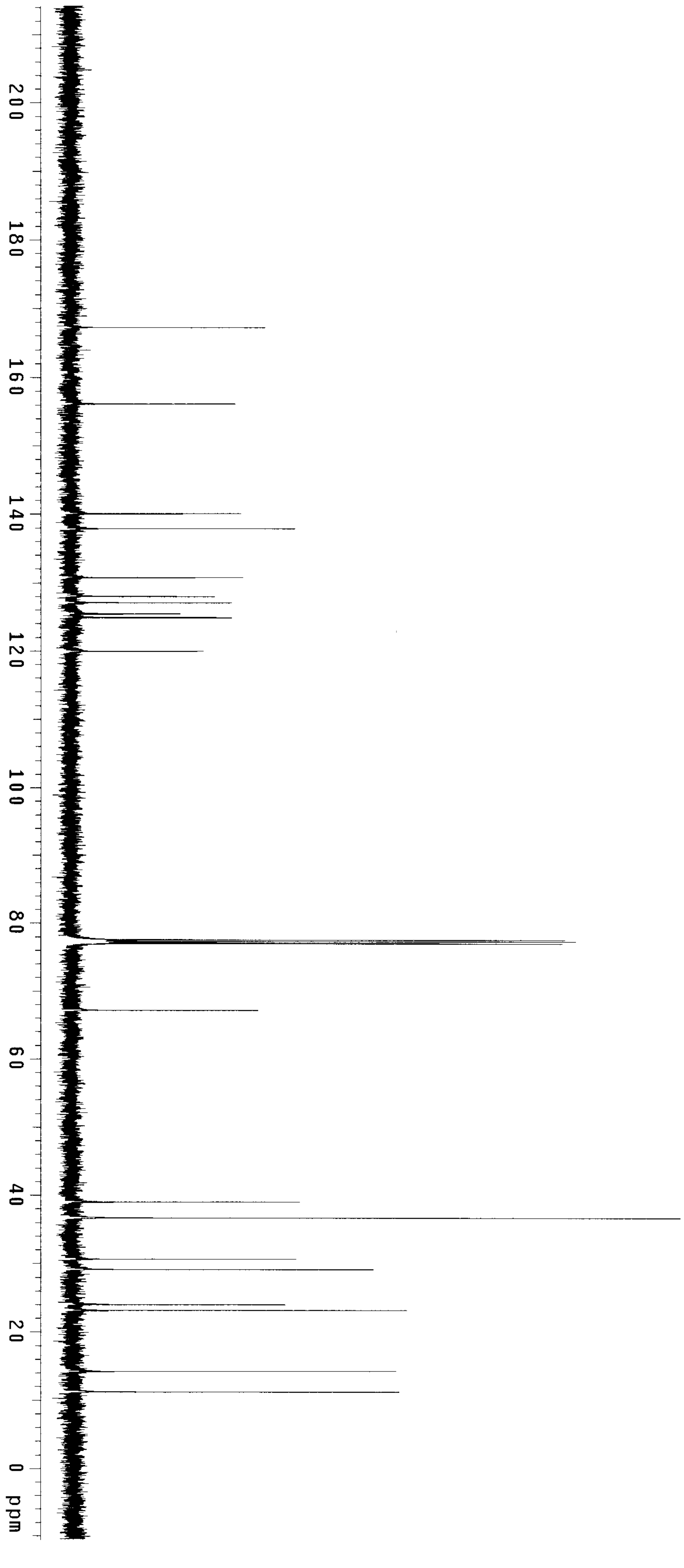

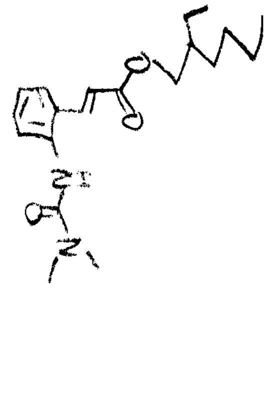

5m

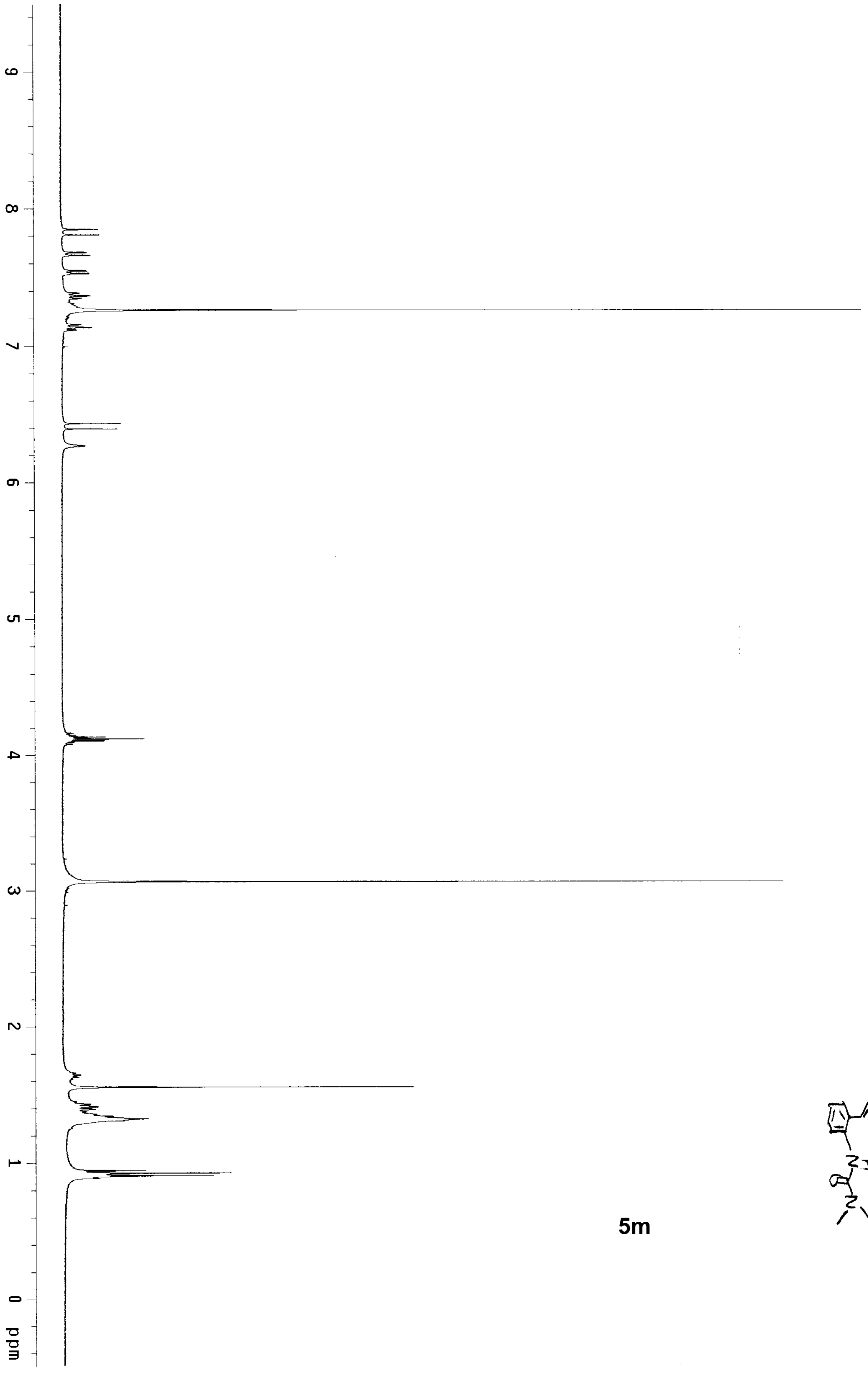

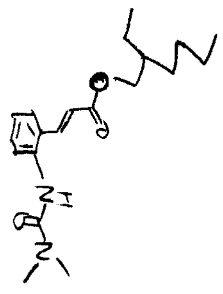

5m

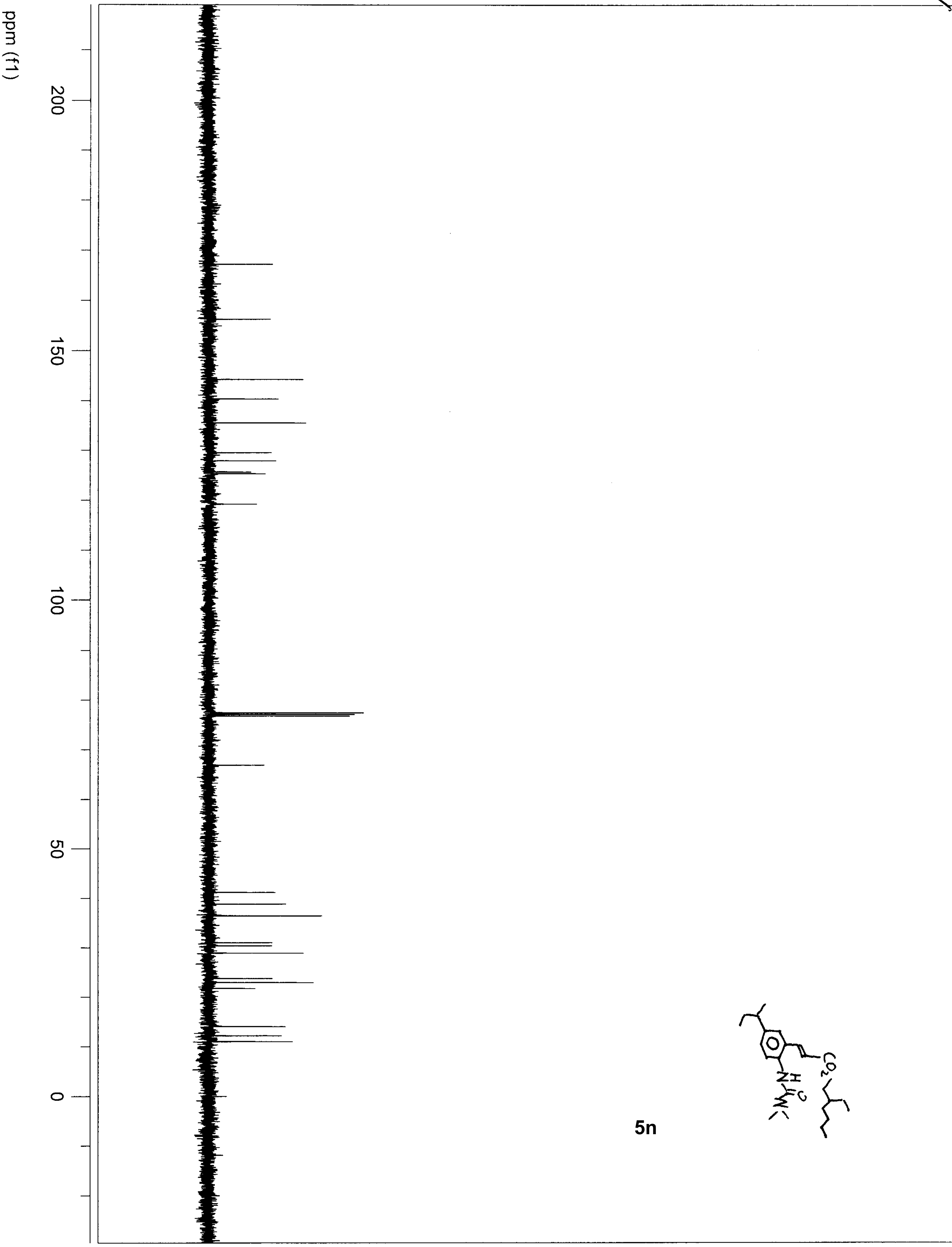
ppm (f1)
200
150
100
50
0
5n

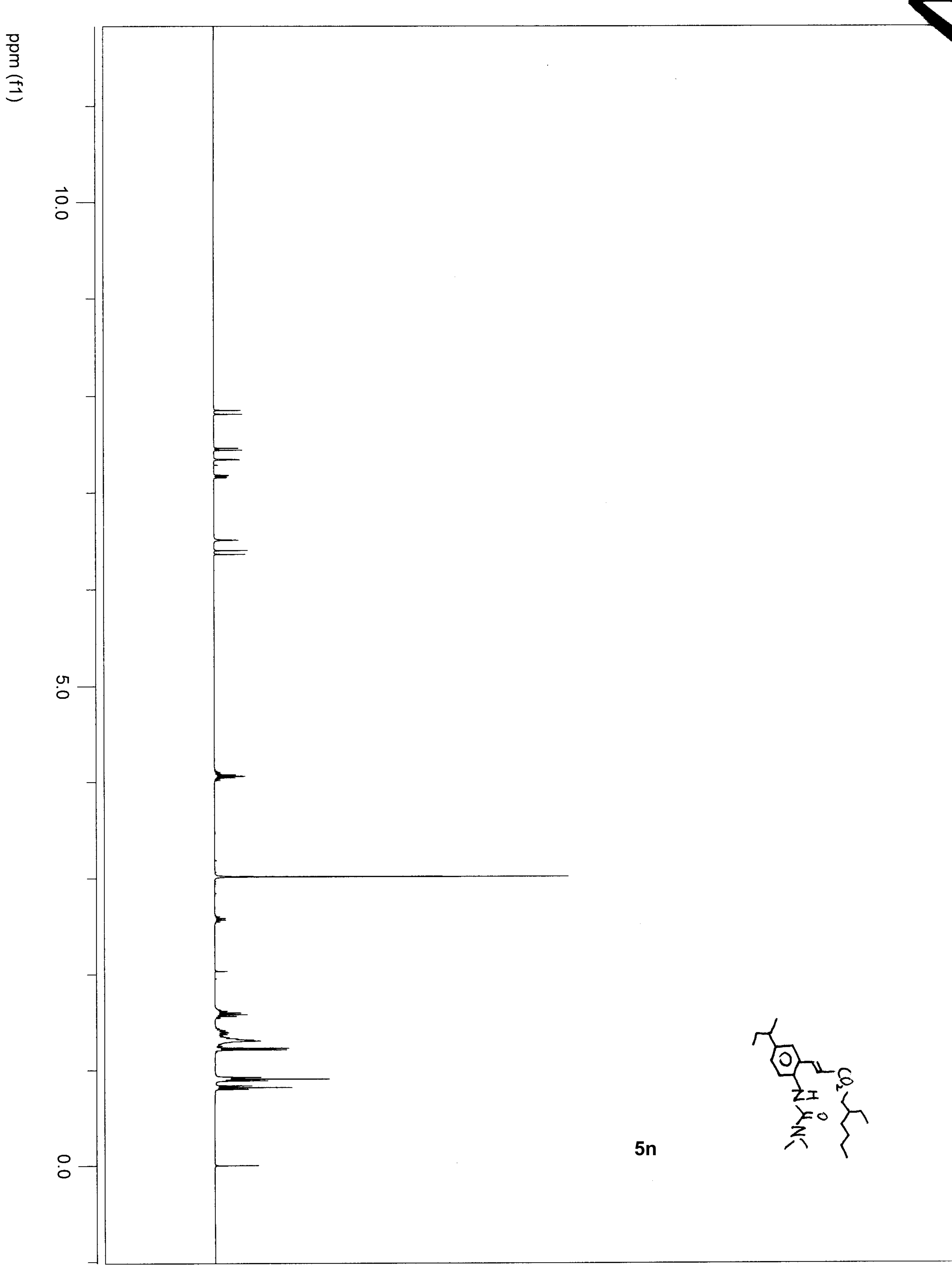
ppm (f1)
10.0
5.0
0.0
5n

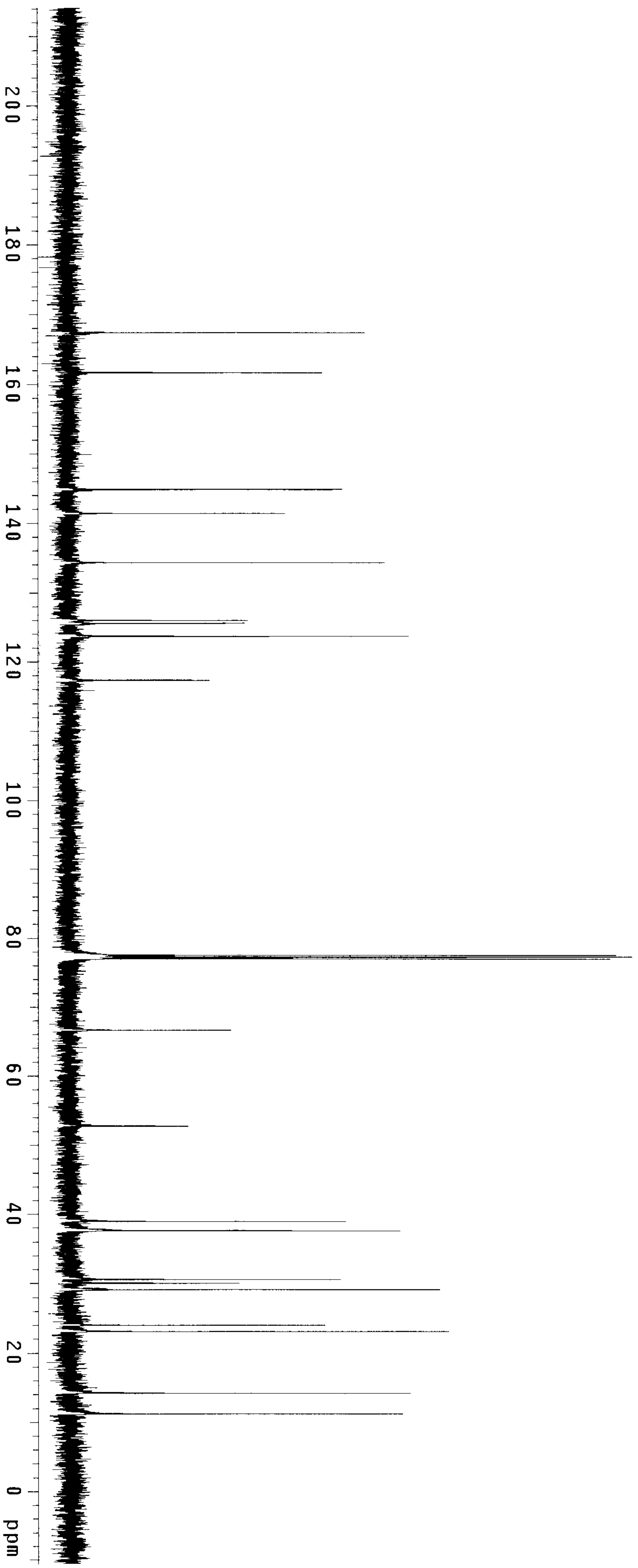

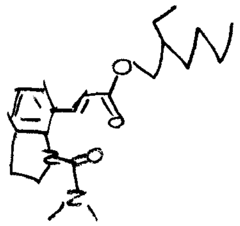

5o

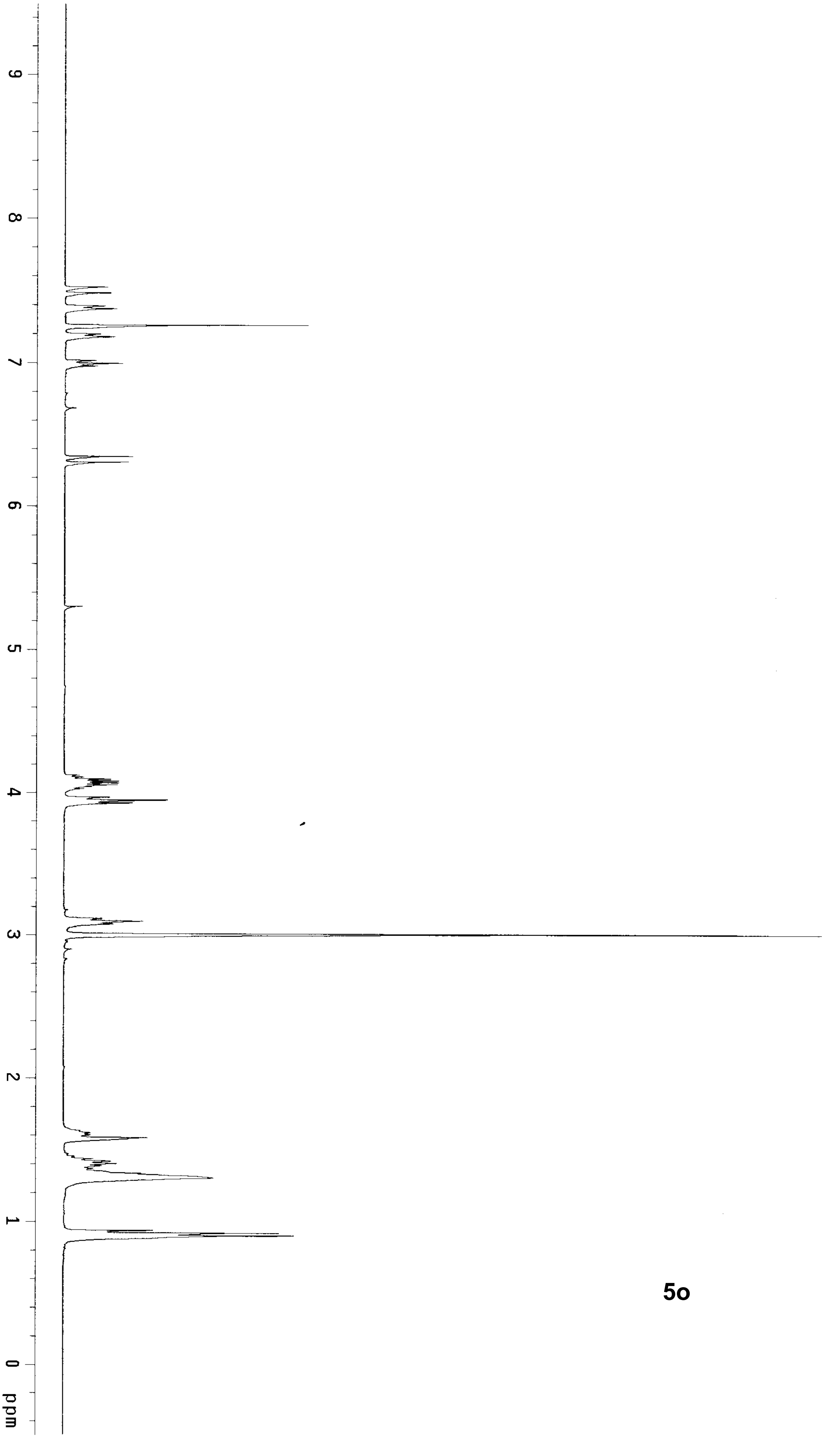

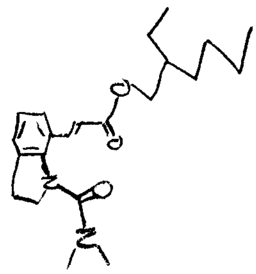

5o

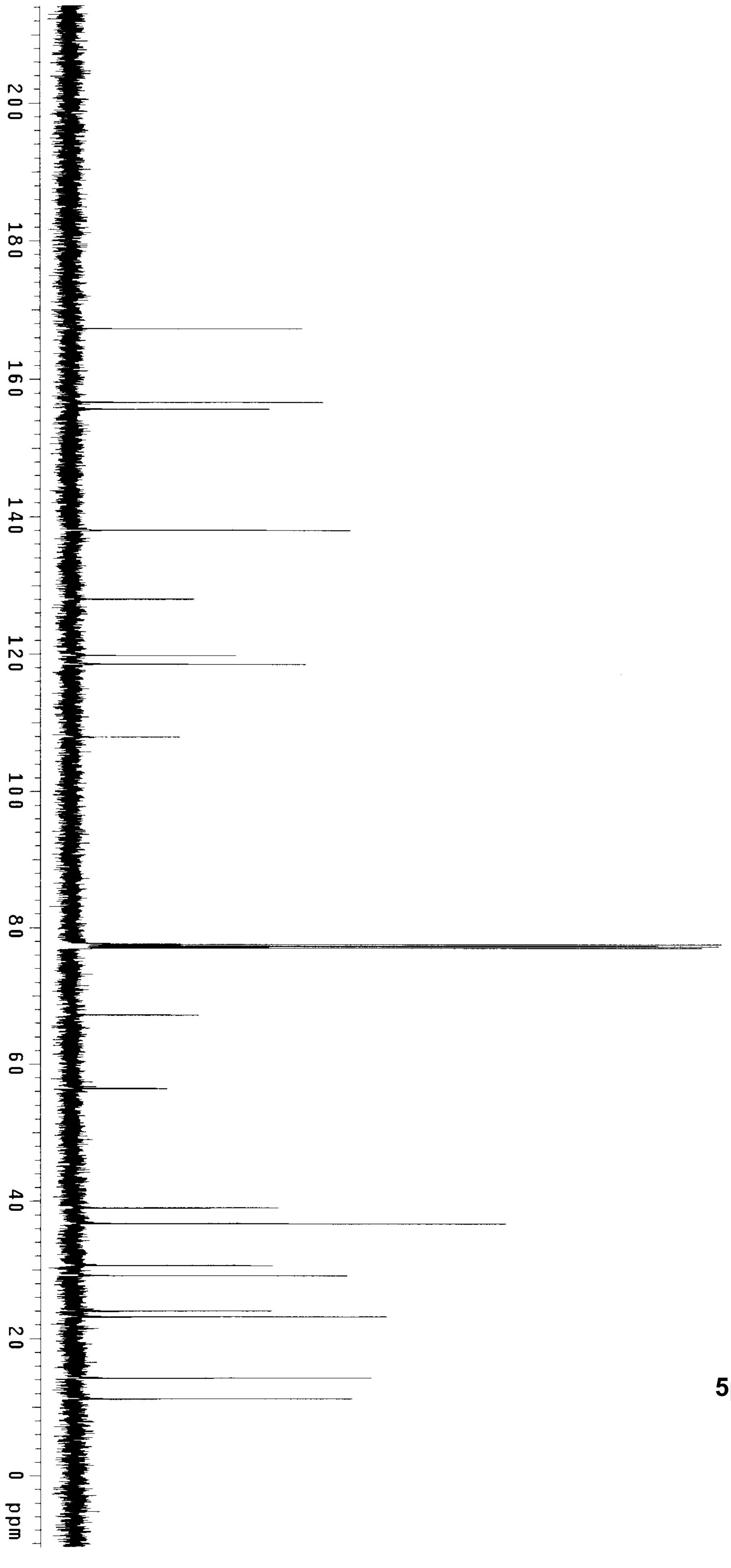

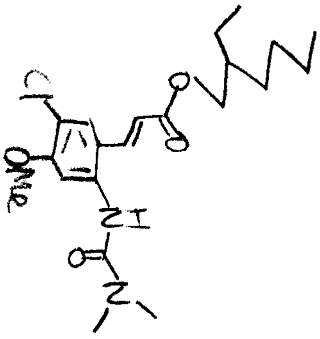

5p

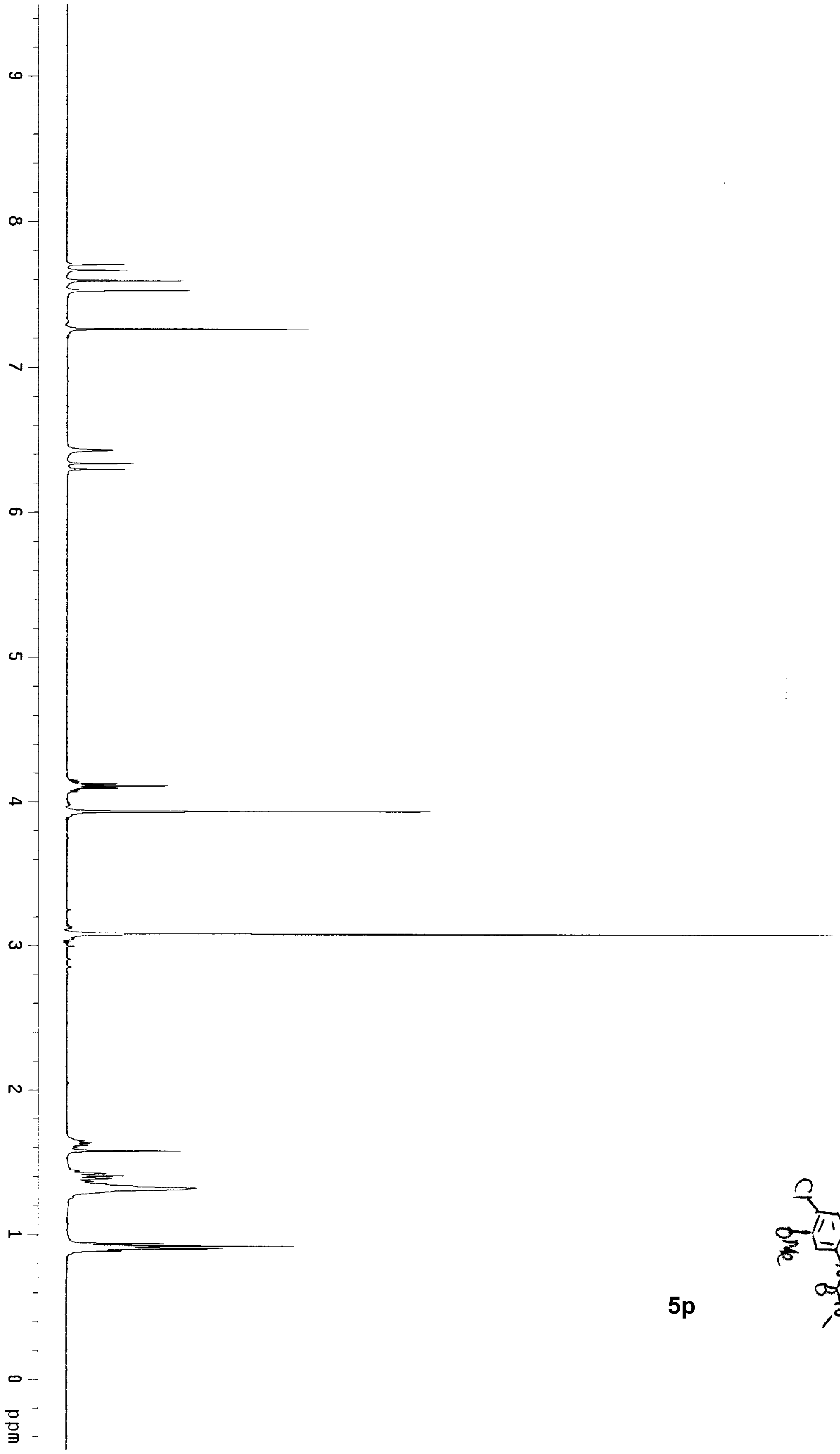

5p

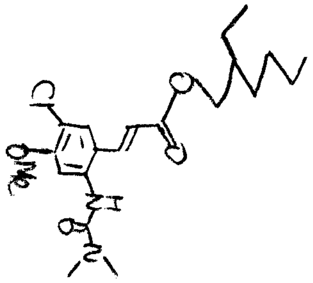

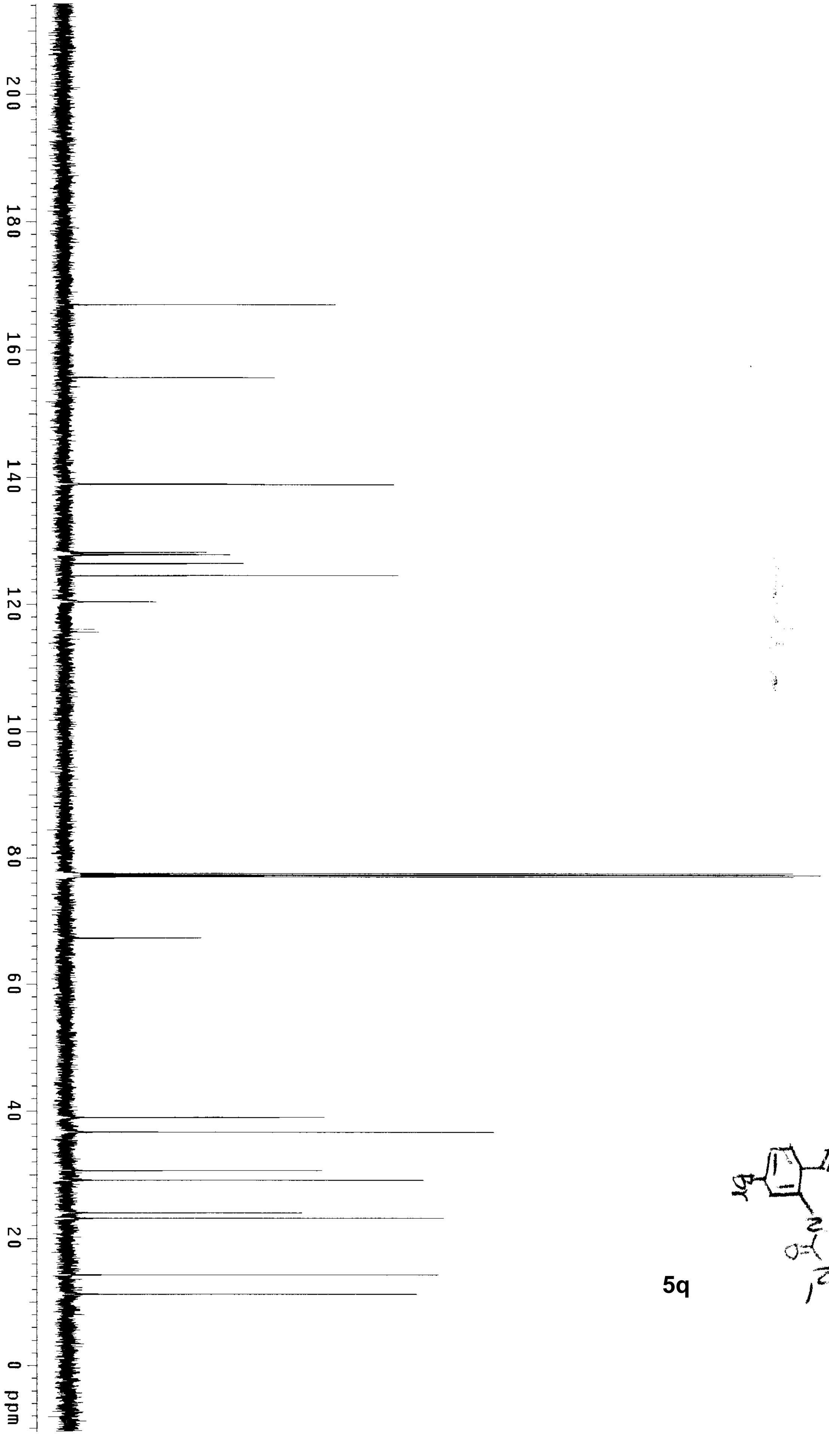

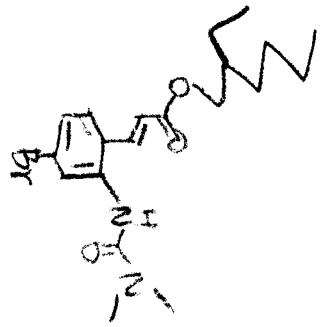

5q

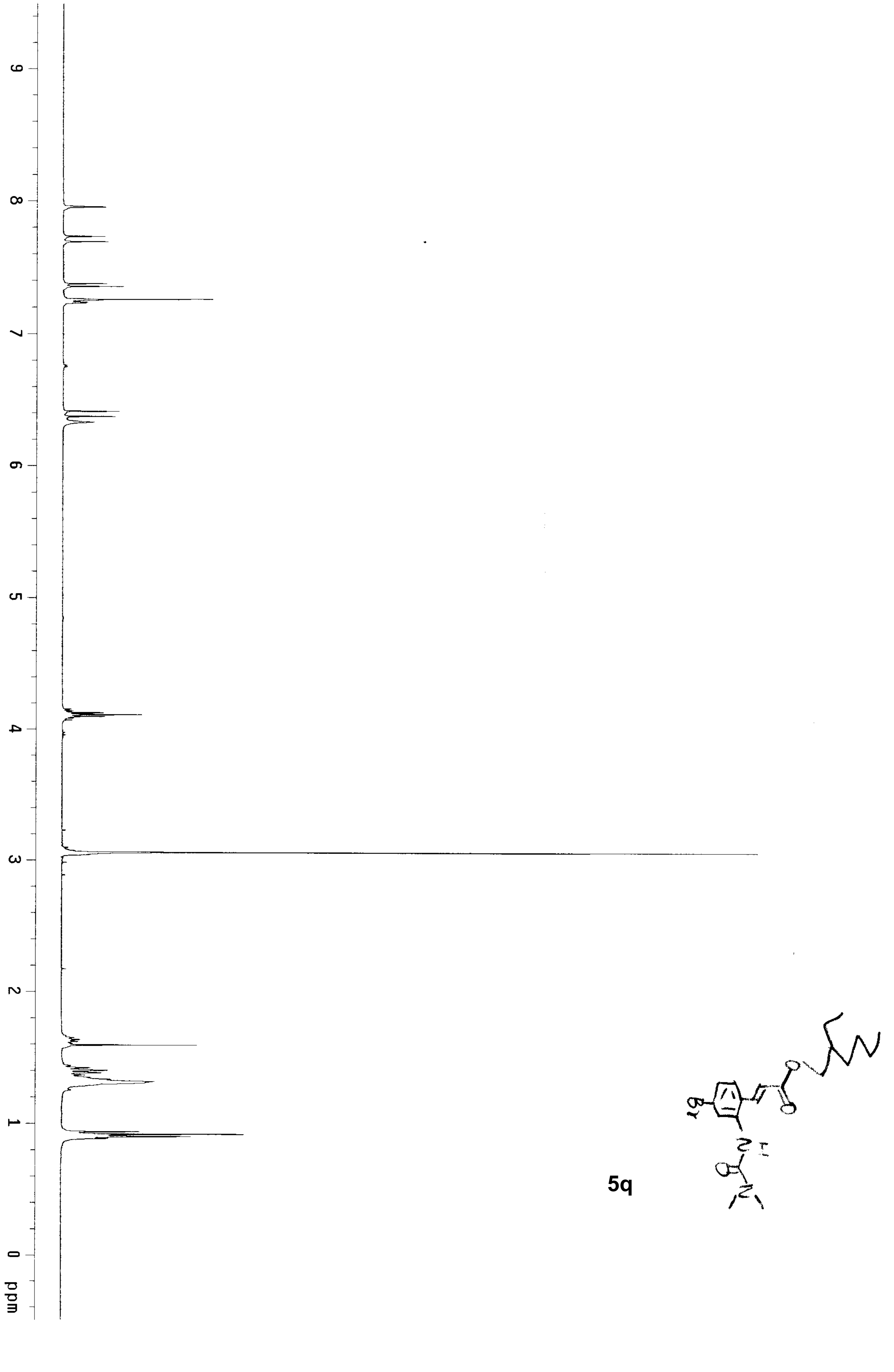

5q

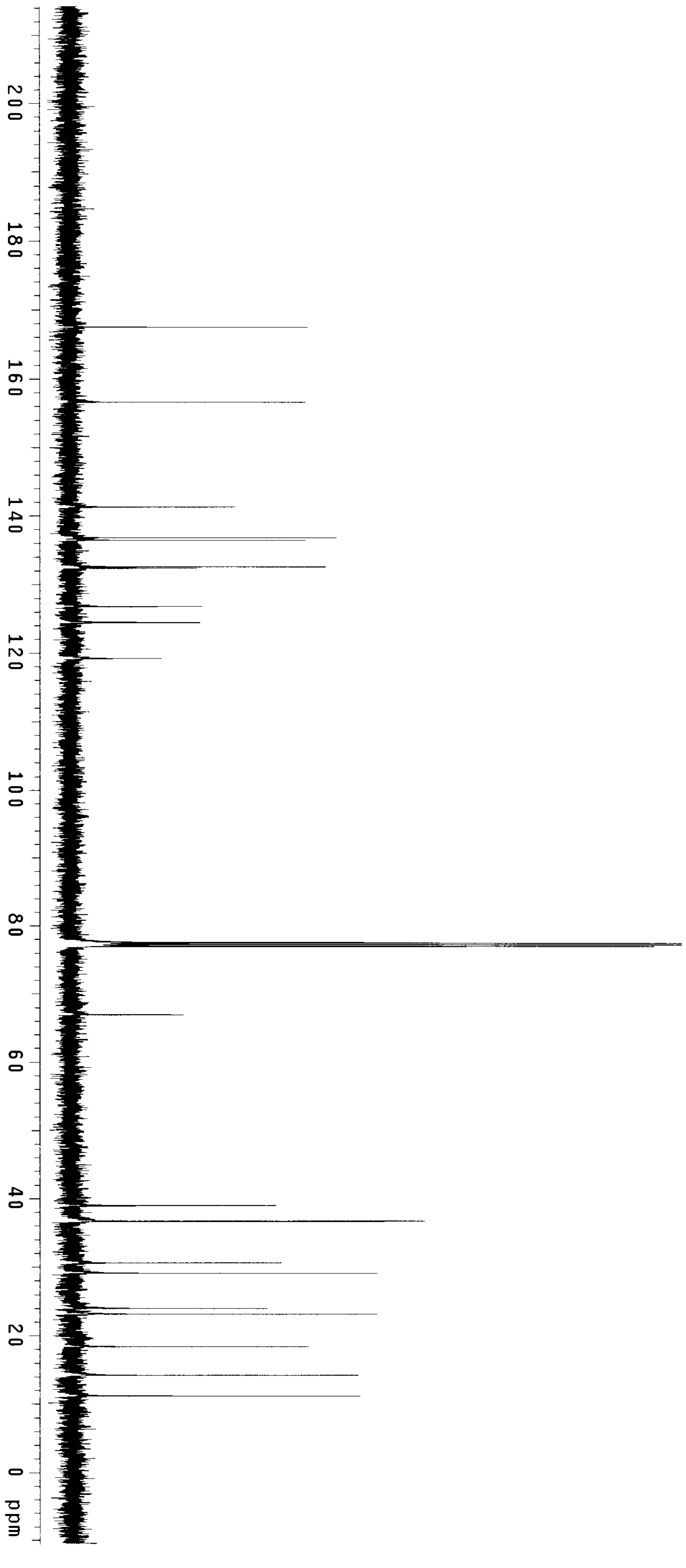

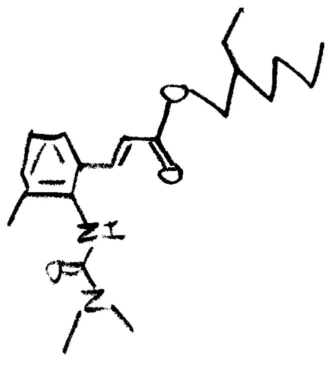

5r

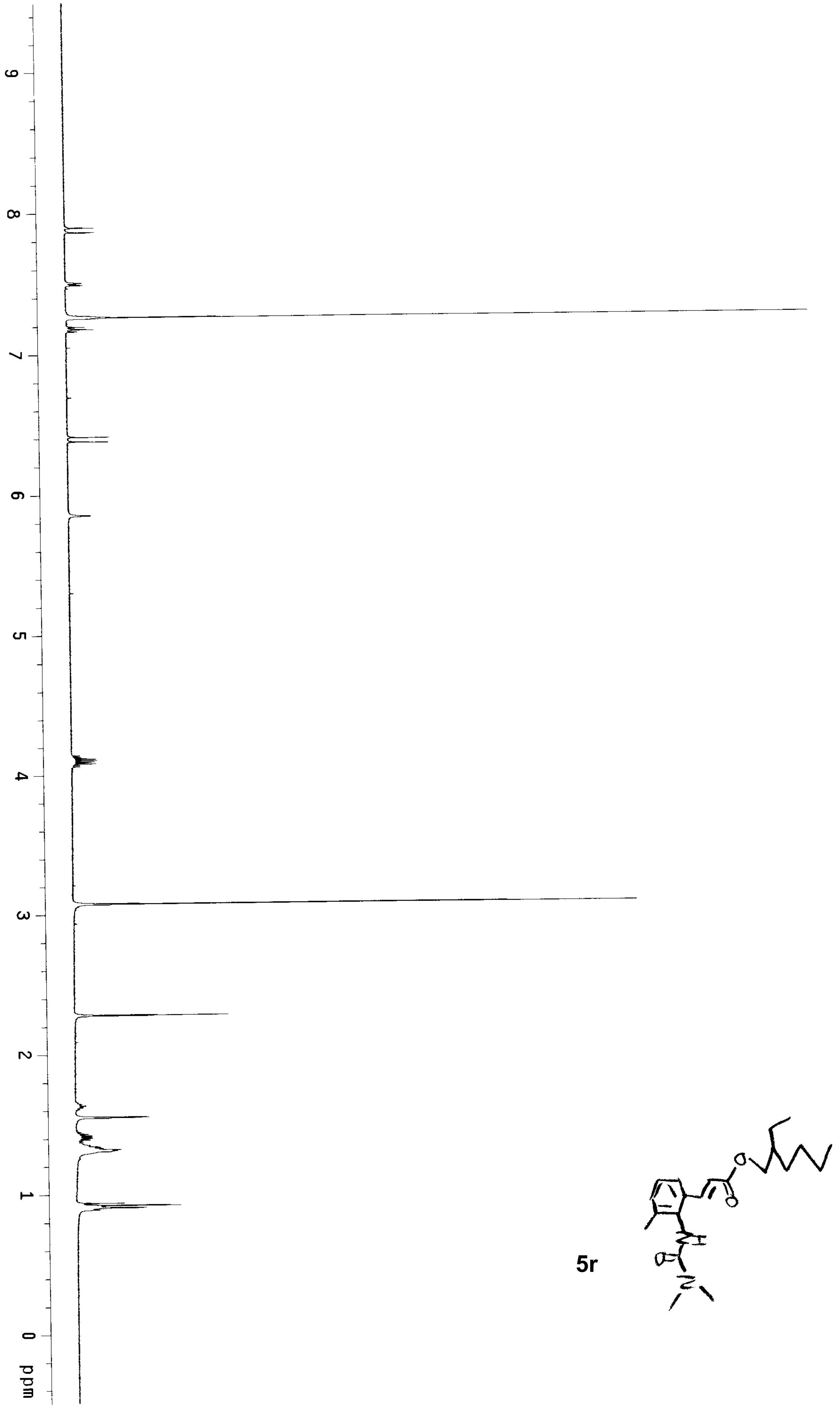

5r

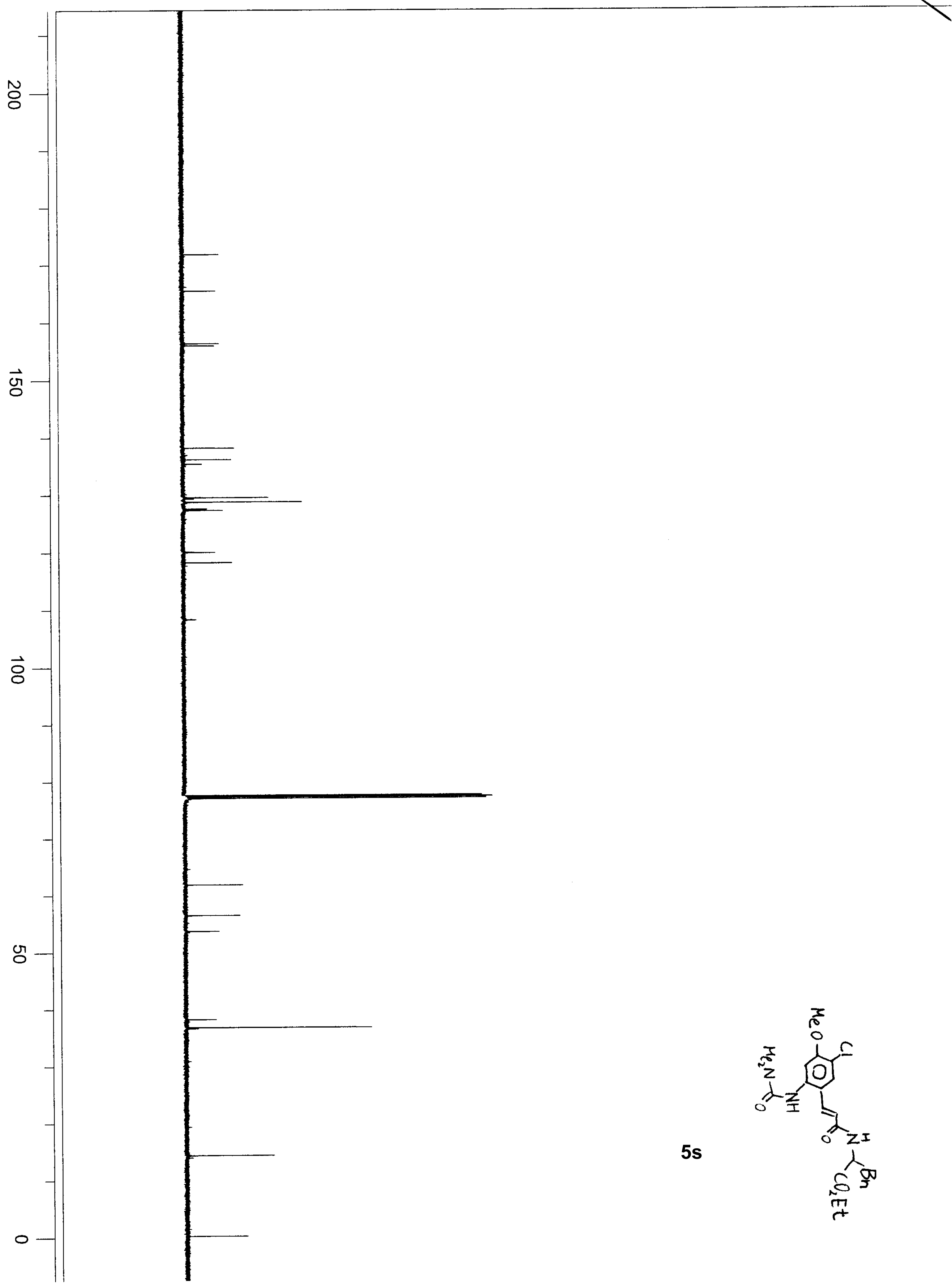

5s

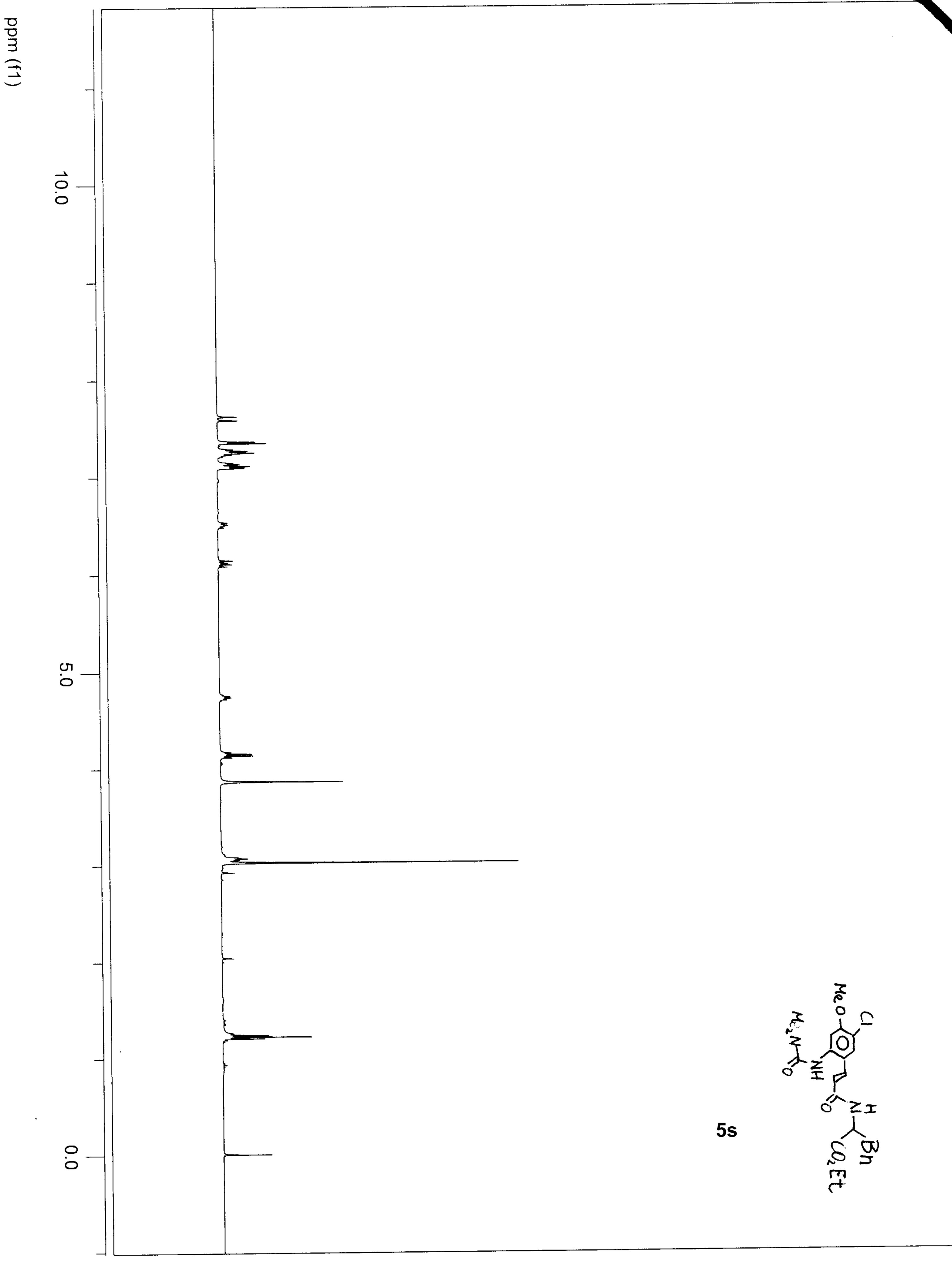
ppm (f1)
10.0
5.0
0.0
5s

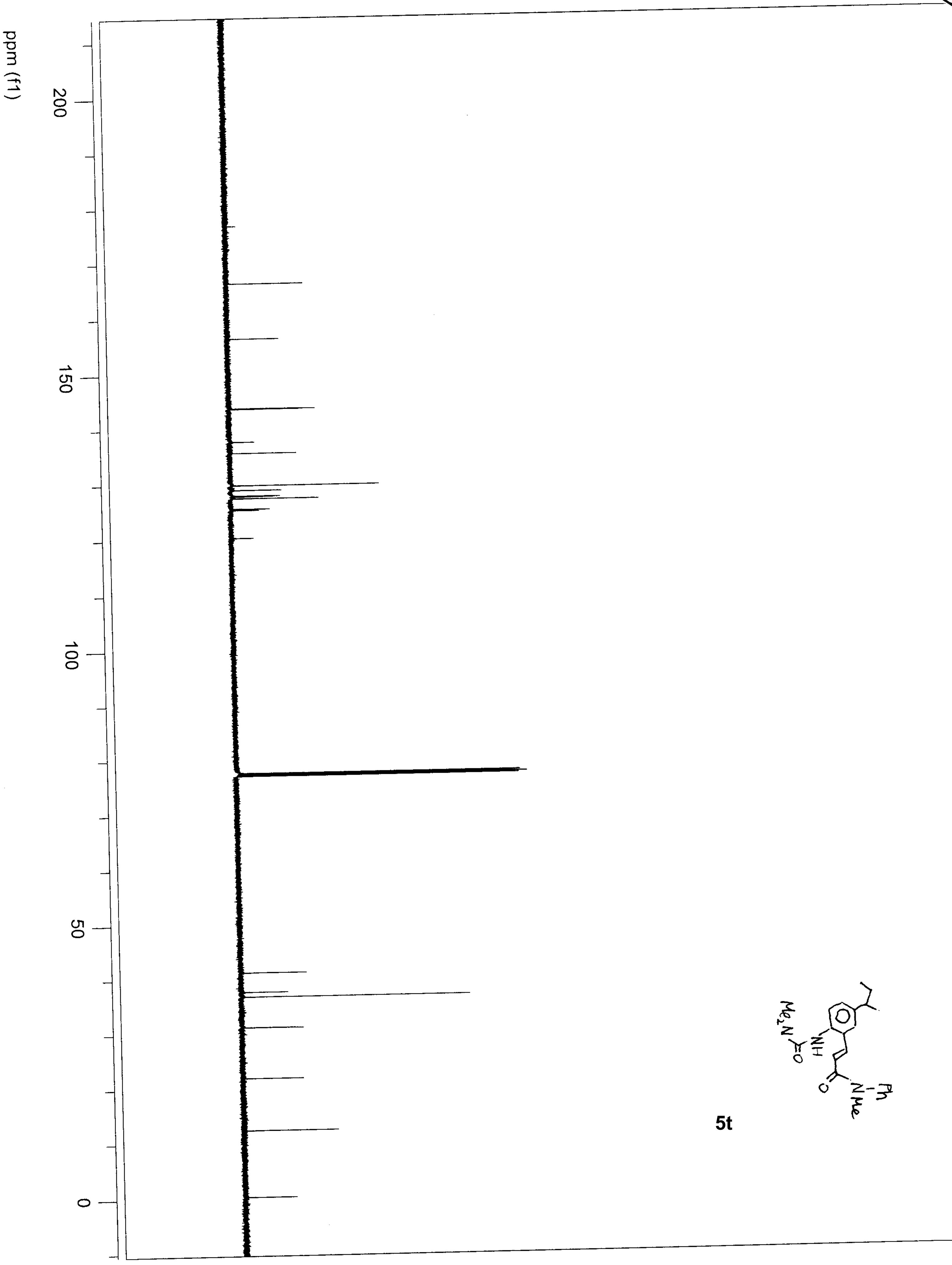

5t

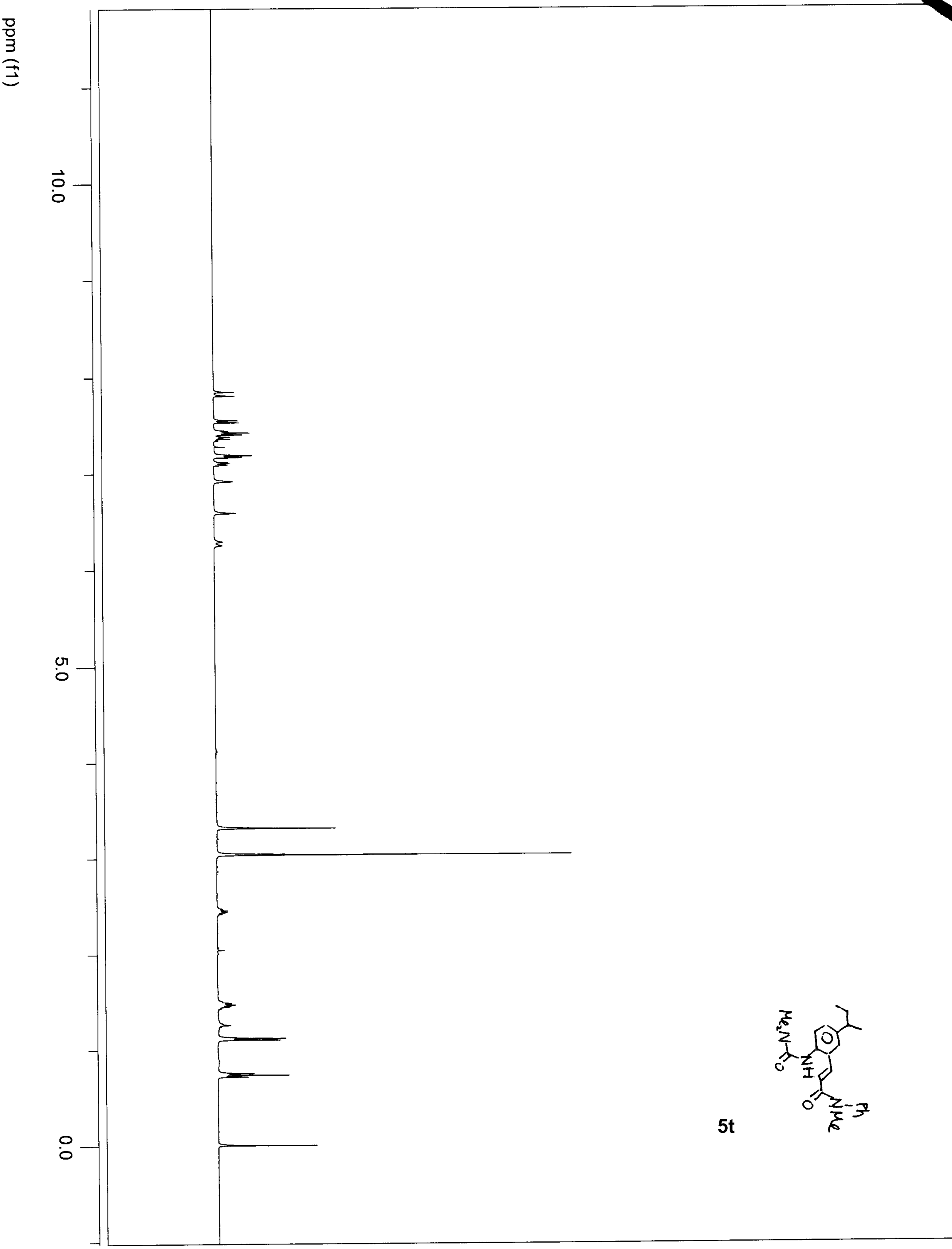
ppm (f1)
10.0
5.0
0.0
5t
Me2N
NH
O
N
Ph
Me

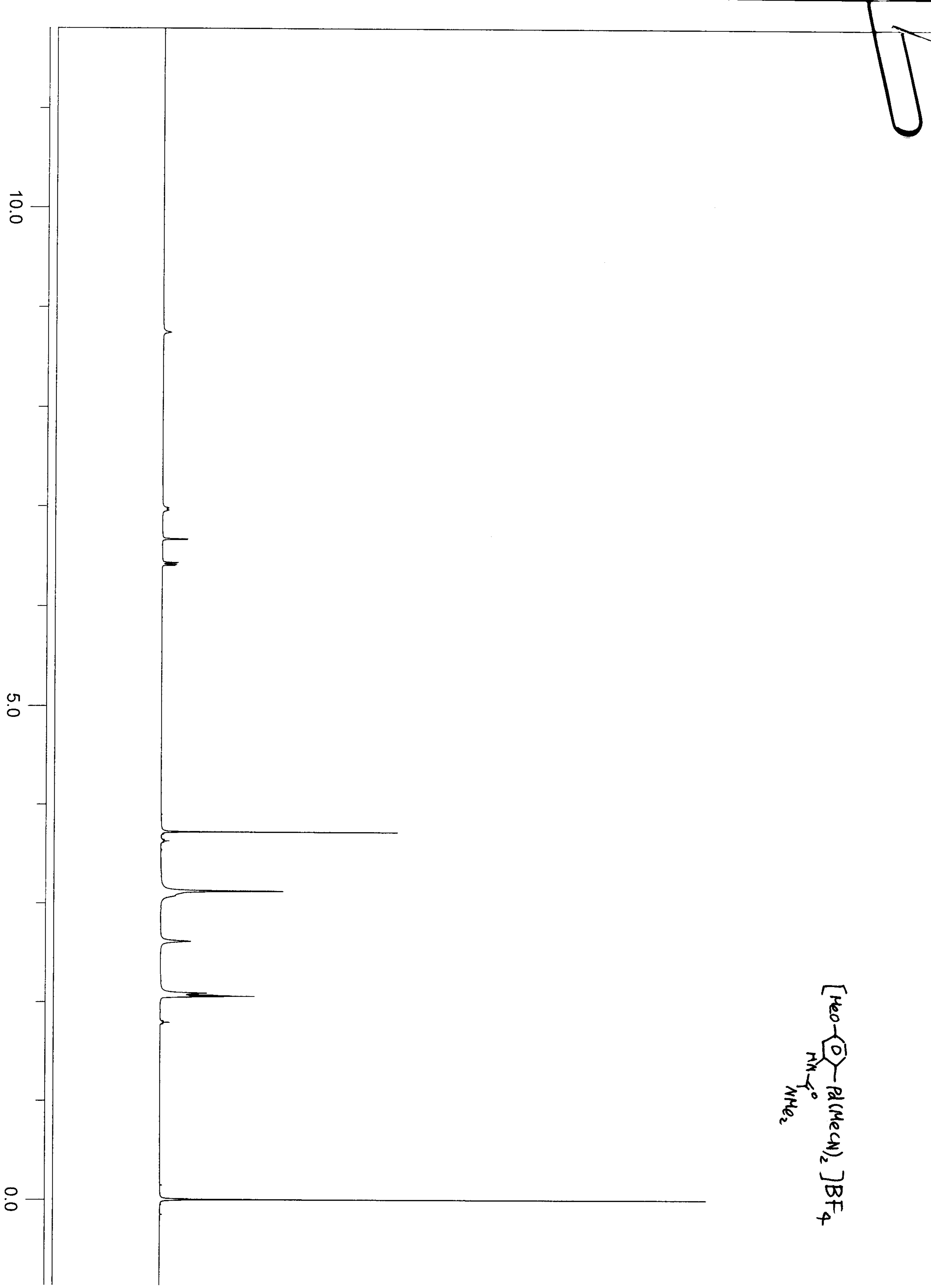
10.0
5.0
0.0

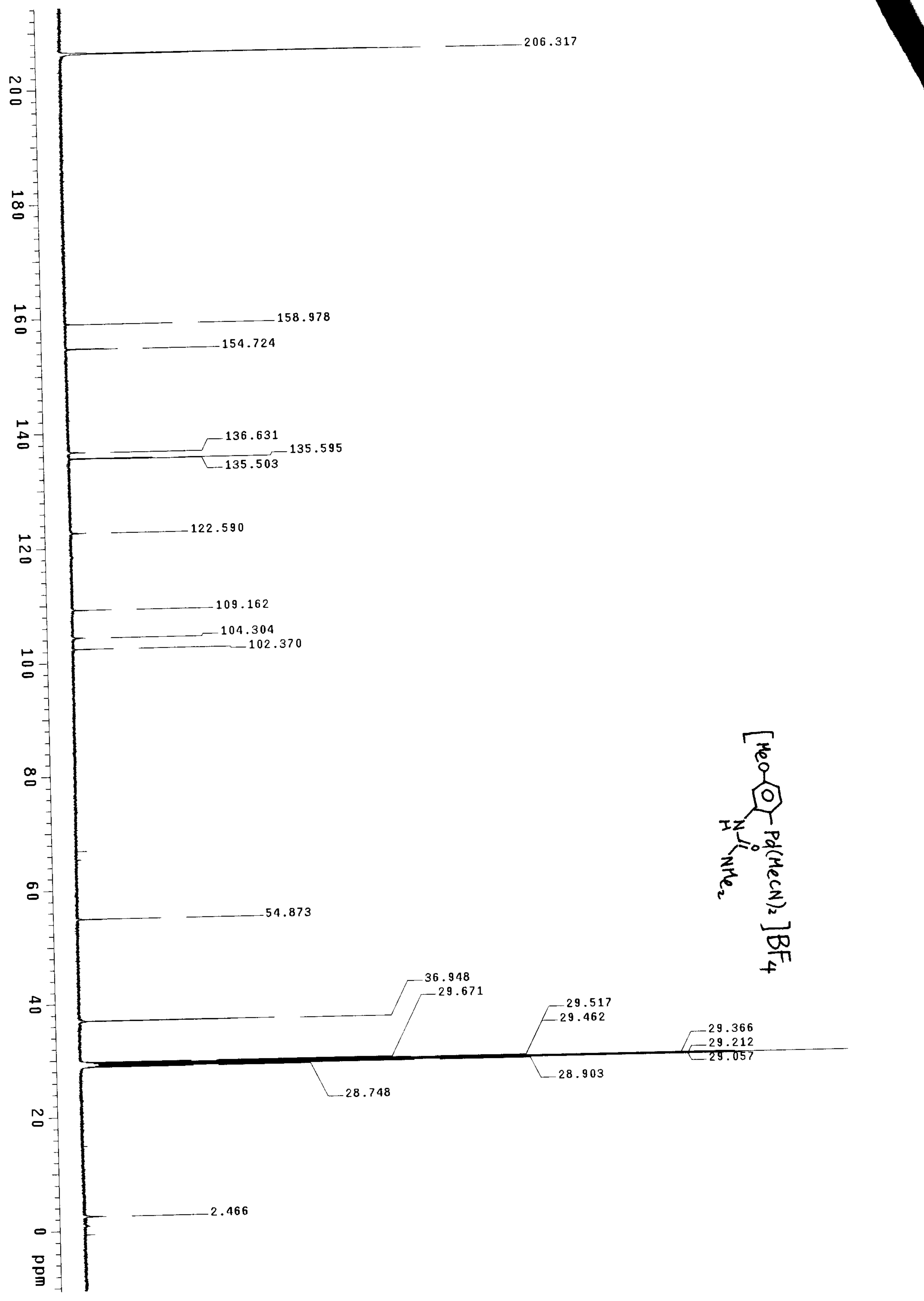
206.317
158.978
154.724
136.631
135.595
135.503
122.590
109.162
104.304
102.370
54.873
36.948
29.671
29.517
29.462
29.366
29.212
29.057
28.903
28.748
2.466
200
180
160
140
120
100
80
60
40
20
0
ppm
MeO
Pd(MeCN)2
NMe2
BF4

TN1F.swp

Data Collected on:
nmr500-inova500
Archive directory:

Sample directory:

File: F19

Pulse Sequence: s2pul
Solvent: acetone

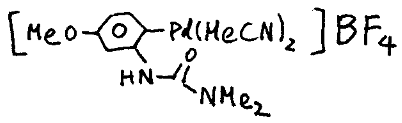

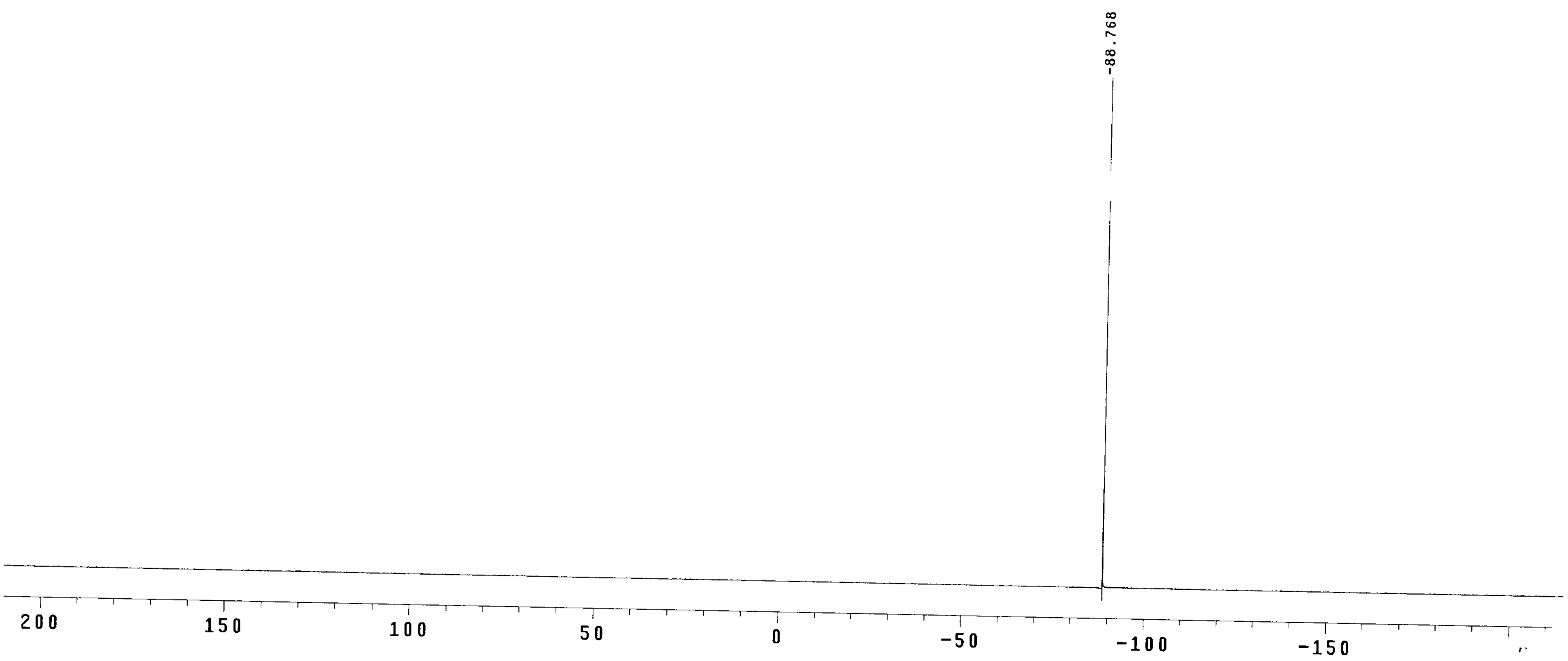

N2B.swp

Data Collected on:
nmr500-inova500
Archive directory:

Sample directory:

File: B11

Pulse Sequence: s2pul
Solvent: cdcl3

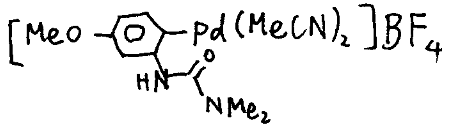

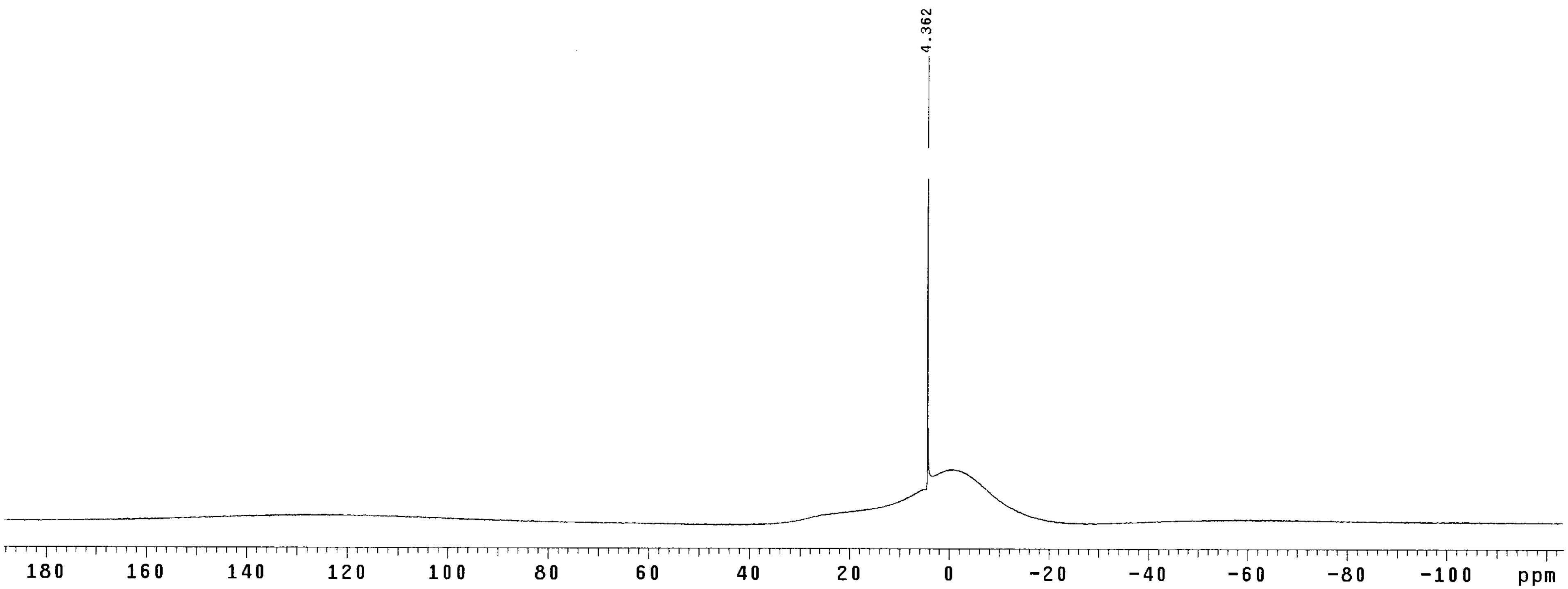

Supplement: File 1 — Experimental procedures and characterization of all new compounds. [file Beilstein_J_Org_Chem-12-1040-s001.pdf]
