# Supplementary material for: Cationic Pd(II)-catalyzed C–H activation/cross-coupling reactions at room temperature: synthetic and mechanistic studies
Source: Beilstein J Org Chem. 2016 May 20;12:1040–64. doi: 10.3762/bjoc.12.99 (PMC4902085; doi:10.3762/bjoc.12.99)
Supplement: File 3 — Crystal structure of 6 No 2. [file Beilstein_J_Org_Chem-12-1040-s003.pdf]

Supporting Information

for

Cationic Pd(II)-catalyzed C–H activation/cross-coupling reactions at room temperature: synthetic and mechanistic studies

Takashi Nishikata, Alexander R. Abela, Shenlin Huang and Bruce H. Lipshutz*

Address: Department of Chemistry & Biochemistry, University of California, Santa Barbara, CA 93106, USA

Email: lipshutz@chem.ucsb.edu

* Corresponding author

Crystal structure of 6 No 2

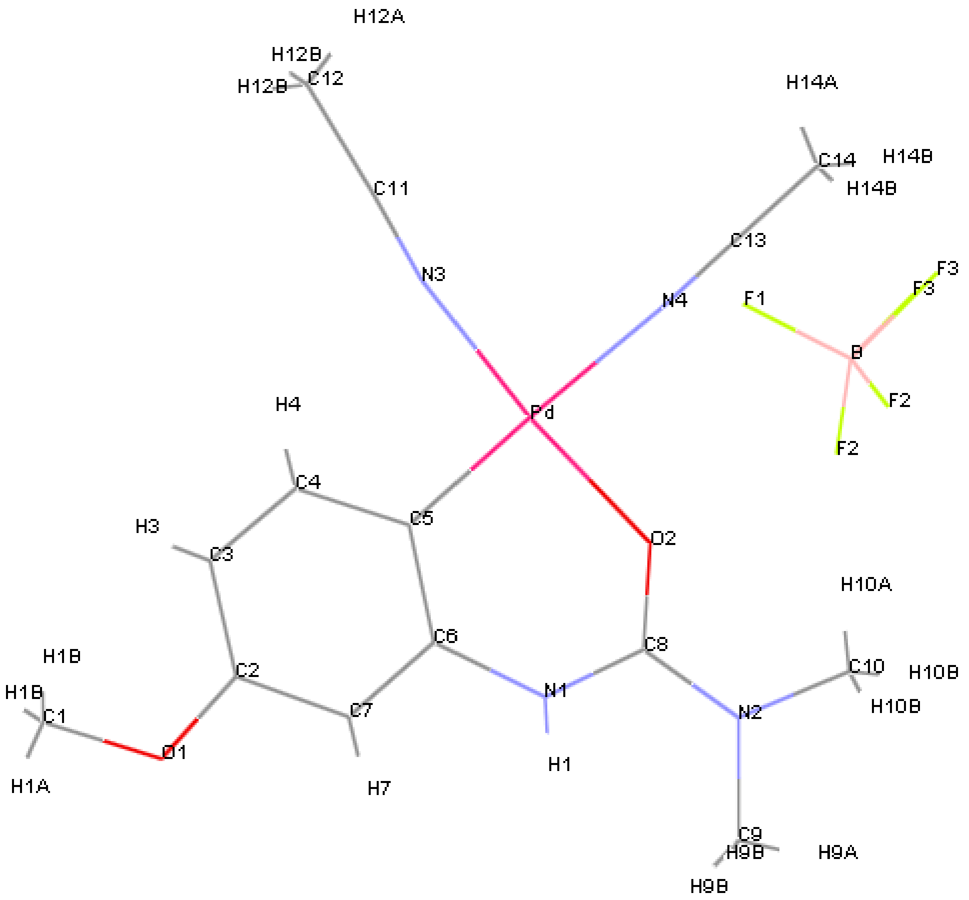

One of F atoms has disorder.

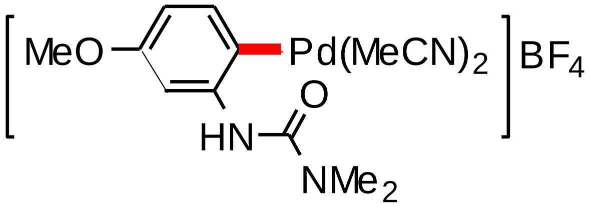

Table 1. Crystal data and structure refinement for palladacycle.

Identification code	1
Empirical formula	C14 H19 B F4 N4 O2 Pd
Formula weight	468.54
Temperature	150(2) K
Wavelength	0.71073 A
Crystal system, space group	Orthorhombic, Pnma
Unit cell dimensions	a = 25.328(5) A alpha = 90 deg.<br>b = 6.6951(13) A beta = 90 deg.<br>c = 10.795(2) A gamma = 90 deg.
Volume	1830.6(6) A^3
Z, Calculated density	4, 1.700 Mg/m^3
Absorption coefficient	1.068 mm^-1
F(000)	936
Crystal size	0.25 x 0.2 x 0.08 mm
Theta range for data collection	2.05 to 26.76 deg.
Limiting indices	-31<=h<=28, -8<=k<=8, -13<=l<=13
Reflections collected / unique	11933 / 2076 [R(int) = 0.0573]
Completeness to theta = 26.76	97.8 %
Absorption correction	Empirical
Max. and min. transmission	0.291 and 0.215
Refinement method	Full-matrix least-squares on F^2
Data / restraints / parameters	2076 / 12 / 193
Goodness-of-fit on F^2	1.345
Final R indices [I>2sigma(I)]	R1 = 0.0392, wR2 = 0.0989
R indices (all data)	R1 = 0.0455, wR2 = 0.1010
Extinction coefficient	0.0040(7)
Largest diff. peak and hole	0.789 and -0.815 e.A^-3

Table 2. Atomic coordinates (x 10^4) and equivalent isotropic displacement parameters (A^2 x 10^3) for 1.
U(eq) is defined as one third of the trace of the orthogonalized Uij tensor.

	x	y	z	U(eq)
B	7970(3)	7500	5625(6)	49(2)
C(1)	11670(2)	2500	-1426(5)	40(1)
C(2)	11080(2)	2500	302(4)	25(1)
C(3)	10630(2)	2500	-420(5)	26(1)
C(4)	10142(2)	2500	141(4)	28(1)
C(5)	10070(2)	2500	1414(4)	20(1)
C(6)	10530(2)	2500	2130(4)	23(1)
C(7)	11027(2)	2500	1582(4)	23(1)
C(8)	10137(2)	2500	4234(4)	25(1)
C(9)	10772(2)	2500	5942(5)	47(2)
C(10)	9815(2)	2500	6360(5)	34(1)
C(11)	8702(2)	2500	-273(5)	28(1)
C(12)	8364(2)	2500	-1359(5)	35(1)
C(13)	8203(2)	2500	3480(4)	33(1)
C(14)	7683(2)	2500	4028(6)	41(1)
F(1)	7950(1)	7500	4347(3)	54(1)
F(2)	8257(2)	9112(4)	6026(3)	96(1)
F(3)	7493(2)	8216(9)	6066(4)	73(2)
N(1)	10542(1)	2500	3448(4)	24(1)
N(2)	10239(2)	2500	5451(4)	31(1)
N(3)	8980(1)	2500	556(4)	29(1)
N(4)	8608(2)	2500	3057(4)	30(1)
O(1)	11589(1)	2500	-112(3)	38(1)
O(2)	9661(1)	2500	3877(3)	29(1)
Pd	9359(1)	2500	2177(1)	24(1)

Table 3. Bond lengths [A] and angles [deg] for 1.

B-F(2)	1.372(5)
B-F(2)#1	1.372(5)
B-F(1)	1.381(7)
B-F(3)#1	1.384(8)
B-F(3)	1.384(8)
C(1)-O(1)	1.433(6)
C(1)-H(1A)	0.93(6)
C(1)-H(1B)	0.96(4)
C(2)-O(1)	1.366(5)
C(2)-C(3)	1.380(6)
C(2)-C(7)	1.388(6)
C(3)-C(4)	1.377(6)
C(3)-H(3)	0.9300
C(4)-C(5)	1.387(6)
C(4)-H(4)	0.9300
C(5)-C(6)	1.399(6)
C(5)-Pd	1.980(4)
C(6)-C(7)	1.390(6)
C(6)-N(1)	1.422(6)
C(7)-H(7)	0.9300
C(8)-O(2)	1.266(5)
C(8)-N(1)	1.332(6)
C(8)-N(2)	1.340(6)
C(9)-N(2)	1.449(7)
C(9)-H(9A)	0.961(10)
C(9)-H(9B)	0.961(10)
C(10)-N(2)	1.455(6)
C(10)-H(10A)	0.958(10)
C(10)-H(10B)	0.961(10)
C(11)-N(3)	1.139(6)
C(11)-C(12)	1.451(7)
C(12)-H(12A)	0.951(10)
C(12)-H(12B)	0.952(10)

C(13)-N(4)	1.123(6)
C(13)-C(14)	1.444(7)
C(14)-H(14A)	0.956(10)
C(14)-H(14B)	0.953(10)
F(3)-F(3)#1	0.959(12)
N(1)-H(1)	0.8600
N(3)-Pd	1.995(4)
N(4)-Pd	2.127(4)
O(2)-Pd	1.987(3)

F(2)-B-F(2)#1	103.8(5)
F(2)-B-F(1)	109.6(4)
F(2)#1-B-F(1)	109.6(4)
F(2)-B-F(3)#1	128.8(5)
F(2)#1-B-F(3)#1	94.7(4)
F(1)-B-F(3)#1	108.1(5)
F(2)-B-F(3)	94.7(4)
F(2)#1-B-F(3)	128.8(5)
F(1)-B-F(3)	108.1(5)
F(3)#1-B-F(3)	40.6(5)
O(1)-C(1)-H(1A)	100(4)
O(1)-C(1)-H(1B)	110(2)
H(1A)-C(1)-H(1B)	112(3)
O(1)-C(2)-C(3)	126.5(4)
O(1)-C(2)-C(7)	114.7(4)
C(3)-C(2)-C(7)	118.9(4)
C(4)-C(3)-C(2)	119.5(5)
C(4)-C(3)-H(3)	120.3
C(2)-C(3)-H(3)	120.3
C(3)-C(4)-C(5)	123.7(4)
C(3)-C(4)-H(4)	118.2
C(5)-C(4)-H(4)	118.2
C(4)-C(5)-C(6)	116.0(4)
C(4)-C(5)-Pd	122.1(3)
C(6)-C(5)-Pd	121.9(3)
C(7)-C(6)-C(5)	121.2(4)

C(7)-C(6)-N(1)	114.0(4)
C(5)-C(6)-N(1)	124.8(4)
C(2)-C(7)-C(6)	120.8(4)
C(2)-C(7)-H(7)	119.6
C(6)-C(7)-H(7)	119.6
O(2)-C(8)-N(1)	122.7(4)
O(2)-C(8)-N(2)	118.9(4)
N(1)-C(8)-N(2)	118.4(4)
N(2)-C(9)-H(9A)	100(4)
N(2)-C(9)-H(9B)	117(3)
H(9A)-C(9)-H(9B)	107.2(15)
N(2)-C(10)-H(10A)	110(3)
N(2)-C(10)-H(10B)	109.6(19)
H(10A)-C(10)-H(10B)	107.9(14)
N(3)-C(11)-C(12)	177.9(5)
C(11)-C(12)-H(12A)	112(3)
C(11)-C(12)-H(12B)	113(3)
H(12A)-C(12)-H(12B)	109.7(15)
N(4)-C(13)-C(14)	179.7(5)
C(13)-C(14)-H(14A)	111(4)
C(13)-C(14)-H(14B)	110(3)
H(14A)-C(14)-H(14B)	108.9(15)
F(3)#1-F(3)-B	69.7(3)
C(8)-N(1)-C(6)	128.3(4)
C(8)-N(1)-H(1)	115.8
C(6)-N(1)-H(1)	115.8
C(8)-N(2)-C(9)	122.6(4)
C(8)-N(2)-C(10)	121.2(4)
C(9)-N(2)-C(10)	116.2(4)
C(11)-N(3)-Pd	170.5(4)
C(13)-N(4)-Pd	177.4(4)
C(2)-O(1)-C(1)	117.4(4)
C(8)-O(2)-Pd	130.3(3)
C(5)-Pd-O(2)	91.97(15)
C(5)-Pd-N(3)	94.16(16)
O(2)-Pd-N(3)	173.87(14)

C(5)-Pd-N(4)	178.02(16)
O(2)-Pd-N(4)	86.05(14)
N(3)-Pd-N(4)	87.82(15)

Symmetry transformations used to generate equivalent atoms:

#1 x,-y+3/2,z

Table 4. Anisotropic displacement parameters (A^2 x 10^3) for 1.
The anisotropic displacement factor exponent takes the form:
-2 pi^2 [h^2 a*^2 U11 + ... + 2 h k a* b* U12]

	U11	U22	U33	U23	U13	U12
B	37(3)	72(5)	38(4)	0	-3(3)	0
C(1)	33(3)	63(4)	24(3)	0	2(2)	0
C(2)	23(2)	27(2)	25(2)	0	2(2)	0
C(3)	19(2)	39(2)	20(2)	0	-3(2)	0
C(4)	22(2)	35(2)	26(3)	0	-8(2)	0
C(5)	17(2)	18(2)	23(2)	0	-2(2)	0
C(6)	27(2)	23(2)	18(2)	0	-3(2)	0
C(7)	19(2)	26(2)	23(2)	0	-6(2)	0
C(8)	29(2)	20(2)	26(2)	0	1(2)	0
C(9)	31(3)	85(5)	25(3)	0	-4(2)	0
C(10)	40(3)	34(2)	29(3)	0	6(2)	0
C(11)	17(2)	30(2)	38(3)	0	1(2)	0
C(12)	29(3)	37(3)	39(3)	0	-7(2)	0
C(13)	26(3)	44(3)	28(3)	0	-4(2)	0
C(14)	21(3)	48(3)	53(4)	0	8(2)	0
F(1)	42(2)	87(2)	33(2)	0	-12(1)	0
F(2)	159(3)	56(2)	72(2)	-2(1)	-60(2)	-8(2)
F(3)	39(3)	128(7)	53(3)	-1(3)	9(2)	11(2)
N(1)	23(2)	28(2)	20(2)	0	-4(2)	0
N(2)	30(2)	44(2)	18(2)	0	2(2)	0
N(3)	20(2)	28(2)	39(2)	0	-2(2)	0
N(4)	23(2)	34(2)	34(2)	0	-4(2)	0
O(1)	20(2)	70(2)	22(2)	0	2(1)	0
O(2)	22(2)	41(2)	24(2)	0	1(1)	0
Pd	18(1)	27(1)	27(1)	0	-1(1)	0

Table 5. Hydrogen coordinates (x 10^4) and isotropic displacement parameters (A^2 x 10^3) for 1.

	x	y	z	U(eq)
H(3)	10657	2500	-1279	31
H(4)	9844	2500	-363	33
H(7)	11327	2500	2078	27
H(1A)	12040(30)	2500	-1460(60)	61(19)
H(9A)	10690(20)	2500	6814(18)	120(40)
H(10A)	9479(11)	2500	5950(40)	53(18)
H(12A)	8000(7)	2500	-1140(40)	52(17)
H(14A)	7410(20)	2500	3410(50)	90(30)
H(1B)	11525(14)	1310(50)	-1790(40)	38(10)
H(9B)	10986(14)	1350(40)	5780(30)	65(14)
H(10B)	9836(11)	1310(30)	6860(30)	31(9)
H(12B)	8436(13)	1420(50)	-1910(30)	73(16)
H(14B)	7640(19)	1350(50)	4540(40)	120(20)
H(1)	10851	2500	3779	28

Table 6. Torsion angles [deg] for 1.

O(1)-C(2)-C(3)-C(4)	180.0
C(7)-C(2)-C(3)-C(4)	0.0
C(2)-C(3)-C(4)-C(5)	0.0
C(3)-C(4)-C(5)-C(6)	0.0
C(3)-C(4)-C(5)-Pd	180.0
C(4)-C(5)-C(6)-C(7)	0.0
Pd-C(5)-C(6)-C(7)	180.0
C(4)-C(5)-C(6)-N(1)	180.0
Pd-C(5)-C(6)-N(1)	0.0
O(1)-C(2)-C(7)-C(6)	180.0
C(3)-C(2)-C(7)-C(6)	0.0
C(5)-C(6)-C(7)-C(2)	0.0
N(1)-C(6)-C(7)-C(2)	180.0
F(2)-B-F(3)-F(3)#1	-150.7(4)
F(2)#1-B-F(3)-F(3)#1	-38.8(4)
F(1)-B-F(3)-F(3)#1	97.0(2)
O(2)-C(8)-N(1)-C(6)	0.0
N(2)-C(8)-N(1)-C(6)	180.0
C(7)-C(6)-N(1)-C(8)	180.0
C(5)-C(6)-N(1)-C(8)	0.0
O(2)-C(8)-N(2)-C(9)	180.0
N(1)-C(8)-N(2)-C(9)	0.0
O(2)-C(8)-N(2)-C(10)	0.0
N(1)-C(8)-N(2)-C(10)	180.0
C(12)-C(11)-N(3)-Pd	180.000(3)
C(14)-C(13)-N(4)-Pd	180.0(3)
C(3)-C(2)-O(1)-C(1)	0.0
C(7)-C(2)-O(1)-C(1)	180.0
N(1)-C(8)-O(2)-Pd	0.0
N(2)-C(8)-O(2)-Pd	180.0
C(4)-C(5)-Pd-O(2)	180.0
C(6)-C(5)-Pd-O(2)	0.0

C(4)-C(5)-Pd-N(3)	0.0
C(6)-C(5)-Pd-N(3)	180.0
C(4)-C(5)-Pd-N(4)	180.000(4)
C(6)-C(5)-Pd-N(4)	0.000(4)
C(8)-O(2)-Pd-C(5)	0.0
C(8)-O(2)-Pd-N(3)	180.0
C(8)-O(2)-Pd-N(4)	180.0
C(11)-N(3)-Pd-C(5)	180.0
C(11)-N(3)-Pd-O(2)	0.000(1)
C(11)-N(3)-Pd-N(4)	0.0
C(13)-N(4)-Pd-C(5)	180.000(7)
C(13)-N(4)-Pd-O(2)	180.000(6)
C(13)-N(4)-Pd-N(3)	0.000(6)

Symmetry transformations used to generate equivalent atoms:

#1 x,-y+3/2,z
